# Unveiling non-Hermitian band structures with non-Bloch supercells

**DOI:** 10.1038/s41467-026-77152-5

**Published:** 2026-08-26

**Authors:** Jia-Xin Zhong, Jing Lin, Kai Chen, Jing Lu, Kun Ding, Yun Jing

**Affiliations:** 1 Graduate Program in Acoustics, The Pennsylvania State University, University Park, PA 16802 USA; 2 Department of Physics, State Key Laboratory of Surface Physics, and Key Laboratory of Micro and Nano Photonic Structures (Ministry of Education), Fudan University, Shanghai, 200438 China; 3 Key Laboratory of Modern Acoustics and Institute of Acoustics, Nanjing University, Nanjing, 210093 China; 4 NJU-Horizon Intelligent Audio Lab, Horizon Robotics, Beijing, 100094 China

**Keywords:** Condensed-matter physics, Acoustics

## Abstract

Real-valued band structures underpin the analysis of periodic Hermitian systems and are well established experimentally. By contrast, non-Hermitian systems feature complex band structures, in which energy and momentum acquire imaginary parts, enabling phenomena that defy conventional Bloch theory. Mapping these complex bands—relating complex momentum to complex energy—and identifying their associated eigenstates is essential yet remains challenging. Here, we introduce a non-Bloch supercell framework that addresses this challenge by treating non-Bloch Fermi points (NBFPs) as key observables and disentangling the roles of real and imaginary parts of momentum. As the non-Hermitian counterparts of Fermi surfaces, NBFPs provide experimentally accessible markers of point‑gap topology and open‑boundary behavior. Experimentally, our approach combines an exponent-flattening protocol with twisted boundary conditions, enabling system-size-independent control of imaginary momentum and preserving high-resolution sampling in real momentum. Implemented in programmable one- and two-dimensional acoustic crystals, our framework acquires momentum-resolved complex energy surfaces and biorthogonal eigenstates by Green’s function measurements. The resulting non‑Hermitian band structures, including complex momentum and energy and Berry curvature distributions, accurately predict open‑boundary spectra and eigenstates, validated in independent open‑geometry experiments. Our work delivers a broadly applicable experimental toolkit for exploring non-Hermitian band geometry and topology in diverse platforms with programmable couplings.

## Abstract (web summary)

Non-Hermitian band structures enable unusual phenomena but remain difficult to map beyond one dimension. Here, the authors develop a general non-Bloch supercell framework to resolve complex momentum, energy, eigenstates, and topology, demonstrated in one- and two-dimensional acoustic crystals.

## Introduction

The standard quantum framework uses Hermitian operators, yielding real spectra and probability conservation in closed systems. Most physical systems are effectively open, motivating non‑Hermitian descriptions^1–3^ in which energies are complex: real parts set resonant frequencies, while imaginary parts encode decay or gain^4,5^. Complex spectra support unique gap and degeneracy types, known as point gaps^6,7^ and exceptional points^2,8–12^, underpinning phenomena such as non-Hermitian skin effect (NHSE)^13–20^ and sensitivity enhancement^8,21,22^. While these effects have been observed across diverse platforms^15–17,23–27^, directly accessing complex spectra and eigenstates remains important because key features of non-Hermitian systems (e.g., van Hove singularities^28^ and distributions of Berry curvature^7,29^) are difficult to infer reliably from emergent phenomena alone. Recent measurements of system-wide Green’s functions (two-point correlators) have enabled direct reconstructions of complex eigenenergies and eigenstates^17,25,30^.

**Fig. 1:**
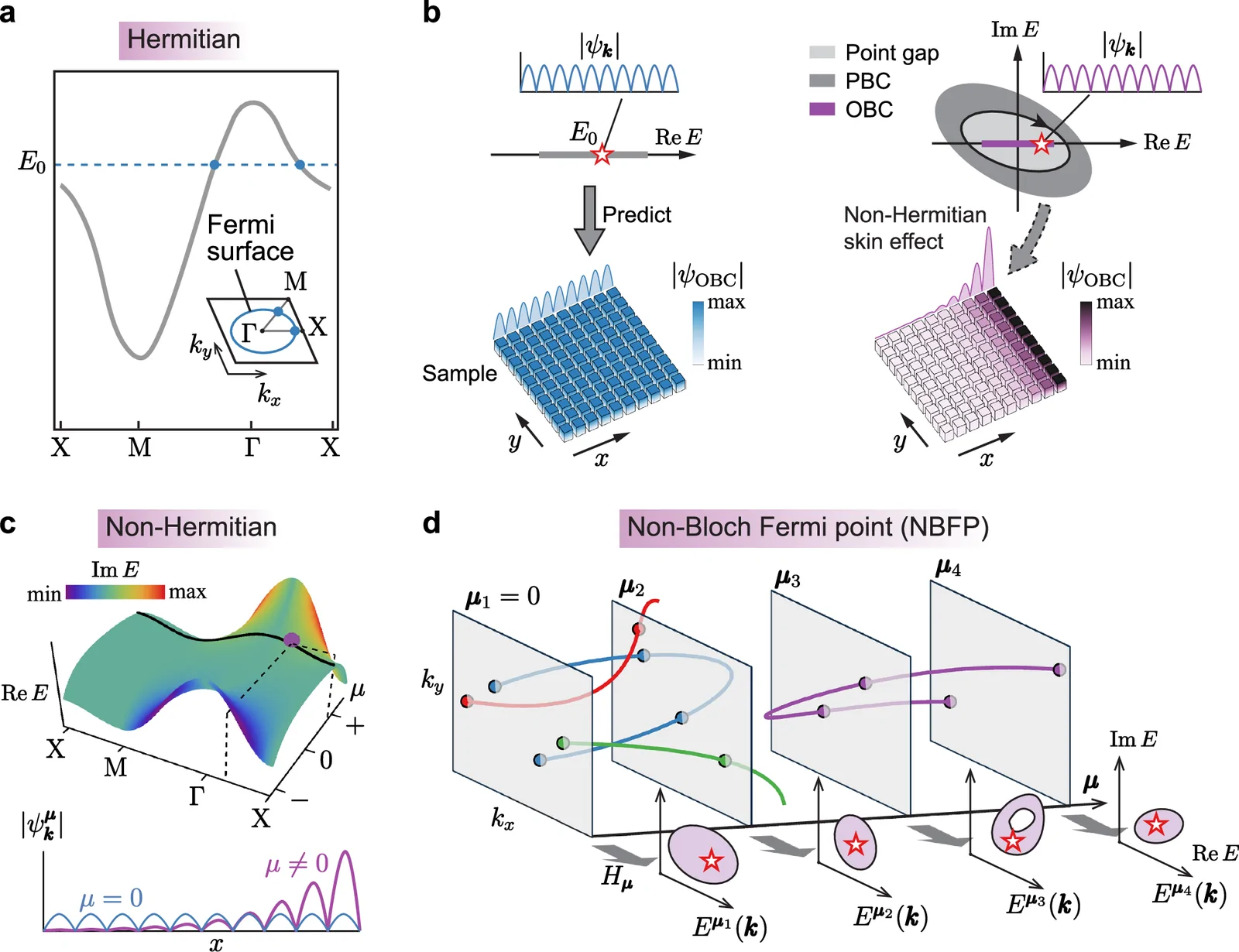
Non-Hermitian band structures and Fermi points. **a** Representative Hermitian band structure. Only one high-symmetry line of a square lattice is shown, and the inset depicts a typical Fermi surface. **b** PBC-OBC (Periodic/Open Boundary Condition) correspondence in a Hermitian (left) and non-Hermitian (right) system. Dark gray lines and areas represent PBC spectra. In Hermitian scenarios, a representative OBC eigenstate at ${E}_{0}$ (bottom) can be analyzed by its corresponding Fermi surface and Bloch eigenstates. Whereas, it breaks down in non-Hermitian scenarios due to the existence of point gaps, which are tied to the skin effect (bottom). **c** Representative non-Hermitian band structure. The surface (top) displays real parts of eigenenergy under analytic continuation ${{\boldsymbol{k}}}{{\to }}{{\boldsymbol{k}}}-i{{\boldsymbol{\mu }}}$, while its color encodes imaginary parts of eigenenergy. A representative non-Bloch eigenstate is shown in the bottom panel. **d** Evolution of NBFPs with ${{\boldsymbol{\mu }}}$. The insets sketch the map ${H}_{{{\boldsymbol{\mu }}}}:{{\boldsymbol{k}}}\mapsto E$ from BZ to complex spectra ${E}^{{{\boldsymbol{\mu }}}}({{\boldsymbol{k}}})$ for various ${{\boldsymbol{\mu }}}$. For a chosen complex energy ${E}_{b}$ (star), the preimages are indicated by colorful spheres in the BZ and define the NBFPs.

From a band-theory perspective, complex energies compel a generalization in which the full energy-momentum dispersion *E*(***k***) and the associated eigenstates are equally essential^1,2,7,14^. Figure 1a displays a representative Hermitian band structure computed under periodic boundary conditions (PBCs). The Fermi surface, defined by $\left\{{{\boldsymbol{k}}}\right|E\left({{\boldsymbol{k}}}\right)={E}_{0}{{\boldsymbol{\}}}}$ (Fig. 1a, blue lines in the inset), provides essential information of low-energy excitations, scattering channels, and transport properties. Absent nontrivial boundary topology or strong surface reconstruction, Fermi surfaces can predict bulk spectra and eigenstates under open boundary conditions (OBCs), the experimentally relevant situation since real samples always have finite boundaries (Fig. 1b, bottom-left). By contrast, the emergence of point gaps under PBC (Fig. 1b, top-right) in non-Hermitian systems leads to exponential localization of bulk eigenstates at the boundary (Fig. 1b, bottom-right), the celebrated NHSE, breaking the conventional PBC-OBC correspondence^7,13,14^. Real-momentum band structures then become insufficient, and a faithful description requires complex-valued momenta ${{\boldsymbol{k}}}-i{{\boldsymbol{\mu }}}$ (Fig. 1c, top), yielding modes of the form ${e}^{{{\boldsymbol{\mu }}}{{\cdot }}{{\boldsymbol{r}}}}$ times Bloch waves (Fig. 1c, bottom)—non-Bloch eigenstates^6,31,32^. The non-Hermitian band structure, defined as the mapping from complex momentum to complex E together with its non-Bloch eigenstates, therefore encodes all the information needed to predict open-boundary behavior, making direct experimental access crucial.

Standard momentum-resolved spectroscopies, such as angle-resolved photoemission spectroscopy^33,34^, inelastic neutron scattering^35,36^, and photon-induced near-field electron microscopy^37^, measure spectral functions at real momenta and provide excellent tools to probe band structures and residing Fermi surfaces. However, in non-Hermitian systems, the imaginary part of the momentum, together with complex energies, imparts exponential decay to eigenstates, rapidly pushing observables below the experimental noise floor. The resulting irreversible information loss in spectral functions erodes the predictive power that conventional Fermi surfaces would otherwise afford^38,39^. Consequently, probing non-Hermitian band structures requires both (i) strategies to mitigate information loss due to exponential attenuation and (ii) the ability to actively set and vary the imaginary parts of momentum ***μ***. Experimental reconstruction of non-Hermitian band structures in arbitrary dimensions has therefore remained an open challenge.

Here, we introduce a non-Bloch supercell framework that enables direct experimental reconstruction of non-Hermitian band structures. By identifying non-Bloch Fermi points (NBFPs) that serve as non-Hermitian counterparts of Fermi surfaces in complex momentum space, we show that ***μ*** control should be decoupled from the sampling of Bloch phase ***k***. We thus implement an exponent-flattening protocol that imprints ***k*** via twisted boundary conditions (TBCs) at the supercell scale while independently tuning ***μ*** through engineered intercell hoppings. This provides scale-free control of ***μ*** (independent of supercell size) alongside high-resolution control of ***k***, addressing the two intrinsic experimental challenges outlined above. Combined with Green’s-function-based reconstruction of complex eigenenergies and eigenstates^30^, this framework yields non-Hermitian band structures, encompassing the high-accuracy complex momentum-energy map and non-Bloch eigenstates. Implemented in programmable one-dimensional (1D) and two-dimensional (2D) acoustic crystals, it resolves NBFPs, reveals complex eigenenergy features such as point-gap patterns and spectral potentials, and reconstructs Berry curvature distributions. Taking the supercell-reconstructed non-Hermitian bands as the large-*N* reference, we experimentally characterize and verify the finite-size OBC spectra and eigenstates in independent open-boundary acoustic crystals. Notably, in two dimensions, boundary geometry selectively activates specific Fermi points, a behavior absent in lower dimensions.

## Results

### Non-Bloch supercell framework

Since experimental reconstruction of non-Hermitian band structures requires active control of the imaginary momentum ***μ***, we revisit non-Bloch band theory to build up a theoretical formulation that cleanly separates the roles of ***k*** and ***μ***. Analytically continuing the Bloch factor$\,{{\boldsymbol{\beta }}}\equiv {e}^{i{{\boldsymbol{k}}}+{{\boldsymbol{\mu }}}}$ gives a ***μ***-parameterized non-Bloch Hamiltonian ${H}_{{{\boldsymbol{\mu }}}}\left({{\boldsymbol{k}}}\right)\equiv H\left({{\boldsymbol{\beta }}}\right)$, with $({{\boldsymbol{k}}},{{\boldsymbol{\mu }}})\in {{\rm{BZ}}}\times {{\mathbb{R}}}^{d}$ and d being the system dimension. This yields spectra ${E}^{{{\boldsymbol{\mu }}}}({{\boldsymbol{k}}})$ and eigenstates ${\psi }_{{{\boldsymbol{k}}}}^{{{\boldsymbol{\mu }}}}$, as exhibited in Fig. 1d. Relative to the PBC case (${{\boldsymbol{\mu }}}=0$), ***μ*** acts as an independent deformation parameter, decoupled from ***k***, that reshapes both eigenvalues and eigenstates. Accordingly (Fig. 1d, insets), we view the non-Bloch Hamiltonian as a mapping ${H}_{{{\boldsymbol{\mu }}}}:{{\boldsymbol{k}}}\to E$ from Brillouin zone (BZ) to complex spectrum with attached eigenstates. For a fixed ***μ***, its image is ${E}^{{{\boldsymbol{\mu }}}}\left({{\boldsymbol{k}}}\right)$ and the set $\Sigma=\{{E}^{{{\boldsymbol{\mu }}}}\left({{\boldsymbol{k}}}\right)\in {\mathbb{C}}{{\boldsymbol{\mu }}}\in {{\mathbb{R}}}^{d},k\in {{\rm{BZ}}}\}$ defines the spectral manifold. Conversely, for a chosen ${E}_{b}$ (Fig. 1d, star) and given ${H}_{{{\boldsymbol{\mu }}}}$, the preimage on the BZ is the roots of $\det [E-{H}_{{{\boldsymbol{\mu }}}}\left({{\boldsymbol{k}}}\right)]=0$ (spheres in Fig. 1d). We coin them as NBFPs, the generalization of Fermi surfaces to non-Hermitian settings, and denote

1 $${K}_{{{\boldsymbol{\mu }}}}({E}_{b})=\{{{\boldsymbol{k}}}\in {{\rm{BZ}}}|\det [{E}_{b}-{H}_{{{\boldsymbol{\mu }}}}({{\boldsymbol{k}}})]=0\}.$$

This treatment preserves the full information of $H\left({{\boldsymbol{\beta }}}\right)$ while explicitly disentangling ***μ*** from ***k***.

Compared with Hermitian Fermi surfaces, NBFPs differ in both their dimension and their ambient parameter space. A conventional Fermi surface resides in a *d*-dimensional momentum space and has dimension d−1. By contrast, NBFPs inhabit a 2*d*-dimensional space of complex momenta and have dimension 2d−2. The doubling of the ambient dimension reflects the complexification of momentum, whereas the −2 offset arises from the two independent constraints from $\det [E-{H}_{{{\boldsymbol{\mu }}}}\left({{\boldsymbol{k}}}\right)]=0$. For instance, when d=2, NBFPs constitute hypersurfaces in a four-dimensional space and manifest as isolated points in the BZ for a fixed ***μ***, as shown in Fig. 1d. It hints that NBFPs can merge and annihilate, as will be demonstrated later. At the same time, NBFPs share a key commonality with conventional Fermi surfaces: both are tightly linked to open‑boundary spectra and eigenstates^13,32^. Accordingly, we will show that NBFPs serve as experimentally accessible markers of point‑gap topology and, crucially, can be directly probed and examined in open‑boundary experiments.

**Fig. 2:**
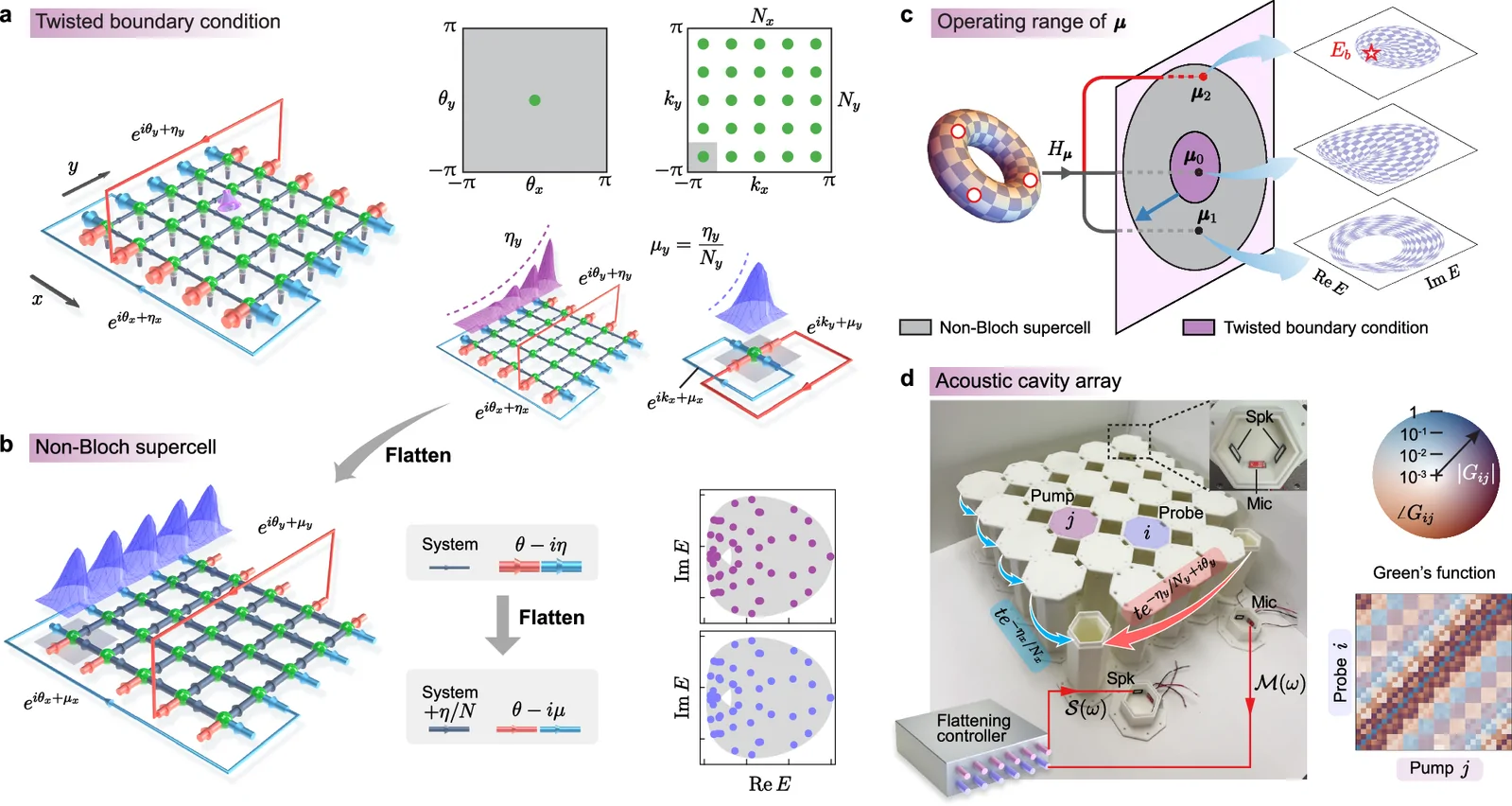
Non-Bloch supercell framework for reconstructing non-Hermitian band structures in experiment. **a** Schematic illustration of a supercell with analytically continued TBCs, ${{\boldsymbol{\theta }}}-i{{\boldsymbol{\eta }}}$ (left). The red and blue thick lines highlight the hopping modifications induced by ${{\boldsymbol{\theta }}}$ and ${{\boldsymbol{\eta }}}$ at the supercell boundaries, while the thin gray lines denote hoppings of the system under consideration. The first and second rows on the right depict the equivalence of the real and imaginary momentum components, respectively, between complex-continuation TBCs (first column) and primitive-cell level non-Bloch analysis (second column). **b** The non-Bloch supercell framework. The left panel displays the non-Bloch supercell with ${e}^{i{{\boldsymbol{\theta }}}}$ applied at the boundaries (red and blue thin lines) and the flattening factor ${e}^{{{\boldsymbol{\eta }}}}$ distributed across the entire lattice at each intercell hopping (gray lines). Purple circles in the top-right panel show spectra obtained using complex-continuation TBCs, which coincide with those from the non-Bloch supercell framework (blue circles in the bottom-right panel). ${N}_{x}$ (${N}_{y}$) denotes the number of unit cells along the x (y) direction. **c** Operating range of μ with TBCs (purple region) and non-Bloch supercells (gray region). The preimages of ${E}_{b}$ are NBFPs an**d** shown by red circles in the BZ. **d** Experimental setups. The left panel shows the cavity array, each equipped with speakers (Spk) and microphones (Mic), enabling controllable hopping terms via the flattening controller. The right panel depicts a representative Green’s function measurement with the pump and probe marked on the left, rendering complex spectra and eigenstates observable experimentally.

Establishing such a connection experimentally requires a protocol that links non-Bloch analysis with OBC physics. Following the Hermitian recipe, a system with TBCs serves as a practical tool for establishing such a connection. However, generalizing this approach to non-Hermitian systems is nontrivial due to the presence of imaginary momentum. To clarify these challenges, we start with an ${N}_{x}\times {N}_{y}$ supercell with TBCs and assess the tunability of ***μ*** afforded by the boundary twists. A twisted-phase factor ${e}^{i{\theta }_{x,y}}$ with ${\theta }_{x,y}\in (-\pi,\pi ]$ is applied across each supercell boundary (Fig. 2a). This construction folds the primitive-cell band structure (Supplementary Note 1), usually computed over the BZ parameterized by ${{\boldsymbol{k}}}=\left({k}_{x},{k}_{y}\right)$. In systems with translational symmetry, varying the twist phases is equivalent to shifting the Bloch momenta and therefore provides a standard approach for probing band structures in finite-size systems. Increasing the supercell size increases the number of BZ sampling points along each direction, ${N}_{{k}_{m}}\propto {N}_{m}$ for m=x,y (Fig. 2a, top-right). In non-Hermitian systems, extending the twist phase into the complex plane naturally provides access to the imaginary part of momentum and the associated non-Bloch band structure, i.e., the analytic continuation ${\theta }_{m}\to {\theta }_{m}-i{\eta }_{m}$ indicates multiplying the inter-supercell hopping along direction ${{\rm{m}}}$ by ${{e}^{{\eta }_{m}}e}^{{i\theta }_{m}}$, which is equivalent to the configuration shown in the bottom-right panels of Fig. 2a. It preserves ${N}_{{k}_{m}}\propto {N}_{m}$ because sampling points still follow Fig. 2a for any ${\eta }_{m}$, while providing access to imaginary parts of the primitive-cell momenta ${{\boldsymbol{\mu }}}=\left({\mu }_{x},{\mu }_{y}\right)$ as ${\mu }_{m}={\eta }_{m}/{N}_{m}$. At this stage, we have established the equivalence between complex-continuation TBCs and the non-Bloch description.

In the experiment, the finite dynamic range of the programmable hopping amplitudes, defined as the finite interval over which the hopping magnitude can be reliably tuned before saturation or noise-floor limitations are reached, inevitably constrains the operating range R of ${\eta }_{m}$, denoted as $R\left({\eta }_{m}\right)$, further indicating $R\left({\mu }_{m}\right)\propto {N}_{m}^{-1}$ (Methods). Thus, reducing the supercell size enlarges $R\left({\mu }_{m}\right)$ but decreases ${N}_{{k}_{m}}$. In short, *k*-resolution and *μ*-range are interlocked: larger supercells yield finer k sampling but a narrower accessible *μ* range, and vice versa. Although ***μ*** can be tuned via complex-continued TBCs, this k-μ trade-off motivates a framework that decouples a wide *μ*-range from high *k*-resolution.

We now introduce a non-Bloch supercell scheme to resolve the k-μ trade-off. Concretely, we promote ***μ*** from an implicit imaginary part of momentum to an independent, experimentally controllable deformation parameter, and encode it into real-space hoppings via a non-unitary transformation ${D}_{{{\boldsymbol{\mu }}}}$^40,41^. For a tight-binding Hamiltonian $H={\sum }_{i,j}{{{\boldsymbol{t}}}}_{i-j}{c}_{i}^{{\dagger} }{c}_{j}$ with unit-cell indices i,j and hopping matrices ${{{\boldsymbol{t}}}}_{i-j}$, such a transformation ${D}_{{{\boldsymbol{\mu }}}}:\,{\widetilde{c}}_{i}^{{\dagger} }\to {{{\rm{e}}}}^{-{{\boldsymbol{\mu }}}\cdot {{{\boldsymbol{R}}}}_{i}}{c}_{i}^{{\dagger} },{\widetilde{c}}_{i}\to {{{\rm{e}}}}^{{{\boldsymbol{\mu }}}\cdot {{{\boldsymbol{R}}}}_{i}}{c}_{i}$ yields

2 $${H}_{{{\boldsymbol{\mu }}}}={D}_{{{\boldsymbol{\mu }}}}^{-1}H{D}_{{{\boldsymbol{\mu }}}}=\mathop{\sum }\limits_{i,j}{\widetilde{{{\boldsymbol{t}}}}}_{i-j}{\widetilde{c}}_{i}^{{\dagger} }{\widetilde{c}}_{j}\,,\,{\widetilde{{{\boldsymbol{t}}}}}_{i-j}={{{\boldsymbol{t}}}}_{i-j}{{{\rm{e}}}}^{-{{\boldsymbol{\mu }}}\cdot ({{{\boldsymbol{R}}}}_{i}-{{{\boldsymbol{R}}}}_{j})},$$

where ${{{\boldsymbol{R}}}}_{i}$ and ${{{\boldsymbol{R}}}}_{j}$ are Bravais lattice vectors. Thus, as shown in Fig. 2b, ***μ*** imprints itself into each intercell hopping (i≠j) and flattens ${e}^{{\eta }_{m}}$ across the entire supercell, resulting in a supercell under TBCs without analytic continuation. This maintains dense k-sampling ${N}_{{k}_{m}}\propto {N}_{m}$ while making the operating μ-range independent of supercell size, i.e., $R\left({\mu }_{m}\right)\propto {N}_{m}^{0}$. Hence, the k-μ trade-off is removed, and we designate it as the non-Bloch supercell. Compared to Fig. 2a, the non-Bloch supercell (Fig. 2b) redistributes the boundary exponential factor ${\eta }_{m}$ uniformly over internal hoppings—a procedure we call exponent flattening—thereby substantially enlarging the experimentally accessible *μ*-range (Fig. 2c). In this way, the exponential decay problem that plagues non-Hermitian measurements is converted into a coupling-modulation problem. The experimentally achievable range of ${{\boldsymbol{\mu }}}$ is then determined solely by the dynamic range of controllable couplings, rather than by the system size or dimensionality. As a result, the scheme is scale-free, dimension-independent, and readily portable across experimental platforms.

We implement the non-Bloch supercell in an active acoustic lattice (Fig. 2d, left). The platform comprises an array of acoustic cavities coupled by programmable active elements, including microphones and loudspeakers, under real-time control (Methods and Supplementary Note 2). The tight-binding analogy of such networks makes them ideal for engineering intricate hoppings and non-Hermitian terms^23,42–44^. Using a custom controller, we realize the required hopping profile for each $({{\boldsymbol{k}}},{{\boldsymbol{\mu }}})$. By point-exciting individual sites and recording the full multi-site response, we construct the Green’s function ${G}_{{ij}}(E)$ and its diagonalization yields complex eigenenergies ${E}^{{{\boldsymbol{\mu }}}}\left({{\boldsymbol{k}}}\right)$ and eigenstates ${\psi }_{{{\boldsymbol{k}}}}^{{{\boldsymbol{\mu }}}}$ (Fig. 2d, right) (Methods), enabling high-resolution experimental reconstructions of non-Bloch band structures by the proposed non-Bloch supercell framework. Its robustness against intrinsic acoustic losses and other uncertainties is guaranteed by a high signal-to-noise ratio and dynamic range with which the Green’s function is measured (Methods).

### 1D non-Hermitian band structures, non-Bloch Fermi points, and validations

**Fig. 3:**
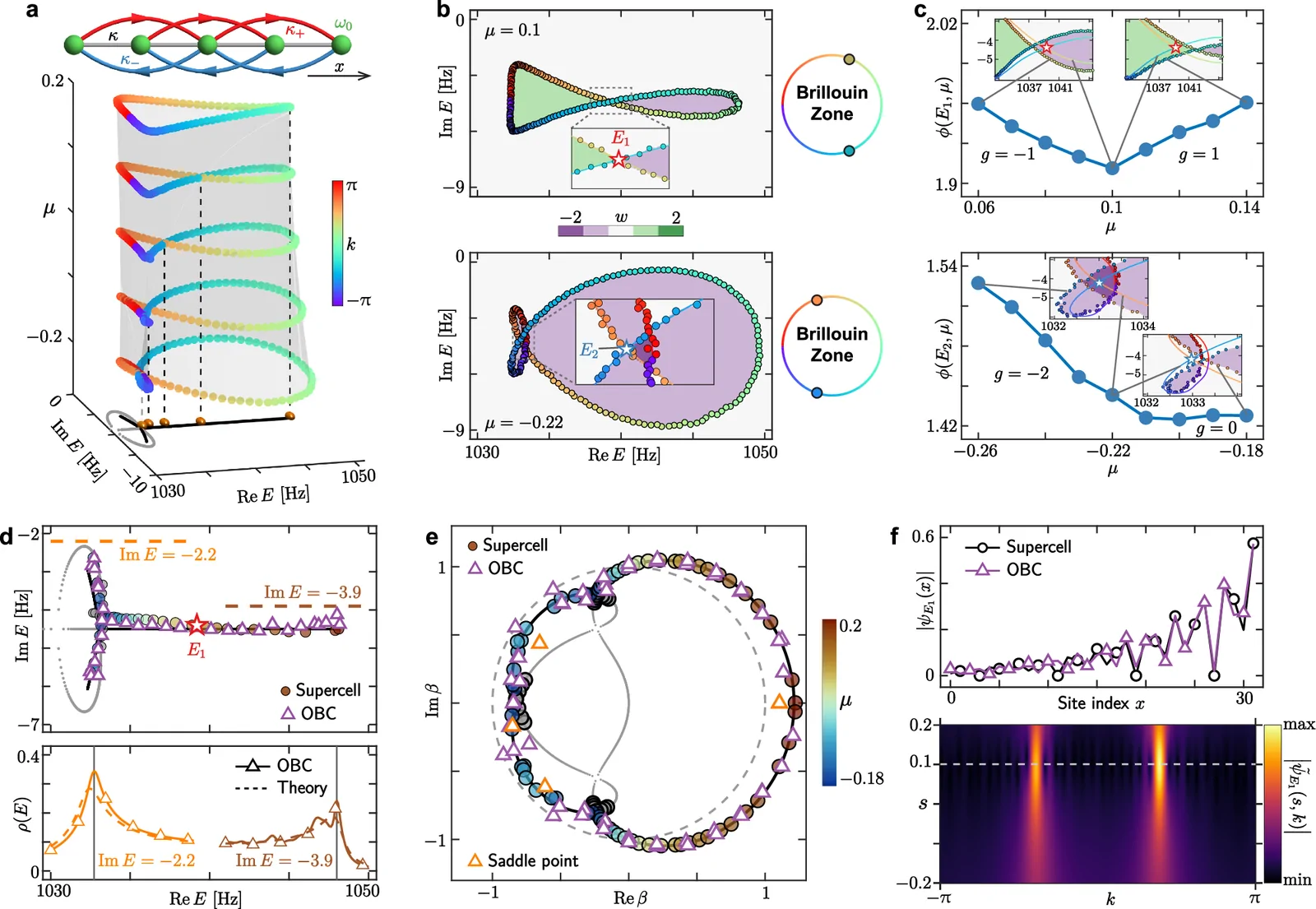
Experimentally determined 1D non-Hermitian band structures and OBC verifications. **a** Non-Hermitian band structures with both complex-valued energies and wavenumbers. The used lattice model is ${H}_{1D}\left(\beta \right)={\omega }_{0}+\kappa \left(\beta+{\beta }^{-1}\right)+{\kappa }_{+}{\beta }^{-2}+{\kappa }_{-}{\beta }^{2}$, as sketched in the top. The gray surface is from theory, while the colored spheres display experimental results with k encoded by color. The bottom plane shows the projection of five self-intersection points, and the black line indicates the theoretical OBC spectra. **b** Measured point-gap patterns $w\left(E,\mu \right)$ for μ=0.1 (top) and μ=−0.22 (bottom). The marker colors represent k, while region shading encodes the value of w for E within each region. Insets highlight point-gap patterns near spectral self-intersection points ${E}_{1}=\left(1039.2-4.4i\right){\mbox{Hz}}$ and ${E}_{2}=\left(1033.0-4.2i\right){\mbox{Hz}}$. Their corresponding k preimages in the BZ, NBFPs, are shown by filled circles in the right panels. **c** Measured spectral potential $\phi \left(E,\mu \right)$ for ${E}_{1}$ (top) and ${E}_{2}$ (bottom). A V-shaped cusp in ϕ indicates E is an OBC eigenenergy (top); otherwise, E is excluded from the OBC spectrum (bottom). Insets display evolutions of point-gap patterns with μ. OBC spectra (**d**, top) and generalized Brillouin zones (**e**) determined from non-Bloch supercells (circles) and measured in a finite OBC lattice (triangles). The bottom panel in **d** shows the density of states, $\rho \left(E\right)$, versus ${\mbox{Re}}\,E$ at ${\mbox{Im}}{E}=-2.2\,{\mbox{Hz}}$ (orange) and ${\mbox{Im}}\,E=-3.9\,{\mbox{Hz}}$ (brown). The feature in the density of states arises from the saddle points highlighted in **e**. Black and gray dots represent theoretical predictions. **f** Measured real-space wavefunction ${\psi }_{{E}_{1}}\left(x\right)$ (triangles, top) and *s*-resolved momentum-space wavefunction ${\widetilde{\psi }}_{{E}_{1}}(s,k)$ (bottom) from open boundary lattices. Hotspots of ${\widetilde{\psi }}_{{E}_{1}}(s,k)$ at s=0.1 agree with NBFPs determined in **b**. Filled circles on the top depict wavefunctions determined from the non-Bloch supercell measurement. The parameters used are ${\omega }_{0}=\left(1038-4i\right){\mbox{Hz}}$, $\kappa=4{\mbox{Hz}}$, ${\kappa }_{+}=2{\mbox{Hz}}$, and ${\kappa }_{-}=0.4{\mbox{Hz}}$. The supercell is composed of ${N}_{x}=25$ unit cells, while the size of the OBC system is L=32.

We demonstrate the capability of the proposed framework from a 1D non-Hermitian lattice (Fig. 3a, top). The measured ${E}^{\mu }\left(k\right)$ for selected *μ* are shown by filled circles with their color encoding *k* (Fig. 3a, bottom). The excellent agreement with the calculated spectral manifold Σ (gray surface) unveils our non-Bloch supercell framework in reconstructing non-Bloch band structures.

As discussed, the spectral manifold contains the necessary information to determine OBC spectra and eigenstates. To make such predictions from Fig. 3a, we analyze point gaps via eigenenergy winding numbers along a direction $\hat{n}$

3 $${w}_{\hat{n}}\left(E,{{\boldsymbol{\mu }}};{{{\boldsymbol{k}}}}_{{{\perp }}}\right)=\frac{1}{2\pi i}\oint {\partial }_{{k}_{\hat{n}}}\log \det \left[E-{H}_{{{\boldsymbol{\mu }}}}\left({k}_{\hat{n}};{{{\boldsymbol{k}}}}_{{{\perp }}}\right)\right]d{k}_{\hat{n}},\,$$

where E is the reference energy. Equation (3) applies in arbitrary dimensions. We decompose ${{\boldsymbol{k}}}$ into ${k}_{\hat{n}}$ and ${{{\boldsymbol{k}}}}_{{{\perp }}}$, where ${k}_{\hat{n}}={{\boldsymbol{k}}}{{\cdot }}\hat{n}$ selects the direction along which we probe point gaps, while ${{{\boldsymbol{k}}}}_{{{\perp }}}$ parametrizes the remaining (d−1)-dimensional section of BZ and only requires linear independence of $\hat{n}$. For the 1D system in Fig. 3a, $\hat{n}$ points along *x* and ${{{\boldsymbol{k}}}}_{{{\perp }}}$ is absent. Since ${w}_{\hat{n}}$ reflect topological structures of eigenenergies relative to E, its value depends on E across the complex plane. We select two μ-slices from Fig. 3a and utilize the measured ${E}^{\mu }\left(k\right)$ to calculate $w\left(E,\mu \right)$, as shown by region shading colors in Fig. 3b. We refer to these $w\left(E,\mu \right)$ distributions as point-gap patterns. Self-intersections in these patterns anchor parameter pairs $\left({E}_{1},\mu=0.1\right)$ and $\left({E}_{2},\mu=-0.22\right)$, indicating that multiple NBFPs exist at this energy (Fig. 3b, inset), which permits non-Bloch standing waves^6,13,32^. However, self-intersection alone does not automatically belong to the OBC spectra because the OBC eigenenergy requires that it resides in point gaps for all ${{\boldsymbol{\mu }}}$, namely, $\forall {{\boldsymbol{\mu }}}$, $\exists \left(\hat{n},{{{\boldsymbol{k}}}}_{{{\perp }}}\right)$ such that ${w}_{\hat{n}}\left(E,{{\boldsymbol{\mu }}};{{{\boldsymbol{k}}}}_{{{\perp }}}\right)\ne 0$ (Supplementary Note 3)^41,45^.

This criterion, referred to here as the point-gap criterion, can be stated as the persistence of the point-gap topology for all values of ${{\boldsymbol{\mu }}}$, providing a necessary condition for an energy to belong to the OBC spectrum for a particular geometry in the large-N limit. We define ${{{\rm{\sigma }}}}_{{{\rm{OBC}}}}$ as the geometry-independent spectral envelope, i.e., the union of OBC spectra over all admissible boundary geometries in the large-N limit, and the OBC spectrum for a particular geometry must be a subset of ${\sigma }_{{{\rm{OBC}}}}$. The point-gap criterion states that, for an energy E to belong to this geometry-independent envelope, the relevant point-gap topology must persist for all values of ${{\boldsymbol{\mu }}}$^41,45^. Equivalently, if there exists an admissible ${{\boldsymbol{\mu }}}$ for which the corresponding point-gap topology becomes trivial, then E fails this necessary condition and is excluded from ${\sigma }_{{{\rm{OBC}}}}$. This criterion alone does not determine the OBC spectrum for a specific boundary geometry; additional constraints, including boundary matching and geometric details, further select the geometry-dependent subset of ${\sigma }_{{{\rm{OBC}}}}$.

Although our non-Bloch supercell implementation broadens the experimentally accessible dynamic range of ${{\boldsymbol{\mu }}}$, access to ${{\boldsymbol{\mu }}}$ remains finite. What matters is not exhaustive coverage of all ${{\boldsymbol{\mu }}}$, but whether the accessible range spans the finite ${{\boldsymbol{\mu }}}$ window that determines the OBC spectrum. Once this condition is met, the measured non-Bloch band structures contain all the information required to predict OBC behaviors. To diagnose such a ${{\boldsymbol{\mu }}}$ window, we therefore employ spectral potential^41^, which can be computed alongside eigenenergy winding numbers for a measured discrete set of eigenenergies ${E}_{v}^{{{\boldsymbol{\mu }}}}$ with $v=1,\ldots,{N}_{E}$,

4 $$\phi \left(E,{{\boldsymbol{\mu }}}\right)=\int {\left(\frac{{dk}}{2\pi }\right)}^{d}\log \left|\det \left[E-{H}_{{{\boldsymbol{\mu }}}}\left({{\boldsymbol{k}}}\right)\right]\right|=\frac{1}{{N}_{E}}\mathop{\sum }\limits_{v}\log \left|E-{E}_{v}^{{{\boldsymbol{\mu }}}}\right|,$$

which quantifies the logarithmic distance from E to the spectrum. The spectral potential in the ${{\boldsymbol{\mu }}}$ space is convex, and the point-gap criterion then implies that $\phi \left(E,\mu \right)$ for an OBC eigenenergy has a minimum without a plateau nearby. The absence of a plateau for ${E}_{1}$ (Fig. 3c, top) indicates that ${E}_{1}$ is always in point gaps at nearby μ, as reflected by the insets. In contrast, $\phi \left(E,\mu \right)$ for ${E}_{2}$ exhibits a plateau, and ${E}_{2}$ can then lie outside the point gap (Fig. 3c, bottom). The point-gap criterion tells that ${E}_{1}$ (${E}_{2}$) is (not) an OBC eigenenergy. Since the spectral potential helps pin down the OBC spectral range, identifying its minimum and local behaviors becomes essential for judging a plateau. It motivates analyzing its μ-gradient ${{\boldsymbol{g}}}={\nabla }_{{{\boldsymbol{\mu }}}}\phi \left(E,{{\boldsymbol{\mu }}}\right)$, which relates to the averaged winding via ${{\boldsymbol{g}}}{{\cdot }}\hat{n}={\left\langle {w}_{\hat{n}}\left(E,{{\boldsymbol{\mu }}};{{{\boldsymbol{k}}}}_{{{\perp }}}\right)\right\rangle }_{{{{\boldsymbol{k}}}}_{{{\perp }}}}$, where ${\langle \cdot \rangle }_{{{{\boldsymbol{k}}}}_{{{\perp }}}}$ denotes averaging over ${{{\boldsymbol{k}}}}_{{{\perp }}}$ (Methods and Supplementary Note 4). Thus, the point-gap criterion requires that ${{\boldsymbol{g}}}$ near the minimum of $\phi \left(E,\mu \right)$ for an OBC eigenenergy is nonzero (Fig. 3c). Leveraging ${{\boldsymbol{g}}}$, spectral potentials provide an efficient experimental handle for predicting OBC behavior.

Using such a recipe, the determined OBC spectra for this 1D model are shown as colored circles in Fig. 3d, and the corresponding NBFPs are depicted in Fig. 3e, which are precisely the generalized Brillouin zone (Supplementary Note 5). The μ values of NBFPs are determined by the minimum of $\phi \left(E,\mu \right)$, while their k values are then read from the corresponding measured ${E}^{\mu }\left(k\right)$. To validate, we measure system-wide Green’s functions of an L=32 chain, and the extracted OBC spectra (triangles, Fig. 3d) closely match the supercell prediction. As a key spectral feature, the density of states $\rho \left(E\right)$ exhibits non-Bloch van Hove singularities at energies corresponding to saddle points on generalized Brillouin zones. The bottom panel of Fig. 3d exhibits $\rho \left(E\right)=\frac{1}{L}{\mbox{Im}}{\sum }_{v}{\left(E-{E}_{v}\right)}^{-1}$ computed from experimental OBC eigenenergies ${E}_{v}$ for ${\mbox{Im}}{E}=-2.2\,{\mbox{Hz}}$ (orange) and $-3.9\,{\mbox{Hz}}$ (brown). Evident peaks near ${\mbox{Re}}{E}=1033\,{\mbox{Hz}}$ and $1048\,{\mbox{Hz}}$ align with the energy of saddle points identified by our supercell method (dashed lines).

Besides spectra, the eigenstates obtained by our supercell framework can be examined against OBC eigenstates from Green’s function measurements^30^. We select ${E}_{1}=(1039.2-4.4i){{\rm{Hz}}}$ from Fig. 3b (red star in Fig. 3d) and plot its OBC eigenstate profile $\left|{\psi }_{{E}_{1}}\right|$ (Fig. 3f, triangles), which is clearly localized. For extracting its localization length, we compute the s-resolved Fourier-Laplace transform of ${\psi }_{E}\left({{\boldsymbol{r}}}\right)$

5 $${\widetilde{\psi }}_{E}\left({{\boldsymbol{s}}},{{\boldsymbol{k}}}\right)=\int d{{\boldsymbol{r}}}\left[{{{\rm{e}}}}^{-{{\boldsymbol{s}}}\cdot {{\boldsymbol{r}}}}{\psi }_{E}\left({{\boldsymbol{r}}}\right)\right]{{{\rm{e}}}}^{-i{{\boldsymbol{k}}}\cdot {{\boldsymbol{r}}}},$$

where ${{\boldsymbol{s}}}\in {{\mathbb{R}}}^{d}$ and ${{\boldsymbol{s}}}{{\boldsymbol{+}}}i{{\boldsymbol{k}}}$ is the Laplace variable. The amplitude $\left|{\widetilde{\psi }}_{{E}_{1}}(s,k)\right|$ (Fig. 3f, bottom) clearly shows two hotspots when s=0.1, matching μ values corresponding to the spectral potential minimum from Fig. 3c. To examine further, Fig. 3f displays ${{{\rm{e}}}}^{+0.1x}{\sum }_{k}\exp ({ikx})$ (circles), where $k\in {K}_{\mu=0.1}\left({E}_{1}\right)$ are two NBFPs determined from Fig. 3b. Excellent agreement in the eigenstates further corroborates our supercell framework for acquiring non-Hermitian band structures.

As demonstrated in Fig. 3e–f, one can perform a Fourier-Laplace transformation of the OBC eigenstate to extract μ and the corresponding NBFPs (triangles in Fig. 3e). However, OBC measurements are themselves subject to the intrinsic boundary sensitivity of non-Hermitian systems. Because the imaginary part of momentum induces exponential attenuation, the signal can rapidly fall below the experimental noise floor, leading to increasing information loss as the system size grows. For example, in Fig. 3e, the values extracted from OBC eigenstates (triangles) exhibit noticeable deviations from the analytical solutions (solid lines), particularly near saddle-point regions where the system becomes highly sensitive. In contrast, our non-Bloch supercell measurements (filled color circles) remain substantially more robust due to the μ-flattening mechanism.

**Fig. 4:**
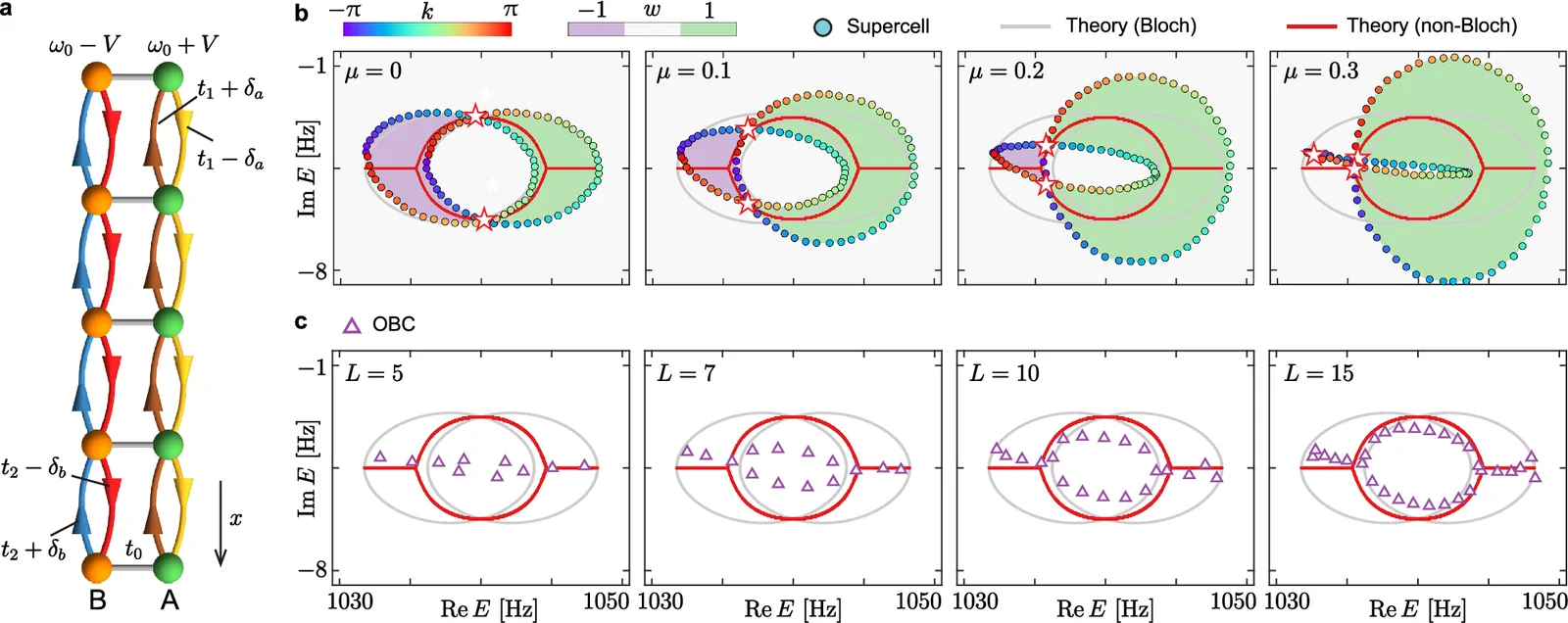
Distinction between the non-Bloch supercell framework and OBC measurements of the critical skin effect. **a** Two-chain model supporting the critical skin effect. The lattice model is $${H}_{{{\rm{CSE}}}}\left(\beta \right)=\left(\begin{array}{cc}{g}_{A}(\beta ) & {t}_{0}\\ {t}_{0} & {g}_{B}(\beta )\end{array}\right)$$ with ${g}_{A}(\beta )={\omega }_{0}+{t}_{A}^{+}\beta+{t}_{A}^{-}{\beta }^{-1}+V$ and ${g}_{B}(\beta )={\omega }_{0}+{t}_{B}^{+}\beta+{t}_{B}^{-}{\beta }^{-1}-V$. The two chains are labeled as A and B. **b** Measured ${E}^{\mu }\left(k\right)$ for four selected values of μ. Filled circles with their color encoding k are from the non-Bloch supercell framework, and their self-intersections (marked as stars) indicate the OBC spectrum in the large-*N* limit, as highlighted by the red stars. **c** Measured OBC spectra (triangles) for increasing system sizes L (left to right). For comparison, the measured PBC spectra are shown as circles. In both **b** and **c**, the gray lines indicate the theoretical PBC spectrum, and the red solid lines are the OBC spectrum in the large-N limit obtained from the generalized Brillouin zone analysis. The red stars in **b** are in excellent agreement with these generalized Brillouin zone analyses, validating the non-Bloch supercell framework. By contrast, the OBC spectra in **c** deviate from the large-N limit, revealing the strong finite-size dependence characteristic of the critical skin effect. The parameters used are ${\omega }_{0}=\left(1040-4.5i\right){{\rm{Hz}}}$, ${t}_{0}=1\,{{\rm{Hz}}}$, ${t}_{1}=3\,{{\rm{Hz}}}$, ${\delta }_{A}=1\,{{\rm{Hz}}}$, ${\delta }_{B}=-1\,{{\rm{Hz}}}$, and $V=2\,{{\rm{Hz}}}$. The supercell is composed of ${N}_{x}=5$ unit cells.

More importantly, beyond the issue of measurement instability, OBC-based approaches are not universally reliable for probing non-Hermitian band structures, particularly in systems where finite-size effects are significant, such as those exhibiting the critical skin effect (Fig. 4). Figure 4a shows a representative multiband model supporting the critical skin effect and multiple point gaps^46^. The non-Bloch supercell framework successfully captures the non-Bloch behavior in the large-N limit (Fig. 4b), which is difficult to access using conventional OBC measurements (Fig. 4c). This result demonstrates that the non-Bloch supercell framework overcomes the intrinsic limitations of OBC-based approaches and enables direct access to non-Bloch information in the large-N limit.

### Boundary-activated non-Bloch Fermi points in 2D lattices

**Fig. 5:**
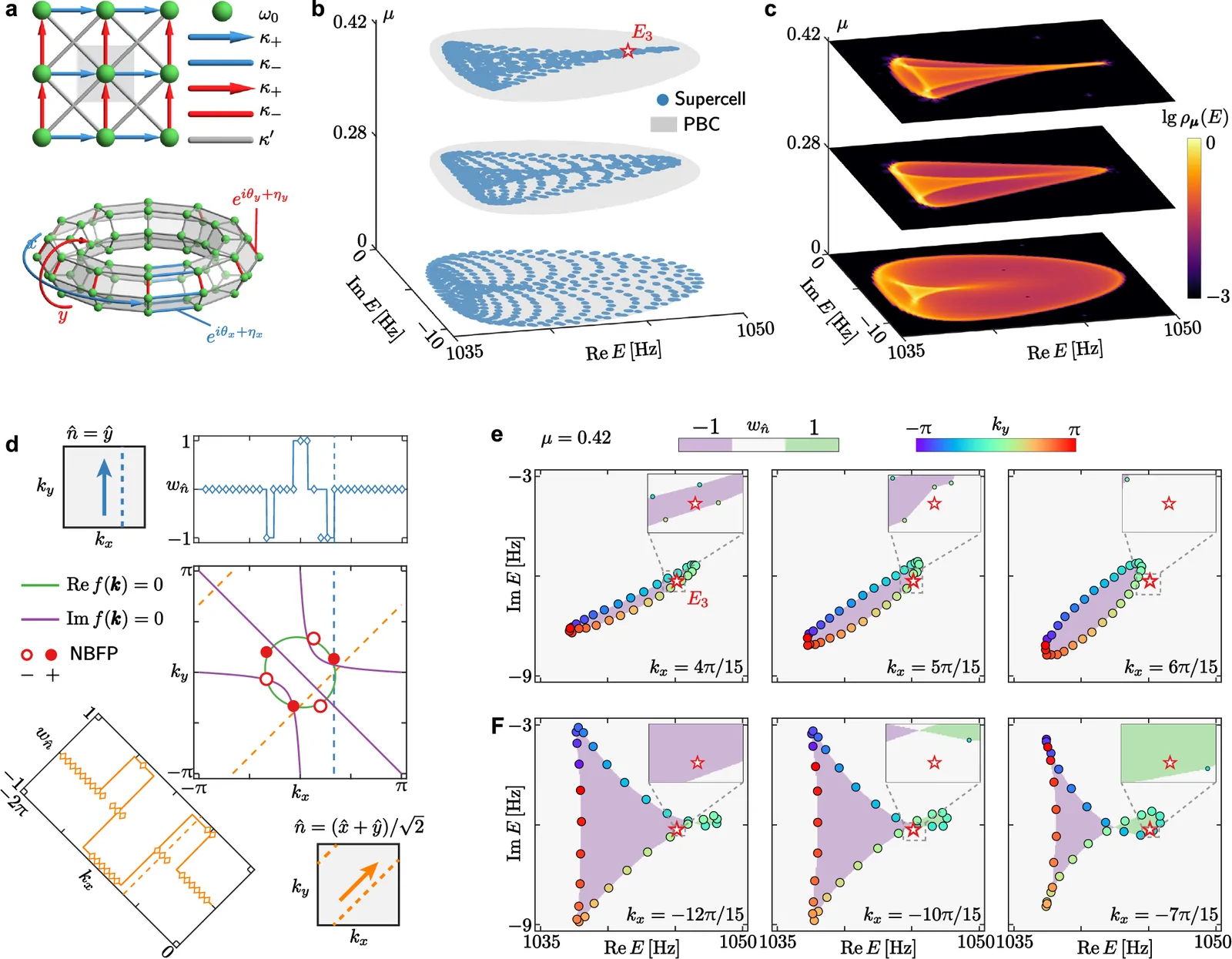
2D non-Hermitian band structures with projection-dependent diagnostics induced by NBFPs. **a** Schematic of a 2D nonreciprocal lattice (top) and its non-Bloch supercell (bottom). The used Hamiltonian is ${H}_{2{{\rm{D}}}}\left({{\boldsymbol{\beta }}}\right)={\omega }_{0}+{\kappa }_{+}\left({\beta }_{x}^{-1}+{\beta }_{y}^{-1}\right)+{\kappa }_{-}\left({\beta }_{x}+{\beta }_{y}\right)+\kappa {\prime} ({\beta }_{x}{\beta }_{y}+{\beta }_{x}^{-1}{\beta }_{y}+{\beta }_{x}{\beta }_{y}^{-1}+{\beta }_{x}^{-1}{\beta }_{y}^{-1})$. **b** Measured complex-energy spectra (blue dots) for ${\mu }_{x}={\mu }_{y}=\mu$. The gray region indicates the experimentally determined PBC spectral range. **c** Calculated density of NBFPs, ${\rho }_{{{\boldsymbol{\mu }}}}\left(E\right)$, for the corresponding μ in **b**. **d** Measured NBFP distribution for μ=0.42 and ${E}_{3}=(1045.1-6{\mbox{i}}){\mbox{Hz}}$ (red star in **b**). Six red markers denote NBFPs extracted from the experiment in **b** with open (filled) indicating their sign. Green (purple) lines show the theoretical loci ${\mathrm{Re}}f\left({{\boldsymbol{k}}}\right)=0$ [${{\rm{Im}}}f\left({{\boldsymbol{k}}}\right)$]; their intersections are the theoretical NBFPs. The top and bottom panels display two projection directions $\hat{n}$ and the ensuing eigenenergy winding number distributions ${w}_{\hat{n}}\left({k}_{x}\right)$, where $\hat{n}$ specifies the direction along which the winding number integral is performed. For example, in the top panel, the eigenenergy winding number ${w}_{\hat{y}}\left({k}_{x}\right)$ is computed by integrating over ${k}_{y}$ and has distributions in ${k}_{x}$. Measured point-gap patterns ${w}_{\hat{n}}\left({E}_{3},\mu=0.42;{k}_{x}\right)$ for $\hat{n}=\hat{y}$ (**e**) and $\hat{n}=\left(\hat{x}+\hat{y}\right)/\sqrt{2}$ (**F**). The three columns show patterns as ${k}_{x}$ crosses one NBFP (**e**) or two NBFPs (**F**). Marker color encodes ${k}_{\hat{n}}$ ($={{\boldsymbol{k}}}{{\cdot }}\hat{n}$), and region shading encodes ${w}_{\hat{n}}\left({E}_{3},\mu ;{k}_{x}\right)$. The evolution in **e** displays a typical topological transition of point gaps, while the evolution in **F** corresponds to the self-intersection scenario, which behaves like Fig. 3b. The parameters used are ${\omega }_{0}=\left(1040-6i\right){\mbox{Hz}}$, ${\kappa }^{{\prime} }=0.64{{\rm{Hz}}}$, ${\kappa }_{+}=2.72{{\rm{Hz}}}$, ${\kappa }_{-}=0.48{{\rm{Hz}}}$, ${N}_{x}=12$, and ${N}_{y}=6$.

We next apply the supercell protocol to higher dimensions using a 2D lattice (Fig. 5a). It is symmetric under x↔y exchange, so we first investigate $\mu={\mu }_{x}={\mu }_{y}$ to simplify interpretations. Figure 5b displays the measured ${E}_{v}^{{{\boldsymbol{\mu }}}}$ from the non-Bloch supercell for three typical μ values and for all experimentally sampled ${{\boldsymbol{k}}}$ points. Compared with Fig. 3b, the most striking difference is that ${E}_{v}^{{{\boldsymbol{\mu }}}}$ occupies a spectral area instead of a spectral line. It is a universal spectral feature for higher-dimensional non-Hermitian systems. A salient characteristic is the nonuniform distribution of ${E}_{v}^{{{\boldsymbol{\mu }}}}$ in the complex plane, which reflects the number of NBFPs mapping to the neighborhood of each E. Hence, for a given ${{\boldsymbol{\mu }}}$, we define the NBFP density ${\rho }_{{{\boldsymbol{\mu }}}}\left(E\right)={\sum }_{v}{{\rm{\delta }}}\left(E-{E}_{v}^{{{\boldsymbol{\mu }}}}\right)/{N}_{E}$, which relates to the density of states for arbitrary OBC shapes via spectral moments (Supplementary Note 6). It can be computed as ${\rho }_{{{\boldsymbol{\mu }}}}\left(E\right)={\nabla }_{E}^{2}\phi \left(E,{{\boldsymbol{\mu }}}\right)/2\pi$, where ${\nabla }_{E}^{2}$ are in the complex E plane and $\phi \left(E,{{\boldsymbol{\mu }}}\right)$ is the spectral potential in Eq. (4). The experimental $\phi \left(E,{{\boldsymbol{\mu }}}\right)$ is obtained by using Fig. 5b, showing good agreement with theoretical results (Supplementary Note 1). Hence, Fig. 5c presents the theoretical NBFP density corresponding to Fig. 5b, which clearly captures the experimental NBFP density and thereby confirms this higher-dimensional spectral feature.

Beyond density, NBFPs also have distinct locations in the BZ. We examine the experimental NBFP distribution at ${E}_{3}$ (marked by a star in Fig. 5b) and present it as circles in Fig. 5d. Because multiple NBFPs imply non-Bloch standing waves and are tied to point gaps, the eigenenergy winding numbers in Eq. (3) are projection-dependent. The choice of $\hat{n}$ sets the direction along which we diagnose point gaps and non-Bloch standing waves. Figure 5d compares two projections, $\hat{n}=\hat{y}$ (top) and $\hat{n}=\left(\hat{x}+\hat{y}\right)/\sqrt{2}$ (bottom), together with their corresponding ${w}_{\hat{n}}\left({{{\boldsymbol{k}}}}_{{{\perp }}}={k}_{x}\right)$. In both cases, we use ${k}_{x}$ to parametrize the sweeping loop (Fig. 5d, inset). For $\hat{n}=\hat{y}$, ${w}_{\hat{n}}\left({k}_{x}\right)$ jumps by 1 whenever a ${k}_{x}$-parametrized loop crosses an NBFP (Fig. 5d, top), meaning that a point gap closes or opens in the point-gap patterns (Fig. 5e and Supplementary Note 7). For $\hat{n}=\left(\hat{x}+\hat{y}\right)/\sqrt{2}$, ${w}_{\hat{n}}\left({k}_{x}\right)$ can jump by 1 or 2, depending on how many NBFPs are traversed by the ${k}_{x}$-parametrized loops (Fig. 5d, bottom). A unit jump is the same as in the $\hat{n}=\hat{y}$ case. A jump by 2 (e.g., −1→+1) occurs when the loop intersects two NBFPs simultaneously, corresponding to spectral self-intersections at ${E}_{3}$ (Fig. 5F and Supplementary Note 7). This motivates assigning a sign to each NBFP by tracing the phase gradient of the characteristic polynomial $f\left({{\boldsymbol{k}}}\right)=\,\det \left[{E}_{b}-{H}_{{{\boldsymbol{\mu }}}}\left({{\boldsymbol{k}}}\right)\right]$. By examining the phase gradient of $f\left({{\boldsymbol{k}}}\right)$ (Supplementary Note 1), we determine the sign of each NBFP, displayed by filled (+1, counterclockwise) and open (−1, clockwise) circles in Fig. 5d. The jump of ${w}_{\hat{n}}\left({k}_{x}\right)$ is then straightforward by examining the sum of signed NBFPs traversed by the ${k}_{x}$-parametrized loops. Experimentally, this capability not only predicts the potential formation of non-Bloch standing waves along the concerned direction but also offers a reliable knob for further anchoring the OBC spectral ranges.

**Fig. 6:**
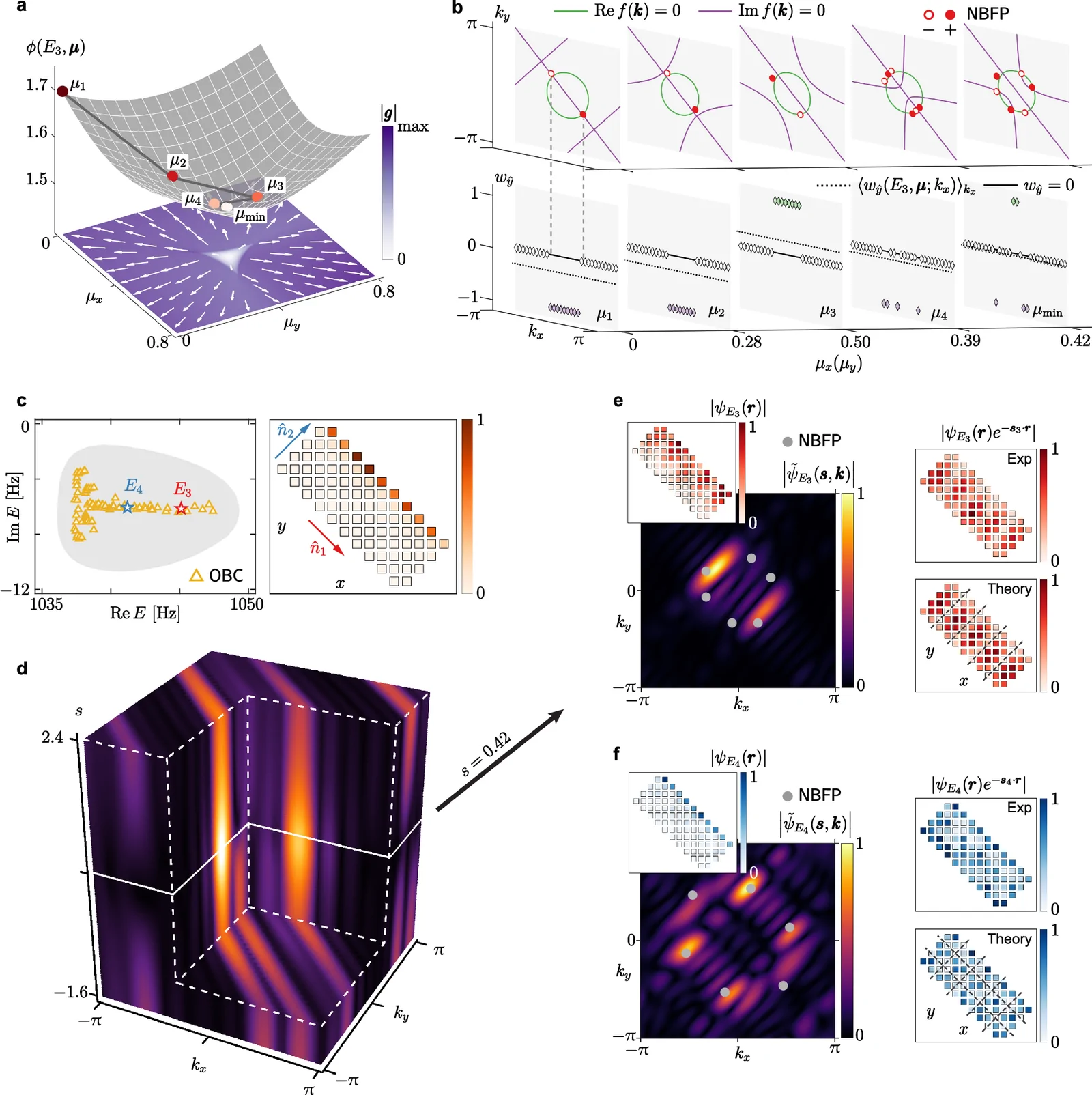
Experimental determination of spectral-potential minima, ensuing NBFPs, and OBC validation. **a** Spectral potential $\phi (E,{{\boldsymbol{\mu }}})$ for ${E}_{3}=(1045.1-6{\mbox{i}}){\mbox{Hz}}$ (red star in Fig.5b). The color map and arrows on the bottom plane show $\left|{{\boldsymbol{g}}}\right|$ and $-{{\boldsymbol{g}}}/\left|{{\boldsymbol{g}}}\right|$, respectively, where ${{\boldsymbol{g}}}={\nabla }_{{{\boldsymbol{\mu }}}}\phi (E,{{\boldsymbol{\mu }}})$ relates to eigenenergy winding numbers as ${\left\langle {w}_{\hat{n}}\left(E,{{\boldsymbol{\mu }}};{k}_{x}\right)\right\rangle }_{{k}_{x}}={{\boldsymbol{g}}}{{\cdot }}\hat{n}$. The ${{\boldsymbol{\mu }}}$ value at which $\left|{{\boldsymbol{g}}}\right|$ vanishes, ${{{\boldsymbol{\mu }}}}_{\min }$, is marked by white spheres and coincides with the minimum of $\phi ({E}_{3},{{\boldsymbol{\mu }}})$. **b** Evolution of measured NBFPs (open and filled circles, top) and their eigenenergy winding numbers ${w}_{\hat{y}}\left({E}_{3},{{\boldsymbol{\mu }}};{k}_{x}\right)$ (diamonds, bottom) for the ${{\boldsymbol{\mu }}}$ values indicated in **a**. The ${{\boldsymbol{\mu }}}$ positions follow a BFGS minimizer to approach ${{{\boldsymbol{\mu }}}}_{\min }$. Green (purple) curves in the top row are the theoretical loci ${\mathrm{Re}}f\left({{\boldsymbol{k}}}\right)=0$ $[{{\rm{Im}}}f\left({{\boldsymbol{k}}}\right)=0]$. In the bottom row, the solid (dashed) horizontal line denotes ${w}_{\hat{y}}=0$ [${\left\langle {w}_{\hat{y}}\left({E}_{3},{{\boldsymbol{\mu }}};{k}_{x}\right)\right\rangle }_{{k}_{x}}$]. **c** OBC spectra (top) and the spectral sum of right-eigenstate amplitudes (bottom) measured on a finite OBC lattice with 80 sites. Red and blue stars mark two selected energies, ${E}_{3}=(1045.1-6{\mbox{i}}){\mbox{Hz}}$ and ${E}_{4}=(1041.2-6{\mbox{i}}){\mbox{Hz}}$. Their ${{{\boldsymbol{\mu }}}}_{\min }$ values, determined via non-Bloch supercells, are 0.42 and 0.47, respectively. **d** *s*-resolved momentum-space wavefunction $\left|{\widetilde{\psi }}_{E}({{\boldsymbol{s}}},{{\boldsymbol{k}}})\right|$ for $E={E}_{3}$ as a function of ${{\boldsymbol{k}}}$ and ${s}_{x}={s}_{y}=s$. White solid lines mark the s=0.42 slice hosting the hotspot of $\left|{\widetilde{\psi }}_{{E}_{3}}\left({{\boldsymbol{s}}},{{\boldsymbol{k}}}\right)\right|$. *s*-resolved momentum-space wavefunction $\left|{\widetilde{\psi }}_{E}({{\boldsymbol{s}}},{{\boldsymbol{k}}})\right|$ (left panels) for $E={E}_{3}$ (**e**) and $E={E}_{4}$ (**f**), evaluated at the hotspot of $\left|{\widetilde{\psi }}_{E}\left({{\boldsymbol{s}}},{{\boldsymbol{k}}}\right)\right|$ for each energy. Gray circles mark NBFP positions measured by non-Bloch supercells, and the insets display the corresponding real-space right eigenstates measured by OBC lattices. The right panels display compensated wavefunctions $\left|{e}^{-{{\boldsymbol{s}}}\cdot {{\boldsymbol{r}}}}{\psi }_{E}({{\boldsymbol{r}}})\right|$ for $E={E}_{3}$ (**d**) and $E={E}_{4}$ (**e**) with corresponding $s={\mu }_{\min }=0.42$ and 0.47. The top and bottom panels show experimental and theoretical results, respectively. Dashed lines are plotted to indicate the interference nodes of $\left|{e}^{-{{\bf{s}}}\cdot {{\bf{r}}}}{\psi }_{E}({{\boldsymbol{r}}})\right|$.

Since ${w}_{\hat{n}}\left({{{\boldsymbol{k}}}}_{{{\perp }}}\right)$ is experimentally accessible, we again employ the spectral potential to investigate point gaps. Figure 6a shows the theoretical $\phi \left({E}_{3},{{\boldsymbol{\mu }}}\right)$ (surface) and its μ-gradient ${{\boldsymbol{g}}}$ (arrows), indicating the absence of a plateau. Following the previous discussion, ${E}_{3}$ belongs to the OBC spectra because $\left|{{\boldsymbol{g}}}\right|\ne 0$ near the minimizer ${{{\boldsymbol{\mu }}}}_{\min }$ of $\phi \left({E}_{3},{{\boldsymbol{\mu }}}\right)$. These ${{{\boldsymbol{\mu }}}}_{\min }$ play the same role as in 1D systems, and constitute the 2D generalized Brillouin zone together with the ${{\boldsymbol{k}}}$ values corresponding to this $\left(E,{{{\boldsymbol{\mu }}}}_{\min }\right)$. Exploiting the convexity of $\phi \left({E}_{3},{{\boldsymbol{\mu }}}\right)$ and the relation between ${{\boldsymbol{g}}}$ and ${\left\langle {w}_{\hat{n}}\left({{{\boldsymbol{k}}}}_{{{\perp }}}\right)\right\rangle }_{{{{\boldsymbol{k}}}}_{{{\perp }}}}$, we start from the PBC scenario (${{\boldsymbol{\mu }}}=0$) and follow the trajectory determined by a quasi-Newton search method (Fig. 6a, spheres) to locate ${{{\boldsymbol{\mu }}}}_{\min }$ experimentally (Methods). Figure 6b displays typical NBFPs (top, circles) and ${w}_{\hat{y}}\left({k}_{x}\right)$ (bottom, diamonds) for five ${{\boldsymbol{\mu }}}$. To pin down ${{{\boldsymbol{\mu }}}}_{\min }$, we compute ${\left\langle {w}_{\hat{y}}\left({k}_{x}\right)\right\rangle }_{{k}_{x}}$, shown as dashed lines (Fig. 6b, bottom), and minimize its distance to zero. Clearly, ${\left\langle {w}_{\hat{y}}\left({k}_{x}\right)\right\rangle }_{{k}_{x}}$ approaches zero at ${{{\boldsymbol{\mu }}}}_{\min }=\left(0.42,0.42\right)$ and remains nonzero nearby, consistent with the point-gap criterion.

Having predicted OBC behaviors from non-Bloch supercells, we next deploy a finite 2D lattice. One uniqueness in high dimensions is the system geometry, which strongly affects both OBC spectra and eigenstates. The measured NBFPs in Fig. 6b forecast non-Bloch standing waves along the ${\hat{n}}_{1}=\left(\hat{x}-\hat{y}\right)/\sqrt{2}$ and ${\hat{n}}_{2}=\left(\hat{x}+\hat{y}\right)/\sqrt{2}$ directions, which are intrinsically guaranteed by symmetry. We therefore fabricate a parallelogram lattice with edges aligned with these directions. Figure 6c shows the measured OBC spectra (top) and the spatial intensity summed over all right eigenstates (bottom), clearly revealing the wave localization on one edge, a signature of the NHSE.

We first analyze ${E}_{3}=(1045.1-6{\mbox{i}}){\mbox{Hz}}$, and its OBC eigenstate $\left|{\psi }_{{E}_{3}}\right|$ is shown in the inset of Fig. 6e. Performing the 2D s-resolved Fourier-Laplace transform in Eq. (5), we obtain the amplitude $\left|{\widetilde{\psi }}_{{E}_{3}}(s={s}_{x}={s}_{y},{{\boldsymbol{k}}})\right|$ (Fig. 6d), which exhibits two pronounced hot spots on the slice s=0.42, in excellent agreement with the non-Bloch supercell prediction. The amplitude $\left|{\widetilde{\psi }}_{{E}_{3}}(s=0.42,{{\boldsymbol{k}}})\right|$ (Fig. 6e) shows that two of the six NBFPs are strongly excited, indicating that the open boundary selectively couples to particular standing-wave pairs. We repeat the analysis for ${\psi }_{{E}_{4}}$ (Fig. 6f, inset) at ${E}_{4}=(1041.2-6{\mbox{i}}){\mbox{Hz}}$ and exhibit $\left|{\widetilde{\psi }}_{{E}_{4}}({s}_{x}={s}_{y}=0.47,{{\boldsymbol{k}}})\right|$ in Fig. 6f. In contrast to ${\psi }_{{E}_{3}}$, four of the six NBFPs are prominently excited, reflecting a distinct standing-wave composition. These measurements demonstrate that, in two dimensions, OBCs selectively excite specific NBFP combinations depending on energy and lattice symmetry, and reveal that the NBFP locations in the BZ are as important as $\left(E,{{{\boldsymbol{\mu }}}}_{\min }\right)$.

The NBFP localizations closely relate to the underlying symmetry, so for a given boundary geometry in Fig. 6c, the selection of NBFP combinations can be understood as the formation of standing waves through the interference of different NBFP pairs that satisfy the boundary constraints. To reveal, we compensate for the exponential decay by applying ${e}^{-{{\boldsymbol{s}}}\cdot {{\boldsymbol{r}}}}{\psi }_{E}({{\boldsymbol{r}}})$ with ${{\boldsymbol{s}}}={{{\boldsymbol{\mu }}}}_{\min }$, as shown in the right panels of Fig. 6e–f, where the upper and lower panels correspond to the experimental and theoretical results, respectively. For ${E}_{3}$ (Fig. 6e), the compensated wavefunction exhibits four nodes along ${\hat{n}}_{1}$ and none along ${\hat{n}}_{2}$, whereas for ${E}_{4}$(Fig. 6f), it exhibits four nodes along ${\hat{n}}_{1}$ and two along ${\hat{n}}_{2}$, respectively. These interference nodes can be inferred by the momentum separations between the corresponding NBFP pairs together with the standing-wave conditions (see Supplementary Note 8 for details).

To date, the conceptual role of NBFPs in non-Hermitian band theory has become transparent, especially when going beyond 1D scenarios. The NBFP density determines the spectral structure of fixed-decay-length wavefunctions and relates to OBC behaviors via a conservative quantity, namely, the spectral moment^47^. The NBFP locations specify interference directions of non-Bloch waves supported by particular geometries. Detecting NBFPs from OBC measurements by using an s-resolved Fourier-Laplace transform offers a practical approach to determining localization behaviors of skin modes (Supplementary Note 9). Therefore, NBFPs are not only indicators of non-Bloch spectral topology but also observable quantities that can be directly probed and independently verified in OBC experiments.

### Non-Bloch band reconstruction beyond the skin effect

All the examples discussed above are associated with point-gap topology and exhibit the NHSE under OBCs. Since the essence of the non-Bloch supercell framework lies in the equivalence between complex-continuation TBCs and the non-Bloch description, its applicability is not restricted to systems with skin effects. More generally, it provides a versatile experimental platform for characterizing quantities defined on non-Hermitian eigenenergies and eigenstates, including band dispersions, Berry curvature, and related spectral and topological properties.

**Fig. 7:**
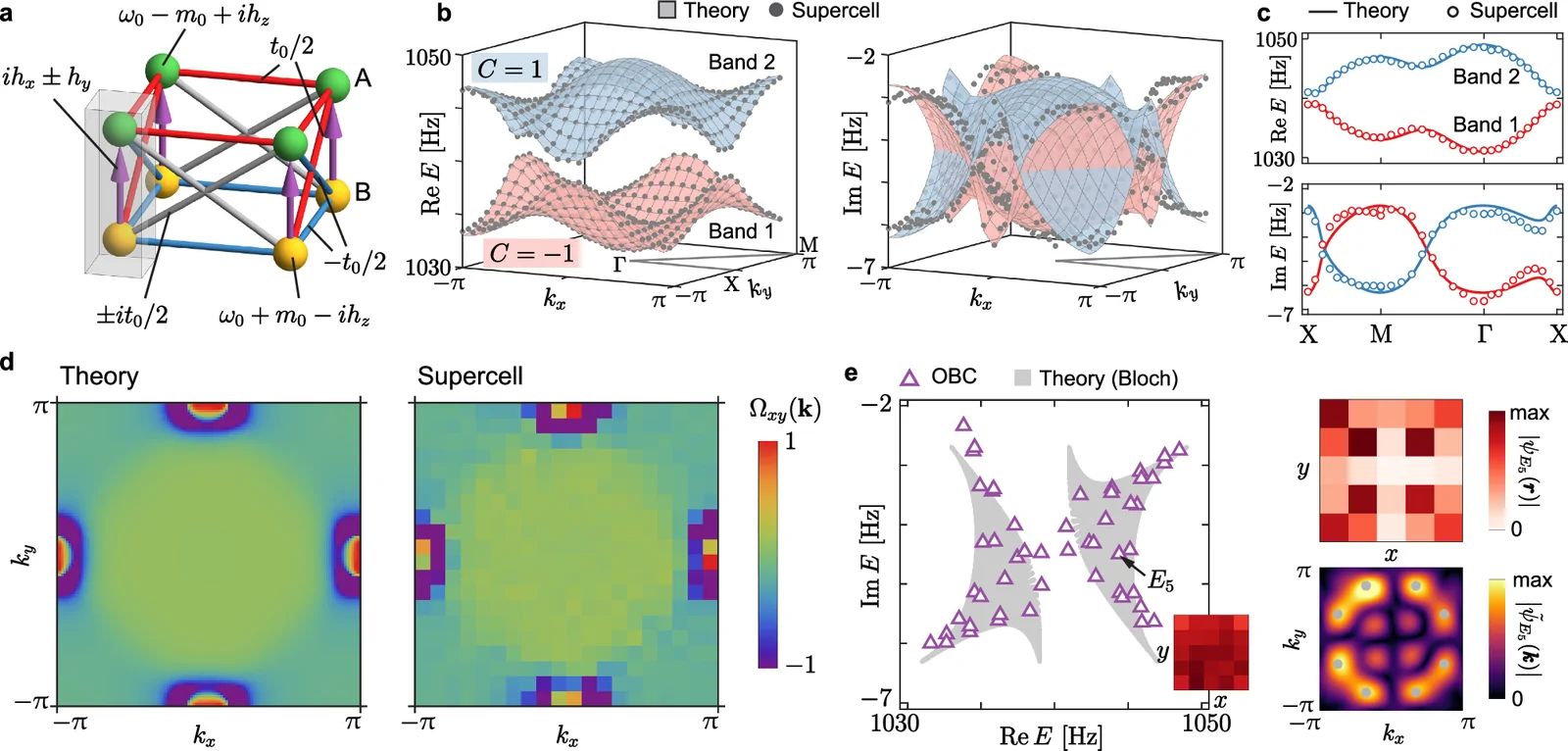
Generalizations of the non-Bloch supercell framework: band structures and Berry curvature. **a** Schematic of a 2D non-Hermitian Chern insulator. The used Hamiltonian is ${H}_{{{\rm{CI}}}}({{\boldsymbol{\beta }}})={\omega }_{0}{\sigma }_{0}+{{\boldsymbol{\sigma }}}\cdot [{{\boldsymbol{d}}}({{\boldsymbol{\beta }}})+i{{\boldsymbol{h}}}]$, where ${{\boldsymbol{d}}}\left({{\boldsymbol{\beta }}}\right)=[{-{it}}_{0}({\beta }_{x}-{\beta }_{x}^{-1}),\,{-{it}}_{0}({\beta }_{y}-{\beta }_{y}^{-1}),{t}_{0}({\beta }_{x}+{\beta }_{x}^{-1}+{\beta }_{y}+{\beta }_{y}^{-1})-{2m}_{0}]/2$, ${\sigma }_{0}$ is the identity matrix, ${{\boldsymbol{\sigma }}}=({\sigma }_{x},{\sigma }_{y},{\sigma }_{z})$ denotes the Pauli matrices, and ${{\boldsymbol{h}}}=({h}_{x},{h}_{y},{h}_{z})\in {{\mathbb{R}}}^{3}$. **b** Measured non-Hermitian band structures ${\mathrm{Re}}\,{E}^{{{\boldsymbol{\mu }}}}({{\boldsymbol{\beta }}})$ (left) and ${{\rm{Im}}}\,{E}^{{{\boldsymbol{\mu }}}}({{\boldsymbol{\beta }}})$ (right) at ${{{\boldsymbol{\mu }}}}_{\min }=({\mathrm{0,0}})$. The surfaces represent theoretical predictions, while the filled spheres denote experimental measurements. **c** Band dispersions along the high-symmetry lines indicated in **b**, showing excellent agreement between theory (lines) and experiment (circles). **d** Berry curvature distributions reconstructed from experimentally measured left and right eigenvectors (right), compared with theoretical calculations (left). **e** OBC measurements on a finite lattice containing 50 sites. Left: OBC spectra (triangles). The gray background denotes the theoretical PBC spectra. The inset shows the spectral sum of bulk states, confirming the absence of NHSE. Right: measured real-space wavefunction of the A-sublattice at the selected energy ${E}_{5}$ (top) and the corresponding Fourier transforms (bottom). Gray circles mark NBFPs obtained from non-Bloch supercells. The parameters used are ${\omega }_{0}=\left(1040-4.5i\right){{\rm{Hz}}}$, ${m}_{0}=-1\,{{\rm{Hz}}}$, ${t}_{0}=4\,{{\rm{Hz}}}$, ${h}_{x}={h}_{y}=0\,{{\rm{Hz}}}$, and ${h}_{z}=1.8{{\rm{Hz}}}$. The supercell is composed of ${N}_{x}={N}_{y}=1$ unit cell, corresponding to the single-cell limit of our non-Bloch supercell framework.

To explicitly demonstrate the generality of our framework, we have deployed a non-Hermitian Chern insulator (Fig. 7a) that respects spectral reciprocity (Fig. 7b, surfaces). The model possesses inversion symmetry ${PH}({{\boldsymbol{k}}}){P}^{-1}=H(-{{\boldsymbol{k}}})$, with $P={\sigma }_{z}$, which guarantees ${{{\boldsymbol{\mu }}}}_{\min }=({\mathrm{0,0}})$. In such systems, the generalized Brillouin zone coincides with the ordinary BZ, and no spectral-potential minimization is required to determine ${{{\boldsymbol{\mu }}}}_{\min }$. Consequently, the system does not support the nonreciprocal skin effect, and exponential accumulation is absent, implying that no exponent flattening is required. We then directly use a primitive-cell configuration under TBCs by sweeping $({k}_{x},\,{k}_{y})$. The experimentally measured complex band structures, shown as filled spheres in Fig. 7b and open circles in Fig. 7c, exhibit excellent agreement with the theoretical predictions. A ${{\rm{Re}}}E$ line gap is clearly observed, thereby enabling the experimental characterization of the associated Chern topology and topological boundary modes.

Because both left and right eigenstates are experimentally accessible within our framework, we now directly reconstruct the Berry curvature from the measured eigenstates. At each ${{\boldsymbol{k}}}=({k}_{x},{k}_{y})$, the measured eigenstates form the biorthogonal set $\left\{{u}_{n}^{{\mbox{R}}}({{\boldsymbol{k}}}),{u}_{n}^{{\mbox{L}}}({{\boldsymbol{k}}})\right\}$, where n is the band index. We then compute Berry curvature distributions throughout the BZ using the discretized biorthogonal Wilson-loop formalism (Methods). Figure 7d exhibits the experimentally reconstructed Berry curvature distribution (right) and the corresponding theoretical results (left) for the upper band. Two pronounced hot spots are observed near the band edges. Integrating the Berry curvature over the BZ yields the Chern number of each band, as indicated in Fig. 7b.

According to the bulk-boundary correspondence, we then experimentally investigate the OBC behavior. Figure 7e shows the measured OBC spectra (left), where topological boundary modes emerge within the ${{\rm{Re}}}E$ line gap. The inset shows the spectral sum of bulk states, confirming the absence of skin accumulation. Finally, we select the OBC eigenstate at the target energy ${E}_{5}$ (Fig. 7e, top-right) and perform Fourier transforms of its A-sublattice wavefunction component. The resulting momentum-space hot spots agree remarkably well with the NBFPs predicted by the supercell framework, as shown in the bottom-right panel of Fig. 7e.

These results demonstrate explicitly that systems without skin accumulation correspond to the special case ${{{\boldsymbol{\mu }}}}_{\min }=0$ within our framework, rather than constituting an exception to it. More generally, this example shows that the non-Bloch supercell framework naturally extends beyond skin-effect diagnostics to multiband non-Hermitian systems with nontrivial topology, enabling the experimental reconstruction of biorthogonal quantum geometry and topological invariants.

## Discussion

In summary, we have introduced and experimentally demonstrated a scalable protocol for mapping non-Hermitian band structures. By treating Bloch phase and the imaginary part of crystal momentum as independent knobs—implemented via non-Bloch supercells with exponent flattening—and by extracting complex eigenenergies and biorthogonal eigenstates through the Green’s function measurements, we have achieved high-resolution, momentum-resolved mapping of complex-energy surfaces, along with their diagnostics, including NBFPs, point-gap patterns, and spectral potentials, and have reconstructed Berry curvature distributions. Implemented in programmable 1D and 2D acoustic crystals, the non-Bloch supercell scheme experimentally predicts OBC spectra and mode compositions, providing a compact toolkit for characterizing the geometric, topological, and spectral content of non-Hermitian bands.

A key physical insight offered by our non-Bloch supercell framework is that k-μ decoupling converts imaginary momentum from an implicit analytic continuation into an experimentally controllable and physically interpretable variable. Probing non-Hermitian band structures can therefore be reformulated in terms of NBFPs. In this picture, NBFPs acquire a concrete physical role: they link the spectral deformation induced by ${{\boldsymbol{\mu }}}$ to the corresponding spatial amplification or attenuation of eigenstates. In particular, applying an s-resolved Fourier-Laplace transform to OBC eigenstates to extract NBFPs further demonstrates the necessity of imaginary-momentum compensation for recovering information lost due to non-Hermitian attenuation, thereby providing a general strategy for recovering boundary-sensitive information in real-space non-Hermitian systems with open boundaries. This connection remains conceptually useful even in systems where hopping amplitudes cannot be directly programmed, as it provides a way to classify the relationship between spectral features and eigenstate decay behaviors within a unified non-Bloch description (Supplementary Note 9).

Beyond the specific platform demonstrated here, our non-Bloch supercell framework provides a transferable experimental protocol for reconstructing non-Bloch band structures, provided that the hopping amplitudes can be programmed and the full Green’s function response can be measured^30,48–51^. Since non-Bloch band theory is primarily intended to explain non-Hermitian phenomena under OBCs, any experimental reconstruction of non-Bloch band structures should be benchmarked against the corresponding OBC spectra and eigenstates. Our framework enables such a benchmark by connecting TBC-based non-Bloch band reconstruction with direct OBC spectral and eigenstate measurements, which is particularly useful because realizing genuine OBCs in synthetic dimensions is often experimentally challenging^52–55^. For platforms that naturally support OBCs, OBC measurements and probing non-Bloch band structures are equally vital, as neither alone provides all the information about non-Hermitian systems. Bridging these concepts, both conceptually and technically, is what our framework provides. The conceptual advance lies in the k-μ decoupling and NBFPs through TBCs, while the technical advance over the single-cell limit is an N-fold improvement in ${{\boldsymbol{k}}}$ space resolution without sacrificing the accessible dynamic range of ${{\boldsymbol{\mu }}}$. Compared with prior experimental work on probing non-Bloch bands in synthetic dimensions^48,52,53^ and OBC measurements^18,25,56,57^, our non-Bloch supercell framework enables the simultaneous investigation of non-Bloch band structures alongside OBC spectral and eigenstates across dimensions.

Looking forward, the non-Bloch supercell framework can serve as a modular probe for more general non-Hermitian systems incorporating additional physical ingredients, such as temporal modulations^58,59^. By reconstructing non-Hermitian band structures while retaining predictive power for open-geometry responses, it provides a controllable route to characterize the spatial non-Bloch sector of such systems^60^. Combined with stroboscopic, Floquet, or dynamical measurements, this approach may enable experimental access to broader non-Hermitian phenomena, including Floquet topology and dynamical non-Bloch effects^42,51,58^.

## Methods

### Finite dynamic range

Dynamic range refers to the interval between the largest and smallest signals that a measurement system can reliably resolve; the upper limit is typically set by saturation, compression, or distortion, while the lower limit is determined by the noise floor.

In our case, finite dynamic range means that the experimentally controllable parameter—here, the hopping amplitude—can only be varied within a finite interval. Beyond this range, the signal would either be buried in noise or enter a nonlinear or saturated regime, rendering it unreliable for resolution. The inter-supercell hopping along direction m is modulated as ${t}_{{ij}}\to {t}_{{ij}}{e}^{{\eta }_{m}}{e}^{i{\theta }_{m}}$, so the effective hopping magnitude becomes $\left|{t}_{{ij}}\right|{e}^{{\eta }_{m}}$. If the experimentally realizable hopping strengths are constrained to a finite interval $[{{{\mathcal{K}}}}_{\min },\,{{{\mathcal{K}}}}_{\max }]$ then the accessible range of ${\eta }_{m}$ is

6 $${\eta }_{m}\in \left[{{\mathrm{ln}}}\left(\frac{{{{\mathcal{K}}}}_{\min }}{\left|{t}_{{ij}}\right|}\right),\,{{\mathrm{ln}}}\left(\frac{{{{\mathcal{K}}}}_{\max }}{\left|{t}_{{ij}}\right|}\right)\right].$$

Since ${\eta }_{m}={N}_{m}{\mu }_{m}$, where ${\mu }_{m}$ is the imaginary part of the primitive-cell momenta along the direction m, the corresponding range of ${\mu }_{m}$ scales as

7 $${\mu }_{m}\in \frac{1}{{N}_{m}}\left[{{\mathrm{ln}}}\left(\frac{{{{\mathcal{K}}}}_{\min }}{\left|{t}_{{ij}}\right|}\right),\,{{\mathrm{ln}}}\left(\frac{{{{\mathcal{K}}}}_{\max }}{\left|{t}_{{ij}}\right|}\right)\right],$$

which explains why the accessible range $R({\mu }_{m})$ decreases as ${N}_{m}^{-1}$.

### Sample fabrication and experimental setup

The system comprises multiple acoustic cavities, each emulating a lattice site. These acoustic cavities are fabricated using three-dimensional printing with a tolerance of 0.2 mm or within 0.3%. The material is LEDO 6060 photosensitive resin, which behaves acoustically as a rigid material for airborne sound. Each printed cavity has a wall thickness of 6 mm. As shown in Supplementary Note 1, the cavities are hexagonal prisms with an interior height of 164 mm and a side length of 25 mm. To characterize the cavity in the experiment, a loudspeaker (the source) excites the cavity, and a microphone (the detector) measures the acoustic pressure.

A custom-designed digital controller is employed to precisely tune the onsite potentials of acoustic cavities and hoppings between them. The tuning of onsite potentials and hoppings is implemented using active components, specifically microphone-loudspeaker (pump-probe) pairs. The hopping implementations are independent of spatial distances between sites, enabling flexible implementation of various boundary conditions and lattice geometries.

The custom-made digital controller comprises three components: the core board, the motherboard, and the input-output board. The core board houses a field-programmable gate array (FPGA, XC7K325T, Xilinx) and a digital signal processor (DSP, TMS320C6678, Texas Instruments), enabling real-time signal processing. During experiments, the system operates at a sampling frequency of 12.8 kHz. The motherboard supplies power and facilitates communication between the core and input-output boards. The input-output board handles analog signal acquisition and output, interfacing with microphones and loudspeakers, respectively. Each input-output board supports 16 channels for both analog inputs and outputs. The input channels utilize analog-to-digital converters (ADCs, ADC7606B, ADI), and the output channels employ digital-to-analog converters (DACs, DAC8568, Texas Instruments).

### Green’s-function-based measurement of eigenenergies and eigenstates

In the experiments, we measure the system-wide Green’s functions (two-point correlators) and subsequently retrieve the complex eigenenergies and eigenstates of non-Hermitian systems^17,25,30^. Specifically, for a lattice of N sites, we measure the steady-state response at every probe sites i for each pump site j under monochromatic excitation at frequency ω. This yields the full response matrix ${G}_{{ij}}\left(\omega \right)$ over a discrete set of driving frequencies spanning the band of interest. The amplitude and phase of the response are obtained by referencing the microphone signals to the excitation signal, allowing coherent extraction of ${G}_{{ij}}\left(\omega \right)$. The full matrix acquisition over all pump-probe pairs yields ${N}^{2}$ complex transfer functions per frequency point.

The Green’s function satisfies $G\left(\omega \right)={\left(\omega -H\right)}^{-1}$, so the eigenvectors of $G\left(\omega \right)$ coincide with those of H, and its eigenenergies follow the single-pole form ${{{\rm{\lambda }}}}_{{{\rm{\nu }}}}\left({{\rm{\omega }}}\right)=1/({{\rm{\omega }}}-{{{\rm{E}}}}_{{{\rm{\nu }}}})$ up to a complex prefactor set by the measurement normalization. Concerning the effective tight-binding description here, we diagonalize $G\left(\omega \right)$ for each sampled ${{\rm{\omega }}}$, track each eigenenergie branch ${\lambda }_{\nu }\left(\omega \right)$ across frequency, and fit to ${\lambda }_{\nu }\left(\omega \right)=1/(\omega -{E}_{\nu })$ to extract the system eigenenergy ${E}_{\nu }$. This procedure yields both ${\mbox{Re}}\,{E}_{\nu }$ and ${\mbox{Im}}\,{E}_{\nu }$, allowing direct access to the complex spectrum. The biorthogonal eigenstates corresponding to ${E}_{\nu }$ are simultaneously obtained from the eigenvectors of $G\left(\omega \right)$ evaluated along the branch ${\lambda }_{\nu }\left(\omega \right)$.

### Robustness of Green’s-function-based spectral reconstruction

In our acoustic platform, passive material loss and parasitic dissipation enter the effective Hamiltonian primarily as imaginary onsite terms. If the loss is spatially uniform, it can be written as a scalar contribution

8 $$H={H}_{{\mbox{id}}}-i{\alpha }_{0}I,$$

where ${H}_{{\mbox{id}}}$ is the ideal Hamiltonian, I is the identity matrix, and ${\alpha }_{0} > 0$ is the uniform background loss. This term shifts all eigenenergies by the same amount, ${E}_{n}\to {E}_{n}-i{\alpha }_{0}$, but leaves the right and left eigenstates unchanged. Equivalently, with the convention ${e}^{-i2\pi {Et}}$, the field acquires a common temporal decay factor ${e}^{-2\pi {\alpha }_{0}t}$. Therefore, a uniform loss in principle does not modify the extracted eigenstate profiles, complex momenta, generalized Brillouin zone, or point-gap winding structure, except for a global displacement of the spectrum along the imaginary-energy axis.

However, the more relevant source of experimental uncertainty is residual nonuniform loss, and the complex spectra and eigenstates are reconstructed from the experimentally measured full Green’s function. As a result, any residual loss remaining in the physical system can still affect the reconstruction accuracy indirectly through finite measurement precision. In particular, when the loss is large, the response away from the excitation site may become weak and approach the noise floor of the microphone measurement. In this situation, the measured Green’s function contains uncertainties, which can invalidate the extraction of complex poles and eigenstates. Therefore, the relevant practical limitation is not the existence of loss itself, but the signal-to-noise ratio and dynamic range with which the Green’s function is measured. In our experiments, the signal-to-noise ratio is more than 40 dB, ensuring a negligible impact on the measured results^30^.

### Experimental measurement of eigenenergy winding numbers

For each given ${{\boldsymbol{\mu }}}$, the experimental data $\{{E}_{v}^{{{\boldsymbol{\mu }}}},{\theta }_{x},\,{\theta }_{y}\}$ with $v=1,\ldots,{N}_{E}$ can give ${E}^{{{\boldsymbol{\mu }}}}({k}_{x},{k}_{y})$ by using the method detailed in Supplementary Note 1. If we take $\hat{n}=\hat{y}$, the eigenenergy winding number is then computed

9 $${w}_{\hat{y}}\left(E,{{\boldsymbol{\mu }}};{k}_{x}\right)=\frac{1}{2\pi }\mathop{\sum }\limits_{{v}_{y}}{{\rm{arg }}}\frac{{E}^{{{\boldsymbol{\mu }}}}\left({k}_{x},{k}_{{v}_{y}+1}\right)-E}{{E}^{{{\boldsymbol{\mu }}}}\left({k}_{x},{k}_{{v}_{y}}\right)-E},$$

where ${v}_{y}=1,\ldots,{N}_{{k}_{y}}$ indexes sampling points in BZ along the ${k}_{y}$ direction. Sweeping ${k}_{x}$ and averaging yields the directional derivative

10 $${\left\langle {w}_{\hat{y}}\left(E,{{\boldsymbol{\mu }}};{k}_{x}\right)\right\rangle }_{{k}_{x}}={\partial }_{{\mu }_{y}}\phi \left(E,{{\boldsymbol{\mu }}}\right)={g}_{y}.$$

Measuring averaged windings for a set of linearly independent directions $\hat{n}$ gives the full gradient ${{\boldsymbol{g}}}$.

### Locating the spectral-potential minimum

With spectral potential $\phi \left(E,{{\boldsymbol{\mu }}}\right)$ and its gradient ${{\boldsymbol{g}}}$ measured, we locate the minimizer ${{{\boldsymbol{\mu }}}}_{\min }$ using a BFGS (Broyden-Fletcher-Goldfarb-Shanno) quasi-Newton method. Starting from an initial guess ${{{\boldsymbol{\mu }}}}_{0}$ (e.g., ${{{\boldsymbol{\mu }}}}_{0}=0$ for the PBC case), it maintains an approximation ${H}_{q}$ to the inverse Hessian and performs iterations ${{{\boldsymbol{\mu }}}}_{q+1}={{{\boldsymbol{\mu }}}}_{q}-{H}_{q}{{{\boldsymbol{g}}}}_{q}$, where q indexes the iteration. We declare convergence when both ${||}{{{\boldsymbol{g}}}}_{q}{||}$ and the relative change in ϕ fall below a preset tolerance. Because $\phi \left(E,{{\boldsymbol{\mu }}}\right)$ is convex with respect to ${{\boldsymbol{\mu }}}$, any stationary point found by this procedure is the global minimizer.

### Experimental measurement of Berry curvature and Chern numbers

Following the gauge-invariant lattice formulation^61,62^, the link matrix along direction m=x,y is defined as

11 $$[{M}_{m}\left({{\boldsymbol{k}}}\right)]_{n^{\prime} n}=\left\langle {u}_{n^{\prime} }^{L}({{\boldsymbol{k}}})\bigg|{u}_{n}^{R}\left({{\boldsymbol{k}}}+\delta {{{\boldsymbol{k}}}}_{m}\right.\right\rangle,$$

where $\delta {{{\boldsymbol{k}}}}_{m}$ denotes the momentum increment between neighboring sampling points in the BZ along direction m, i.e., $\delta {{{\boldsymbol{k}}}}_{x}=({k}_{{v}_{x}+1}-{k}_{{v}_{x}},0)$ and $\delta {{{\boldsymbol{k}}}}_{y}=(0,{k}_{{v}_{y}+1}-{k}_{{v}_{y}})$. Using the measured biorthogonal left and right eigenstates, the discretized Berry curvature is computed as

12 $${\varOmega }_{{xy}}\left({{\boldsymbol{k}}}\right)=\frac{{{\rm{Im}}} \, {{\rm{In}}}\det \left[{M}_{x}({{\boldsymbol{k}}}){M}_{y}({{\boldsymbol{k}}}+{\delta {{\boldsymbol{k}}}}_{x}){M}_{x}^{-1}({{\boldsymbol{k}}}+{\delta {{\boldsymbol{k}}}}_{y}){M}_{y}^{-1}({{\boldsymbol{k}}})\right]}{{||}{\delta {{\boldsymbol{k}}}}_{x}{||||}{\delta {{\boldsymbol{k}}}}_{y}{||}}.$$

The Chern number is then obtained by summing the Berry curvature over the BZ

13 $$C=\frac{1}{2\pi }\mathop{\sum}\limits_{{{\boldsymbol{k}}}}{\varOmega }_{{xy}}\left({{\boldsymbol{k}}}\right)\left|\left|{\delta {{\boldsymbol{k}}}}_{x}\right|\right|\left|\left|{\delta {{\boldsymbol{k}}}}_{y}\right|\right|.$$

## Supplementary information

Supplementary Information

Transparent Peer Review file

## Data Availability

All the data supporting this study are available in the paper and Supplementary Information. Additional data related to this paper are available from the corresponding authors upon request.

## Code availability

All the data supporting this study are available in the paper and Supplementary Information. Additional data related to this paper are available from the corresponding authors upon request.
