# Supplementary material for: Unveiling non-Hermitian band structures with non-Bloch supercells
Source: Nat Commun. 2026 Aug 26;17:10184. doi: 10.1038/s41467-026-77152-5 (PMC13612471; doi:10.1038/s41467-026-77152-5)
Supplement: Supplementary file 1 [file 41467_2026_77152_MOESM1_ESM.pdf]

Supplemental Information for

Unveiling non-Hermitian band structures with non-Bloch supercells

Jia-Xin Zhong[*,1,3], Jing Lin[*,#,2], Kai Chen[3,4], Jing Lu[3,4,§], Kun Ding[2,‡], and Yun Jing[1,†]

[1] *Graduate Program in Acoustics, The Pennsylvania State University, University Park, PA 16802, USA*

[2] *Department of Physics, State Key Laboratory of Surface Physics, and Key Laboratory of Micro and Nano Photonic Structures (Ministry of Education), Fudan University, Shanghai 200438, China*

[3] *Key Laboratory of Modern Acoustics and Institute of Acoustics, Nanjing University, Nanjing 210093, China*

[4] *NJU-Horizon Intelligent Audio Lab, Horizon Robotics, Beijing 100094, China*

*These authors contributed equally.

#E-mail: jing_lin@fudan.edu.cn, §E-mail: lujing@nju.edu.cn,

‡E-mail: kunding@fudan.edu.cn, †E-mail: yqj5201@psu.edu

Contents

Supplementary Note 1. Post-processing of experimental data 2
Supplementary Note 2. More experimental details....... 5
Supplementary Note 3. Point-gap criterion for the open-boundary spectrum 10
Supplementary Note 4. Relation between spectral potential and averaged winding number along arbitrary directions 13
Supplementary Note 5. Equivalence of the supercell recipe to the auxiliary generalized Brillouin zone criterion in 1D systems 16
Supplementary Note 6. Density of non-Bloch Fermi points and spectral moments....... 17
Supplementary Note 7. Extended experimental results of point-gap patterns observed in 2D non-Hermitian lattices....... 20
Supplementary Note 8. NBFP-compatible standing waves 22
Supplementary Note 9. Using NBFPs to distinguish different classes of skin effects 27
References 32

Supplementary Note 1. Post-processing of experimental data

We illustrate the post-processing of experimental data measured with the setup described in the main text. In our implementation of the non-Bloch supercell method, the control of the imaginary momentum $\boldsymbol{\mu}$ is encoded in the intercell hoppings. For each fixed deformation parameter $\boldsymbol{\mu}$, sweeping the twisted boundary phases (θ_x, θ_y) yields a set of sampled points in the complex-energy plane. These raw points are further processed to obtain richer, physically meaningful information.

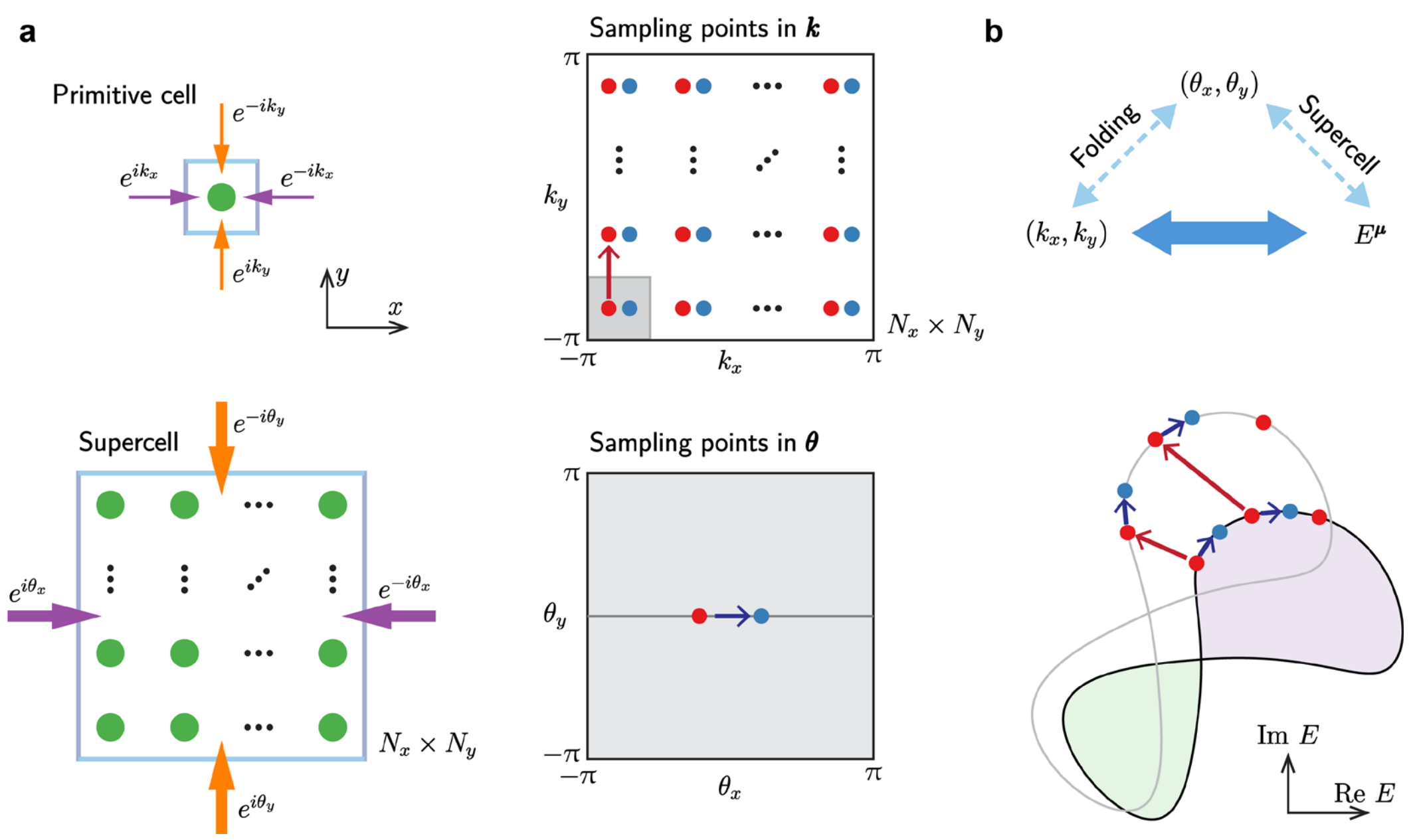

Supplementary Figure 1 | Constructing dispersion relations with supercells. a, Momentum sampling in primitive cells and supercells. The first column displays schematics of a primitive cell (top) and a supercell (bottom), while the second column displays the corresponding momentum sampling. The supercell consists of $N_x \times N_y$ primitive cells with TBCs (θ_x, θ_y). As an illustration, two representative momentum points (red and blue circles) in the (θ_x, θ_y) plane map to an $N_x \times N_y$ set of sampling points in the primitive-cell BZ (k_x, k_y). **b**, Relations between momentum and complex eigenenergy. For a single band, any fixed (θ_x, θ_y) yields $N_x N_y$ complex eigenenergies E. Starting from a given (θ_x, θ_y), with its mapping to (k_x, k_y) specified in **a**, we trace the evolution of the complex eigenenergies along closed loops in the (θ_x, θ_y) plane (blue arrows in **a** and **b**), thereby establishing a one-to-one correspondence between (k_x, k_y) and E.

First, we construct dispersion relations, i.e., associate the measured energies $\{E_v^{\boldsymbol{\mu}}\}_{v=1}^{N_E}$ with primitive-cell momenta $\boldsymbol{k} = (k_x, k_y)$. A supercell of size $N_x \times N_y$ under twisted boundary conditions (TBCs) yields $N_x \times N_y$ sampling points in the primitive-cell Brillouin zone (BZ)

(Fig. 1a). For a given (θ_x, θ_y) the corresponding primitive-cell momenta are $(k_x, k_y) = (\frac{\theta_x}{N_x} - \pi + \frac{2\pi v_x}{N_x}, \frac{\theta_y}{N_y} - \pi + \frac{2\pi v_y}{N_y})$, with $v_{x(y)} = 1, \ldots, N_{x(y)}$ (Fig. 1a, right). This band-folding relation is illustrated in Fig. 1a (right panels) and explains why the supercell improves k-space sampling density.

By sweeping a path in twist-angle space (θ_x, θ_y) (blue arrow in Fig. 1a, lower right), the measured eigenenergies trace trajectories (solid lines) in the complex-energy plane (Fig. 1b); following each trajectory provides a one-to-one pairing between measured energies $E_v^{\boldsymbol{\mu}}$ and primitive k points, which yields the discrete $E^{\boldsymbol{\mu}}(\boldsymbol{k})$ mapping.

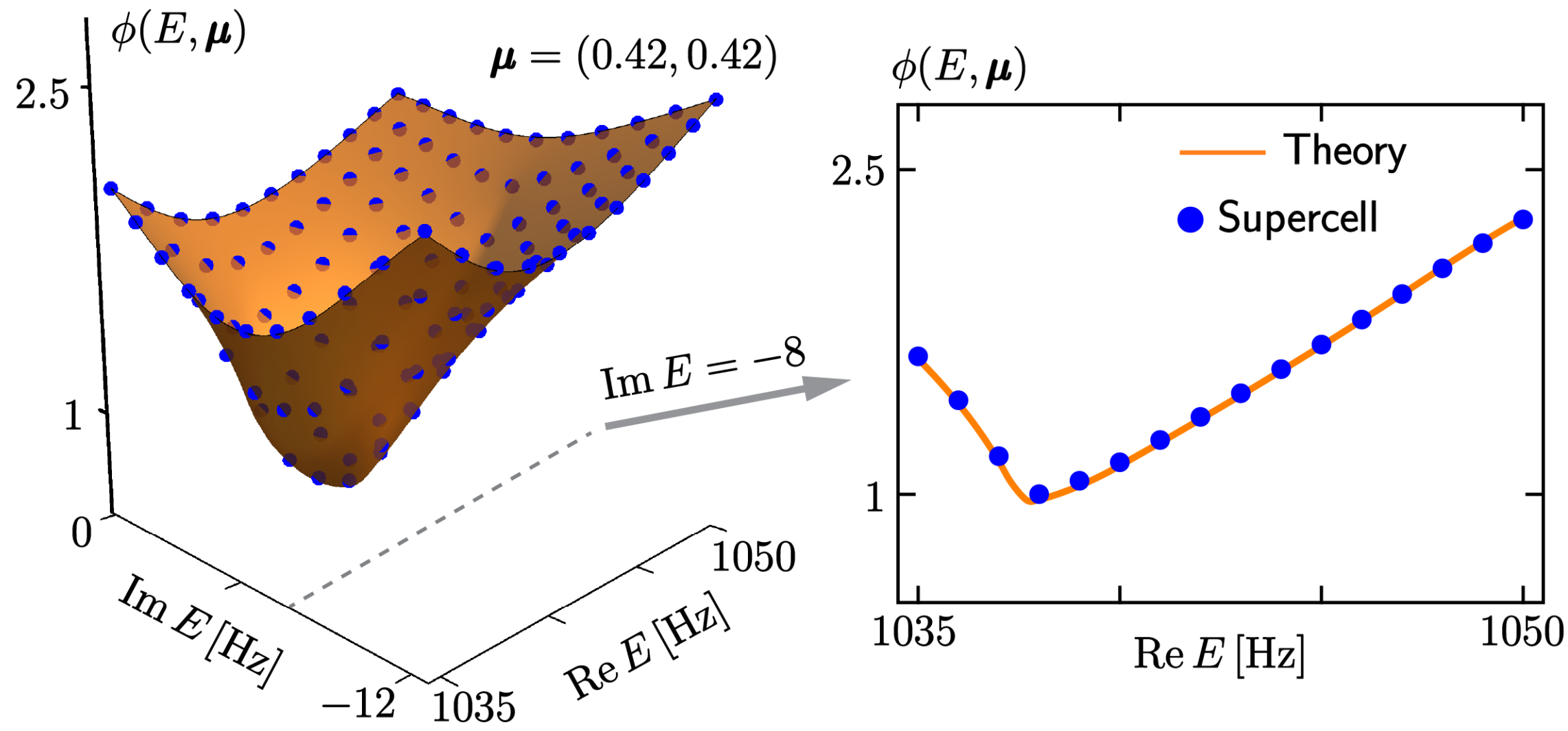

Supplementary Figure 2 | Comparison of spectral potential. Left: spectral potential $\phi(E, \boldsymbol{\mu})$ as a function of Re E and Im E; the orange surface shows the theory, and measured results are overlaid as blue spheres. Right: spectral potential $\phi(E, \boldsymbol{\mu})$ as a function of Re E at Im $E = -8$ (indicated by the dashed line in the left panel). All parameters are the same as those in Fig. 5 of the main text, and $\boldsymbol{\mu} = (0.42, 0.42)$.

Next, we illustrate the computation of the spectral potential. For each fixed $\boldsymbol{\mu}$, the experiment returns a discrete set of complex eigenenergies $\{E_v^{\boldsymbol{\mu}}\}_{v=1}^{N_E}$. We compute the spectral potential [equation (4) in the main text] in discrete form $\phi(E, \boldsymbol{\mu}) = \frac{1}{N_E} \sum_v \log\left|E - E_v^{\boldsymbol{\mu}}\right|$ [equation (4) in the main text]. Figure 2 shows the measured potential for $\mu = 0.42$ (data from Fig. 5b in the main text) and its agreement with the theoretical surface, demonstrating that the k-sampling is sufficiently dense to resolve the complex spectral manifold.

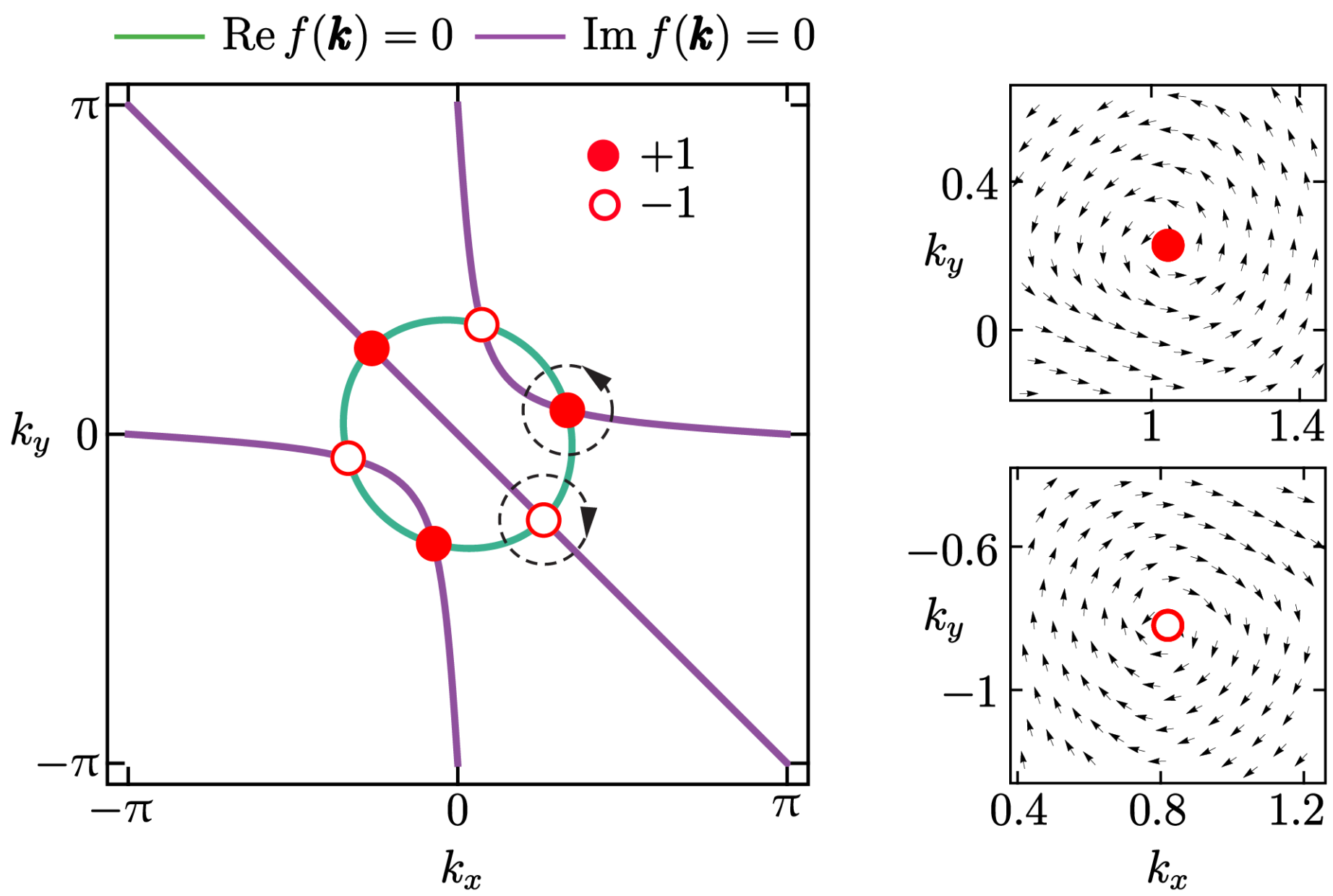

Supplementary Figure 3 | Determining the NBFP sign. The left panel shows six NBFPs at E_3, with filled and open indicating their sign. We determine the sign using the phase gradient $\nabla_{\boldsymbol{k}}\arg[f(\boldsymbol{k})]$ together with the right-hand rule. The solid purple and green lines represent the loci $\mathrm{Re}f(\boldsymbol{k}) = 0$ and $\mathrm{Im}f(\boldsymbol{k}) = 0$. The right panel displays the vector fields of $\nabla_{\boldsymbol{k}}\arg[f(\boldsymbol{k})]$ near two representative NBFPs, and their corresponding senses of rotation are indicated in the left panel.

Finally, we show the extraction of non-Bloch Fermi points (NBFPs) from experimental data and sign assignment. With an established $E^{\mu}(\boldsymbol{k})$ mapping, for any chosen reference energy E_b, we locate the experimental $\boldsymbol{k}$-points with minimal $|\ E^{\mu}(\boldsymbol{k}) - E_b\ |$. Clustered minima (within experimental tolerance) are taken as the NBFPs, as shown in Figs. 5-6 in the main text.

To assign a sign to each NBFP, we examine the phase gradient of the characteristic polynomial $f(\boldsymbol{k}) = \det\left(E - H_{\boldsymbol{\mu}}(\boldsymbol{k})\right)$. Figure 3 (parameters as Fig. 5 in the main text) shows the NBFP distribution for $\mu = 0.42$ and $E_3 = (1045.1 - 6i)$ Hz; two NBFPs are enlarged and overlaid with the normalized phase-gradient vector field $\nabla_k \arg\ f(\mathbf{k})$. The two vortices have opposite circulation; following the convention used in the figures, counterclockwise winding is taken as positive and clockwise as negative, as indicated.

Supplementary Note 2. More experimental details

The non-Bloch supercell framework requires sophisticated control of hoppings to achieve exponent flattening alongside all other hoppings in the model, so we detail experimental implementations in this section.

A. Experimental control of hoppings

The hopping between cavities is implemented using source-detector pairs (loudspeaker-microphone). On our platform, hopping strength and phase are precisely controlled by a digital multi-channel controller, allowing flexible and reconfigurable manipulation of lattice hoppings. As illustrated in Fig. 4a, each unidirectional hopping is realized through a loudspeaker, a microphone, and an audio amplifier. The loudspeaker and microphone are positioned at the bottom of each cavity. The microphone captures the acoustic pressure signal, which is processed by the controller to adjust its phase and amplitude before being emitted by the loudspeaker in the connected cavity. The analytical model to quantify these parameters experimentally will be described later.

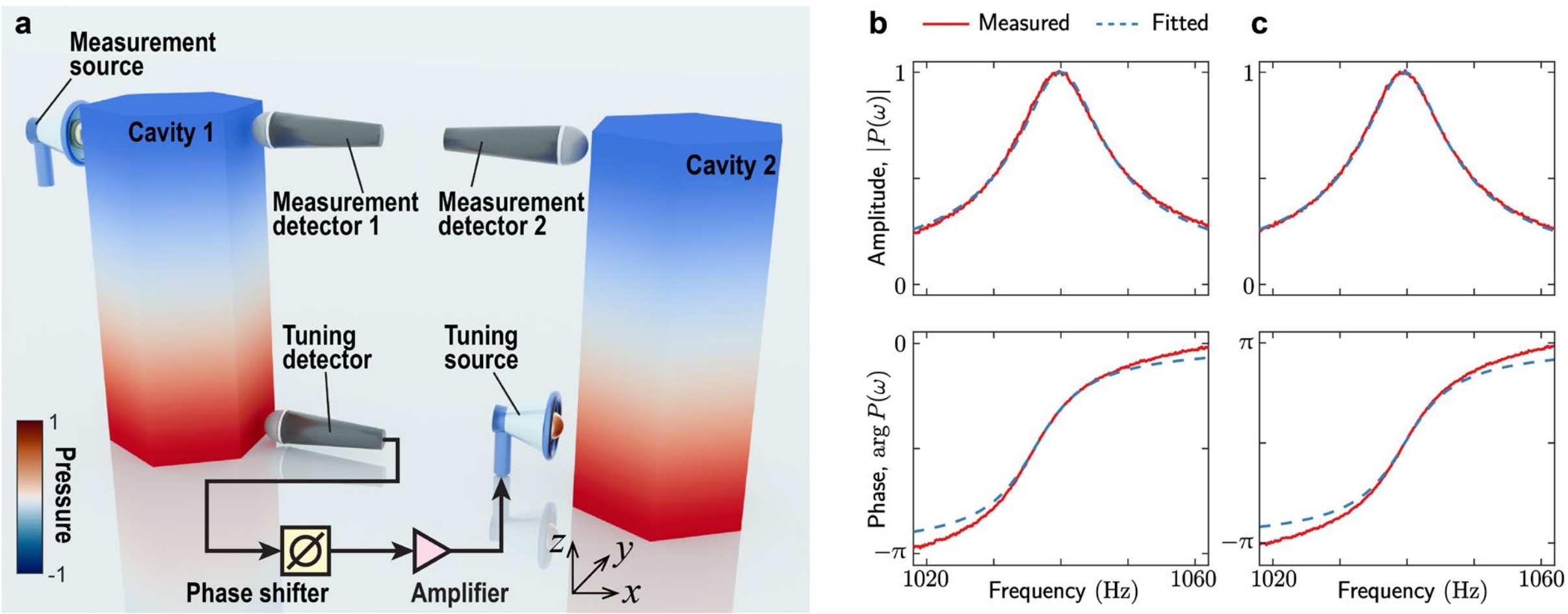

Supplementary Figure 4 | Tuning hoppings between two acoustic cavities. a, Schematic illustration of the unidirectional hopping implemented using a detector and a source. The bottom microphone (tuning detector) in cavity 1 captures the acoustic pressure signal, which is processed by the controller to adjust its phase and amplitude before being emitted by the bottom loudspeaker (tuning source) in cavity 2. **b**,**c**, Experimentally measured and numerically fitted amplitudes (top) and phase responses (bottom) of the cross-power spectral density defined in equation (S5). For configuration **b**, the retrieved hopping strength is $\kappa_0 = 4.5\text{Hz} + 0.05i\text{Hz}$. For configuration **c**, the retrieved hopping strength is $\kappa_0 = 0.02\text{Hz} + 2.26i\text{Hz}$.

The tuning of the hopping parameters is performed as follows. As illustrated in Fig. 4a, a microphone is placed inside cavity 1 to detect the acoustic pressure. The detected pressure signal is then phase-adjusted, amplified, and emitted by the loudspeaker positioned in cavity 2. To determine both the amplitude and phase of the unidirectional hopping, we excite at cavity 1 using a loudspeaker (measurement source in Fig. 4a) and measure the acoustic pressure in both cavities using two microphones (measurement detectors in Fig. 4a).

For quantifying the amplitude and phase of the unidirectional hopping, we suppose a unidirectional hopping κ_0 that connects two acoustic cavities in a tight-binding model Hamiltonian

$$H_2 = \begin{pmatrix} \omega_0 & 0 \\ \kappa_0 & \omega_0 \end{pmatrix}. \tag{S1}$$

Here, $\mathrm{Re}(\omega_0)$ is the first resonant (angular) frequency of a single cavity, and $\mathrm{Im}(\omega_0) < 0$ is the intrinsic loss. In general, $\kappa_0 \in \mathbb{C}$ is a complex number. Based on the coupled-mode theory, the dynamic equation for this system with a source excitation $|s\rangle$ is

$$i\frac{d|\Psi(t)\rangle}{dt} = H|\Psi(t)\rangle + |s(t)\rangle, \tag{S2}$$

where $|\Psi(t)\rangle = [\Psi_1(t) \quad \Psi_2(t)]^T$ is the time-dependent sound pressure vector.

For a harmonic excitation at an angular frequency ω, the excitation and sound pressure vectors can be written as $|s(t)\rangle = |s\rangle e^{-i\omega t}$ and $|\Psi(t)\rangle = |\Psi\rangle e^{-i\omega t}$, respectively. The dynamic equation (S2) is then expressed as

$$(\omega - H_2)|\Psi\rangle = |s\rangle. \tag{S3}$$

When the source is excited at cavity 1, the excitation vector is $|s\rangle = \Psi_{\mathrm{in}}[1\ 0]^T$, where Ψ_{in} is the complex amplitude. Consequently, the sound pressure distribution is obtained by solving equation (S3)

$$|\Psi(t)\rangle = \Psi_{\mathrm{in}} e^{-i\omega t} \left[\frac{1}{\omega - \omega_0} \quad \frac{\kappa_0}{(\omega - \omega_0)^2}\right]^T. \tag{S4}$$

To determine κ_0, the cross-power spectral density between two measured signals (two components of the above equation) is calculated in experiments

$$P(\omega) = \frac{\Psi_2(\omega)}{\Psi_1(\omega)} = \frac{\kappa_0}{\omega - \omega_0}. \tag{S5}$$

It is observed that, when $\omega = \mathrm{Re}(\omega_0)$, the phase of P and κ_0 follows the relation

$$\arg P[\omega = \mathrm{Re}(\omega_0)] = \arg(\kappa_0) - \frac{\pi}{2}. \tag{S6}$$

Figures 4b and 4c present the cross-power spectral density of two measured pressure signals for two representative cases, where the desired hopping strengths are set to $\kappa_0 = 4.5\mathrm{Hz}$ and

$\kappa_0 = 2.25i$Hz. The amplitude responses exhibit a single peak near the resonant frequency of 1040 Hz. At 1040 Hz, the phase responses in Figs. 4b and 4c are approximately $-90°$ and 0°, respectively. These results correspond to hopping phases of $0°$ and $90°$ in the two configurations, consistent with the relationship described in equation (S6). In summary, the measured amplitude and phase responses align well with the fitted curves, confirming the accurate implementation of unidirectional hoppings.

B. Experimental implementation of non-Bloch supercells

Figure 5 illustrates the experimental realization of a representative 2×2 non-Bloch supercell. As shown in Fig. 5a, each cavity is equipped with a loudspeaker (Spk) and a microphone (Mic). The full set of hopping terms and onsite potentials is dynamically programmed via a real-time controller. Another loudspeaker (shown in Cavity 1) is used to excite the lattice, while the responses are measured by microphones. Figure 5b shows the equivalent tight-binding model corresponding to the physical system, making explicit the mapping between hardware and model parameters. Figure 5c presents a photo of the experimental setup. This configuration provides the foundational hardware platform for implementing μ-deformed boundary conditions and performing high-resolution complex spectral measurements.

C. Experimental setup

Figure 6 shows a photo of the whole experimental setup, which comprises 95 acoustic cavities interconnected via the controller. This platform can emulate non-Hermitian lattices with a lattice size of up to 95. To ensure precise tuning, each hopping strength is adjusted individually, while all other hoppings are disabled to prevent interference. Once all hoppings have been tuned, they are simultaneously enabled for subsequent measurements. This method ensures consistent and accurate implementation of the desired hopping parameters across the lattice. Since all hoppings are implemented through the programmable controller, there are no geometric constraints in implementation. For instance, it can realize both one-dimensional and two-dimensional lattices (including rectangular and parallelogram lattices).

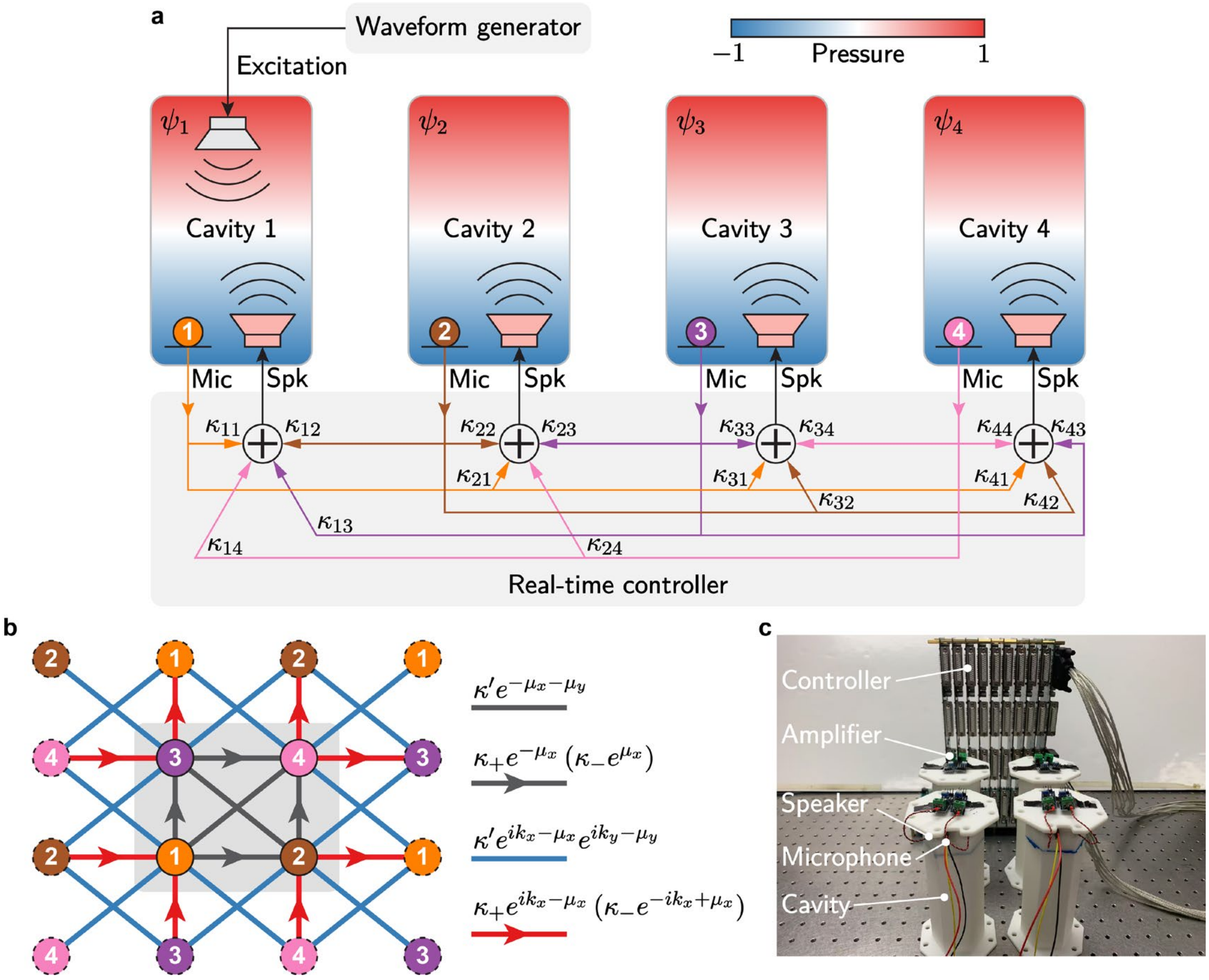

Supplementary Figure 5 | Experimental implementation of a 2×2 supercell. a, Schematic of a 2×2 non-Bloch supercell. Each cavity is equipped with a speaker (Spk) and a microphone (Mic) for actuation and sensing. The onsite potentials (κ_{11}, κ_{22}, κ_{33}, κ_{44}) and hopping terms (κ_{ij}) are dynamically programmed via a real-time controller that processes the microphone signals and drives the speakers accordingly. **b**, Equivalent tight-binding model of the supercell, showing all possible hopping paths and onsite modulations. **c**, A photo of the experimental setup.

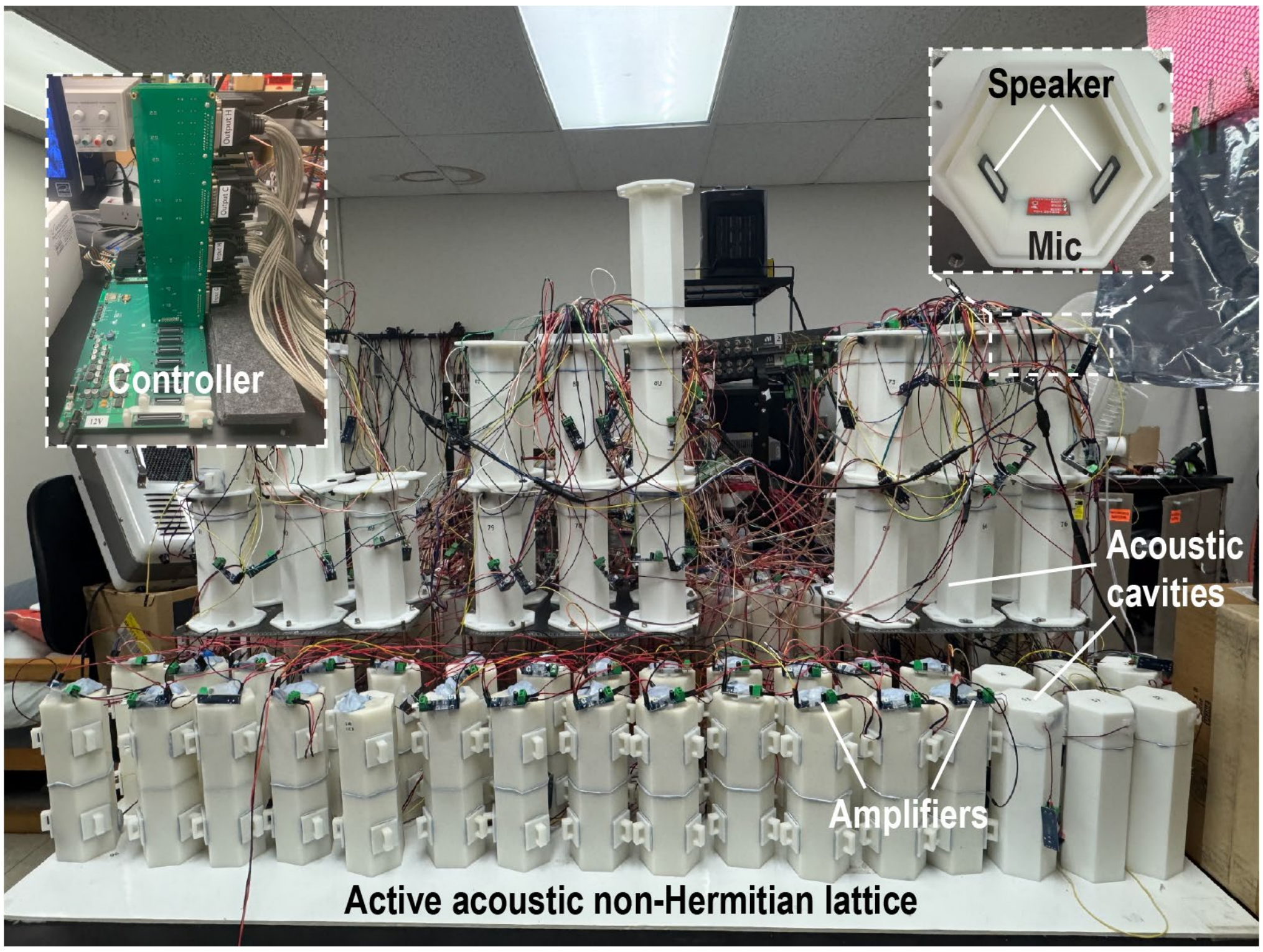

Supplementary Figure 6 | Photos of the experimental setup. The acoustic non-Hermitian lattice consists of 95 acoustic cavities. Onsite potentials and hoppings are implemented using loudspeaker-microphone pairs. The magnitude and phase of hoppings are tuned using a real-time digital controller. A data acquisition system is used to measure the Green's function for all possible combinations of source-detector positions across the entire lattice.

Supplementary Note 3. Point-gap criterion for the open-boundary spectrum

In this section, we detail the point-gap criterion for the open-boundary spectrum stated in the main text and relate it to the conditions established in non-Bloch band theory[1-2]. For simplicity, we denote the open-boundary spectrum as σ_{OBC}, which is defined as the geometry-independent spectral envelope, *i.e.*, the union of open boundary condition (OBC) spectra over all admissible boundary geometries in the large-N limit.

In arbitrary dimensions, there exist two complementary and mathematically equivalent formulations to determine σ_{OBC}, i.e., the amoeba formulation[1] and the uniform-spectrum formulation[2]. The former utilizes amoeba features and spectral potential as indicators, while the latter is based on the connotation of point gaps, a direct generalization of the one-dimensional (1D) scenario. We summarize both below and illustrate our recipe.

We start with the uniform-spectrum formulation, which directly reflects the point-gap topology. The OBC spectrum in this formulation is obtained by the intersection

$$\sigma_{\text{uniform}} \coloneqq \bigcap_{\forall \boldsymbol{\mu}} \text{Sp}(\boldsymbol{\mu}), \tag{S7}$$

where, for each $\boldsymbol{\mu}$, the spectrum $\text{Sp}(\boldsymbol{\mu})$ is defined by

$$\text{Sp}(\boldsymbol{\mu}) = \left\{ E \in \mathbb{C}: \sum\nolimits_{\hat{n},\boldsymbol{k}_\perp} |w_{\hat{n}}(E,\boldsymbol{\mu};\boldsymbol{k}_\perp)| \neq 0 \right\}. \tag{S8}$$

Here, $\hat{n}$ and $\boldsymbol{k}_\perp$ need to run ergodically over all possible values. For the 1D case, $\hat{n} = \hat{x}$ and $\boldsymbol{k}_\perp$ is absent. In general, equation (S8) determines the nontrivial point-gap region for a given $\boldsymbol{\mu}$, and equation (S7) implies that OBC eigenenergies must reside within point gaps for all possible values of $\boldsymbol{\mu}$. Because the nonzero condition only requires the existence of at least one nontrivial $\hat{n}$ and $\boldsymbol{k}_\perp$, we interpret equation (S8) as

$$\text{Sp}(\boldsymbol{\mu}) = \{E \in \mathbb{C}: \exists(\hat{n},\boldsymbol{k}_\perp) \text{ such that } w_{\hat{n}}(E,\boldsymbol{\mu};\boldsymbol{k}_\perp) \neq 0\}. \tag{S9}$$

This is precisely the point-gap criterion stated in the main text. Equivalently, σ_{uniform} is the set of energies whose eigenenergy winding numbers cannot be removed by any choices of $\boldsymbol{\mu}$.

We then introduce the amoeba formulation. Let $f(E,\boldsymbol{\beta}) = \det[E - H(\boldsymbol{\beta})]$ be the characteristic polynomial of the non-Bloch Hamiltonian, where $\boldsymbol{\beta} = (\beta_1, \beta_2, \dots, \beta_d)$ and d is the spatial dimension. For fixed E, the log-moduli of the zero locus of f form the amoeba

$$A_f(E) = \{\boldsymbol{\mu} = \log|\boldsymbol{\beta}| : f(E,\boldsymbol{\beta}) = 0\} \subset \mathbb{R}^d. \tag{S10}$$

The associated Ronkin function is nothing but the spectral potential $\phi(E,\boldsymbol{\mu})$, i.e., equation (4) in the main text, which is the $\boldsymbol{k}$-averaged log-modulus of the polynomial. The amoeba theory links geometric features of $A_f(E)$ and analytic features of $\phi(E,\boldsymbol{\mu})$ to the OBC membership.

Specifically, equations (S7) and (S8) in the amoeba formulation mean that $\phi(E, \boldsymbol{\mu})$ has a minimum without a plateau. Due to the convex characteristics of $\phi(E, \boldsymbol{\mu})$ in the $\boldsymbol{\mu}$ space, the absence of a plateau in $\phi(E, \boldsymbol{\mu})$ signals that E belongs to the OBC spectra. We therefore refer to the amoeba criterion as

$$\sigma_{\text{amoeba}} \coloneqq \{E \in \mathbb{C} : \phi(E, \boldsymbol{\mu}) \text{ has no plateau in } \boldsymbol{\mu}\}, \tag{S11}$$

It is known that the two formulations coincide[2]

$$\sigma_{\text{uniform}} = \sigma_{\text{amoeba}} = \sigma_{\text{OBC}}, \tag{S12}$$

and thus, we use experimental features of spectral potential as the point-gap criterion to predict OBC spectra.

We then demonstrate these equivalences using the examples from the main text. For the 1D example (Fig. 3 in the main text), we choose $E_5 = 1045.0 - 3.0i$, which lies outside σ_{OBC}, and the spectral potential $\phi(E_5, \mu)$ exhibits a plateau (Fig. 7b). As shown by the blue lines in Fig. 7a, the deformed spectral loops $E^{\mu}(k)$ for $\mu = 0.2$ on the plateau does not enclose E_5, consistent with the point-gap criterion. To further verify, we calculate σ_{OBC}, as displayed by green curves in Fig. 7a, by the point-gap criterion, and density of states from the amoeba theory via $\rho(E) = \frac{1}{2\pi}\nabla_E^2\phi(E, \mu_{\text{min}})$, as shown in Fig. 7c. Good agreements show the equivalence in the 1D model.

For the 2D model (Figs. 5-6 in the main text), Fig. 7d shows the predicted OBC spectral region (green area) obtained from the point-gap criterion. Energies chosen outside this region (for example, $E_6 = 1045.0 - 3.0i$) display a plateau in $\phi(E_6, \boldsymbol{\mu})$ (orange surface in Fig. 7e) and a central hole in the amoeba $A_f(E_6)$ (red area in Fig. 7e). The mirror symmetry of the Hamiltonian enforces that both the amoeba and the spectral potential are symmetric with respect to the line $\mu_x = \mu_y$ (Fig. 7e). Because $\phi(E, \boldsymbol{\mu})$ is convex in $\boldsymbol{\mu}$, any global minimizer must lie on this symmetry axis; hence $\boldsymbol{\mu}_{\text{min}}$ satisfies $\mu_x = \mu_y$. The $\boldsymbol{\mu}$-deformed spectrum $E^{\boldsymbol{\mu}}$ for $\boldsymbol{\mu}$ inside the central hole (blue circle in Fig. 7e) fails to enclose the energy E_6 (blue region in Fig. 7d), again demonstrating agreement between the point-gap criterion and spectral potential features in higher dimensions. Figure 7f shows the $\boldsymbol{\mu}_{\text{min}}$ distribution predicted by the amoeba theory, indicating the skin mode localizations.

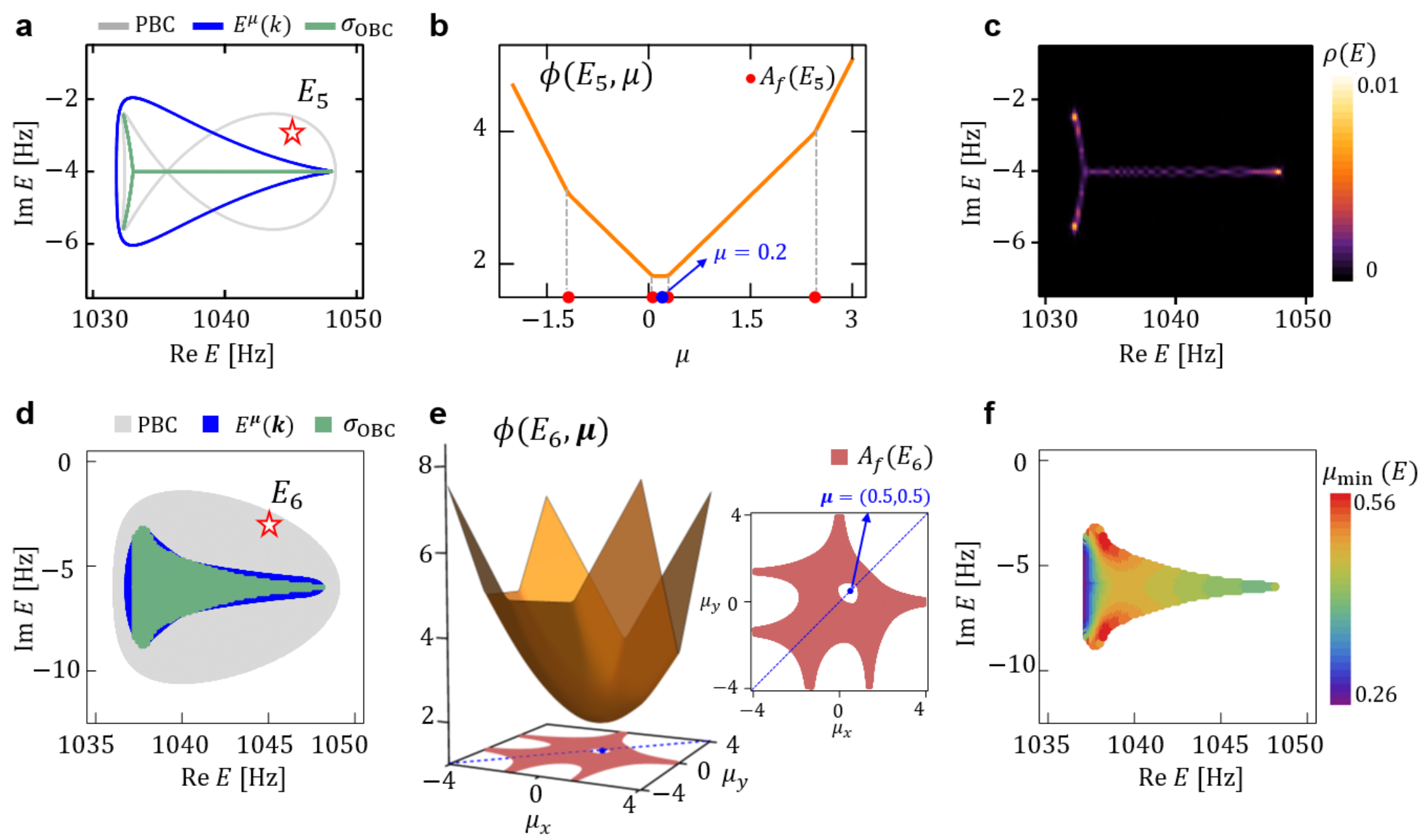

Supplementary Figure 7 | Point-gap criterion and spectral potentials. a, Predicted σ_{OBC} for the 1D model (green curve) obtained from the point-gap criterion. The grey curve is the spectrum under periodic boundary conditions (PBCs), and the blue curve shows the μ-deformed dispersion $E^{\mu}(k)$ for $\mu = 0.2$ (indicated by the blue marker in panel **b**). The red star marks the reference energy E_5. **b**, Spectral potential $\phi(E_5,\mu)$ for the 1D model. Red circles are the solutions of $\{\mu = \log|\beta| : f(E_5,\beta) = 0\}$, i.e., the amoeba $A_f(E_5)$. **c**, Density of states computed from amoeba theory, $\rho(E) = \frac{1}{2\pi}\nabla_E^2\phi(E,\mu_{\min})$. **d**, Predicted σ_{OBC} (green) for the two-dimensional (2D) model obtained from the point-gap criterion. The grey area is the PBC spectrum; the blue area corresponds to $E^{\boldsymbol{\mu}}(\boldsymbol{k})$ at $\boldsymbol{\mu} = (0.5,0.5)$ (marked by the blue point in panel **e**). The red star marks the reference energy E_6. **e**, Spectral potential $\phi(E_6,\boldsymbol{\mu})$ (orange surface) and the amoeba $A_f(E_6)$ (shaded red) for the 2D model. The right panel shows a close-up of the boxed region in the left panel; the solid line laid on the amoeba marks the symmetry axis $\mu_x = \mu_y$. **f**, Map of minimizers $\boldsymbol{\mu}_{\min}(E)$ computed by amoeba theory. $\boldsymbol{\mu}_{\min}(E)$ are locations of spectral-potential minima in the $\boldsymbol{\mu}$ space for given E. White regions denote energies for which the potential exhibits a plateau.

Supplementary Note 4. Relation between spectral potential and averaged winding number along arbitrary directions

For a fixed reference energy E, the spectral potential (Ronkin function) is defined by equation (4) of the main text, namely,

$$\phi(E,\boldsymbol{\mu}) = \int \left(\frac{dk}{2\pi}\right)^d \log\left|\det\left[E - H\left(e^{\boldsymbol{\mu}}e^{i\boldsymbol{k}}\right)\right]\right|. \tag{S13}$$

Spectral potential $\phi(E,\boldsymbol{\mu})$ is a full k-space average and therefore independent of any choice of projection. By contrast, the eigenenergy winding number is computed along 1D loops and thus depends on the chosen projection direction.

Noting $\beta_j = e^{\mu_j + ik_j}$, the chain rule yields the operator identity

$$\partial_{\mu_j} = \beta_j \partial_{\beta_j} = -\mathrm{i}\partial_{k_j}. \tag{S14}$$

Applying ∂_{μ_j} to ϕ and using equation (S14) gives

$$\partial_{\mu_j}\phi(E,\boldsymbol{\mu}) = \mathrm{Re}\int \left(\frac{dk}{2\pi}\right)^d \left(-i\partial_{k_j}\right)\log\det\left[E - H\left(e^{\boldsymbol{\mu}}e^{i\boldsymbol{k}}\right)\right]. \tag{S15}$$

By fixing the transverse momenta $\boldsymbol{k}_\perp = (k_1, \cdots k_{j-1}, k_{j+1}, \cdots k_d)$, the integral over k_j can be evaluated as a contour integral in the complex e^{ik_j}-plane, and it yields[1]

$$\partial_{\mu_j}\phi(E,\boldsymbol{\mu}) = \int \left(\frac{dk_\perp}{2\pi}\right)^{d-1} w_{k_j}(E,\boldsymbol{\mu};\boldsymbol{k}_\perp) = \langle w_{k_j}(E,\boldsymbol{\mu};\boldsymbol{k}_\perp)\rangle_{\boldsymbol{k}_\perp}, \tag{S16}$$

where $w_{k_j}(E,\boldsymbol{\mu};\boldsymbol{k}_\perp)$ is the eigenenergy winding number computed along the k_j-loop at fixed $\boldsymbol{k}_\perp$ [equation (3) of the main text] and $\langle\cdot\rangle_{\boldsymbol{k}_\perp}$ denotes averaging over $\boldsymbol{k}_\perp$.

We then consider the gradient of the spectral potential and the eigenenergy winding number along an arbitrary direction $\hat{n}$. Writing the gradient $\boldsymbol{g}(\boldsymbol{\mu}) = \nabla_{\boldsymbol{\mu}}\phi$ componentwise gives

$$\boldsymbol{g}(\boldsymbol{\mu}) = \left(\partial_{\mu_1}, \partial_{\mu_2}, \cdots, \partial_{\mu_d}\right)\phi(E,\boldsymbol{\mu}) = -i\left(\partial_{k_1}, \partial_{k_2}, \cdots, \partial_{k_d}\right)\phi(E,\boldsymbol{\mu}), \tag{S17}$$

and projecting onto the unit direction $\hat{n}$ yields

$$\hat{n}\cdot\boldsymbol{g} = -i\partial_{k_{\hat{n}}}\phi(E,\boldsymbol{\mu}) = \langle w_{\hat{n}}(E,\boldsymbol{\mu};\boldsymbol{k}_\perp)\rangle_{\boldsymbol{k}_\perp}, \tag{S18}$$

where $\partial_{k_{\hat{n}}} = \hat{n}\cdot\left(\partial_{k_1}, \partial_{k_2}, \cdots, \partial_{k_d}\right)$. Equation (S18) shows the directional derivative of the spectral potential along $\hat{n}$ equals the $k_\perp$-averaged eigenenergy winding number computed for loops parallel to $\hat{n}$. This is the relation between spectral potential and averaged winding number along arbitrary directions used in the main text.

To verify equation (S18) numerically, we choose a reference energy E_3 (Fig. 8a, same as Fig. 5 in the main text) and evaluate $\phi(E_3,\boldsymbol{\mu})$ over $\boldsymbol{\mu}$ (Fig. 8b). At a chosen off-symmetry point $\boldsymbol{\mu} = (0.3, 0.1)$, the gradient is $\boldsymbol{g}(\boldsymbol{\mu}) = (-0.359593, -0.999592)$. We then select four

projection directions $\hat{n} = \hat{x}, \hat{y}, (\hat{x}+\hat{y})/\sqrt{2}$, and $(\hat{x}-\hat{y})/\sqrt{2}$. For each direction, we construct the corresponding family of 1D loops (Fig. 8c-f, right) and compute the eigenenergy winding number distribution $w_{\hat{n}}(E_6, \boldsymbol{\mu}; k_\perp)$ (Fig. 8c-f, left). Vertical dashed lines mark the transverse momenta where loops cross non-Bloch Fermi points (NBFPs), leading to discrete eigenenergy winding number jumps. Based on Figs. 8c to 8f, we compare the averaged winding numbers with $\hat{n} \cdot \boldsymbol{g}$, i.e., $\langle w_{\hat{x}} \rangle_{\boldsymbol{k}_\perp} = -0.359911 \sim g_x = -0.359593$, $\langle w_{\hat{y}} \rangle_{\boldsymbol{k}_\perp} = -1.00750 \sim g_y = -0.999592$, $\langle w_{(\hat{x}+\hat{y})/\sqrt{2}} \rangle_{\boldsymbol{k}_\perp} = -0.325136 \sim \boldsymbol{g} \cdot (\hat{x}+\hat{y})/\sqrt{2} = -0.324952$, and $\langle w_{(\hat{x}-\hat{y})/\sqrt{2}} \rangle_{\boldsymbol{k}_\perp} = -0.183244 \sim \boldsymbol{g} \cdot (\hat{x}-\hat{y})/\sqrt{2} = -0.183589$. The excellent agreement confirms equation (S18) numerically.

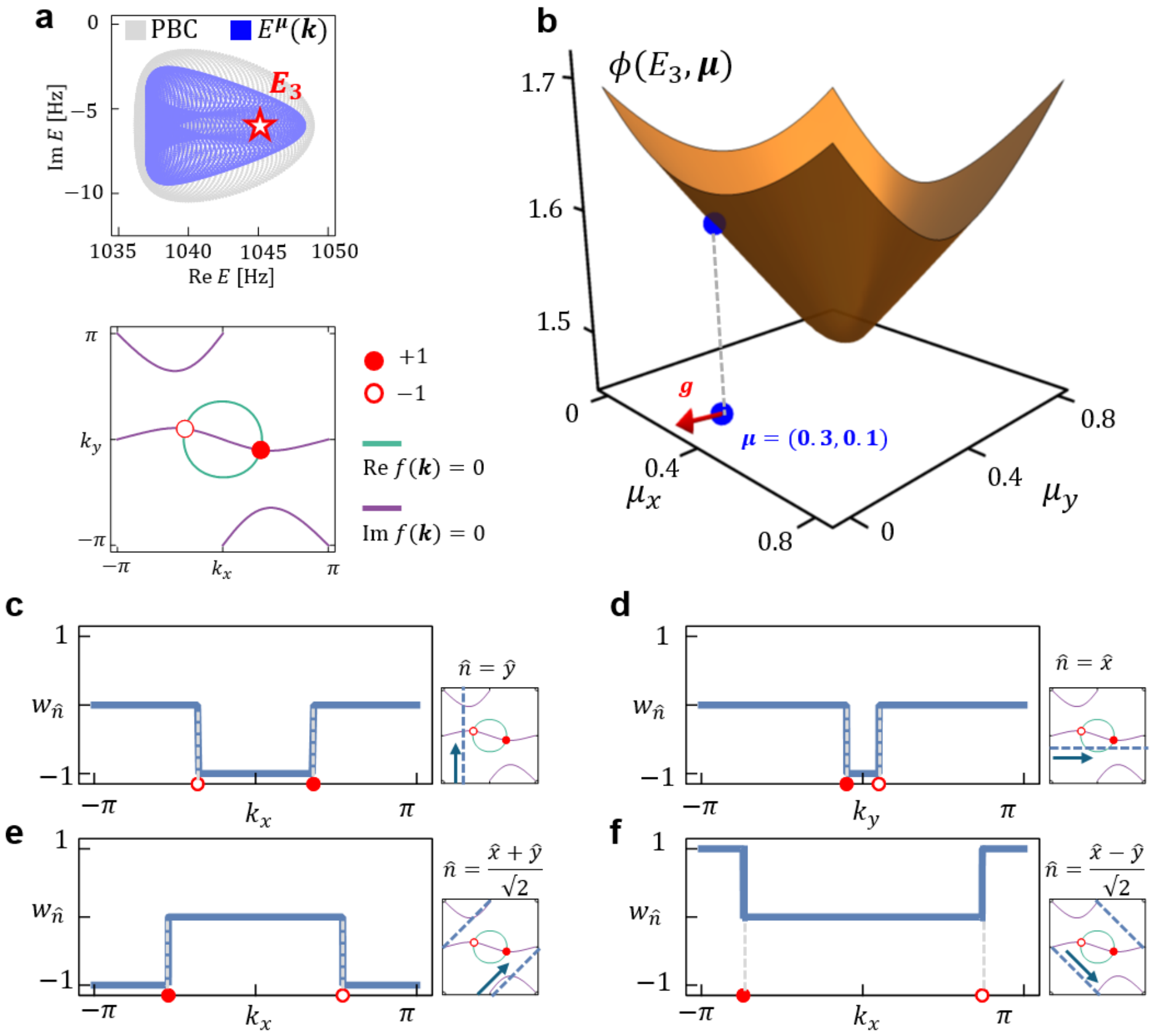

Supplementary Figure 8 | Spectral potential and eigenenergy winding number distributions. a, Reference energy E_3 (top) and its NBFPs (bottom). Top: the $\boldsymbol{\mu}$-deformed spectral $E^{\boldsymbol{\mu}}(\boldsymbol{k})$ (blue region); the red star marks E_3 (same as in Fig. 5 of the main text). The grey region denotes the PBC spectrum. Bottom: the two NBFPs corresponding to E_3 and $\boldsymbol{\mu} = (0.3{,}0.1)$; filled (open) circles indicate NBFPs with positive (negative) sign. The solid purple and green curves show the loci $\mathrm{Re} f(\boldsymbol{k}) = 0$ and $\mathrm{Im} f(\boldsymbol{k}) = 0$, whose intersections yield the NBFPs. **b**, Spectral potential $\phi(E_3, \boldsymbol{\mu})$ (orange surface). The blue marker indicates $\boldsymbol{\mu} = (0.3{,}0.1)$, and its gradient $\boldsymbol{g} = \nabla_{\boldsymbol{\mu}}\phi$ is shown by the red arrow. **c-f**, Eigenenergy winding number distributions for four families of projected 1D loops (projection directions $\hat{n}$ schematically indicated in the right-hand insets). Each panel plots $w_{\hat{n}}(E_3, \boldsymbol{\mu}; k_\perp)$ as a function of transverse momentum. Gray dashed vertical lines mark transverse momenta where the loop crosses an NBFP and the winding number exhibits a discrete jump.

Supplementary Note 5. Equivalence of the supercell recipe to the auxiliary generalized Brillouin zone criterion in 1D systems

We now formalize the relation between spectral self-intersections, NBFPs, and auxiliary generalized Brillouin zones (aGBZs) in 1D non-Hermitian systems. We first define the self-intersection energy set as

$$\sigma_{\text{self}} = \{E \in \mathbb{C} \mid \exists \mu \text{ and } \exists\, k_1 \neq k_2, \text{such that } H_\mu(k_1) = H_\mu(k_2) = E\}, \tag{S19}$$

where $H_\mu(k)$ is the mapping introduced in Fig. 1 of the main text. Equivalently, σ_{self} consists of those energies E for which, for some μ and band α, the band dispersion $E_\alpha^\mu(k)$ (as a function of real momentum k) satisfies $E = E_\alpha^\mu(k_1) = E_\alpha^\mu(k_2)$ with $k_1 \neq k_2$. In other words, $E_\alpha^\mu(k)$ exhibits a self-intersection, for example, E_3, E_4 in Fig. 3. Physically speaking, any $E \in \sigma_{\text{self}}$ implies that, at that μ, at least two distinct real momenta map to the same energy, and these momenta are precisely NBFPs for that μ.

The self-intersection condition is equivalent to the standard aGBZ formulation used in the literature[3,4]. For a 1D tight-binding model, the characteristic equation at energy E reads $f(\beta = e^{ik+\mu}, E) = \det[E - H(\beta)] = 0$, whose $p+q$ roots $\{\beta_i\}_{i=1}^{p+q}$ (at fixed E) are ordered by modulus $|\beta_1| \leq |\beta_2| \leq \cdots \leq |\beta_{p+q}|$. Here p (q) is the maximal hopping range to the right (left), so that the polynomial degree is $p+q$. The aGBZ criterion states that E belongs to the aGBZ energy set σ_{aGBZ} if there exists an index i for which $|\beta_i| = |\beta_{i+1}|$. Therefore, the aGBZ energy set can be formally expressed as

$$\sigma_{\text{aGBZ}} = \{E \in \mathbb{C} \mid \exists i, \text{ with } |\beta_i| = |\beta_{i+1}| = e^\mu\}. \tag{S20}$$

Generally, the real parts of β_i and β_{i+1} are distinct (hence different real momenta k) while sharing the same modulus e^μ, so the condition $|\beta_i| = |\beta_{i+1}|$ is equivalent to the self-intersection condition, $\sigma_{\text{aGBZ}} = \sigma_{\text{self}}$. Physically speaking, at energy E, there are (at least) two exponential solutions with the identical decay/growth rate μ that can interfere to satisfy OBCs. The conventional generalized Brillouin zone (GBZ) relevant for thermodynamic OBC spectra is the subset defined by the special index $i = p$, i.e., $|\beta_p| = |\beta_{p+1}|$. We denote the GBZ spectrum by σ_{GBZ}, with $\sigma_{\text{GBZ}} \subset \sigma_{\text{aGBZ}}$.

In experiments, one typically measures $E_\alpha^\mu(k)$ for a finite range of μ and cannot directly recover all algebraic β-roots at each E. To distinguish σ_{GBZ} from σ_{aGBZ} in practice, we therefore use the point-gap criterion and the spectral potential, as described in the main text.

Supplementary Note 6. Density of non-Bloch Fermi points and spectral moments

We have introduced the NBFP density $\boldsymbol{\rho_{\mu}(E)}$ in the main text, and in this section, we detail its calculations and relations with spectral moments. We first verify the relation between $\boldsymbol{\rho_{\mu}(E)}$ and the spectral potential $\boldsymbol{\phi(E,\mu)}$. Let the characteristic determinant factorize (consider the multi-band case) as

$$\det\left[E - H_{\boldsymbol{\mu}}(\boldsymbol{k})\right] = \prod_{\alpha}\left(E - E_{\alpha}^{\boldsymbol{\mu}}(\boldsymbol{k})\right), \tag{S21}$$

with the band index α and $\boldsymbol{k} = (k_1, \cdots, k_d)$. d is the system dimension. We then have

$$\log\left|\det\left[E - H_{\boldsymbol{\mu}}(\boldsymbol{k})\right]\right| = \sum_{\alpha}\log\left|E - E_{\alpha}^{\boldsymbol{\mu}}(\boldsymbol{k})\right|. \tag{S22}$$

Using the identity in the complex plane[5]

$$\partial_z\partial_{z^*}\log zz^* = \pi\delta(x)\delta(y), \quad z = x + iy\,, \qquad z^* = x - iy, \tag{S23}$$

one can get

$$\nabla_z^2\log|z| = \left(\partial_x^2 + \partial_y^2\right)\log|z| = 4\partial_z\partial_{z^*}\log\left(\sqrt{zz^*}\right) \ = 2\pi\delta(x)\delta(y), \tag{S24}$$

where $\partial_z = \frac{(\partial_x - i\partial_y)}{2}$ and $\partial_{z^*} = \frac{(\partial_x + i\partial_y)}{2}$. Using equations (S21)-(S24), we obtain

$$\begin{aligned}\rho_{\boldsymbol{\mu}}(E) &= \frac{1}{2\pi}\nabla_E^2\phi(E,\boldsymbol{\mu}) = \frac{1}{2\pi}\nabla_E^2\int\left(\frac{dk}{2\pi}\right)^d\log\left|\det\left[E - H_{\boldsymbol{\mu}}(\boldsymbol{k})\right]\right| \\ &= \frac{1}{2\pi}\int\left(\frac{dk}{2\pi}\right)^d\nabla_E^2\sum_{\alpha}\log\left|E - E_{\alpha}^{\boldsymbol{\mu}}(\boldsymbol{k})\right| \\ &= \int\left(\frac{dk}{2\pi}\right)^d\sum_{\alpha}\delta\left(\mathrm{Re}\,E - \mathrm{Re}\,E_{\alpha}^{\boldsymbol{\mu}}(\boldsymbol{k})\right)\delta\left(\mathrm{Im}\,E - \mathrm{Im}\,E_{\alpha}^{\boldsymbol{\mu}}(\boldsymbol{k})\right).\end{aligned} \tag{S25}$$

For a generic energy E, the kernel of the delta functions in equation (S25) is the set

$$K_{\boldsymbol{\mu}}(E) = \{\boldsymbol{k} \in \mathrm{BZ}\,|E_{\alpha}^{\boldsymbol{\mu}}(\boldsymbol{k}) = E\}, \tag{S26}$$

which is precisely the set of NBFPs defined in equation (1) of the main text. For 2D systems, $K_{\boldsymbol{\mu}}(E)$ consists of a discrete set of points $\{\boldsymbol{k}_q\}_{q=1}^{N_K}$. Near an isolated root $\boldsymbol{k}_q$, the mapping $\boldsymbol{k} \mapsto (\mathrm{Re}\,E^{\boldsymbol{\mu}}(\boldsymbol{k}), \mathrm{Im}\,E^{\boldsymbol{\mu}}(\boldsymbol{k}))$ has a Jacobian matrix

$$J_q = \frac{\partial(\mathrm{Re}\,E, \mathrm{Im}\,E)}{\partial(k_1, \cdots, k_d)}|_{\boldsymbol{k}_q}. \tag{S27}$$

By changing variables in equation (S25) from $\boldsymbol{k}$ to E, we obtain

$$\rho_{\boldsymbol{\mu}}(E) = \sum_q\frac{1}{(2\pi)^2\left|\det\left[J_q\right]\right|}, \ \text{ for } d = 2, \tag{S28}$$

showing that $\rho_{\boldsymbol{\mu}}(E)$, the density of NBFPs on the complex-E plane, is inversely proportional to the Jacobian determinant that encodes the local dispersion around each NBFP.

For dimension $d > 2$, the preimage $K_{\boldsymbol{\mu}}(E)$ is generically a $(d-2)$-dimensional smooth submanifold of the Brillouin zone. In this case, introduce the Gram matrix

$$G_{jj'}(\boldsymbol{k}) = \partial_{k_j}\mathrm{Re}\, E^{\boldsymbol{\mu}}(\boldsymbol{k})\partial_{k_{j'}}\mathrm{Re}\, E^{\boldsymbol{\mu}}(\boldsymbol{k}) + \partial_{k_j}\mathrm{Im}\, E^{\boldsymbol{\mu}}(\boldsymbol{k})\partial_{k_{j'}}\mathrm{Im}\, E^{\boldsymbol{\mu}}(\boldsymbol{k}), \tag{S29}$$

which is the metric induced on the normal bundle of the level set. The delta-function integral in equation (S25) then reduces to an integral over the $(d-2)$-dimensional manifold $K_{\boldsymbol{\mu}}(E)$

$$\rho_{\boldsymbol{\mu}}(E) = \int_{K_{\boldsymbol{\mu}}(E)} \frac{d^{d-2}S(\boldsymbol{k})}{(2\pi)^d}\frac{1}{\sqrt{\det G(\boldsymbol{k})}}, \quad \text{for } d > 2, \tag{S30}$$

where $d^{\,d-2}S(\boldsymbol{k})$ denotes the natural surface element on $K_{\boldsymbol{\mu}}(E)$ induced from the Brillouin-zone measure. Equations (S28) and (S30) are equivalent, namely, equation (S28) is the special case of equation (S30) when the integration manifold consists of isolated points.

Finally, in numerical or experimental practice, the integral in equation (S25) is approximated either by finite sampling of k-points or by a sampled set of eigenvalues $\{E_v^{\mu}\}_{v=1}^{N_E}$

$$\rho_{\boldsymbol{\mu}}(E) = \sum\nolimits_v \delta\left(E - \mathrm{Re}\, E_v^{\boldsymbol{\mu}}\right)\delta\left(E - \mathrm{Im}\, E_v^{\boldsymbol{\mu}}\right)/N_E\,. \tag{S31}$$

The continuum expressions (S28)–(S30) clarify why the experimentally observed counts of NBFPs (and their local dispersion through $\det J_q$ or $\det G$) directly reflect the spectral density.

We then turn to the spectral moments associated with $\rho_{\boldsymbol{\mu}}(E)$. The complex spectral moments are formally defined as

$$M_n = \int (E - E_{\mathrm{ref}})^n \rho_{\boldsymbol{\mu}}(E) d^2E\,, n \in \mathbb{N}, \tag{S32}$$

where E_{ref} is the reference energy. Using equation (S25), we obtain

$$M_n = \int \left(\frac{dk}{2\pi}\right)^d \sum\nolimits_\alpha \left(E_\alpha^{\boldsymbol{\mu}}(\boldsymbol{k}) - E_{\mathrm{ref}}\right)^n = \int \left(\frac{dk}{2\pi}\right)^d \mathrm{Tr}\left(\left(H_{\boldsymbol{\mu}}(\boldsymbol{k}) - E_{\mathrm{ref}}\right)^n\right). \tag{S33}$$

Crucially, M_n is independent of $\boldsymbol{\mu}$ because

$$\partial_{\mu_j}M_n = -\mathrm{i}\partial_{k_j}M_n = \int \left(\frac{dk}{2\pi}\right)^d \left(-\mathrm{i}\partial_{k_j}\right)\mathrm{Tr}\left(\left(H_{\boldsymbol{\mu}}(\boldsymbol{k}) - E_{\mathrm{ref}}\right)^n\right) = 0, \tag{S34}$$

where we used $\partial_{\mu_j} = -\mathrm{i}\,\partial_{k_j}$ and the periodicity of the Brillouin zone to drop the total derivative. Hence, $\partial_{\mu_j}M_n = 0$ for all j, so M_n does not depend on $\boldsymbol{\mu}$. Reference[6] further shows that the spectral moments computed from the OBC spectrum are

$$\int (E - E_{\mathrm{ref}})^n \rho_{\mathrm{OBC}}(E) d^2E = \int \left(\frac{dk}{2\pi}\right)^d \mathrm{Tr}\left(\left(H_{\boldsymbol{\mu}}(\boldsymbol{k}) - E_{\mathrm{ref}}\right)^n\right) = M_n\,. \tag{S35}$$

So, the moments M_n are thermodynamic invariants that can be computed from the μ-deformed supercell data and used to predict OBC measurements.

Figure 9 compares several low-order spectral moments M_n computed in three independent ways: (i) from the theoretical μ-deformed band structure (lines), (ii) from the experimentally reconstructed μ-deformed spectrum via the supercell method (circles), and (iii) from the open-boundary spectra (triangles). Here, we evaluate moments with the reference energy $E_{\mathrm{ref}} = (1040.0 - 6.0\,i)$ Hz. As expected from the theory, M_n is independent of $\boldsymbol{\mu}$. The three datasets are in good agreement, demonstrating that the spectral moments are conserved and can be obtained equivalently from either supercell reconstructions or OBC measurements. The experimental uncertainty increases with n because higher moments amplify the influence of measurement noise, finite k-sampling, and limited sample size in the spectrum.

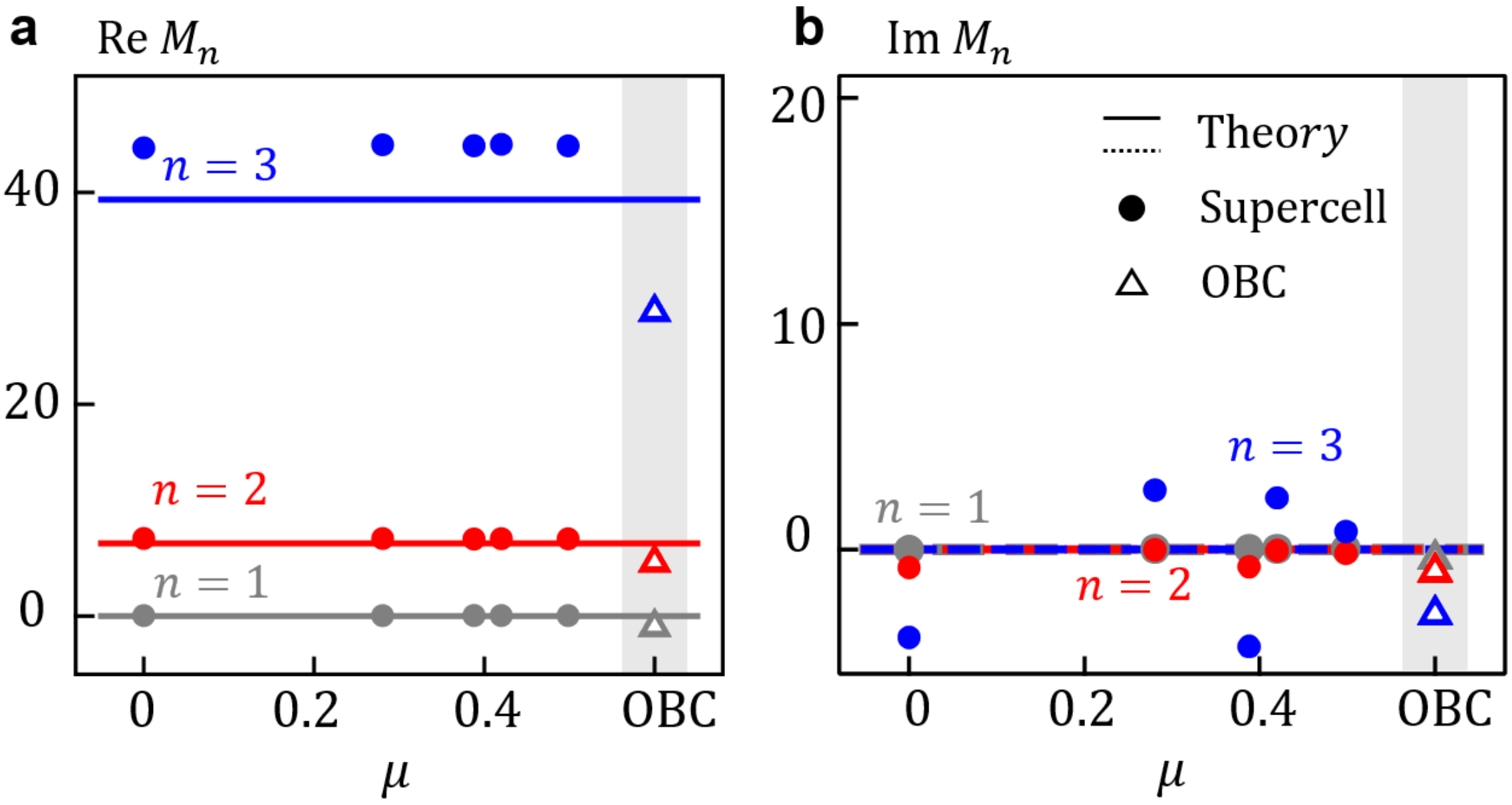

Supplementary Figure 9 | Spectral moments. Real (**a**) and imaginary (**b**) parts of the spectral moments M_n for orders $n = 1,2,3$. Solid and dashed lines show the real and imaginary parts of the theoretical moments computed from the Bloch Hamiltonian via $\int_{\mathrm{BZ}} \mathrm{Tr}\,[(H(\boldsymbol{k}) - E_{\mathrm{ref}})^n]$; circles denote moments obtained from the non-Bloch supercell measurements; triangles denote moments computed directly from the experimental OBC spectra. All experimental data are presented in Figs. 5-6 of the main text.

Supplementary Note 7. Extended experimental results of point-gap patterns observed in 2D non-Hermitian lattices

Extended experimental results of point-gap patterns along $\hat{n} = \hat{y}$ and $\hat{n} = (\hat{x} + \hat{y})/\sqrt{2}$ are presented in Figs. 10 and 11, respectively. In both figures, the measurements are taken at discrete values of k_x ranging from $-14\pi/15$ to π in steps of $\pi/15$.

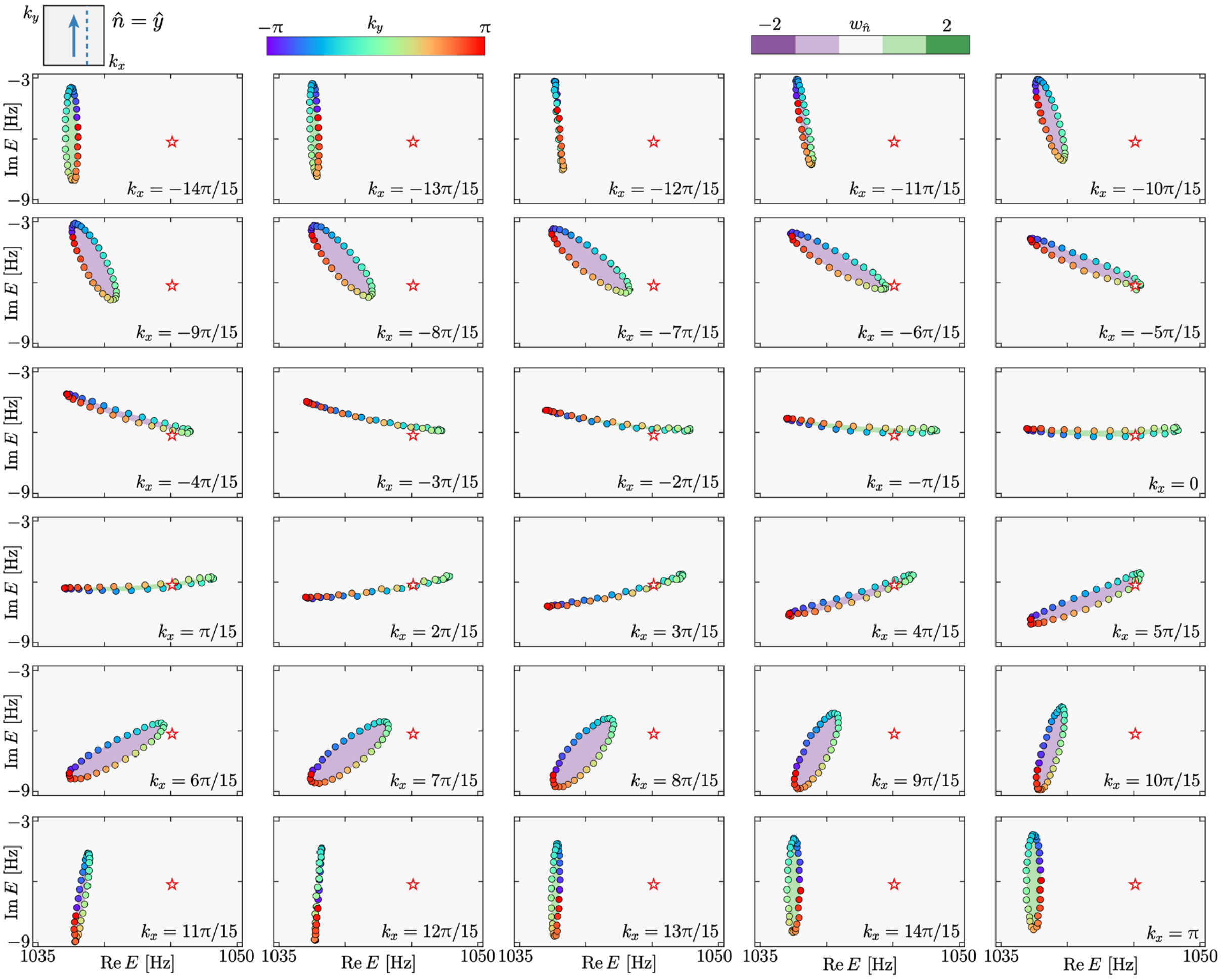

Supplementary Figure 10 | Experimental point-gap patterns along $\hat{n} = \hat{y}$ with k_x-parametrized loops. All panels correspond to $\mu = 0.42$, and plotting conventions follow Fig. 5 of the main text. The star indicates the energy at $E_3 = (1045.1 - 6.1i)$Hz.

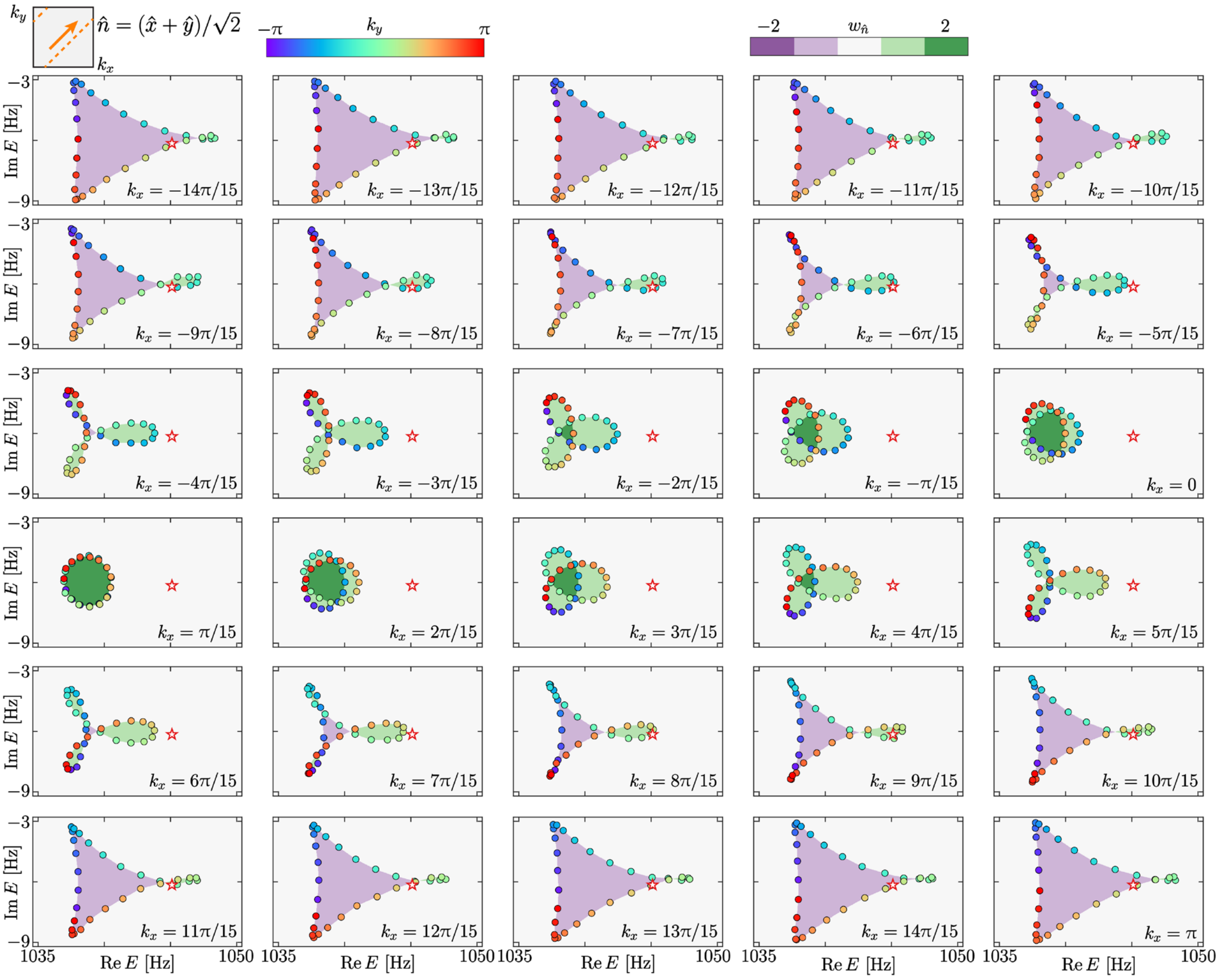

Supplementary Figure 11 | Experimental point-gap patterns along $\hat{\boldsymbol{n}} = (\hat{\boldsymbol{x}} + \hat{\boldsymbol{y}})/\sqrt{2}$ with $\boldsymbol{k_x}$-parametrized loops. All panels correspond to $\mu = 0.42$, and plotting conventions follow Fig. 5 of the main text. The star indicates the energy at $E_3 = (1045.1 - 6.1i)$Hz.

Supplementary Note 8. NBFP-compatible standing waves

The distribution of NBFPs for a given energy is an intrinsic property of the non-Hermitian band structure and independent of any particular open-boundary geometry. How different NBFPs interfere to form standing-wave patterns that satisfy boundary conditions depends sensitively on many details, making a complete description, in general, highly complicated and challenging. However, by employing symmetry and focusing on specific symmetric systems, we can predict possible interference patterns for a given boundary geometry.

A. Energy windows of NBFP-compatible standing waves

We take the example shown in Figs. 5 and 6 of the revised main text. For the energy window depicted by the solid line in Fig. 12a, the procedure developed in the main text determines the corresponding $\boldsymbol{\mu}_{min}$ and the associated NBFP distributions for each energy. As an example, the six NBFPs corresponding to E_b are shown as circles in Fig. 12b and are labeled sequentially as $\boldsymbol{k}_1$ to $\boldsymbol{k}_6$ with $\boldsymbol{\mu}_{min} = (0.42{,}0.42)$. Owing to the symmetry of the model, these six NBFPs are symmetrically distributed with respect to both $k_x = k_y$ and $k_x = -k_y$. Within the energy window of interest, the number and symmetry of NBFPs are the same as E_b.

In principle, any pair of NBFPs can interfere to form a standing-wave pattern along the direction connecting the two NBFPs, thereby satisfying the corresponding OBCs. To make the discussion transparent, we focus on the two high-symmetry directions, namely, $\hat{n}_1 = (\hat{x} - \hat{y})/\sqrt{2}$ and $\hat{n}_2 = (\hat{x} + \hat{y})/\sqrt{2}$.

Figure 12b shows that six NBFPs are certainly three pairs, i.e., $\{\boldsymbol{k}_1, \boldsymbol{k}_2\}$, $\{\boldsymbol{k}_4, \boldsymbol{k}_5\}$, and $\{\boldsymbol{k}_3, \boldsymbol{k}_6\}$, which are protected by the symmetry with respect to $k_x = k_y$. Another symmetry with respect to $k_x = -k_y$ implies that $\{\boldsymbol{k}_1, \boldsymbol{k}_2\}$ and $\{\boldsymbol{k}_4, \boldsymbol{k}_5\}$ are correlated, and hence, the six NBFPs naturally divide into two groups, i.e., group $a = \{\boldsymbol{k}_1, \boldsymbol{k}_2, \boldsymbol{k}_4, \boldsymbol{k}_5\}$ and group $b = \{\boldsymbol{k}_3, \boldsymbol{k}_6\}$.

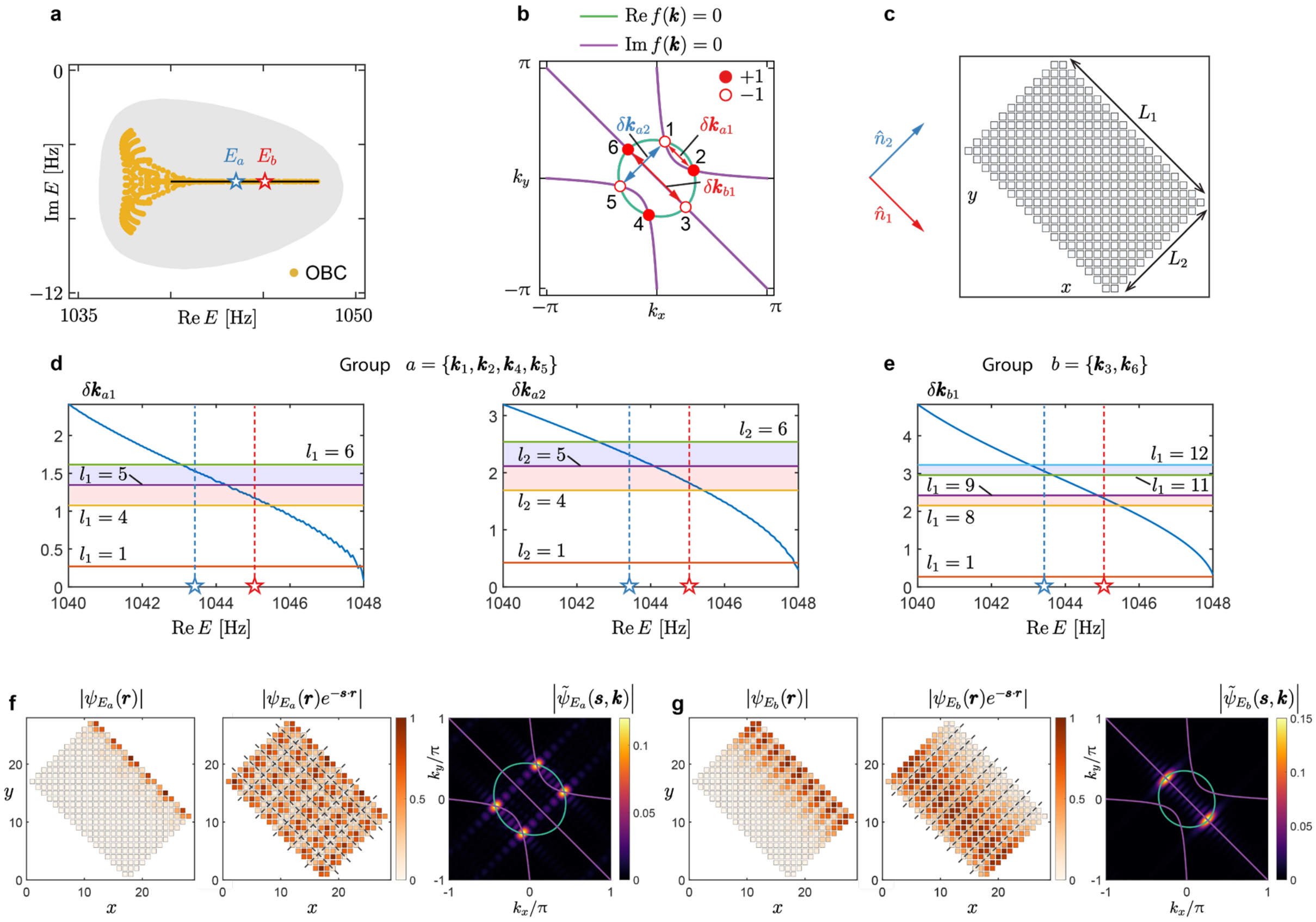

Supplementary Figure 12 | NBFP-compatible standing waves. a, PBC (gray) and OBC (blue) spectra with the energy window (black line), $\mathrm{Im}\, E = -6.0\,\mathrm{Hz}$ and $\mathrm{Re}\, E \in [1040.0, 1048.0]\mathrm{Hz}$. Red and blue stars mark the selected energy E_a and E_b, with the corresponding $\boldsymbol{\mu}_{\min} = (0.44, 0.44)$ and $(0.42, 0.42)$, respectively. **b**, NBFP distribution at E_b for $\boldsymbol{\mu}_{\min}$. The six NBFPs (red circles) are labeled $1 \rightarrow 6$ in clockwise order. **c**, Schematic of an OBC geometry. The two edges are oriented along $\hat{n}_1 = (\hat{x} - \hat{y})/\sqrt{2}$ and $\hat{n}_2 = (\hat{x} + \hat{y})/\sqrt{2}$, with lengths $L_1 = 16.5\sqrt{2}$ and $L_2 = 10.5\sqrt{2}$, respectively. **d**,**e**, Energy dependence of the momentum separations between the NBFP pairs in group a, $\{\boldsymbol{k}_3, \boldsymbol{k}_6\}$ (**d**), and group b, $\{\boldsymbol{k}_1, \boldsymbol{k}_2, \boldsymbol{k}_4, \boldsymbol{k}_5\}$ (**e**), i.e., δk_{a1}, δk_{b1}, and δk_{b2} defined in **b**. The horizontal lines denote the quantized momentum separations satisfying the standing-wave condition, i.e., $2\pi l_{1(2)}/L_{1(2)}$, with $l_{1(2)} \in \mathbb{N}$. The vertical dashed lines mark the selected energies E_a and E_b. The blue (red) shaded region indicates the interval in which the momentum separations corresponding to E_a (E_b) lie. **f**,**g**, Eigenstate amplitude (left), compensated wavefunctions $|e^{-\boldsymbol{s}\cdot\boldsymbol{r}}\psi_E(\boldsymbol{r})|$ with corresponding $\boldsymbol{s} = \boldsymbol{\mu}_{\min}$ (middle), and s-resolved momentum-space wavefunction $|\tilde{\psi}_E(\boldsymbol{s}, \boldsymbol{k})|$ (right) at E_a (**f**) and E_b (**g**). Dashed lines are plotted to indicate the interference nodes of $|e^{-\mathbf{s}\cdot\mathbf{r}}\psi_E(\boldsymbol{r})|$. Gray circles indicate the NBFP positions obtained from the non-Bloch supercell.

Group a consists of the four NBFPs $\{\boldsymbol{k}_1, \boldsymbol{k}_2, \boldsymbol{k}_4, \boldsymbol{k}_5\}$, corresponding to two symmetry-related interference channels along the $\hat{n}_1$ and $\hat{n}_2$ directions. Since the momentum separation between paired NBFPs determines the interference pattern, we define

$$\delta k_{a1} = (\boldsymbol{k}_5 - \boldsymbol{k}_4) \cdot \hat{n}_1 = (\boldsymbol{k}_1 - \boldsymbol{k}_2) \cdot \hat{n}_1, \quad \delta k_{a2} = (\boldsymbol{k}_1 - \boldsymbol{k}_5) \cdot \hat{n}_2 = (\boldsymbol{k}_2 - \boldsymbol{k}_4) \cdot \hat{n}_2, \tag{S36}$$

as illustrated in Fig. 12b. The corresponding δk_{a1} and δk_{a2} as functions of $\mathrm{Re}\, E$ within the energy window in Fig. 12a are shown in the left and right panels of Fig. 12d, respectively. Similarly, group b consists of the NBFP pair $\{\boldsymbol{k}_3, \boldsymbol{k}_6\}$. We define

$$\delta k_{b1} = (\boldsymbol{k}_6 - \boldsymbol{k}_3) \cdot \hat{n}_1, \;\; \delta k_{b2} = (\boldsymbol{k}_6 - \boldsymbol{k}_3) \cdot \hat{n}_2 \equiv 0. \tag{S37}$$

The corresponding δk_{b1} as a function of $\mathrm{Re}\, E$ is shown in Fig. 12e. To evaluate the possible OBC wavefunction patterns for the geometry shown in Fig. 12c, we consider the standing-wave conditions

$$\delta k\, L_1 = 2\pi l_1 \quad \text{and} \quad \delta k\, L_2 = 2\pi l_2, \tag{S38}$$

which indicate that the corresponding interference channels can support standing-wave patterns with l_1 and l_2 interference nodes (excluding the boundary nodes) along the $\hat{n}_1$ and $\hat{n}_2$ directions, respectively. The horizontal lines in Figs. 12d and 12e correspond to the quantized values $2\pi l_{1(2)}/L_{1(2)}$ for several values of $l_{1(2)}$, thereby indicating the energy windows in which NBFP-compatible standing waves can arise.

B. Standing-wave nodes inferred from NBFPs under symmetry constraints

For the energy window of interest, there exist several possible standing wave channels, and therefore, without explicitly enforcing the boundary conditions, we cannot predict the OBC wavefunctions at a particular energy. However, examining the energy lying between distinct l_1 and l_2 values can still indicate possible standing-wave patterns, such as the presence of nodes, for an OBC geometry shown in Fig. 12c.

Taking E_a as an example, if the boundary selects group a, both corresponding δk_{a1} and δk_{a2} fall between the $l_{1(2)} = 5$ and 6 lines in Fig. 12d. Therefore, the OBC eigenstate is expected to exhibit approximately 5 to 6 interference nodes along both the $\hat{n}_1$ and $\hat{n}_2$ directions. By contrast, if the boundary selects group b, δk_{b1} falls between the $l_1 = 11$ and 12 lines in Fig. 12e, whereas $l_2 \equiv 0$. Consequently, the corresponding OBC eigenstate is expected to exhibit approximately 11 to 12 interference nodes along the $\hat{n}_1$ direction while 0 interference nodes along the $\hat{n}_2$ direction.

Figure 12f shows the corresponding OBC wavefunction $\psi_{E_a}(\boldsymbol{r})$ (left) and its compensated wavefunction $\left|e^{-\boldsymbol{s}\cdot\boldsymbol{r}}\psi_{E_a}(\boldsymbol{r})\right|$ (middle) with $\boldsymbol{s} = \boldsymbol{\mu}_{\min}$ to compensate for the exponential decay. A clear standing-wave pattern is observed, exhibiting 5 interference nodes along the $\hat{n}_1$ direction and 5 nodes along the $\hat{n}_2$ direction. This agrees well with the above prediction from NBFPs and standing waves, indicating that the OBC eigenstate is formed by the interference of the NBFP pair in group a, $\{\boldsymbol{k}_1, \boldsymbol{k}_2, \boldsymbol{k}_4, \boldsymbol{k}_5\}$, as further confirmed by the s-resolved momentum-space wavefunction $\left|\tilde{\psi}_E(\boldsymbol{s}, \boldsymbol{k})\right|$ shown in the right of Fig. 12f, whose hot spots coincide with the predicted NBFP pair. To demonstrate another NBFP group, i.e., group $b = \{\boldsymbol{k}_3, \boldsymbol{k}_6\}$, we have used another energy E_b, and the results are shown in Fig. 12g. Clearly, $\{\boldsymbol{k}_3, \boldsymbol{k}_6\}$ has been seen in the interference pattern and the s-resolved momentum-space wavefunction.

C. Experimental verification of the inferred standing-wave nodes

We now use the experimental example in Fig. 6 of the revised main text to perform the above analysis to verify the NBFP-inferred standing-wave nodes. By simply replacing the system lengths with the corresponding values of $L_1 = 9.5\sqrt{2}$ and $L_2 = 3.5\sqrt{2}$, as illustrated in Fig. 13a, we calculate interference indices for E_3 and E_4 under this OBC geometry, as shown in Figs. 13b and 13c, respectively. Figures 13d and 13e present the corresponding experimental results for E_4 and E_3. In each figure, the upper-left inset shows the measured OBC wavefunction, while the left panel shows its s-resolved momentum-space wavefunction $\left|\tilde{\psi}_E(\boldsymbol{s}, \boldsymbol{k})\right|$. The right panels display the compensated wavefunction $e^{-\boldsymbol{s}\cdot\boldsymbol{r}}\psi_E(\boldsymbol{r})$, with the experimental result shown in the upper panel and the theoretical result in the lower panel. For E_4 (Fig. 13d), the compensated wavefunction exhibits four nodes along $\hat{n}_1$ and two along $\hat{n}_2$, identifying the interference channel associated with group a (Fig. 13b), consistent with the $\left|\tilde{\psi}_{E_4}(\boldsymbol{s}, \boldsymbol{k})\right|$. By contrast, E_3 (Fig. 13e) exhibits four nodes along $\hat{n}_1$ and none along $\hat{n}_2$, identifying the interference channel associated with group b (Fig. 13c), again in agreement with the $\left|\tilde{\psi}_{E_3}(\boldsymbol{s}, \boldsymbol{k})\right|$.

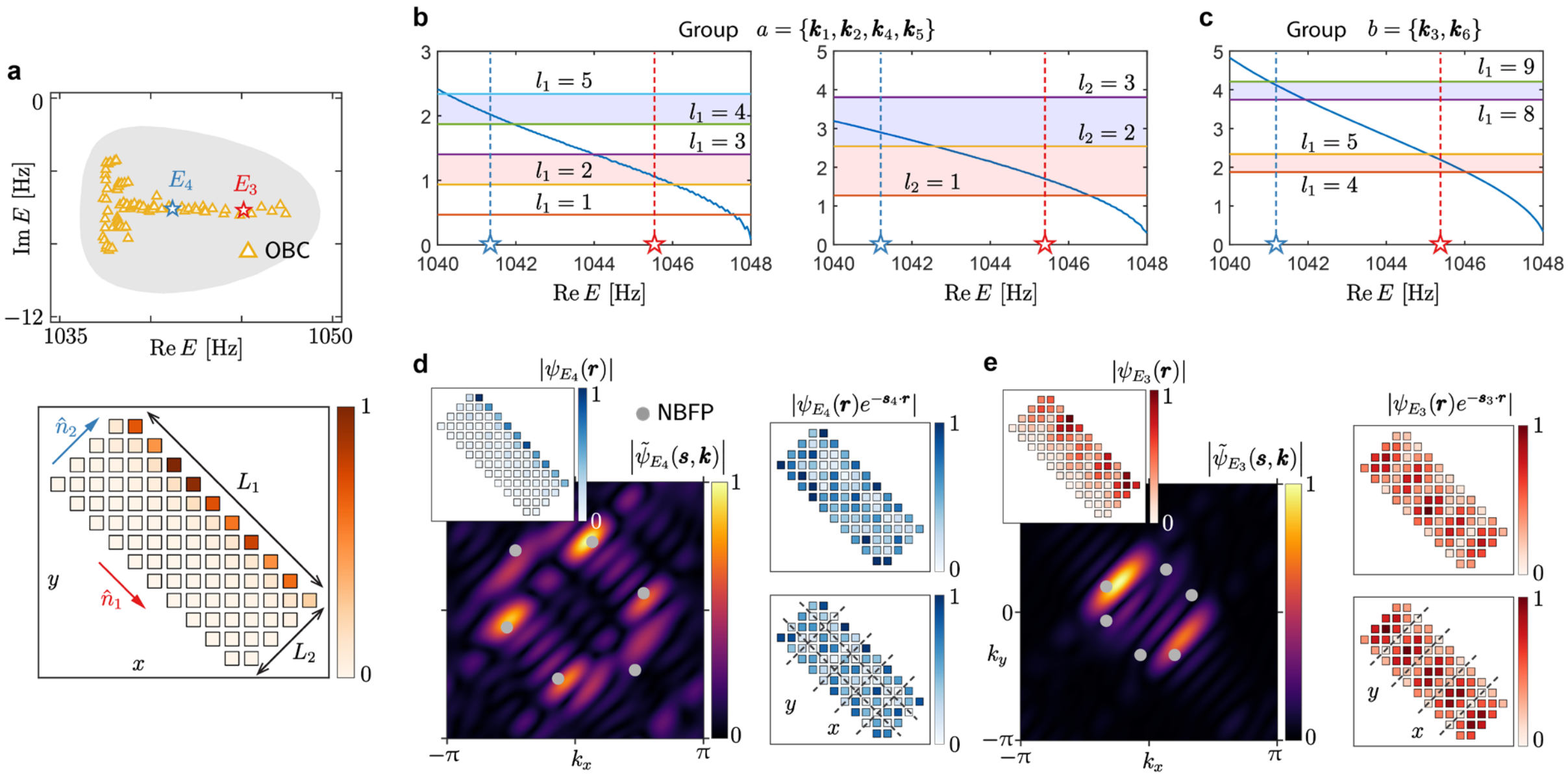

Supplementary Figure 13 | Experimental verification of NBFP-inferred standing-wave nodes. a, OBC spectra (top) and schematic of the OBC geometry (bottom) with lengths $L_1 = 9.5\sqrt{2}$ and $L_2 = 3.5\sqrt{2}$. **b**,**c**, Energy dependence (solid line in Fig. 12a) of the momentum separations between the NBFP pairs in group a, $\{\boldsymbol{k}_1, \boldsymbol{k}_2, \boldsymbol{k}_4, \boldsymbol{k}_5\}$ (**b**), and group b, $\{\boldsymbol{k}_3, \boldsymbol{k}_6\}$ (**c**), i.e., δk_{a1}, δk_{a2}, and δk_{b1} defined in Fig. 12b. The horizontal lines denote the quantized momentum separations satisfying the standing-wave condition, i.e., $2\pi l_{1(2)}/L_{1(2)}$, with $l_{1(2)} \in \mathbb{N}$. The vertical dashed lines mark the selected energies E_3 and E_4. The red (blue) shaded region indicates the interval in which the momentum separations corresponding to E_3 (E_4) lie. **d**,**e**, s-resolved momentum-space wavefunctions $|\tilde{\psi}_E(\boldsymbol{s}, \boldsymbol{k})|$ (left panels) for $E = E_4$ (**d**) and $E = E_3$ (**e**). Grey circles indicate NBFP positions obtained from non-Bloch supercells. Insets show measured real-space eigenstates. The right panels display compensated wavefunctions $|e^{-\boldsymbol{s}\cdot\boldsymbol{r}}\psi_E(\boldsymbol{r})|$ for $E = E_4$ (**d**) and $E = E_3$ (**e**) with corresponding $s = \mu_{\min} = 0.47$ and 0.42. The top and bottom panels show experimental and theoretical results, respectively. Dashed lines are plotted to indicate the interference nodes of $|e^{-\mathbf{s}\cdot\mathbf{r}}\psi_E(\boldsymbol{r})|$.

In summary, while NBFPs alone do not uniquely determine the exact OBC wavefunction for a given energy and boundary geometry, their distribution, when combined with the relevant symmetry constraints, can identify NBFP-compatible standing waves and thereby suggest possible nodal patterns that can be tested experimentally.

Supplementary Note 9. Using NBFPs to distinguish different classes of skin effects

A key ingredient of our framework is the decoupling of the real and imaginary components of momentum through exponent flattening. This ultimately reduces the problem of probing non-Hermitian band structures to the characterization of NBFPs. As shown below, the NBFP provides a unified framework for understanding and distinguishing different classes of skin effects through their wavefunction distributions and momentum-space signatures.

A. Exponentially localized skin modes: signatures of $\boldsymbol{\mu}$ and NBFPs in OBC wavefunctions

Although the programmable-hopping implementation demonstrated in this work is not directly transferable to conventional electronic solids, the underlying k - μ decoupling perspective remains applicable. Once OBC eigenstates are experimentally accessible, one can analyze their localization properties through imaginary-momentum compensation and the corresponding Fourier-Laplace transform, thereby revealing signatures of $\boldsymbol{\mu}$ and the underlying NBFPs.

First, the k-μ decoupling implies that we can perform $e^{-\boldsymbol{s}\cdot\boldsymbol{r}}\psi_E(\boldsymbol{r})$ to an OBC eigenstate $\psi_E(\boldsymbol{r})$ for investigating its decay behavior. Experimentally measuring eigenstates is now feasible through techniques such as Green's-function measurements[7] and state tomography[8]. We therefore begin with an OBC eigenstate to illustrate our point. Figures 14a and 14b depict a staggered Hatano-Nelson model and one OBC eigenstate $\psi_{E_1}(\boldsymbol{r})$, respectively. Introducing an imaginary-momentum compensation factor $e^{-\boldsymbol{s}\cdot\boldsymbol{r}}$, we define the inverse participation ratio (IPR) to the resulting $e^{-\boldsymbol{s}\cdot\boldsymbol{r}}\psi_E(\boldsymbol{r})$

$$\mathrm{IPR} = \frac{\int \left|e^{-\boldsymbol{s}\cdot\boldsymbol{r}}\psi_E(\boldsymbol{r})\right|^4 d\boldsymbol{r}}{\left(\int \left|e^{-\boldsymbol{s}\cdot\boldsymbol{r}}\psi_E(\boldsymbol{r})\right|^2 d\boldsymbol{r}\right)^2}. \tag{S39}$$

Figure 14c shows the corresponding IPR as a function of $\boldsymbol{s}$. Since smaller values of IPR correspond to more extended states, we seek values of $\boldsymbol{s}$ for which the compensated wavefunction $e^{-\boldsymbol{s}\cdot\boldsymbol{r}}\psi_E(\boldsymbol{r})$ becomes as close as possible to an extended state. For exponentially localized OBC wavefunctions, an appropriate compensation can effectively restore the state toward a clean extended profile, as illustrated in the bottom panel of Fig. 14d, where the IPR approaches $1/N$. In short, the behavior of the IPR minimum for $e^{-\boldsymbol{s}\cdot\boldsymbol{r}}\psi_E(\boldsymbol{r})$ dictate the localization nature of the eigenstate.

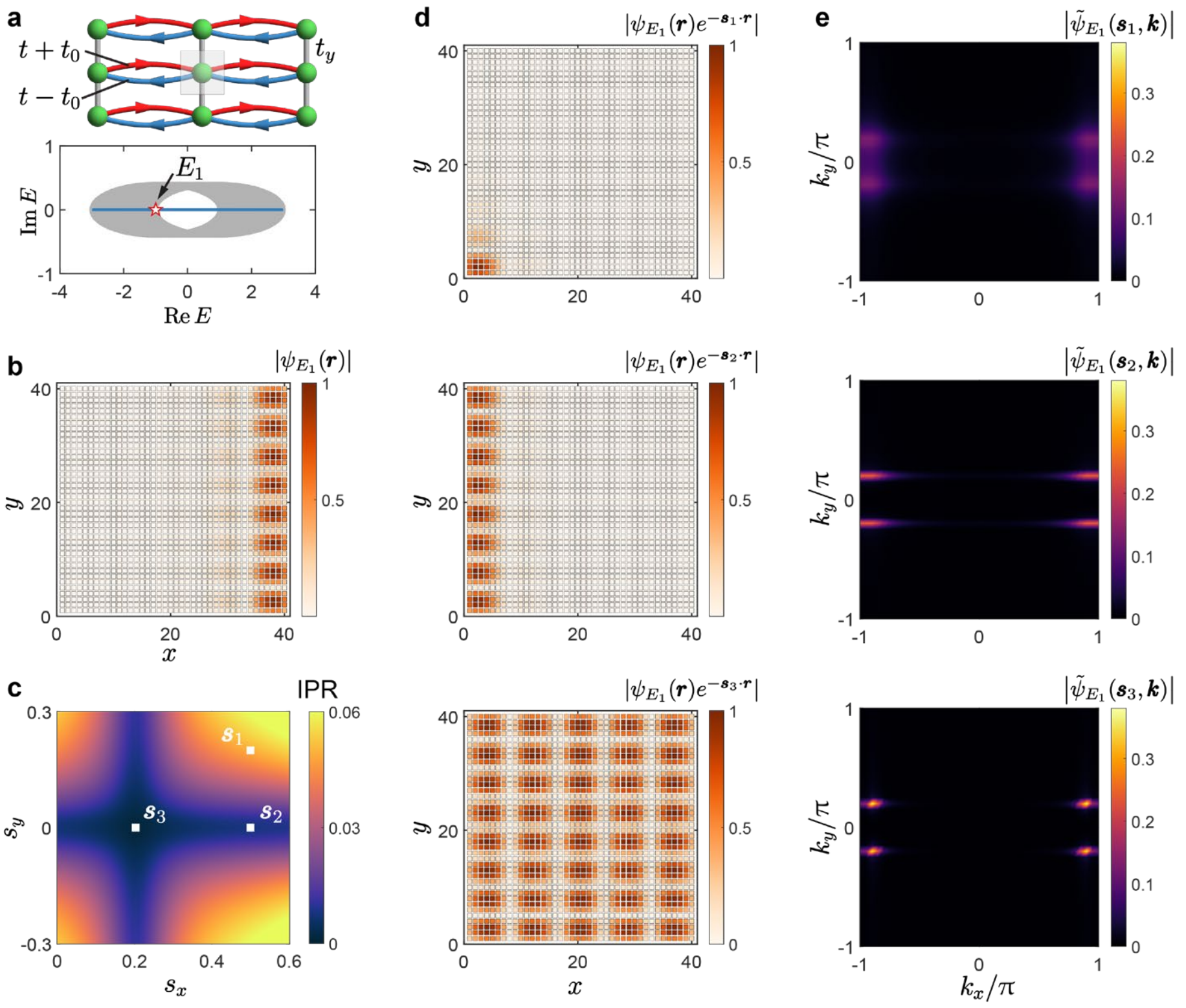

Supplementary Figure 14 | Insights for probing non-Hermitian systems without programmable hopping. a, Tight-binding model (top) and OBC spectrum (bottom). The Hamiltonian is given by $H_{\mathrm{HN}}(\boldsymbol{\beta}) = (t - t_0)\beta_x + (t + t_0)\beta_x^{-1} + t_y(\beta_y + \beta_y^{-1})$. **b**, An OBC eigenstate $|\psi_{E_1}(\boldsymbol{r})|$ with $E_1 = -1.0$, marked by the star in the top panel, which typically exhibits exponential localization toward the boundary in non-Hermitian systems. **c**, Inverse participation ratio (IPR) of the compensated wavefunction $e^{-\boldsymbol{s}\cdot\boldsymbol{r}}\psi_{E_1}(\boldsymbol{r})$. The IPR reaches its minimum at $\boldsymbol{s} = \boldsymbol{s}_3$. **d**, Wavefunctions compensated by different values of $\boldsymbol{s}$, *i.e.*, $\boldsymbol{s}_1 = (0.5, 0.2)$, $\boldsymbol{s}_2 = (0.5, 0.0)$, and $\boldsymbol{s}_3 = (0.2, 0.0)$ .The imaginary-momentum compensation amplifies information that would otherwise be obscured by exponential decay. **e**, Fourier transform of the compensated wavefunction. When $\boldsymbol{s}$ reaches the optimal compensation value, the Fourier intensity is maximized, generating hot spots in momentum space corresponding to the NBFPs selected by the boundary. The parameters used are $t = 1$, $t_0 = 0.2$, and $t_y = 0.5$.

Second, NBFPs provide an experimentally meaningful observable for detecting complex momentum, regardless of whether the hopping parameters are programmable. Performing an s-resolved Fourier-Laplace transform on the eigenstate yields

$$\tilde{\psi}_E(\boldsymbol{s}, \boldsymbol{k}) = \int d\boldsymbol{r}\, [e^{-\boldsymbol{s}\cdot\boldsymbol{r}}\psi_E(\boldsymbol{r})]e^{-i\boldsymbol{k}\cdot\boldsymbol{r}}, \quad \text{(S40)}$$

which corresponds to Eq. (5) in the main text and is illustrated in Fig. 14c. The resulting hot spots in momentum space—namely the Fourier peaks obtained at the compensation parameter

where the IPR is minimized—correspond to the NBFPs. As demonstrated in Figs. 3 and 6 of the main text, $|\tilde{\psi}_E(\boldsymbol{s},\boldsymbol{k})|$ is maximized when the compensation parameter $\boldsymbol{s}$ matches the optimal value $\boldsymbol{\mu}_{\min}$, corresponding to the case where the exponentially localized wavefunction is most effectively restored toward an extended state. The locations of these NBFPs in the $\boldsymbol{k}$-space reflect the underlying interference structure of wavefunctions. In this way, one can investigate the imaginary part of momentum and thereby infer the full complex wavevector $\boldsymbol{k}+i\boldsymbol{\mu}$.

B. Non-Exponentially localized skin modes

The critical skin effect in 1D systems provides a representative example of a non-exponentially localized skin mode. As demonstrated by the critical-skin experiment as shown in Fig. 15 (corresponding to the model discussed in Fig.4 of the main text), one cannot reliably extract μ from finite-size OBC eigenstates $\psi_E(x)$ for chain A and chain B as shown in the top panels of Fig. 15c-d. The s-resolved Fourier-Laplace transform of the eigenstates on chains A and B are shown in the left panels of Figs. 15e and 15f, respectively. The resulting hot spots in momentum space, namely, the Fourier peaks obtained at the compensation parameter where the inverse participation ratio (IPR) is minimized ($s_{\min}=0.01$ and $s_{\min}=0.16$) for chains A and B, are respectively seen in the right panels of Figs. 15e and 15f. Only a single hot spot is observed for each chain, and the extracted values of $s_{\min}$ differ between the two chains. Using the procedure mentioned earlier, the compensated wavefunction $e^{-s_{\min}x}\psi_E(x)$ for both chains A and B never approach extended states, as shown in the bottom panels in Fig. 15c-d. Correspondingly, the IPRs to the resulting $e^{-sx}\psi_E(x)$ does not approach $1/N$. As a result, neither a reliable value of μ nor the corresponding NBFP structure can be inferred from the OBC measurements alone. By contrast, the μ predicted by the non-Bloch supercell framework is free from this limitation (Fig. 15a). Hence, extracting $\boldsymbol{\mu}$ from OBC measurements is not universally applicable, because certain classes of skin effects do not exhibit simple exponential localization.

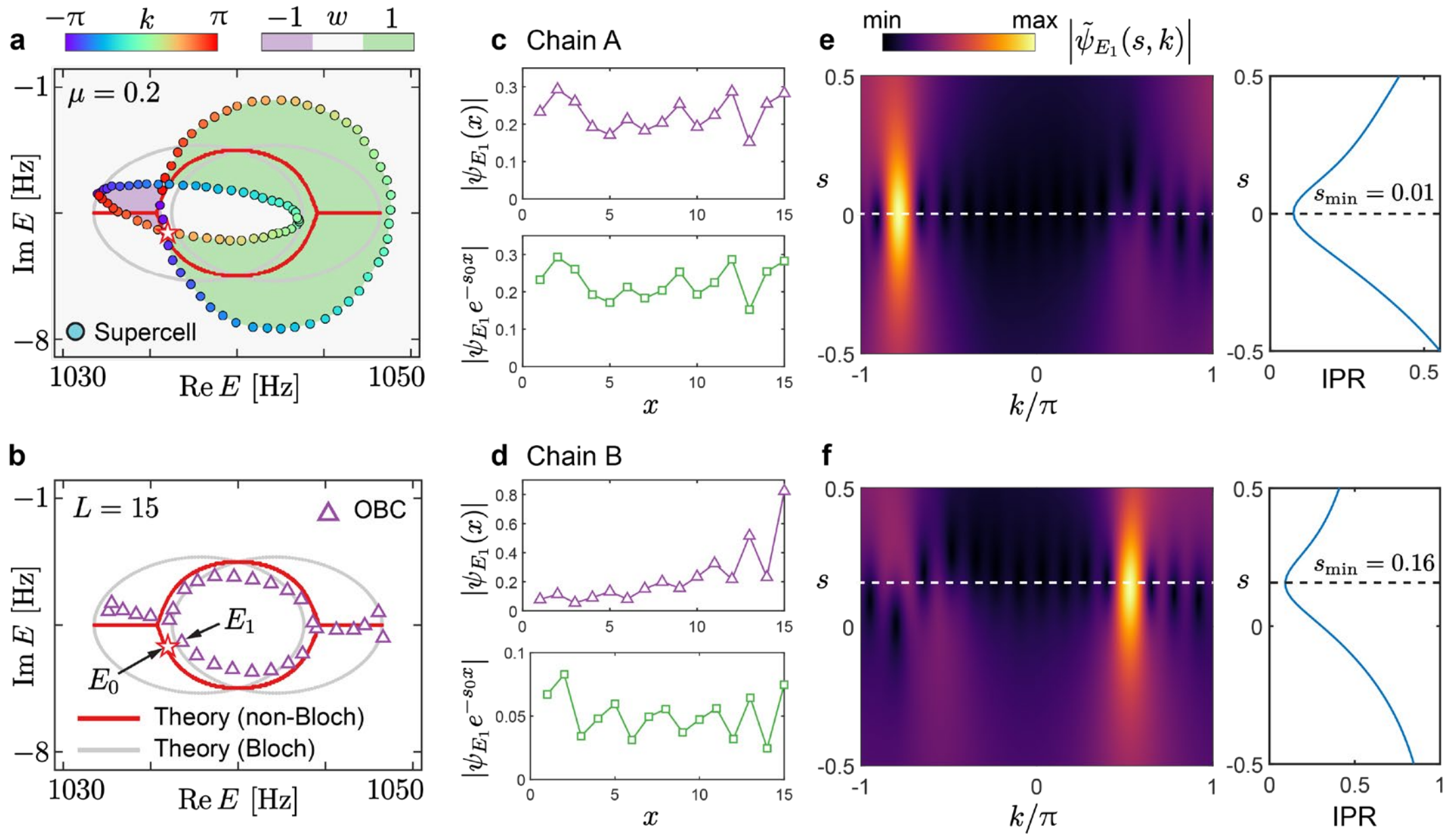

Supplementary Figure 15 | Critical skin effect: a non-exponentially localized skin mode in one dimension. a, Measured $E^{\mu}(k)$ at $\mu = 0.2$ obtained using the non-Bloch supercell framework (filled colour circles). The red star marks the self-intersection energy E_0, which resides on the OBC spectrum in the large-N limit. **b**, Measured OBC spectra (triangles) for a system size of $L = 15$. In both **a** and **b**, the grey lines indicate the theoretical PBC spectrum, and red solid lines denote the OBC spectrum in the large-N limit obtained from generalized Brillouin zone analysis. **c**,**d**, Measured real-space wavefunctions $\psi_{E_1}(x)$ (top) and the corresponding s-compensated wavefunctions $e^{-s_{\min}x}\psi_{E_1}(x)$ (bottom) for chain A (**c**) and B (**d**). The energy E_1 marked in **b** is chosen as the OBC eigenenergy closest to E_0 in **a**. **e**,**f**, s-resolved momentum-space wavefunction $\tilde{\psi}_{E_1}(s,k)$ (left) from open boundary lattices and the corresponding IPR distributions of $e^{-sx}\psi_{E_1}(x)$ (right) for chain A (**e**) and B (**f**). Here, $s_{\min}$ denotes the value of s at which the IPR reaches its minimum. The extracted values are $s_{\min} = 0.01$ and $s_{\min} = 0.16$ for chains A and B, respectively, neither of which coincides with the non-Bloch parameter predicted by the supercell framework.

More generally, similar phenomena can arise in higher-dimensional systems exhibiting non-exponentially localized skin effects. A prominent example is the algebraic skin effect[9]. As an example, we consider the case shown in Fig. 16a (corresponding to the model discussed in Fig. 7 of the main text). In contrast to exponentially localized skin modes, the IPR may develop multiple minima (Fig. 16b, left). None of these minima can fully restore the wavefunction to a conventional extended state (Fig. 16b, right), indicating that the wavefunction contains

multiple $\boldsymbol{\mu}$ components and therefore cannot be characterized by a single $\boldsymbol{\mu}$. In short, the behavior of the IPR minima for $e^{-\boldsymbol{s}\cdot\boldsymbol{r}}\psi_E(\boldsymbol{r})$ tells the localization nature of an eigenstate.

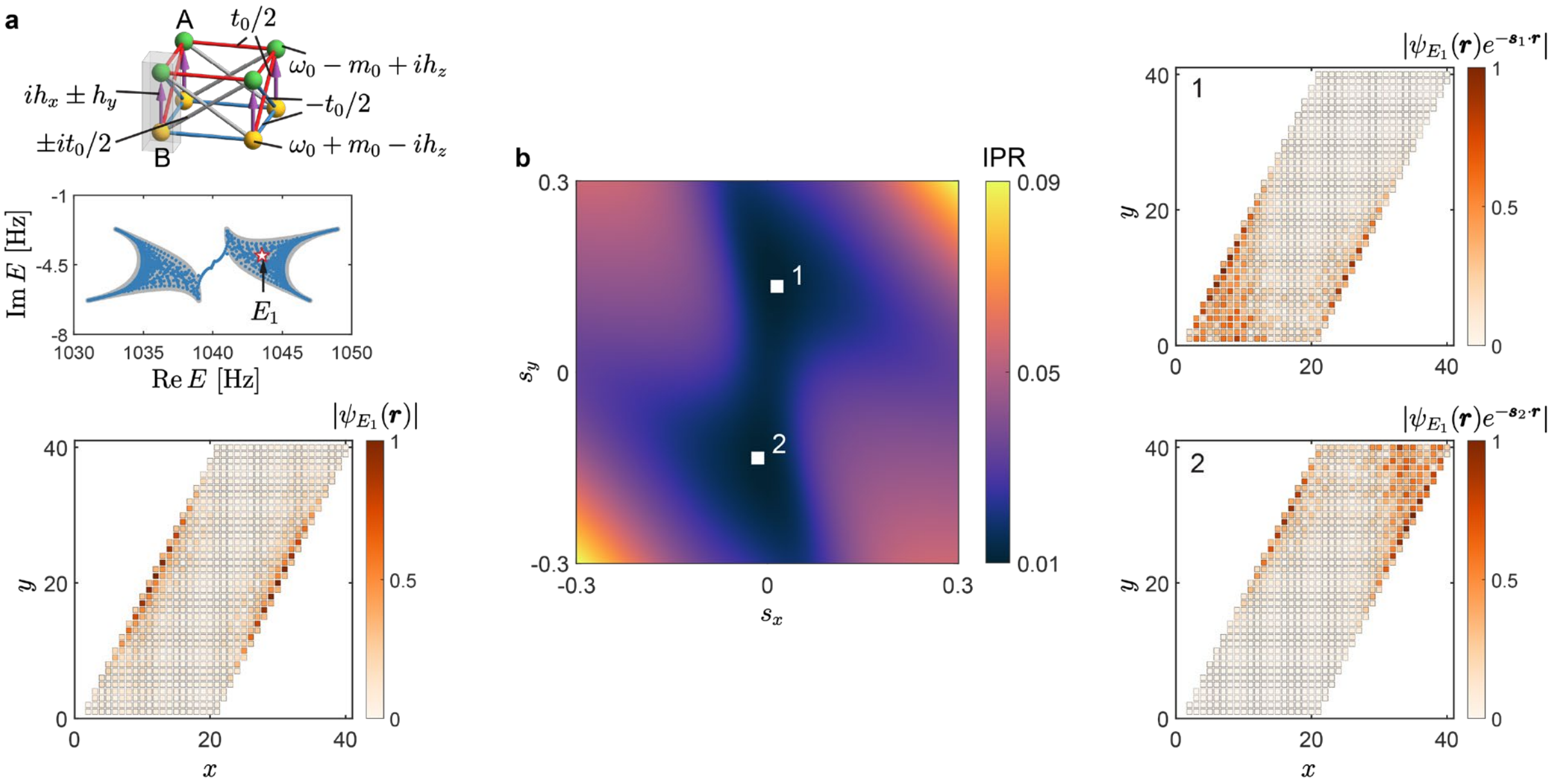

Supplementary Figure 16 | Algebraic skin effect: a non-exponentially localized skin mode in higher dimensions. a, Top: tight-binding model and the corresponding OBC spectrum. Bottom: an OBC eigenstate $|\psi_{E_1}(\boldsymbol{r})|$ of the A-sublattice with $E_1 = (1043.54 - 4.04i)$Hz, which does not exhibit simple exponential localization. **b**, Left: IPR of the compensated wavefunction $e^{-\boldsymbol{s}\cdot\boldsymbol{r}}\psi_{E_1}(\boldsymbol{r})$, showing two clearly distinguishable minima. Right: compensated wavefunctions evaluated at the two minima. In both cases, the compensation fails to restore the wavefunction to a clean extended state.

References

1. Wang, H.-Y., Song, F. & Wang, Z. Amoeba formulation of non-Bloch band theory in arbitrary dimensions. *Phys. Rev. X* **14**, 021011 (2024).
2. Hu, H. Topological origin of non-Hermitian skin effect in higher dimensions and uniform spectra. *Sci. Bull.* **70**, 51–57 (2024).
3. Yang, Z., Zhang, K., Fang, C. & Hu, J. Non-Hermitian bulk-boundary correspondence and auxiliary generalized Brillouin zone theory. *Phys. Rev. Lett.* **125**, 226402 (2020).
4. Wu, D., Xie, J., Zhou, Y., & An. J. Connections between the open-boundary spectrum and the generalized Brillouin zone in non-Hermitian systems, *Phys. Rev. B* **105**, 045422 (2022).
5. Feinberg, J. & Zee, A., Non-Hermitian random matrix theory: method of Hermitian reduction. *Nucl. Phys. B* **504**, 579 (1997).
6. Cheng, C., Shu, C., Zhang, K., Mao, X., & Sun, K. Universal spectral moment theorem and its applications in non-Hermitian systems. *Phys. Rev. Lett.* **133**, 216401 (2024).
7. Zhong, J-X., Kim, J., Chen, K., Lu, J., Ding, K.,& Jing, Y. Experimentally probing non-Hermitian spectral transition and eigenstate skewness. *Phys. Rev. B.* **112**, L220301 (2025).
8. Li, T., Duca, L., Reitter, M., Grusdt, F., Demler, E., Endres, M., Schlerier-Smith, M., Bloch, I., & Schneider, U. Bloch state tomography using Wilson lines. *Science* **352**, 6289 (2016).
9. Zhang, K., Shu, C., Zhang, & Sun, K. Algebraic Non-Hermitian Skin Effect and Generalized Fermi Surface Formula in Arbitrary Dimensions. *Phys. Rev. X.* **15**, 031039 (2025).
