# Supplementary material for: Unveiling non-Hermitian band structures with non-Bloch supercells
Source: Nat Commun. 2026 Aug 26;17:10184. doi: 10.1038/s41467-026-77152-5 (PMC13612471; doi:10.1038/s41467-026-77152-5)
Supplement: Supplementary file 2 [file 41467_2026_77152_MOESM2_ESM.pdf]

nature portfolio

Peer Review File

Unveiling non-Hermitian band structures with non-Bloch supercells

Corresponding Author: Professor yun jing

Parts of this Peer Review File have been redacted as indicated to remove third-party material.

This file contains all reviewer reports in order by version, followed by all author rebuttals in order by version.

Attachments originally included by the reviewers as part of their assessment can be found at the end of this file.

Version 1:

Reviewer comments:

Reviewer #1

(Remarks to the Author)
In the manuscript by Jia-Xin Zhong et al., the authors aim to match the energy spectrum of a non-Hermitian system with OBC to that of a non-Bloch Hamiltonian with PBC. They use the non-Bloch supercell approach to engineer an acoustic lattice with the imaginary momenta embedded in the hopping. The work seems interesting, and the methods are technically sound. However, I am not sure it is compelling enough for the general audience (see the report below).

They have a long supplementary material to support their work. However, I felt that the main text should include more details to better understand their results and experimental setup. I will list them below.

In the introduction, they mention: “Standard momentum-resolved spectroscopies, such as angle-resolved photoemission spectroscopy, inelastic neutron scattering, and photon-induced near-field electron microscopy, measure spectral functions only at real momenta, because $\boldsymbol{\mu}$ cannot be retrieved by Fourier analysis alone. The imaginary part of momentum, together with complex energies, imparts exponential decay to eigenstates, rapidly pushing observables below the experimental noise floor. The resulting irreversible information loss in spectral functions erodes the predictive power that conventional Fermi surfaces would otherwise afford.” The comparison to ARPES and other solid-state probes risks overstating the relevance to condensed-matter systems, since exponent-flattening and μ-engineering rely on programmable couplings not available in electronic materials. The authors should clarify if such a strategy is expected to be transferable to electronic materials. My main doubts about the manuscript are related to this point. I agree with the authors that the non-Bloch supercell approach is transferable to other synthetic systems, such as mechanical, circuit, microwave, and photonic platforms. However, what are we expected to learn from such an experiment beyond validating the formalism?

I did not understand what they meant by “finite dynamic range” in line 132.

They should explain how they extract energy from the Green’s function in the method section.

The manuscript would benefit from a clearer explanation in the main text of why persistence of the point-gap topology as μ varies constitutes a necessary criterion for an energy to belong to the OBC spectrum.

Is their non-Bloch supercell approach applicable in the presence of critical skin-effect as discussed in Nature Comm. 11, 5491 (2020) [https://doi.org/10.1038/s41467-020-18917-4]

The authors discuss that one can also estimate μ by taking a Fourier-Laplace transformation of the eigenstate. So, is it possible then to start with the eigenstate first and find a suitable μ from there (by analyzing the Fourier-Laplace transformation localization length)? In this case, one does not need to go through Fig. 3a-c to predict the OBC eigenvalue. Also, can one consider a Fourier-Laplace transformation of the OBC eigenstate and obtain the imaginary part of the momentum from there? If μ is known in this way, is it still necessary to engineer the synthetic lattice and resolve η with such high precision?

Reviewer #2

(Remarks to the Author)
Please find the report attached below.

Reviewer #3

(Remarks to the Author)
This manuscript presents a significant experimental advancement in the direct reconstruction of non-Hermitian band structures through a non-Bloch supercell framework. By decoupling the real and imaginary parts of momentum via exponent flattening, the authors achieve scale-free control over the complex momentum and high-resolution sampling of the Brillouin zone, overcoming a fundamental trade-off in previous approaches. The method is implemented in programmable one- and two-dimensional acoustic crystals, enabling the extraction of complex eigenenergies, eigenstates, and non-Bloch Fermi points via Green's function measurements. The experimental results are in excellent agreement with theoretical predictions and are further validated by open-boundary measurements, including the observation of boundary-activated non-Bloch Fermi points in two dimensions. The work is timely, well-executed, and conceptually clear, offering a versatile experimental platform for exploring non-Hermitian topology. I recommend publication in Nature Communications after the authors address the following comments.
First, while the manuscript focuses on a single point-gap topology, non-Hermitian systems can also exhibit many point gaps or line gaps. It would be helpful if the authors could clarify whether the proposed non-Bloch supercell framework is equally applicable to these non-Hermitian systems. A brief discussion on the generality of the method across different gap types would enhance its applicability.
Second, the current work deals with static non-Hermitian Hamiltonians. Given the growing interest in time-varying non-Hermitian systems (e.g. Phys. Rev. Lett. 135, 106601 (2025)), it is worth considering whether the proposed experimental reconstruction technique can be directly extended to such settings. A brief outlook on this direction would be valuable.
Third, the experimental platform relies on active acoustic cavities with programmable hoppings. Given that acoustic systems inherently possess material losses and parasitic dissipation, it would be instructive to comment on how such intrinsic losses might affect the precision of the extracted complex energies and eigenstates.
Overall, this work represents a major step forward in the experimental characterization of non-Hermitian band structures and will be of broad interest to the community.

Reviewer #4

(Remarks to the Author)
I co-reviewed this manuscript with one of the reviewers who provided the listed reports. This is part of the Nature Communications initiative to facilitate training in peer review and to provide appropriate recognition for Early Career Researchers who co-review manuscripts.

Version 2:

Reviewer comments:

Reviewer #1

(Remarks to the Author)
I appreciate the authors' hard work in improving the quality of the manuscript. The authors have sufficiently addressed my comments, clarified the novelty of the work in the manuscript and in their responses to all the referees, and provided additional new experimental results, especially one concerning the critical skin effect. I think the manuscript is now well-suited for publication in Nature Communications.

Reviewer #2

(Remarks to the Author)
I have carefully examined the revised manuscript and the authors' response. My main concerns from the first round have been satisfactorily addressed.

The authors now clarify the novelty relative to previous artificial-boundary and open-boundary approaches, explain more systematically the connection between twisted boundary conditions and the non-Bloch supercell construction, and provide a clearer physical interpretation of non-Bloch Fermi points. The new critical-skin-effect and non-Hermitian Chern-insulator

examples also strengthen the scope and significance of the framework. In particular, the symmetry-based standing-wave analysis strengthens the interpretation of the results on the non-Bloch Fermi-point.

Therefore, I can recommend this version of manuscript for publication in Nature Communications.

Reviewer #3

(Remarks to the Author)
The authors have satisfactorily revised the manuscript and addressed all the questions raised. I recommend acceptance for publication.

Reviewer #4

(Remarks to the Author)
I co-reviewed this manuscript with one of the reviewers who provided the listed reports. This is part of the Nature Communications initiative to facilitate training in peer review and to provide appropriate recognition for Early Career Researchers who co-review manuscripts.

Open Access This Peer Review File is licensed under a Creative Commons Attribution 4.0 International License, which permits use, sharing, adaptation, distribution and reproduction in any medium or format, as long as you give appropriate credit to the original author(s) and the source, provide a link to the Creative Commons license, and indicate if changes were made.
In cases where reviewers are anonymous, credit should be given to 'Anonymous Referee' and the source.
The images or other third party material in this Peer Review File are included in the article's Creative Commons license, unless indicated otherwise in a credit line to the material. If material is not included in the article's Creative Commons license and your intended use is not permitted by statutory regulation or exceeds the permitted use, you will need to obtain permission directly from the copyright holder.
To view a copy of this license, visit https://creativecommons.org/licenses/by/4.0/

Revisions are colored in red font. Below are the lists for major revisions.

Main text

Revision	Addressing	Page number
Revised the third paragraph of the *Introduction* to avoid overstatement and clarify the general physical issue more precisely.	Reviewer 1	2-3
Removed the word "complete" in the third paragraph of the *Introduction* and added clarifications in the *1D Non-Hermitian Band Structures, Non-Bloch Fermi Points, and Validations* section to clarify the phrase "complete reconstruction".	Reviewers 2&4	3, 7
Revised Figs. 2a-b and the related discussion in the *Non-Bloch Supercell Framework* section to present the relation between twisted boundary conditions and the non-Bloch analysis more clearly and transparently.	Reviewers 2&4	4
Added a new sentence to the fourth paragraph of the *Non-Bloch Supercell Framework* section, together with a new Methods section, to clarify the role of finite dynamic range in the experiment.	Reviewer 1	5, 20
Added a new sentence to the last paragraph of the *Non-Bloch Supercell Framework* section, together with a new Methods section, to clarify how intrinsic losses affect the experimental measurements.	Reviewer 3	6, 22
Added a new paragraph to the *1D Non-Hermitian Band Structures, Non-Bloch Fermi Points, and Validations* section to provide a clearer explanation of the point-gap criterion.	Reviewer 1	7
Added a new paragraph to the end of the *1D Non-Hermitian Band Structures, Non-Bloch Fermi Points, and Validations* section to illustrate the limitations of OBC measurements.	Reviewers 1	9
Added a new Fig. 4, together with a new paragraph at the end of the *1D Non-Hermitian Band Structures, Non-Bloch Fermi Points, and Validations* section, to highlight the necessity and capability of the non-Bloch supercell framework for accessing large-N physics, including systems with multiple point gaps.	All Reviewers	9
Revised Fig. 6c–f and added new discussions to the end of the *Boundary-Activated Non-Bloch Fermi Points in 2D Lattices* section to clarify the conceptual role of NBFPs and their relation to NBFP-compatible standing-wave interference patterns.	Reviewers 2&4	11

Added a new section entitled *Non-Bloch band reconstruction beyond the skin effect*, together with a new figure (Fig. 7) and a new Methods section, to demonstrate the generality of the framework, its necessity beyond conventional OBC measurements, and its ability to provide information beyond the skin effect, including line-gap physics.	All Reviewers	12-13, 23
Removed the word “transferable” from the first paragraph of the *Discussions and conclusions* section and revised the related discussion to delimit the applicable scope of the framework more accurately.	Reviewers 2&4	13
Added two new paragraphs to the *Discussions and conclusions* section to clarify the conceptual and technical advances of the non-Bloch supercell framework, including its relevance to systems in which hoppings are not directly programmable, to sharpen the distinction from previous works, and to highlight the role of NBFPs further.	Reviewers 1,2&4	14
Added a brief outlook discussion to the *Discussions and conclusions* section to clarify that extending the framework to Floquet non-Hermitian systems is a promising direction for future work.	Reviewer 3	15
Added a new Methods section on extracting complex eigenenergies from the Green’s function.	Reviewer 1	23
Revised the *Abstract*, the final paragraph of the *Introduction*, and the relevant parts of the *Discussions and conclusions* section accordingly to reflect the above changes consistently throughout the manuscript.	All Reviewers	All
Updated the figure numbering and added relevant references.	All Reviewers	All

Supplemental Materials

Revision	Addressing	Section #
Revised the first paragraph of Supplementary Section III to make the above statement more precise and rigorous.	Reviewers 1	Section III
Added a new section to provide a theoretical analysis of the NBFP-compatible standing waves.	Reviewers 2&4	Section VIII
Added a new section to highlight the role of NBFPs and present several examples of how NBFP signatures can be identified from OBC measurements.	Reviewers 1,2&4	Section IX

Reply to Reviewer #1

In the manuscript by Jia-Xin Zhong et al., the authors aim to match the energy spectrum of a non-Hermitian system with OBC to that of a non-Bloch Hamiltonian with PBC. They use the non-Bloch supercell approach to engineer an acoustic lattice with the imaginary momenta embedded in the hopping. The work seems interesting, and the methods are technically sound. However, I am not sure it is compelling enough for the general audience (see the report below). They have a long supplementary material to support their work. However, I felt that the main text should include more details to better understand their results and experimental setup. I will list them below.

Response:

We thank the reviewer for the positive assessment of our work as "interesting" and our methods as "technically sound." We also appreciate the concern regarding the accessibility of the manuscript to a broader audience and the need for a clearer presentation in the main text. In response, we have substantially revised the manuscript to address these concerns and improve its clarity, without relying on supplementary materials. The major revisions include:

- Expanding the discussion and definitions of several key concepts, including non-Bloch Fermi points, Green's function, finite dynamic range, and the point-gap criterion;
- Adding experimental measurements of the critical skin effect to further demonstrate the applicability and generality of the supercell framework;
- Revising the relevant discussions to clarify the necessity of the supercell framework for probing non-Hermitian band structures;
- Improving the presentation and wording throughout the main text to enhance clarity, rigor, and accessibility for a broader readership.

Detailed responses are provided below.

In the introduction, they mention: "Standard momentum-resolved spectroscopies, such as angle-resolved photoemission spectroscopy, inelastic neutron scattering, and photon-induced near-field electron microscopy, measure spectral functions only at real momenta, because mu cannot be

retrieved by Fourier analysis alone. The imaginary part of momentum, together with complex energies, imparts exponential decay to eigenstates, rapidly pushing observables below the experimental noise floor. The resulting irreversible information loss in spectral functions erodes the predictive power that conventional Fermi surfaces would otherwise afford." The comparison to ARPES and other solid-state probes risks overstating the relevance to condensed-matter systems, since exponent-flattening and μ-engineering rely on programmable couplings not available in electronic materials. The authors should clarify if such a strategy is expected to be transferable to electronic materials. My main doubts about the manuscript are related to this point. I agree with the authors that the non-Bloch supercell approach is transferable to other synthetic systems, such as mechanical, circuit, microwave, and photonic platforms. However, what are we expected to learn from such an experiment beyond validating the formalism?

Response:

We thank the reviewer for this important comment. Our original intention in referring to ARPES and related probes was merely to motivate a broader physical issue: in non-Hermitian systems, information encoded in complex momentum is generally inaccessible when experiments can probe only real momenta. Addressing this limitation is precisely one of the central motivations behind the framework developed in this work.

A key ingredient of our framework is the decoupling of the real and imaginary components of momentum through exponent flattening. This ultimately reduces the problem of probing non-Hermitian band structures to the characterization of non-Bloch Fermi points (NBFPs). Such decoupling of real and imaginary momenta, together with NBFPs, provides a useful toolkit to probe properties of non-Hermitian systems beyond the system demonstrated in this work. This treatment not only enables the measurement of non-Bloch band structures in synthetic platforms but also provides useful insights for systems in which hoppings cannot be programmatically engineered, including the electronic materials mentioned by the reviewer. Below, we further clarify these points and summarize the corresponding revisions made in the resubmitted manuscript.

1. From k-μ decoupling to non-Bloch Fermi points

One important physical insight provided by the present framework is that the k-μ decoupling recipe converts imaginary momentum from an implicit analytic continuation (Fig. R1a) into an experimentally controllable and physically interpretable variable (Fig. R1b).

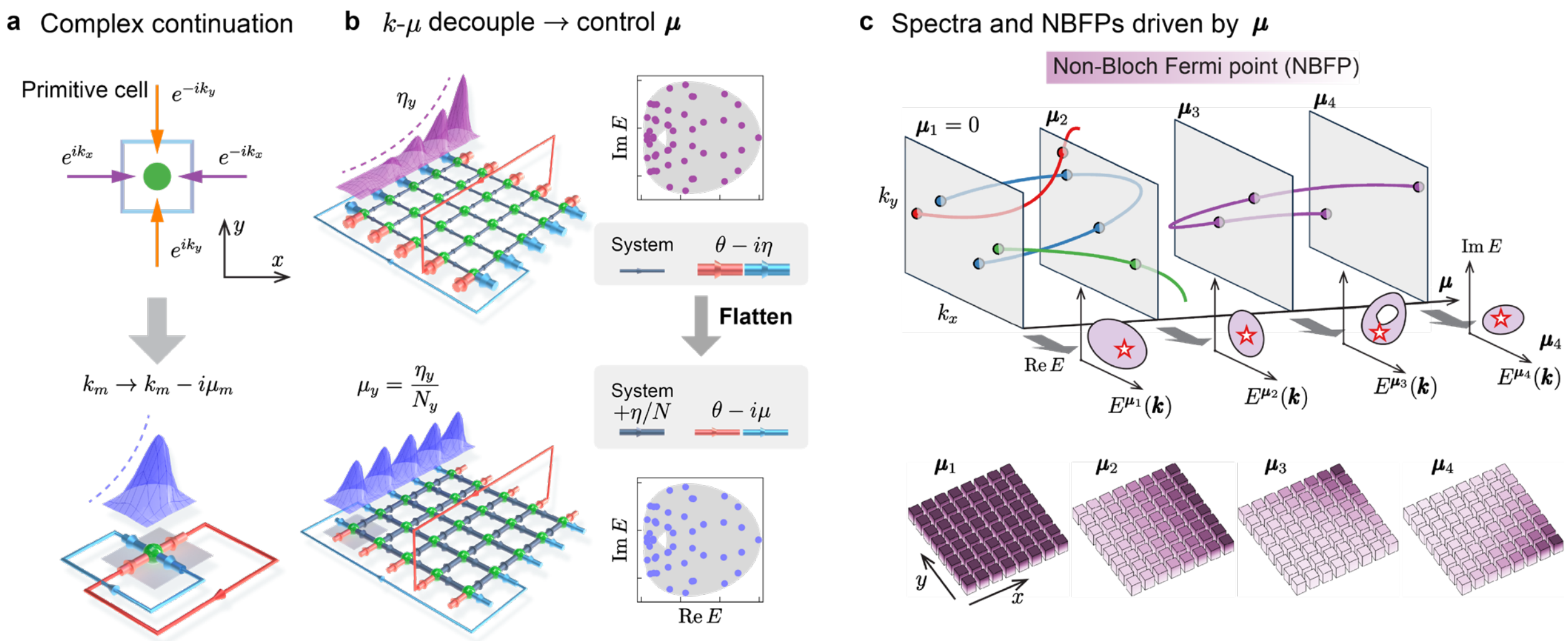

Fig. R1 | Active control of $\boldsymbol{\mu}$ and the resulting relationship between $\boldsymbol{\mu}$, spectra, and wavefunction distributions. **a**, Conventional complex-momentum continuation. **b**, k - μ decoupling in the non-Bloch supercell framework, where $\boldsymbol{\mu}$ is treated as an independently controllable parameter. **c**, Spectral deformation and the corresponding evolution of NBFPs driven by $\boldsymbol{\mu}$. Together, these determine the associated wavefunction distributions (bottom panel).

As demonstrated in Fig. R1c, this treatment allows one to directly evaluate the spectral deformation $E^{\boldsymbol{\mu}}(\boldsymbol{k})$ and the corresponding real momenta $\boldsymbol{k}$ (namely, the NBFPs) associated with a given $\boldsymbol{\mu}$. Here, $\boldsymbol{\mu}$ characterizes the decay length of the wavefunction, while the NBFPs characterize the interference properties of the waves. Together, they determine the possible wavefunction distributions that can be supported by a given OBC geometry at the considered energy. Hence, the established connection among $\boldsymbol{\mu}$, spectral deformation, and wavefunction distributions promotes NBFPs as an essential tool to characterize non-Hermitian properties.

2. Transferability to synthetic platforms with programmable hoppings

For synthetic platforms in which hoppings can be programmably engineered, a large body of work has already been devoted to band-structure measurements in Hermitian systems, including optical ring-resonator and synthetic-frequency-dimension platforms (Fig. R3a), acoustic metamaterials (Fig. R3b), electrical circuits (Fig. R3c), and related systems.

[REDACTED]

Fig. R2 | Experimental measurements of Hermitian band structures in synthetic platforms with programmable hoppings, including (**a**) optical synthetic-dimension platforms [Nature Communications 10:3122 (2019)], (**b**) acoustic platforms [Science 367,797-800 (2020)], and (**c**) electrical-circuit platforms [Nature Communications 11:4385 (2021)].

As pointed out by the reviewer, one important implication of the present work is that the physical concept of k-μ decoupling can be directly transferred to such programmable synthetic platforms. In particular, the imaginary momentum $\boldsymbol{\mu}$ can be independently controlled and encoded into the hopping parameters of the system, following the same principle demonstrated in the main text. Our framework thus enables experimental access to non-Bloch spectra and wavefunction distributions in complex momentum space, thereby extending existing band-structure measurement approaches from the Hermitian to the non-Hermitian regime.

3. Insights for systems in which the hopping cannot be programmably engineered

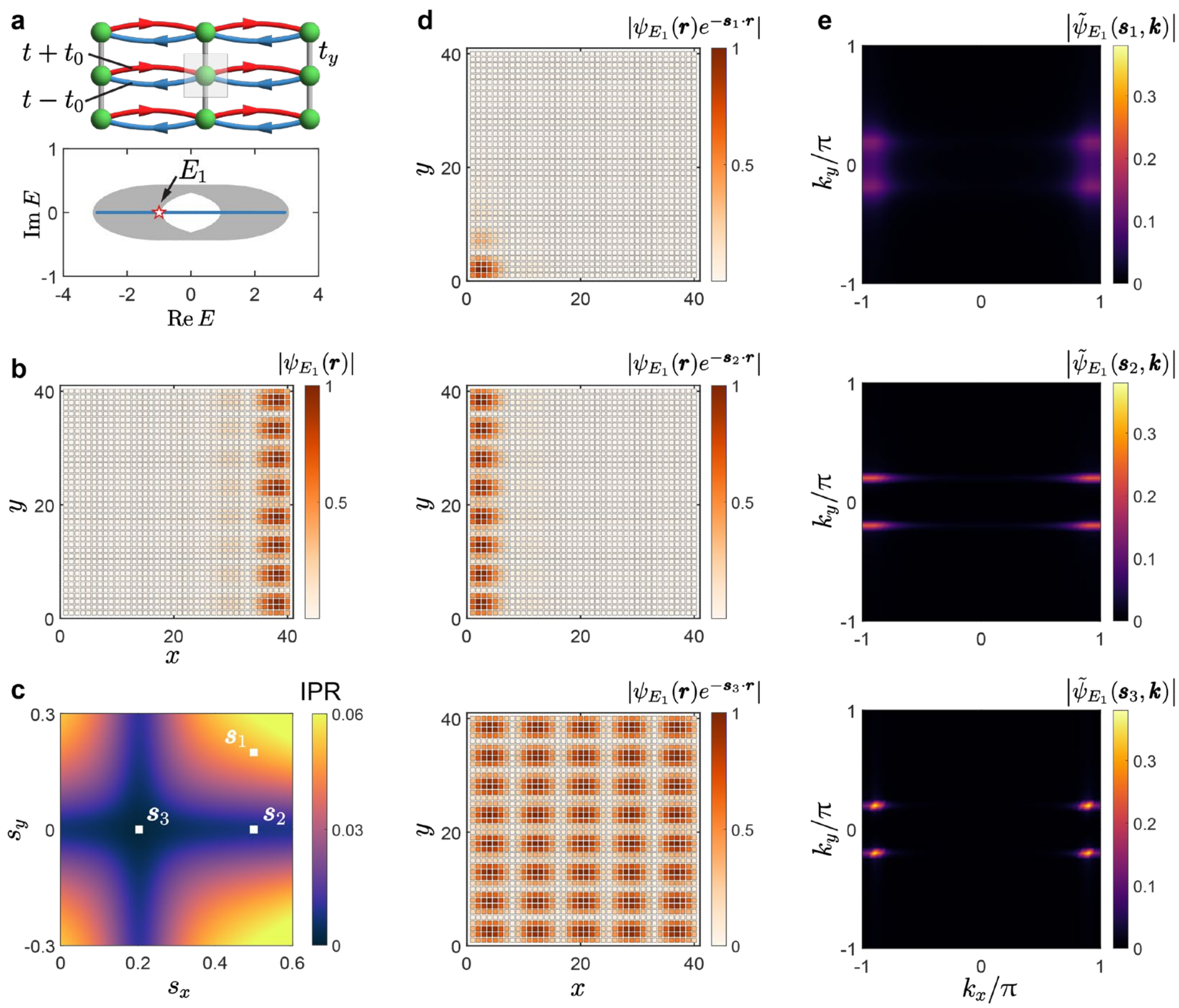

Fig. R3 | Insights for probing non-Hermitian systems without programmable hopping. a, Tight-binding model (top) and OBC spectrum (bottom). The Hamiltonian is given by $H_{\mathrm{HN}}(\boldsymbol{\beta}) = (t-t_0)\beta_x + (t+t_0)\beta_x^{-1} + t_y(\beta_y + \beta_y^{-1})$. **b**, An OBC eigenstate $|\psi_{E_1}(\boldsymbol{r})|$ with $E_1 = -1.0$, marked by the star in the top panel, which typically exhibits exponential localization toward the boundary in non-Hermitian systems. **c**, Inverse participation ratio (IPR) of the compensated wavefunction $e^{-\boldsymbol{s}\cdot\boldsymbol{r}}\psi_{E_1}(\boldsymbol{r})$. The IPR reaches its minimum at $\boldsymbol{s} = \boldsymbol{s}_3$. **d**, Wavefunctions compensated by different values of $\boldsymbol{s}$, *i.e.*, $\boldsymbol{s}_1 = (0.5, 0.2)$, $\boldsymbol{s}_2 = (0.5, 0.0)$, and $\boldsymbol{s}_3 = (0.2, 0.0)$. The imaginary-momentum compensation amplifies information that would otherwise be obscured by exponential decay. **e**, Fourier transform of the compensated wavefunction. When $\boldsymbol{s}$ reaches the optimal compensation value, the Fourier intensity is maximized, generating hot spots in momentum space corresponding to the NBFPs selected by the boundary. The parameters used are $t = 1$, $t_0 = 0.2$, and $t_y = 0.5$.

We agree that the programmable-hopping implementation demonstrated here should not be viewed as directly transferable to conventional electronic solids, although certain approaches, such as cavity-mediated couplings and strain engineering, may offer related routes. Nevertheless, the conceptual framework developed here, including both the k-μ decoupling and the resulting NBFPs, can still provide useful insights into probing non-Hermitian systems in which hopping cannot be programmably engineered.

First, the k-μ decoupling implies that we can perform $e^{-\boldsymbol{s}\cdot\boldsymbol{r}}\psi_E(\boldsymbol{r})$ to an OBC eigenstate $\psi_E(\boldsymbol{r})$ for investigating its decay behavior. Experimentally measuring eigenstates is now feasible through techniques such as Green's-function measurements [e.g., Phys. Rev. B 112, L220301 (2025)] and state tomography [e.g., Science 352, 6289 (2016)]. We therefore begin with an OBC eigenstate to illustrate our point. Figures R3a and R3b depict a staggered Hatano-Nelson model and one OBC eigenstate $\psi_{E_1}(\boldsymbol{r})$, respectively. Introducing an imaginary-momentum compensation factor $e^{-\boldsymbol{s}\cdot\boldsymbol{r}}$, we define the inverse participation ratio (IPR) to the resulting $e^{-\boldsymbol{s}\cdot\boldsymbol{r}}\psi_E(\mathbf{r})$ as

$$\mathrm{IPR} = \frac{\int |e^{-\boldsymbol{s}\cdot\boldsymbol{r}}\psi_E(\boldsymbol{r})|^4\, d\boldsymbol{r}}{\left(\int |e^{-\boldsymbol{s}\cdot\boldsymbol{r}}\psi_E(\boldsymbol{r})|^2\, d\boldsymbol{r}\right)^2}.$$

Figure R3c shows the corresponding IPR as a function of $\boldsymbol{s}$. Since smaller values of IPR correspond to more extended states, we seek values of $\boldsymbol{s}$ for which the compensated wavefunction $e^{-\boldsymbol{s}\cdot\boldsymbol{r}}\psi_E(\boldsymbol{r})$ becomes as close as possible to an extended state. For exponentially localized OBC wavefunctions, an appropriate compensation can effectively restore the state toward a clean extended profile, as illustrated in the bottom panel of Fig. R3d, where the IPR approaches $1/N$.

In contrast, for wavefunctions that are not exponentially localized—such as those exhibiting the algebraic skin effects [Phys. Rev. X 15, 031039 (2025)] (Fig. R4a)—the IPR may develop multiple minima (Fig. R4b, left). None of these minima can fully restore the wavefunction to a conventional extended state (Fig. R4b, right), indicating that the wavefunction contains multiple $\boldsymbol{\mu}$ components and therefore cannot be characterized by a single $\boldsymbol{\mu}$. In short, the behavior of the IPR minima for $e^{-\boldsymbol{s}\cdot\boldsymbol{r}}\psi_E(\boldsymbol{r})$ tells the localization nature of an eigenstate.

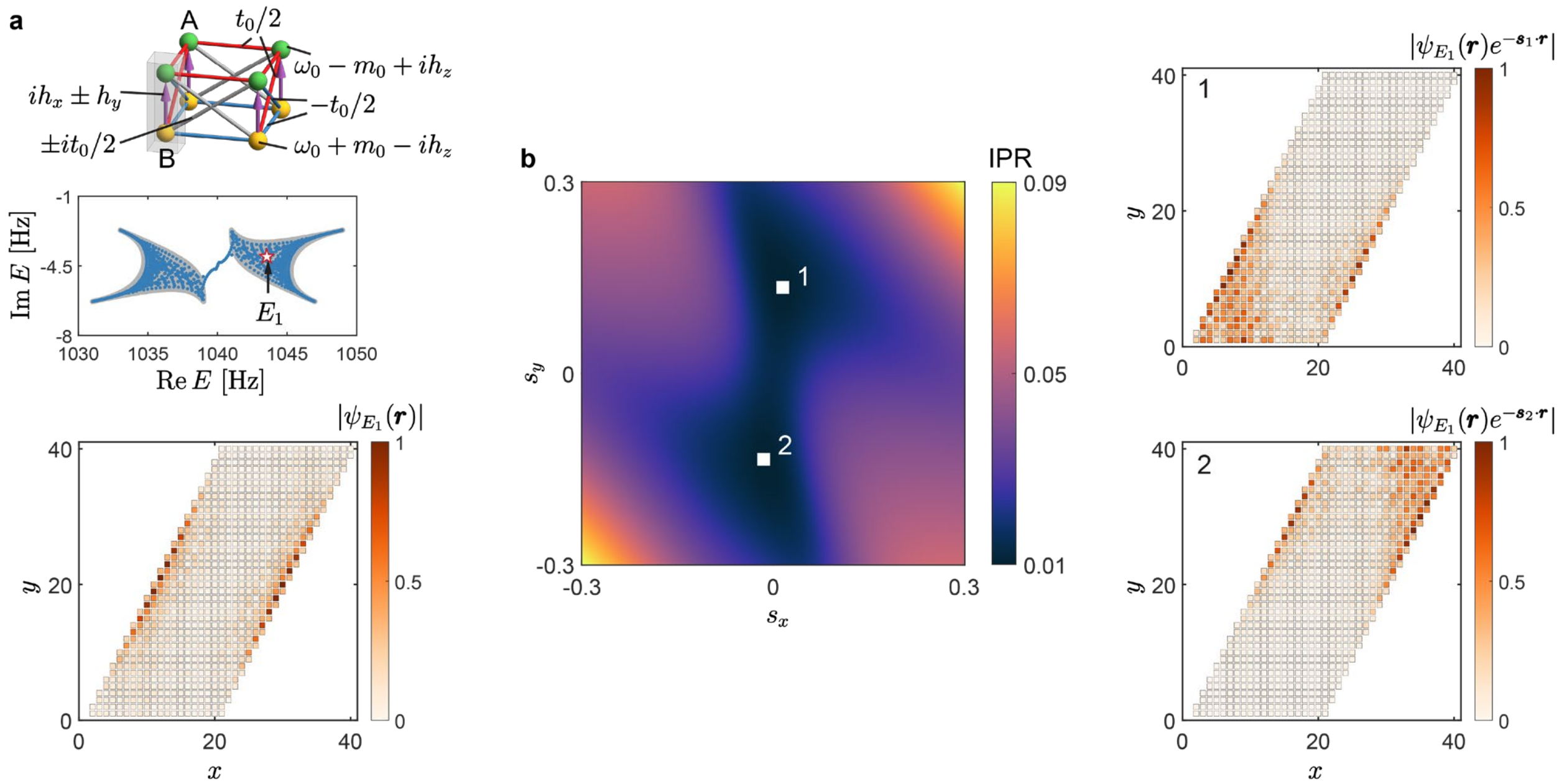

Fig. R4 | A non-exponentially localized case. a, Top: tight-binding model and the corresponding OBC spectrum. The used Hamiltonian is $H_{\mathrm{CI}}(\boldsymbol{\beta}) = \omega_0\sigma_0 + \boldsymbol{\sigma} \cdot [\boldsymbol{d}(\boldsymbol{\beta}) + i\boldsymbol{h}]$, where $\boldsymbol{d}(\boldsymbol{\beta}) = [-it_0(\beta_x - \beta_x^{-1}), -it_0(\beta_y - \beta_y^{-1}), t_0(\beta_x + \beta_x^{-1} + \beta_y + \beta_y^{-1}) - 2m_0]/2$, σ_0 is the identity matrix, $\boldsymbol{\sigma} = (\sigma_x, \sigma_y, \sigma_z)$ denotes the Pauli matrices, and $\boldsymbol{h} = (h_x, h_y, h_z) \in \mathbb{R}^3$. Bottom: an OBC eigenstate $|\psi_{E_1}(\boldsymbol{r})|$ of the A-sublattice with $E_1 = (1043.54 - 4.04i)$Hz, which does not exhibit simple exponential localization. **b**, Left: IPR of the compensated wavefunction $e^{-\boldsymbol{s}\cdot\boldsymbol{r}}\psi_{E_1}(\boldsymbol{r})$, showing two clearly distinguishable minima. Right: compensated wavefunctions evaluated at the two minima. In both cases, the compensation fails to restore the wavefunction to a clean extended state. The parameters used are $\omega_0 = (1040 - 4.5i)$ Hz, $m_0 = -1$ Hz, $t_0 = 4$ Hz, $h_x = h_y = 0$ Hz, and $h_z = 1.8$ Hz.

Second, NBFPs provide an experimentally meaningful observable for detecting complex momentum, regardless of whether the hopping parameters are programmable. Performing an s-resolved Fourier-Laplace transform on the eigenstate yields

$$\tilde{\psi}_E(\boldsymbol{s}, \boldsymbol{k}) = \int d\boldsymbol{r}\, [e^{-\boldsymbol{s}\cdot\boldsymbol{r}}\psi_E(\boldsymbol{r})]e^{-i\boldsymbol{k}\cdot\boldsymbol{r}},$$

which corresponds to Eq. (5) in the main text and is illustrated in Fig. R3e. The resulting hot spots in momentum space—namely, the Fourier peaks obtained at the compensation parameter where the IPR is minimized—correspond to the NBFPs. As demonstrated in Figs. 3 and 6 of the revised

main text, $\left|\tilde{\psi}_E(\boldsymbol{s}, \boldsymbol{k})\right|$ is maximized when the compensation parameter $\boldsymbol{s}$ matches the optimal value $\boldsymbol{\mu}_{\min}$, corresponding to the case where the exponentially localized wavefunction is most effectively restored toward an extended state. The locations of these NBFPs in the $\boldsymbol{k}$-space reflect the underlying interference structure of wavefunctions. In this way, one can investigate the imaginary part of momentum and thereby infer the full complex wavevector $\boldsymbol{k} + i\boldsymbol{\mu}$.

In brief, for electronic materials in which programmable couplings are difficult to implement, the k-μ decoupling perspective and the ensuing NBFPs offer an alternative toolkit for investigating non-Hermitian behaviors, given that eigenstates are now experimentally accessible.

To avoid overstatement and to more clearly distinguish the general physical issue from the specific experimental implementation presented in this work, we have revised the third paragraph in the *Introduction* section as follows:

"Standard momentum-resolved spectroscopies, such as angle-resolved photoemission spectroscopy[33,34], inelastic neutron scattering[35,36], and photon-induced near-field electron microscopy[37], measure spectral functions at real momenta and provide excellent tools to probe band structures and residing Fermi surfaces. However, in non-Hermitian systems, the imaginary part of the momentum, together with complex energies, imparts exponential decay to eigenstates, rapidly pushing observables below the experimental noise floor. The resulting irreversible information loss in spectral functions erodes the predictive power that conventional Fermi surfaces would otherwise afford[38,39]. Consequently, probing non-Hermitian band structures requires both (i) strategies to mitigate information loss due to exponential attenuation and (ii) the ability to actively set and vary the imaginary parts of momentum $\boldsymbol{\mu}$*. Experimental reconstruction of non-Hermitian band structures in arbitrary dimensions has therefore remained an open challenge."*

To further clarify the conceptual and technical advances of our non-Bloch supercell framework, we have added the following discussions to the *Discussions and conclusions* section of the revised manuscript:

"A key physical insight of our non-Bloch supercell framework is that k-μ *decoupling converts imaginary momentum from an implicit analytic continuation into an experimentally controllable and physically interpretable variable. Probing non-Hermitian band structures can therefore be*

reformulated in terms of NBFPs, which link the spectral deformation induced by $\boldsymbol{\mu}$ *to the spatial amplification or attenuation of eigenstates. Applying an* s*-resolved Fourier-Laplace transform to OBC eigenstates to extract NBFPs further demonstrates the necessity of imaginary-momentum compensation for recovering information lost due to non-Hermitian attenuation, providing a general strategy for recovering boundary-sensitive information in real-space non-Hermitian systems with open boundaries. This connection remains conceptually useful even in systems where hopping amplitudes cannot be directly programmed, as it provides a way to classify the relationship between spectral features and eigenstate decay behaviors within a unified non-Bloch description (Supplementary Section IX).”*

“Beyond the specific platform demonstrated here, our framework provides a transferable experimental protocol for reconstructing non-Bloch band structures, provided that the hopping amplitudes can be programmed and the full Green’s function response can be measured[30,48–51]. Since non-Bloch band theory is intended to explain non-Hermitian phenomena under OBCs, experimental reconstruction of non-Bloch band structures should be benchmarked against the corresponding OBC spectra and eigenstates. Our framework enables such a benchmark by connecting TBC-based non-Bloch band reconstruction with direct OBC spectral and eigenstate measurements, which is particularly useful because realizing genuine OBCs in synthetic dimensions is often experimentally challenging[52–55]. For platforms that naturally support OBCs, OBC measurements and probing non-Bloch band structures are equally vital, as neither alone provides all the information about non-Hermitian systems. Our framework bridges these two aspects both conceptually and technically: conceptually through k*-*μ *decoupling and NBFPs enabled by TBCs, and technically through an* N*-fold improvement in* $\boldsymbol{k}$*-space resolution over the single-cell limit without sacrificing the dynamic range of* $\boldsymbol{\mu}$*. Compared with prior experimental work on probing non-Bloch bands in synthetic dimensions[52,53] and OBC measurements[18,25,56,57], our framework enables the simultaneous investigation of non-Bloch band structures alongside OBC spectral and eigenstates across dimensions.”*

A new Supplementary Section IX has been added to highlight the role of NBFPs in a system where hoppings are not directly programmable.

I did not understand what they meant by "finite dynamic range" in line 132.

Response:

Thank you for raising this point. In the context of experiment and instrumentation, the term "dynamic range" refers to the interval between the largest and smallest signals that a measurement system can reliably resolve; the upper limit is typically set by saturation, compression, or distortion, while the lower limit is determined by the noise floor.

In our case, "finite dynamic range" means that the experimentally controllable parameter—here, the hopping amplitude—can only be varied within a finite interval. Beyond this range, the signal would either be buried in noise or enter a nonlinear or saturated regime, rendering it unreliable for resolution. The inter-supercell hopping along direction m is modulated as $t_{ij} \to t_{ij}e^{\eta_m}e^{i\theta_m}$, so the effective hopping magnitude becomes $|t_{ij}|e^{\eta_m}$. If the experimentally realizable hopping strengths are constrained to a finite interval $[\mathcal{K}_{\min}, \mathcal{K}_{\max}]$, then the accessible range of η_m is

$$\eta_m \in \left[\ln\left(\frac{\mathcal{K}_{\min}}{|t_{ij}|}\right), \ln\left(\frac{\mathcal{K}_{\max}}{|t_{ij}|}\right)\right].$$

Since $\eta_m = N_m\mu_m$, where μ_m is the imaginary part of the primitive-cell momenta along the direction m, the corresponding range of μ_m scales as

$$\mu_m \in \frac{1}{N_m}\left[\ln\left(\frac{\mathcal{K}_{\min}}{|t_{ij}|}\right), \ln\left(\frac{\mathcal{K}_{\max}}{|t_{ij}|}\right)\right],$$

which explains why the accessible range $R(\mu_m)$ decreases as N_m^{-1}.

To clarify this point, we have revised the fourth paragraph of the *Non-Bloch supercell framework* section (page 5 of the revised manuscript) as follows:

"*...the finite dynamic range of the programmable hopping amplitudes, defined as the finite interval over which the hopping magnitude can be reliably tuned before saturation or noise-floor limitations are reached, inevitably constrains the operating range R of* η_m*,* inevitably constrains the operating range R of η_m, denoted as $R(\eta_m)$, further indicating $R(\mu_m) \propto N_m^{-1}$ *(Methods)*."

The above details of dynamic range have been included as a new section titled *Finite dynamic range* in the revised Methods.

They should explain how they extract energy from the Green's function in the method section.

Response:

Thank you for your suggestion. The key ingredient is that the eigenenergies and eigenstates of Green's function $G(\omega) = (\omega - H)^{-1}$ are directly related to those of the Hamiltonian H through

$$G(\omega)\psi_v = (\omega - H)^{-1}\psi_v = \frac{1}{\omega - E_v}\psi_v,$$

where ν labels the eigenstates and $\omega \in \mathbb{R}$ is the monochromatic excitation frequency. Hence, once the full Green's function has been measured, its diagonalization yields the complex spectra E_ν and eigenstates ψ_ν of the system. The complete procedure has been detailed in our previous work [J.-X. Zhong, J. Kim, K. Chen, J. Lu, K. Ding, and Y. Jing, Experimentally probing non-Hermitian spectral transition and eigenstate skewness, Phys. Rev. B 112, L220301 (2025).]

To clarify how the complex eigenenergies are extracted from the measured Green's function, we have added the following discussion as a new section in the revised Methods part:

"***Green's-function-based measurement of eigenenergies and eigenstates***

*In the experiments, we measure the system-wide Green's functions (two-point correlators) and subsequently retrieve the complex eigenenergies and eigenstates of non-Hermitian sy*stems[17, 25, 30]*. Specifically, for a lattice of N sites, we measure the steady-state response at every probe sites* i *for each pump site* j *under monochromatic excitation at frequency ω. This yields the full response matrix* $G_{ij}(\omega)$ *over a discrete set of driving frequencies spanning the band of interest. The amplitude and phase of the response are obtained by referencing the microphone signals to the excitation signal, allowing coherent extraction of* $G_{ij}(\omega)$*. The full matrix acquisition over all pump-probe pairs yields* N^2 *complex transfer functions per frequency point.*

The Green's function satisfies $G(\omega) = (\omega - H)^{-1}$*, so the eigenvectors of* $G(\omega)$ *coincide with those of* H*, and its eigenenergies follow the single-pole form* $\lambda_\nu(\omega) = 1/(\omega - E_\nu)$ *up to a complex prefactor set by the measurement normalization. Concerning the effective tight-binding description here, we diagonalize* $G(\omega)$ *for each sampled ω, track each eigenenergy branch* $\lambda_\nu(\omega)$ *across frequency, and fit to* $\lambda_\nu(\omega) = 1/(\omega - E_\nu)$ *to extract the system eigenenergy* E_ν*. This procedure yields both Re* E_ν *and Im* E_ν*, allowing direct access to the complex spectrum. The*

biorthogonal eigenstates corresponding to E_ν are simultaneously obtained from the eigenvectors of $G(\omega)$ evaluated along the branch $\lambda_\nu(\omega)$. "

The manuscript would benefit from a clearer explanation in the main text of why persistence of the point-gap topology as μ varies constitutes a necessary criterion for an energy to belong to the OBC spectrum.

Response:

Thank you for this important and insightful comment. We agree with the reviewer that, for a given particular OBC geometry in the large-N limit, the persistence of the point-gap topology as $\boldsymbol{\mu}$ varies constitutes a necessary criterion for an energy to belong to the corresponding OBC spectrum. This important physical picture deserves a clearer explanation in the manuscript.

This statement depends on the precise definition of the OBC spectrum, which we first clarify. If the OBC spectrum, denoted by σ_{OBC}, is defined as the geometry-independent spectral envelope, *i.e.*, the union of OBC spectra over all admissible boundary geometries in the large-N limit, the persistence of the point-gap topology for all values of $\boldsymbol{\mu}$ constitutes a necessary and sufficient criterion for an energy to belong to σ_{OBC}. This result was established in Refs. [Phys. Rev. X **14**, 021011 (2024) and Sci. Bull. 70, 51–57 (2024)] and a detailed explanation is also given in Supplementary Section III. Here, we restrict our discussion to a particular OBC geometry, and thus, the persistence of the point-gap topology as $\boldsymbol{\mu}$ varies constitutes only a necessary criterion, because the spectrum of a particular geometry represents only a subset of σ_{OBC}.

To make this claim more precise and concrete, we have added a new paragraph to the *1D non-Hermitian band structures, non-Bloch Fermi points, and validations* section (second paragraph on page 7 of the revised manuscript), following the math relation, as follows:

"This criterion, referred to as the point-gap criterion, can be stated as the persistence of the point-gap topology for all values of $\boldsymbol{\mu}$, providing a necessary condition for an energy to belong to the OBC spectrum for a particular geometry in the large-N limit. We define σ_{OBC} as the geometry-independent spectral envelope, i.e., the union of OBC spectra over all admissible boundary geometries in the large-N limit, and the OBC spectrum for a particular geometry must be a subset

of σ_{OBC}. The point-gap criterion states that, for an energy E to belong to this geometry-independent envelope, the relevant point-gap topology must persist for all values of $\boldsymbol{\mu}$[41,45]. Equivalently, if there exists an admissible $\boldsymbol{\mu}$ for which the corresponding point-gap topology becomes trivial, then E fails this necessary condition and is excluded from σ_{OBC}. This criterion alone does not determine the OBC spectrum for a specific boundary geometry; additional constraints, including boundary matching and geometric details, further select the geometry-dependent subset of σ_{OBC}.

”

We have also revised the first paragraph of Supplementary Section III to make the above statement more precise and rigorous.

Is their non-Bloch supercell approach applicable in the presence of critical skin-effect as discussed in Nature Comm. 11, 5491 (2020) [https://doi.org/10.1038/s41467-020-18917-4]

Response:

Thank you for raising this important point, which allows us to clarify further the applicability and necessity of our non-Bloch supercell approach. Figure R5 presents experimental results demonstrating the application of our non-Bloch supercell framework to the critical skin effect. In brief, the non-Bloch supercell approach successfully captures non-Bloch behavior in the large-*N* limit, a regime difficult to probe with conventional OBC configurations.

We consider the model discussed in Nature Commun. 11, 5491 (2020), shown in Fig. R5a. We first apply the non-Bloch supercell method to probe its non-Hermitian band structures. The experimentally reconstructed spectra $E^{\mu}(k)$ for several representative values of μ are shown in Fig. R5b. Importantly, the self-intersections (marked as stars) of these spectral manifolds agree remarkably well with the OBC spectrum in the large-N limit. This agreement demonstrates that the supercell method directly reconstructs the non-Bloch information associated with the large-*N* limit spectrum, even though the experiment itself is performed on a finite system.

We then experimentally measure the OBC spectra of finite systems. As shown in Fig. R5c, the measured spectra exhibit the typical characteristic behavior of the critical skin effect: the OBC spectrum evolves strongly with system size and gradually approaches the red curve, which

corresponds to the OBC spectrum in the large-N limit obtained from the generalized Brillouin zone analysis.

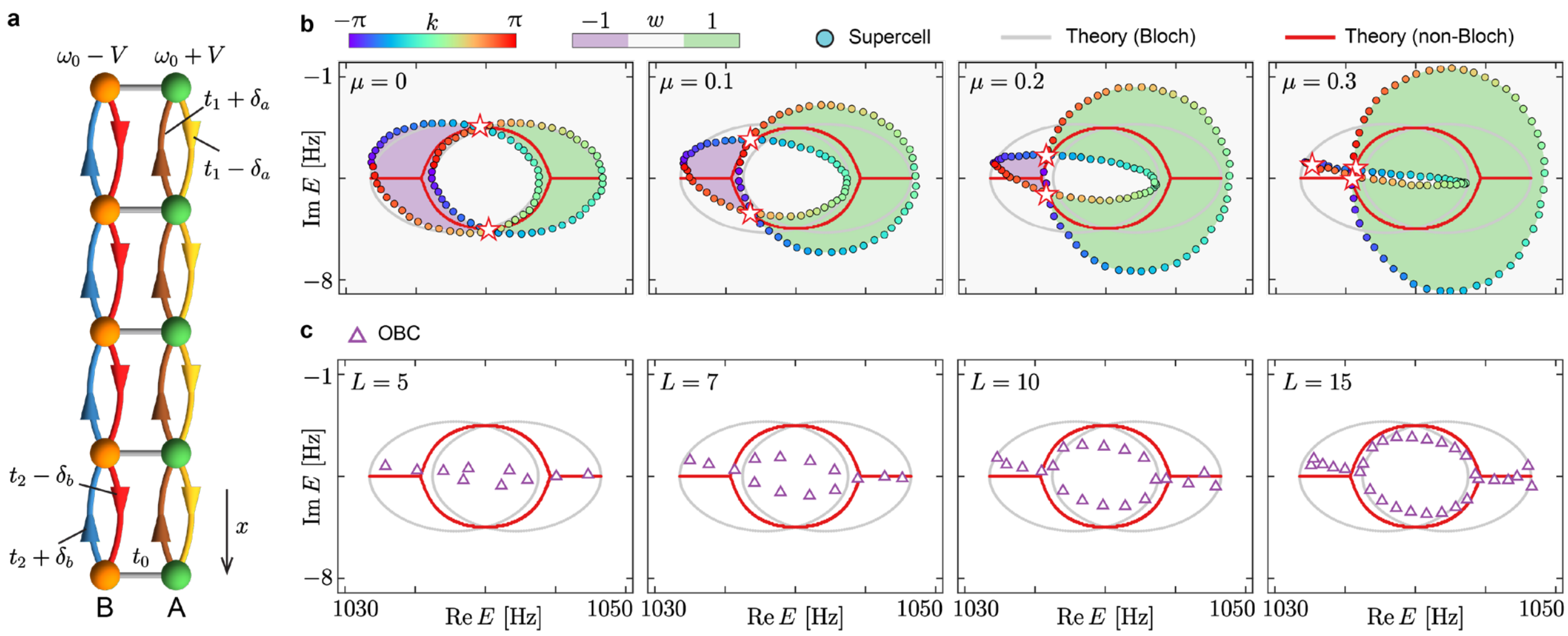

Fig. R5 | Distinction between the non-Bloch supercell framework and OBC measurements of the critical skin effect. a, Two-chain model supporting the critical skin effect. The lattice model is $H_{\text{CSE}}(\beta) = \begin{pmatrix} g_A(\beta) & t_0 \\ t_0 & g_B(\beta) \end{pmatrix}$ with $g_A(\beta) = \omega_0 + t_A^+\beta + t_A^-\beta^{-1} + V$ and $g_B(\beta) = \omega_0 + t_B^+\beta + t_B^-\beta^{-1} - V$. The two chains are labeled as A and B. **b**, Measured $E^\mu(k)$ for four selected values of μ. Filled circles with their colour encoding k are from the non-Bloch supercell framework, and their self-intersections (marked as stars) indicate the OBC spectrum in the large-N limit, as highlighted by the red stars. **c**, Measured OBC spectra (triangles) for increasing system sizes L (left to right). For comparison, the measured PBC spectra are shown as circles. In both **b** and **c**, the grey lines indicate the theoretical PBC spectrum, and the red solid lines are the OBC spectrum in the large-N limit obtained from the generalized Brillouin zone analysis. The red stars in **b** are in excellent agreement with these generalized Brillouin zone analyses, validating the non-Bloch supercell framework. By contrast, the OBC spectra in **c** deviate from the large-N limit, revealing the strong finite-size dependence characteristic of the critical skin effect. The parameters used are $\omega_0 = (1040 - 4.5i)$ Hz, $t_0 = 1$ Hz, $t_1 = 3$ Hz, $\delta_A = 1$ Hz, $\delta_B = -1$ Hz, and $V = 2$ Hz. The supercell is composed of $N_x = 5$ unit cells.

We believe this example is particularly important because it directly demonstrates one key aspect of the supercell framework's physical necessity. In size-sensitive non-Hermitian systems

such as the critical-skin case, where the spectrum evolves with system size, measurements performed on any finite system cannot capture the full information in the large-N limit. The non-Bloch supercell framework overcomes this limitation by providing direct access to non-Bloch information beyond that available from conventional finite-size OBC spectroscopy.

To illustrate the capability of our approach, we have added Fig. R5 (now Fig. 4 in the revised manuscript), together with the following discussion, at the end of the *1D non-Hermitian band structures, non-Bloch Fermi points, and validations* section (third paragraph on page 9 of the revised manuscript) in the revised main text:

"More importantly, beyond measurement instability, OBC-based approaches are not universally reliable for probing non-Hermitian band structures, particularly in systems with significant finite-size effects, such as those exhibiting the critical skin effect (Fig. 4). Figure 4a shows a representative multiband model supporting the critical skin effect and multiple point gaps[46]. Our framework successfully captures the non-Bloch behavior in the large-N limit (Fig. 4b), which is difficult to access using conventional OBC measurements (Fig. 4c). This demonstrates that the non-Bloch supercell framework overcomes intrinsic limitations of OBC-based approaches and enables direct access to non-Bloch information in the large-N limit."

The authors discuss that one can also estimate μ by taking a Fourier-Laplace transformation of the eigenstate. So, is it possible then to start with the eigenstate first and find a suitable μ from there (by analyzing the Fourier-Laplace transformation localization length)? In this case, one does not need to go through Fig. 3a-c to predict the OBC eigenvalue. Also, can one consider a Fourier-Laplace transformation of the OBC eigenstate and obtain the imaginary part of the momentum from there? If μ is known in this way, is it still necessary to engineer the synthetic lattice and resolve η with such high precision?

Response:

Thank you for this insightful question. In principle, one can indeed start from an open-boundary eigenstate and estimate an effective $\boldsymbol{\mu}$ by analyzing its Fourier-Laplace transform, since $\boldsymbol{\mu}$ is encoded in the localization length. (The triangles in Fig. 3e were obtained using this approach.)

We agree that this provides a useful alternative perspective and may improve the efficiency of identifying the relevant $\boldsymbol{\mu}$.

However, extracting $\boldsymbol{\mu}$ directly from OBC eigenstates is not a universal strategy, due to (i) critical cases with size-dependent/ultra-sensitive OBC spectra and (ii) information loss induced by exponential attenuation in large systems. By contrast, the non-Bloch supercell framework provides information beyond that associated with a particular OBC realization by directly reconstructing the full non-Bloch band dispersion in complex momentum space, together with related topological properties such as point-gap topology and Berry curvature. We further clarify these points below.

1. Limitations of extracting $\boldsymbol{\mu}$ from OBC measurements

This approach has two main limitations. First, there are critical or sensitive cases in which the OBC spectral properties depend strongly on system size or other geometric details. As demonstrated by the critical-skin experiment (Fig. R6), one cannot reliably extract μ from finite-size OBC eigenstates $\psi_E(x)$ for chain A and chain B as shown in the top panels of Fig. R6c-d. The s-resolved Fourier-Laplace transform of the eigenstates on chains A and B is shown in the left panels of Figs. R6e and R6f, respectively. The resulting hot spots in momentum space, namely, the Fourier peaks obtained at the compensation parameter where the inverse participation ratio (IPR) is minimized ($s_{\min} = 0.01$ and $s_{\min} = 0.16$) for chains A and B, are respectively seen in the right panels of Figs. R6e and R6f. Only a single hot spot is observed for each chain, and the extracted values of $s_{\min}$ differ between the two chains.

Using the procedure mentioned earlier, the compensated wavefunction $e^{-s_{\min}x}\psi_E(x)$ for both chains A and B never approach extended states, as shown in the bottom panels in Fig. R6c-d. Correspondingly, the IPRs to the resulting $e^{-sx}\psi_E(x)$ does not approach $1/N$. As a result, neither a reliable value of μ nor the corresponding NBFP structure can be inferred from the OBC measurements alone. By contrast, the μ predicted by the non-Bloch supercell framework is free from this limitation (Fig. R6a).

Hence, extracting $\boldsymbol{\mu}$ from OBC measurements is not universally applicable because certain classes of skin effects do not exhibit simple exponential localization. In contrast, our k-μ decoupling perspective offers an alternative tool for distinguishing different classes of skin effects.

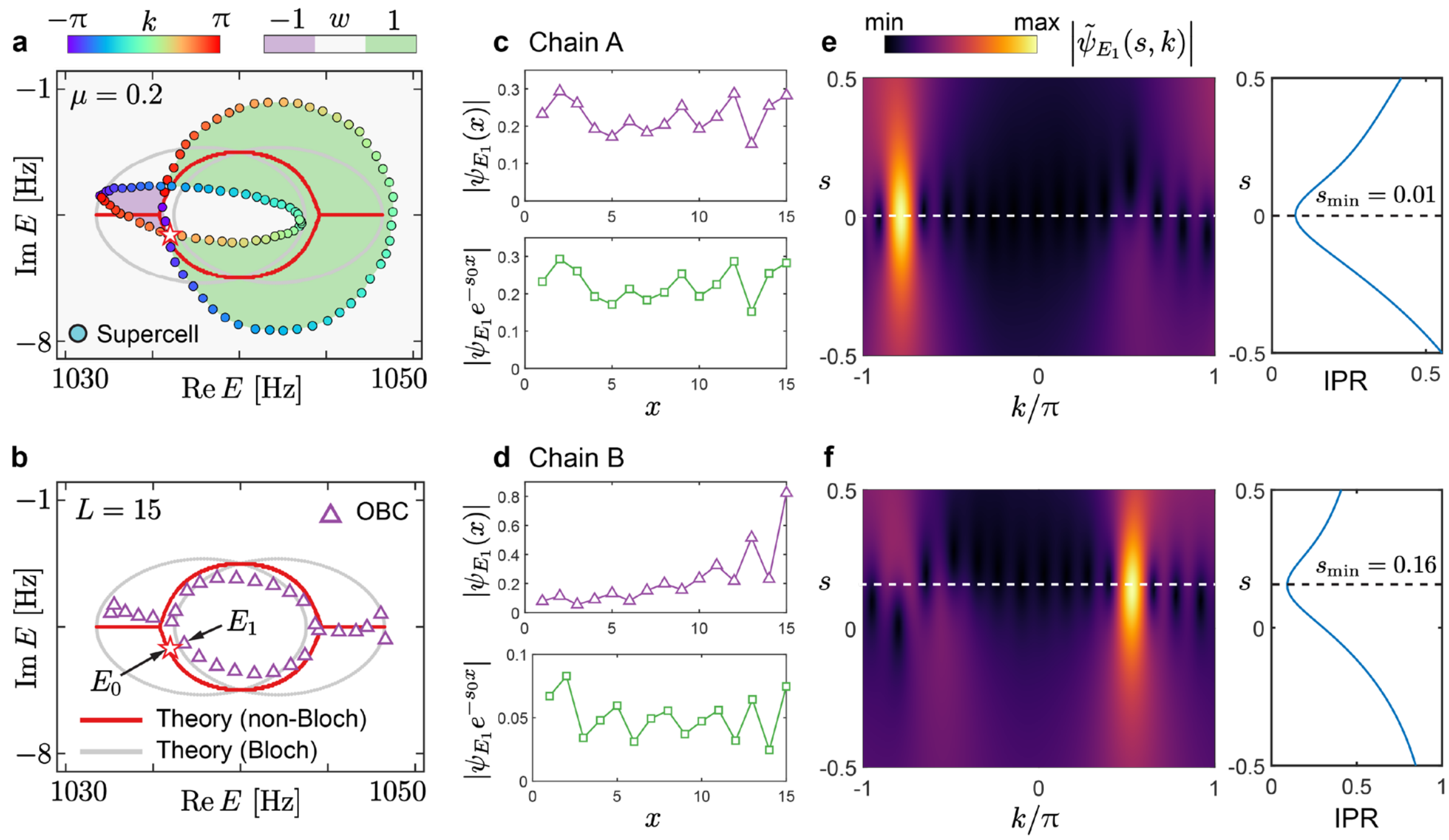

Fig. R6 | Extracting μ from OBC measurements in the presence of critical skin effect. **a**, Measured $E^{\mu}(k)$ at $\mu = 0.2$ obtained using the non-Bloch supercell framework (filled colour circles). The red star marks the self-intersection energy E_0, which resides on the OBC spectrum in the large-N limit. **b**, Measured OBC spectra (triangles) for a system size of $L = 15$. In both **a** and **b**, the grey lines indicate the theoretical PBC spectrum, and red solid lines denote the OBC spectrum in the large-N limit obtained from generalized Brillouin zone analysis. **c**,**d**, Measured real-space wavefunctions $\psi_{E_1}(x)$ (top) and the corresponding s-compensated wavefunctions $e^{-s_{\min}x}\psi_{E_1}(x)$ (bottom) for chain A (**c**) and B (**d**). The energy E_1 marked in **b** is chosen as the OBC eigenenergy closest to E_0 in **a**. **e**,**f**, s-resolved momentum-space wavefunction $\tilde{\psi}_{E_1}(s,k)$ (left) from open boundary lattices and the corresponding IPR distributions of $e^{-sx}\psi_{E_1}(x)$ (right) for chain A (**e**) and B (**f**). Here, $s_{\min}$ denotes the value of s at which the IPR reaches its minimum. The extracted values are $s_{\min} = 0.01$ and $s_{\min} = 0.16$ for chains A and B, respectively, neither of which coincides with the non-Bloch parameter predicted by the supercell framework.

Second, experimental probing of OBC eigenstates is intrinsically affected by the information loss induced by exponential attenuation, a limitation that becomes increasingly severe as the system size grows. As shown in Fig. R7, the $\beta = e^{\mu}e^{ik}$ values extracted from OBC eigenstates (triangles) exhibit noticeable deviations from the analytical solutions (solid lines), particularly near

saddle-point regions where the system becomes highly sensitive to perturbations. In contrast, our non-Bloch supercell measurements (filled colour circles) remain substantially more robust due to the μ-flattening mechanism.

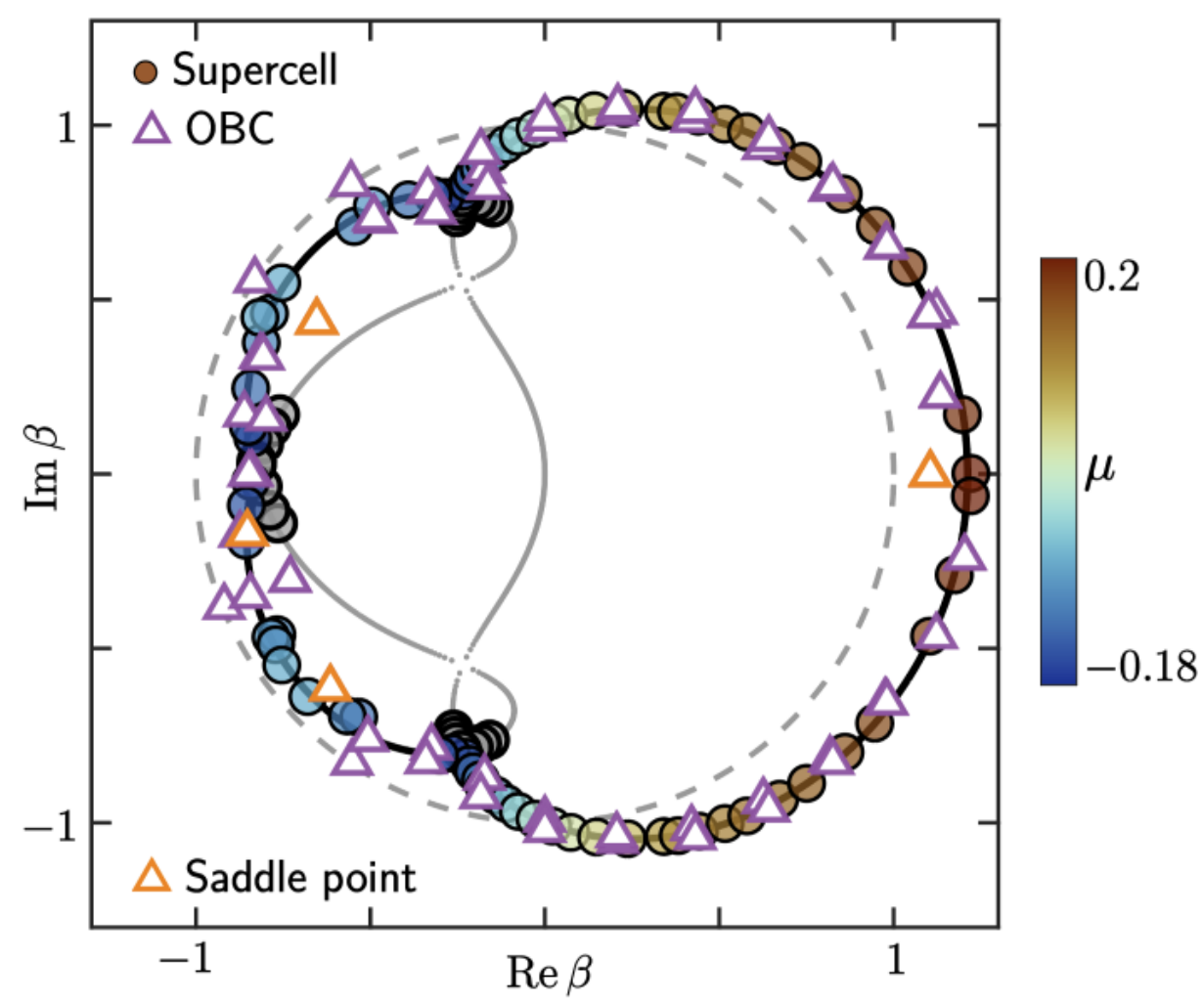

Fig. R7 | μ-instability of OBC measurements compared with the non-Bloch supercell method. Generalized Brillouin zones determined from non-Bloch supercells (circles) and measured in a finite OBC lattice (triangles). The models and parameters are the same as in Fig. 3 of the main text.

2. Generality of the non-Bloch supercell framework: beyond the estimation of μ

The procedure shown in Fig. 3a-c, namely sweeping over all admissible μ, provides a more general and robust approach compared to the OBC-based measurement. Rather than relying on a posteriori analysis of OBC eigenstates, the non-Bloch supercell protocol reconstructs the spectrum directly and identifies the corresponding non-Bloch Fermi points in a controlled and systematic manner.

More importantly, the supercell framework provides information beyond the estimation of $\boldsymbol{\mu}$. The reconstructed non-Bloch band structures contain not only the full complex-energy dispersion but also the associated eigenstate and topological information, including Berry curvature and point-gap topology. We have utilized a non-Hermitian Chern insulator to showcase such capabilities in the revised main text (Fig. 7 and a new section *Non-Bloch band reconstruction beyond the skin effect*).

To illustrate the limitations of OBC measurements in extracting $\boldsymbol{\mu}$ and to highlight the generality of our non-Bloch supercell framework, we have added the following paragraphs at the end of the *1D non-Hermitian band structures, non-Bloch Fermi points, and validations* section (page 9 of the revised manuscript):

"As demonstrated in Figs. 3e and 3f, one can perform a Fourier-Laplace transformation of the OBC eigenstate to extract μ and the corresponding NBFPs (Fig. 3e, triangles). However, OBC measurements are themselves subject to the intrinsic boundary sensitivity of non-Hermitian systems. Because imaginary momenta induce exponential attenuation, the signal can rapidly fall below the experimental noise floor, leading to increasing information loss as the system size grows. For example, in Fig. 3e, the values extracted from OBC eigenstates (triangles) deviate from the analytical solutions (solid lines), particularly near saddle-point regions where the system becomes highly sensitive. In contrast, our non-Bloch supercell measurements (circles) remain robust due to the μ-flattening mechanism.

More importantly, beyond measurement instability, OBC-based approaches are not universally reliable for probing non-Hermitian band structures, particularly in systems with significant finite-size effects, such as those exhibiting the critical skin effect (Fig. 4). Figure 4a shows a representative multiband model supporting the critical skin effect and multiple point gaps[46]. Our framework successfully captures the non-Bloch behavior in the large-N limit (Fig. 4b), which is difficult to access using conventional OBC measurements (Fig. 4c). This demonstrates that the non-Bloch supercell framework overcomes intrinsic limitations of OBC-based approaches and enables direct access to non-Bloch information in the large-N limit."

We have also added a new Supplementary Section IX that offers a holistic understanding of different classes of skin effects from our k-μ decoupling perspective. To highlight that our non-Bloch supercell measurement provides information beyond the estimation of $\boldsymbol{\mu}$, we have also added a new section entitled *Non-Bloch band reconstruction beyond the skin effect*, together with a new figure (Fig. 7) in the revised main text.

Reply to Reviewer #2 & Reviewer #4

This manuscript presents a non-Bloch supercell framework for the reconstruction of non-Hermitian band structures and its implementation in programmable one- and two-dimensional acoustic lattices. The work is technically sophisticated, the experimental platform is impressive, and the overall agreement between theory and experiment appears convincing. The introduction of non-Bloch Fermi points (NBFPs) is also well motivated, and the manuscript makes a plausible case that they organize changes in directional winding and connect to open-boundary spectral features.

Notwithstanding these strengths, I am not persuaded that the manuscript, in its present form, makes a sufficiently compelling case for publication in Nature Communications. My concerns pertain primarily to conceptual positioning and scope rather than to the technical execution itself.

Response:

We sincerely thank the reviewers for the highly positive assessment of our work and for their thoughtful, constructive comments. We particularly appreciate the reviewers' recognition of the technical sophistication of the framework, the experimental implementation, and the agreement between theory and experiment.

We also appreciate the reviewers' concern that the broader conceptual positioning and physical scope of the work were not yet sufficiently clear in the original manuscript. In response, we have revised the relevant sections of both the main text and the Supplementary Materials to highlight the following points:

- Complex-continuation twisted boundary conditions bridge PBC/non-Bloch and OBC analyses, while decoupling the real and imaginary momentum components yields a non-Bloch supercell framework for the controlled probing of non-Hermitian band structures.
- NBFPs, central to our framework, contain essential information about skin modes, including spectral density, geometry-selected standing-wave pairs, etc, providing a useful indicator for distinguishing different classes of skin effects, even when only OBC measurements are available.

- The established framework provides an experimental approach, applicable across a variety of platforms with engineered couplings, for investigating OBC spectra and eigenstates in the large-N limit, which are otherwise inaccessible via conventional OBC measurements.
- To demonstrate the generality of the framework, including its applicability to systems without skin effects, we have applied our method to a non-Hermitian Chern insulator and experimentally reconstructed its two-dimensional band structure and Berry curvature distribution.

Below, we address the reviewers' comments in detail.

Major Comments

1 · The novelty with respect to prior experimental work should be articulated more precisely. The manuscript presents the proposed framework as a general route toward reconstructing non-Hermitian band structures. At the same time, it is stated that the approach recapitulates earlier artificial-boundary protocols in the single-cell limit. This considerably weakens the novelty claim unless the authors explain in a more explicit and precise manner what is genuinely new in the present work relative to existing synthetic-boundary, point-gap, and related non-Hermitian band-diagnostic approaches. In its current form, the distinction is not sufficiently sharp for a journal of this scope.

Response:

Thank you for this important comment. We appreciate the reviewers' suggestion to sharpen the distinction between the present work and previous synthetic-boundary and related non-Hermitian band-diagnostic experimental approaches. Experimentally probing non-Hermitian bands is quite recent and has focused on the 1D scenario to reconstruct non-Bloch bands (Sci. Adv. 12, eaec8239 (2026); arXiv:2511.06844). Besides dimensionality, they limit themselves to non-Bloch bands and lack experimental OBC verification. The novelty of our approach is that it provides a direct bridge between PBC/non-Bloch and OBC analyses, both experimentally and conceptually, within a unified framework that is independent of dimension and lattice type. In our reply to this comment, we will focus on the experimental side and present conceptual insights later.

1. Experimental necessity of jointly resolving non-Bloch band structures and OBC signatures

[REDACTED]

Fig. R8 | Comparison between the non-Bloch supercell framework and previous experimental approaches. **a**, Experimental implementations based on synthetic dimensions in one-dimensional photonic systems [e.g., *Sci. Adv.* **12**, eaec8239 (2026) and arXiv: 2511.06844]. **b**, Schematic illustration of the non-Bloch supercell framework. The top and bottom panels respectively depict the configuration of complex-continuation twisted boundary conditions and our non-Bloch supercell framework. **c**, Open-boundary measurements in real-space lattices [e.g., Nat. Commun. 12, 5377 (2021) and Phys. Rev. B 112, L220301 (2025)]. The approaches shown in (**a**) correspond to the $N = 1$ limit of our framework in terms of non-Bloch bands. Replacing complex-continuation twisted boundary conditions in (**b**) with OBCs yields the configurations shown in (**c**).

Since non-Bloch band theory is primarily intended to explain non-Hermitian phenomena under OBCs, any experimental reconstruction of non-Bloch band structures should ultimately be benchmarked against the corresponding OBC spectra and eigenstates. Previous experimental work can be broadly divided into two major classes: purely OBC-based measurements (Fig. R8c) and single-cell measurements employing artificial-boundary protocols (Fig. R8a). Purely OBC-based measurements certainly do not, by themselves, provide complete information about the non-Bloch band structures. In contrast, artificial-boundary protocols are typically implemented in synthetic

dimensions, making direct experimental benchmarking against genuine OBC systems challenging. Therefore, OBC measurements and non-Bloch band reconstruction are equally essential, as neither alone provides a complete characterization of non-Hermitian systems. Establishing a direct connection between them is precisely the role of the framework developed in this work.

The connection between PBC/non-Bloch and OBC analyses theoretically is provided by the non-Bloch bulk-boundary correspondence, in which the real and imaginary components of momentum are intrinsically linked. As a result, OBC measurements and non-Bloch band measurements generally correspond to distinct experimental configurations. We believe this is why previous experimental studies have focused on either one (Figs. R8a and R8c). As noted above, establishing a unified framework that connects the two is essential. We recognize that complex-continuation twisted boundary conditions (Fig. R8b, top) serve as a bridge between non-Bloch and OBC analyses, thereby enabling both to be accessed within a single supercell-based experimental framework.

2. Experimental challenge in independently controlling complex-momentum components

The supercell approach maintains access to real components of momentum, while restricting experimental access to the imaginary components due to the exponential attenuation problem in real-space non-Hermitian lattices (as detailed in Fig. 2a-b in the main text). Once systems with genuine spatial extent and higher dimensionality are considered, the attenuation induced by non-Hermiticity rapidly becomes a major experimental bottleneck. Consequently, the experimentally accessible dynamic range of $\boldsymbol{\mu}$ shrinks substantially as the system size and dimensionality increase (Fig. R8b, top-right panel).

To overcome this limitation, we propose the k-μ decoupling strategy, which transforms $\boldsymbol{\mu}$ into inter-cell hoppings without sacrificing its accessible dynamic range (Fig. R8b, bottom panel). Experimentally, this requires the capability of programmable hopping, which constitutes one of the key technical achievements of the present work. Together with Green's function measurements, our non-Bloch supercell approach enables the direct reconstruction of non-Bloch band structures across the dynamic range of $\boldsymbol{\mu}$. Regarding non-Bloch bands from supercells, our framework naturally recovers the single-cell limit ($N = 1$). This does not diminish the novelty of our approach, since the single-cell limit lacks OBC functionality. In short, compared with prior experimental

work, our non-Bloch supercell framework enables the simultaneous investigation of non-Bloch band structures alongside OBC spectral and eigenstates across dimensions.

To sharpen the novelty of our approach and distinguish it from previous experimental works, we have added the following paragraph to the *Discussions and conclusions* section (third paragraph on page 14) in the revised main text:

"Beyond the specific platform demonstrated here, our framework provides a transferable experimental protocol for reconstructing non-Bloch band structures, provided that the hopping amplitudes can be programmed and the full Green's function response can be measured[30,48–51]*. Since non-Bloch band theory is intended to explain non-Hermitian phenomena under OBCs, experimental reconstruction of non-Bloch band structures should be benchmarked against the corresponding OBC spectra and eigenstates. Our framework enables such a benchmark by connecting TBC-based non-Bloch band reconstruction with direct OBC spectral and eigenstate measurements, which is particularly useful because realizing genuine OBCs in synthetic dimensions is often experimentally challenging*[52–55]*. For platforms that naturally support OBCs, OBC measurements and probing non-Bloch band structures are equally vital, as neither alone provides all the information about non-Hermitian systems. Our framework bridges these two aspects both conceptually and technically: conceptually through* k-μ *decoupling and NBFPs enabled by TBCs, and technically through an* N*-fold improvement in* $\boldsymbol{k}$*-space resolution over the single-cell limit without sacrificing the dynamic range of* $\boldsymbol{\mu}$*. Compared with prior experimental work on probing non-Bloch bands in synthetic dimensions*[52,53] *and OBC measurements*[18,25,56,57]*, our framework enables the simultaneous investigation of non-Bloch band structures alongside OBC spectral and eigenstates across dimensions."*

2. The conceptual role of NBFPs remains insufficiently established.

NBFPs constitute an appealing and potentially useful construction, and the manuscript suggests that they organize discontinuous changes in directional winding. However, it remains unclear whether NBFPs should be regarded as a substantive conceptual advance in non-Hermitian band theory or primarily as an experimentally convenient reformulation of already established non-Bloch ideas. The present manuscript does not yet make this distinction sufficiently clear.

Response:

Thank you for recognizing that NBFPs constitute an appealing and potentially useful concept. We appreciate this important question, which has helped us clarify the conceptual role of NBFPs more explicitly in the revised manuscript. Within our framework, NBFPs serve as counterparts to Fermi points in non-Hermitian systems, but their significance extends well beyond the characterization of directional winding numbers. They provide a unified description of spectral structures at fixed decay length, the corresponding spatial wavefunction profiles, the connection between the two, and the resulting OBC signatures. Below, we elaborate on these points in detail.

1. NBFP density: spectral structure of fixed-decay-length wavefunctions

From the definition, NBFPs correspond to the set of real momenta satisfying

$$K_{\boldsymbol{\mu}}(E_b) = \{k \in \mathrm{BZ} \mid \det[E_b - H_{\boldsymbol{\mu}}(\boldsymbol{k})] = 0\},$$

for a given energy E_b and imaginary momentum $\boldsymbol{\mu}$, as introduced in Eq. (1) of the main text. Unlike Hermitian Fermi surfaces (Fig. R9a), however, NBFPs depend explicitly on the imaginary momentum $\boldsymbol{\mu}$, and their distributions can merge, annihilate, or emerge as $\boldsymbol{\mu}$ varies (Fig. R9b).

As the natural generalization of Fermi surfaces within non-Hermitian band structures, NBFP also has its density

$$\rho_{\boldsymbol{\mu}}(E) = \frac{\sum_\nu \delta(E - E_\nu^{\boldsymbol{\mu}})}{N_E},$$

where N_E is the number of eigenenergies. It is similar to the conventional density of states but is defined in terms of wavefunctions with a fixed decay length $\boldsymbol{\mu}$. We show that it can be computed from the spectral potential through

$$\rho_{\boldsymbol{\mu}}(E) = \frac{\nabla_E^2 \phi(E, \boldsymbol{\mu})}{2\pi}.$$

Figure R9c displays the NBFP density for three values of $\boldsymbol{\mu}$, and the corresponding wavefunctions with such a decay factor are shown on the left. Clearly, the NBFP density characterizes how energies associated with a given decay parameter $\boldsymbol{\mu}$ are distributed in the complex-energy plane (Fig. R9c), and therefore provides a statistical description of the spectral structures that may support wavefunctions with the corresponding decay profile.

More crucially, the NBFP density has its own set of conserved quantities, namely spectral moments. One can define spectral moments associated with $\rho_{\boldsymbol{\mu}}(E)$ as

$$M_n = \int (E - E_{\mathrm{ref}})^n \rho_{\boldsymbol{\mu}}(E)\, \mathrm{d}^2 E = \int \left(\frac{dk}{2\pi}\right)^d \mathrm{Tr}\left(\left(H_{\boldsymbol{\mu}}(\boldsymbol{k}) - E_{\mathrm{ref}}\right)^n\right), n \in \mathbb{N}.$$

As discussed in the Supplementary Section VI, we show that these moments are conserved quantities independent of $\boldsymbol{\mu}$. Furthermore, spectral moments are also conserved for arbitrary OBC configurations in the large-N limit. Figure R9d shows this relation experimentally using both non-Bloch supercell and OBC spectral data.

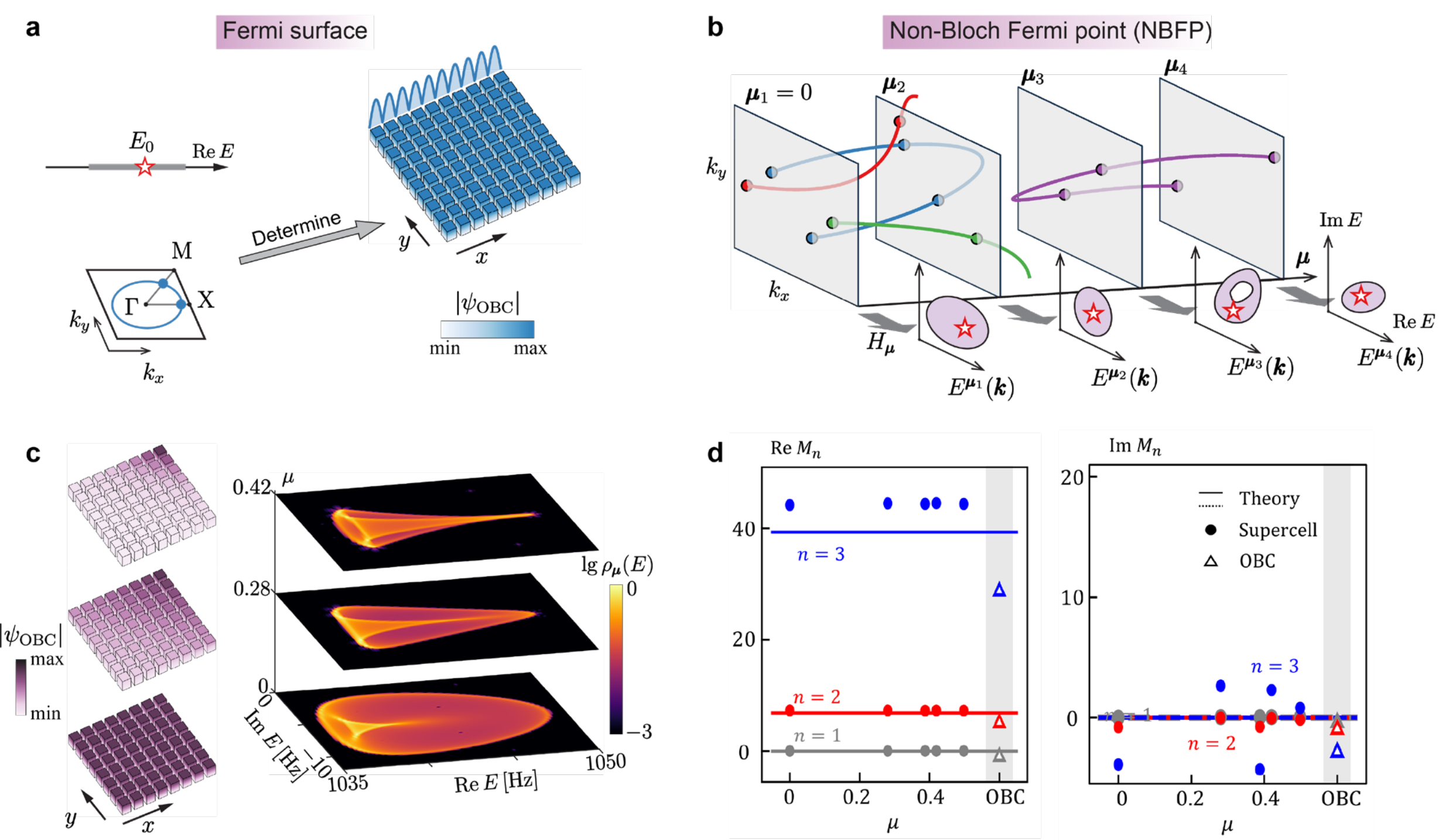

Fig. R9 | NBFPs as organizers of non-Hermitian spectral structures. a, A typical Fermi surface corresponding to E_0 in a Hermitian system. **b**, Evolution of non-Bloch Fermi points (NBFPs) with $\boldsymbol{\mu}$. The insets illustrate the map $H_{\boldsymbol{\mu}}: \boldsymbol{k} \mapsto E$ from the Brillouin zone (BZ) to the complex spectrum $E^{\boldsymbol{\mu}}(\boldsymbol{k})$ for different values of $\boldsymbol{\mu}$. For a chosen energy E_b (star), the preimages in momentum space are indicated by colored spheres and define the corresponding NBFPs. **c**, NBFP density $\rho_{\boldsymbol{\mu}}(E)$ for three values of $\boldsymbol{\mu}$ and the corresponding spatial wavefunction profiles. **d**, Spectral moments associated with $\rho_{\boldsymbol{\mu}}(E)$. Real (left) and imaginary (right) parts of the moments M_n for $n = 1,2,3$. Solid and dashed lines denote the theoretical moments calculated from the Bloch Hamiltonian through $\int \left(\frac{dk}{2\pi}\right)^d \mathrm{Tr}\left(\left(H_{\boldsymbol{\mu}}(\boldsymbol{k}) - E_{\mathrm{ref}}\right)^n\right)$, while circles and triangles denote moments obtained from non-Bloch supercell measurements and experimental OBC spectra, respectively. The experimental data are taken from Figs. 5 and 6 of the revised main text.

2. NBFP locations: interference directions selected by non-Bloch momenta

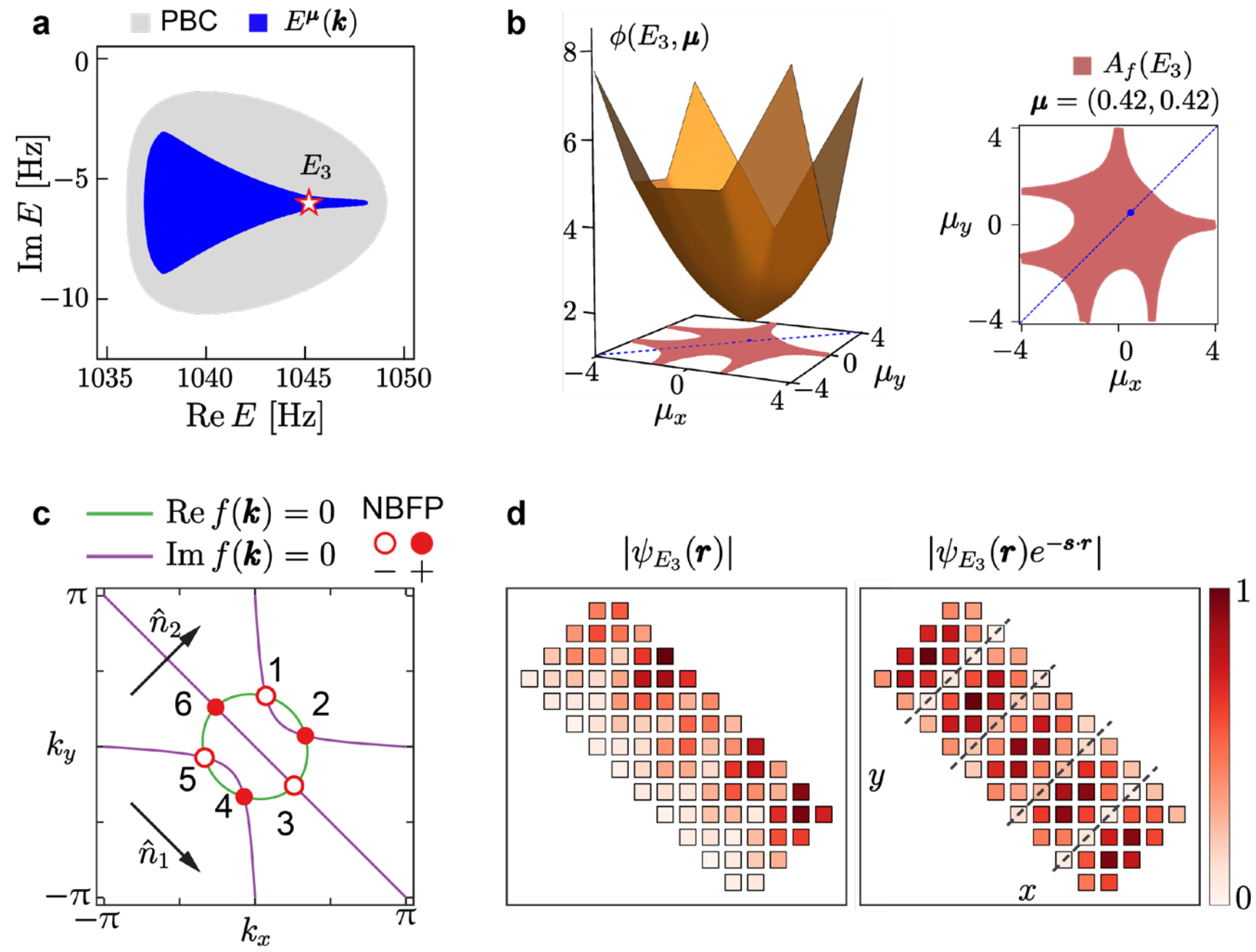

Fig. R10 | NBFP locations and symmetry. a, The grey area is the PBC spectrum; the blue area corresponds to $E^{\mu}(\boldsymbol{k})$ at $\boldsymbol{\mu} = (0.42{,}0.42)$ (marked by the blue point in panel **b**). The red star marks the reference energy E_3. **b**, Spectral potential $\phi(E_3, \boldsymbol{\mu})$ (orange surface) and the amoeba $A_f(E_3)$ (shaded red) for the 2D model. The right panel shows a close-up of $A_f(E_3)$; the solid line laid on the amoeba marks the symmetry axis $\mu_x = \mu_y$. **c**, NBFP distributions (left) corresponding to the selected $\boldsymbol{\mu}$. The NBFPs exhibit mirror symmetries with respect to both $k_x = k_y$ and $k_x = -k_y$, reflecting the underlying symmetry of the Hamiltonian and the associated standing-wave interference patterns. **d**, The measured real-space eigenstate (left) and the corresponding compensated wavefunctions $\left|e^{-\boldsymbol{s}\cdot\boldsymbol{r}}\psi_{E_3}(\boldsymbol{r})\right|$ (right) with corresponding $s = \mu_{\min} = 0.42$. Dashed lines are plotted to indicate the interference nodes of $|e^{-\boldsymbol{s}\cdot\boldsymbol{r}}\psi_E(\boldsymbol{r})|$.

From the band-structure perspective, the distribution of NBFPs corresponding to a given energy E and imaginary momentum $\boldsymbol{\mu}$ directly reflects the symmetry of the system. Taking the 2D model in the main text as an example, the Hamiltonian satisfies the exchange symmetry

$$H(\beta_x, \beta_y) = H(\beta_y, \beta_x),$$

which leads to a mirror symmetry of the corresponding amoeba with respect to the line $\mu_x = \mu_y$ (Fig. R10a-b), as shown in Supplementary Section II. Furthermore, for $\boldsymbol{\mu}$ located on this symmetry

axis, the corresponding NBFP distributions of E_3 (red star in Fig. R10a) exhibit mirror symmetries with respect to both $k_x = k_y$ and $k_x = -k_y$ (Fig. R10c).

Physically, these NBFPs determine the possible standing-wave interference patterns that can be formed at the corresponding energy (Fig. R10d). As a consequence, OBC geometries, which make these standing waves occur, must tend to preserve the underlying symmetry of the system. The detailed influence of a specific boundary geometry on the selection of NBFPs is discussed in response to Comment 6 and is not repeated here. In short, it illustrates that NBFPs not only encode the complex band geometry but also characterize the symmetry and interference structure of boundary-supported wavefunctions.

3. NBFP signatures under OBC measurement

More importantly, NBFPs are not merely theoretical constructs but correspond to experimentally observable quantities that can be independently measured under OBCs.

First, the k-μ decoupling implies that we can perform $e^{-\boldsymbol{s}\cdot\boldsymbol{r}}\psi_E(\boldsymbol{r})$ to an OBC eigenstate $\psi_E(\boldsymbol{r})$ for investigating its decay behavior. Experimentally measuring eigenstates is now feasible through techniques such as Green's-function measurements [e.g., Phys. Rev. B 112, L220301 (2025)] and state tomography [e.g., Science 352, 6289 (2016)]. We therefore begin with an OBC eigenstate to illustrate our point. Figures R11a and R11b depict a staggered Hatano-Nelson model and one OBC eigenstate $\psi_{E_1}(\boldsymbol{r})$, respectively. Introducing an imaginary-momentum compensation factor $e^{-\boldsymbol{s}\cdot\boldsymbol{r}}$, we define the inverse participation ratio (IPR) to the resulting $e^{-\boldsymbol{s}\cdot\boldsymbol{r}}\psi_E(\mathbf{r})$

$$\mathrm{IPR} = \frac{\int \left|e^{-\boldsymbol{s}\cdot\boldsymbol{r}}\psi_E(\boldsymbol{r})\right|^4 d\boldsymbol{r}}{\left(\int \left|e^{-\boldsymbol{s}\cdot\boldsymbol{r}}\psi_E(\boldsymbol{r})\right|^2 d\boldsymbol{r}\right)^2}.$$

Figure R11c shows the corresponding IPR as a function of $\mathbf{s}$. Since smaller values of IPR correspond to more extended states, we seek values of $\boldsymbol{s}$ for which the compensated wavefunction $e^{-\boldsymbol{s}\cdot\boldsymbol{r}}\psi_E(\boldsymbol{r})$ becomes as close as possible to an extended state. For exponentially localized OBC wavefunctions, an appropriate compensation can effectively restore the state toward a clean extended profile, as illustrated in the bottom panel of Fig. R11d, where the IPR approaches $1/N$. In short, the behavior of the IPR minimum for $e^{-\boldsymbol{s}\cdot\boldsymbol{r}}\psi_E(\boldsymbol{r})$ dictate the localization nature of the eigenstate.

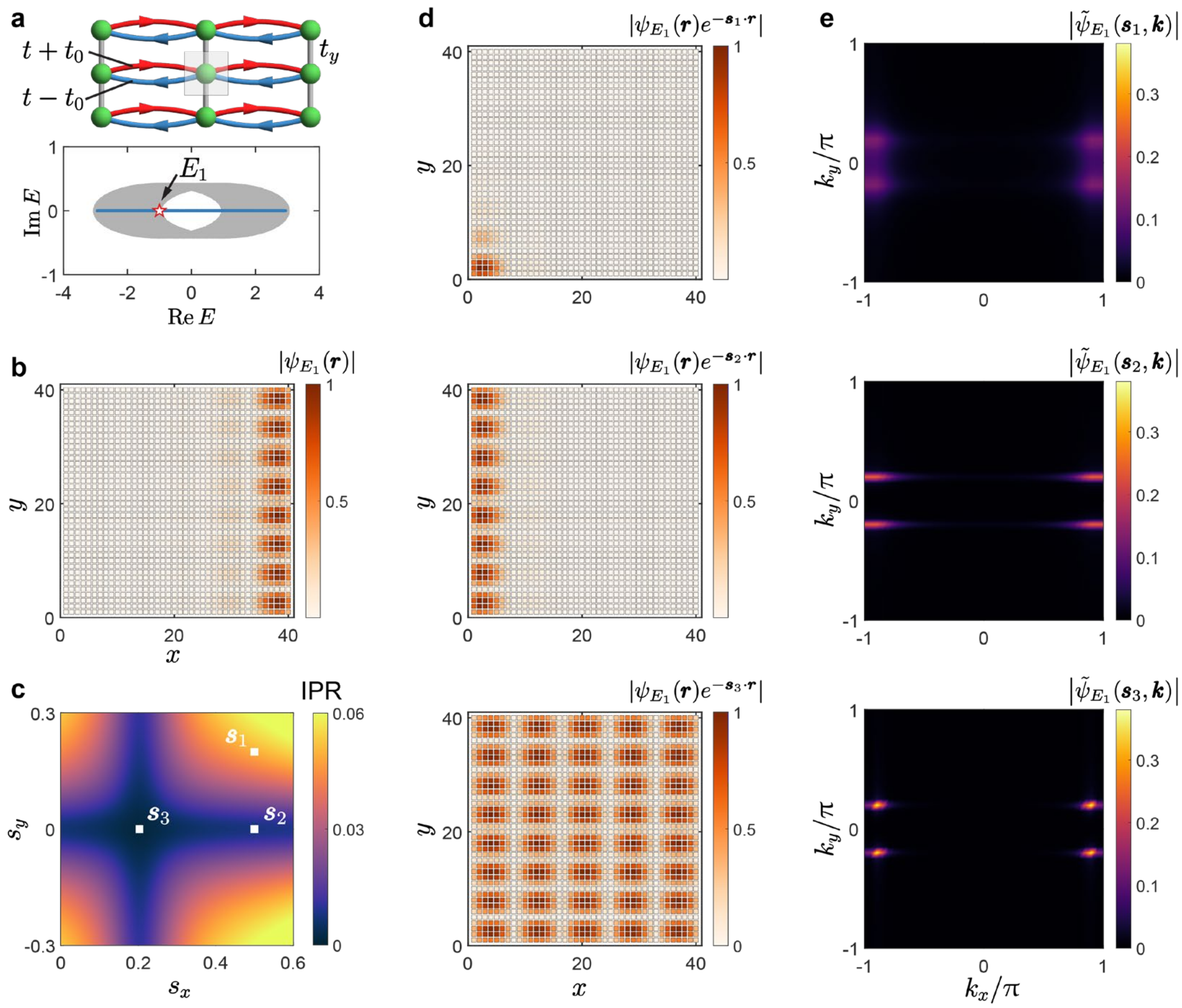

Fig. R11 | Insights for probing non-Hermitian systems without programmable hopping. a, Tight-binding model (top) and OBC spectrum (bottom). The Hamiltonian is given by $H_{\mathrm{HN}}(\boldsymbol{\beta}) = (t - t_0)\beta_x + (t + t_0)\beta_x^{-1} + t_y(\beta_y + \beta_y^{-1})$. **b**, An OBC eigenstate $|\psi_{E_1}(\boldsymbol{r})|$ with $E_1 = -1.0$, marked by the star in the top panel, which typically exhibits exponential localization toward the boundary in non-Hermitian systems. **c**, Inverse participation ratio (IPR) of the compensated wavefunction $e^{-\boldsymbol{s}\cdot\boldsymbol{r}}\psi_{E_1}(\boldsymbol{r})$. The IPR reaches its minimum at $\boldsymbol{s} = \boldsymbol{s}_3$. **d**, Wavefunctions compensated by different values of $\boldsymbol{s}$, *i.e.*, $\boldsymbol{s}_1 = (0.5,0.2)$, $\boldsymbol{s}_2 = (0.5,0.0)$, and $\boldsymbol{s}_3 = (0.2,0.0)$. The imaginary-momentum compensation amplifies information that would otherwise be obscured by exponential decay. **e**, Fourier transform of the compensated wavefunction. When $\boldsymbol{s}$ reaches the optimal compensation value, the Fourier intensity is maximized, generating hot spots in momentum space corresponding to the NBFPs selected by the boundary. The parameters used are $t = 1$, $t_0 = 0.2$, and $t_y = 0.5$.

Second, NBFPs provide an experimentally meaningful observable for detecting complex momentum, regardless of whether the hopping parameters are programmable. Performing an s-resolved Fourier-Laplace transform on the eigenstate yields

$$\tilde{\psi}_E(\boldsymbol{s},\boldsymbol{k}) = \int d\boldsymbol{r}\ [e^{-\boldsymbol{s}\cdot\boldsymbol{r}}\psi_E(\boldsymbol{r})]e^{-i\boldsymbol{k}\cdot\boldsymbol{r}},$$

which corresponds to Eq. (5) in the main text and is illustrated in Fig. R11e. The resulting hot spots in momentum space—namely, the Fourier peaks obtained at the compensation parameter where the IPR is minimized—correspond to the NBFPs. As demonstrated in Figs. 3 and 5 of the main text, $|\tilde{\psi}_E(\boldsymbol{s},\boldsymbol{k})|$ is maximized when the compensation parameter $\boldsymbol{s}$ matches the optimal value $\boldsymbol{\mu}_{\min}$, corresponding to the case where the exponentially localized wavefunction is most effectively restored toward an extended state. The locations of these NBFPs in the $\boldsymbol{k}$-space reflect the underlying interference structure of wavefunctions. In this way, one can investigate the imaginary part of momentum and thereby infer the full complex wavevector $\boldsymbol{k} + i\boldsymbol{\mu}$.

In brief, for electronic materials in which programmable couplings are difficult to implement, the k-μ decoupling perspective and the ensuing NBFPs offer an alternative toolkit for investigating non-Hermitian behaviors once the eigenstates become experimentally accessible.

To clarify the conceptual role of NBFPs, we have added the following discussion to the end of the *Boundary-activated non-Bloch Fermi points in 2D lattices* section of the revised manuscript:

"Since the NBFP localizations closely relate to the underlying symmetry, for a given boundary geometry in Fig. 6c, the selection can be understood as standing-wave interference between different NBFP pairs satisfying the boundary constraints. To reveal, we compensate for the exponential decay by applying $e^{-\boldsymbol{s}\cdot\boldsymbol{r}}\psi_E(\boldsymbol{r})$ with $\boldsymbol{s} = \boldsymbol{\mu}_{min}$, as shown in the right panels of Figs. 6e-f, where the upper and lower panels correspond to the experimental and theoretical results, respectively. For E_3 (Fig. 6e), the compensated wavefunction exhibits four nodes along $\hat{n}_1$ and none along $\hat{n}_2$, whereas for E_4(Fig. 6f), it exhibits four nodes along $\hat{n}_1$ and two along $\hat{n}_2$. These interference nodes can be inferred by the momentum separations between the corresponding NBFP pairs together with the standing-wave conditions (see Supplementary Section VIII).

The conceptual role of NBFPs in non-Hermitian band theory becomes transparent, especially when going beyond 1D scenarios. The NBFP density determines the spectral structure of fixed-decay-length wavefunctions and relates to OBC behaviours via a conservative quantity, namely, the spectral moment[47]. The NBFP locations specify interference directions of non-Bloch waves

supported by particular geometries. Detecting NBFPs from OBC measurements using an s-resolved Fourier-Laplace transform offers a practical approach to determining localization behaviours of skin modes (Supplementary Section IX). Therefore, NBFPs are not only indicators of non-Bloch spectral topology but also observable quantities that can be directly probed and independently verified in OBC experiments."

To further illustrate the NBFP signatures in the OBC measurement and its physical scope, we have added the following discussions to the *Discussions and conclusions* section (second paragraph on page 14) in the revised main text as:

"A key physical insight of our non-Bloch supercell framework is that k-μ decoupling converts imaginary momentum from an implicit analytic continuation into an experimentally controllable and physically interpretable variable. Probing non-Hermitian band structures can therefore be reformulated in terms of NBFPs, which link the spectral deformation induced by $\boldsymbol{\mu}$ to the spatial amplification or attenuation of eigenstates. Applying an s-resolved Fourier-Laplace transform to OBC eigenstates to extract NBFPs further demonstrates the necessity of imaginary-momentum compensation for recovering information lost due to non-Hermitian attenuation, providing a general strategy for recovering boundary-sensitive information in real-space non-Hermitian systems with open boundaries. This connection remains conceptually useful even in systems where hopping amplitudes cannot be directly programmed, as it provides a way to classify the relationship between spectral features and eigenstate decay behaviors within a unified non-Bloch description (Supplementary Section IX). "

3. The relation between twisted boundary conditions and the periodic/non-Bloch analysis requires clearer exposition.

The experimental implementation relies on twisted boundary conditions, whereas the theoretical interpretation is framed in terms of non-Bloch band structure and associated winding properties. The manuscript should explain this connection in a more transparent and systematic way. Since this point is central to the interpretation of the measured data as bona fide band-structure information rather than as a feature of a particular finite setup, the present discussion is not yet sufficiently clear.

Response:

Thank you for raising this important issue and for recognizing the crucial role of twisted boundary conditions (TBCs). Complex-continuation TBCs establish the correspondence between non-Bloch band structures and their supercell implementation, while k-μ decoupling further gives rise to the non-Bloch supercell framework for the controlled probing of non-Hermitian band structures. In the following, we detail this relationship.

TBCs can be understood by treating a finite-size system as a supercell and imposing twisted angles to continuously scan the momentum. Conventional TBCs modify the periodic boundary condition (PBC)

$$\psi(\boldsymbol{r} + L_m \hat{n}_m) = \psi(\boldsymbol{r})$$

to

$$\psi(\boldsymbol{r} + L_m \hat{n}_m) = e^{i\theta_m} \psi(\boldsymbol{r}),$$

where θ_m is the twist angle along the m direction. In systems with well-defined Bloch momenta, varying the twist angles is equivalent to shifting the Bloch momenta and therefore provides a standard approach for probing band structures in finite-size systems. The connection to our framework can then be understood through a three-step construction, progressing from conventional Bloch-momentum sampling to a full non-Bloch implementation.

(i) Equivalence between twisted boundary conditions and the Bloch description

For an $N_x \times N_y$ supercell, a given set of twist angles (θ_x, θ_y) generates a shifted set of primitive-cell Bloch momenta as shown in the first row on the right of Fig. R12a,

$$(k_x, k_y) = \left(\frac{\theta_x}{N_x} - \pi + \frac{2\pi v_x}{N_x}, \frac{\theta_y}{N_y} - \pi + \frac{2\pi v_y}{N_y}\right),$$

with $v_{x(y)} = 1, \ldots, N_{x(y)}$. In this way, TBCs increase the momentum-space sampling density through band folding while remaining within the Bloch framework.

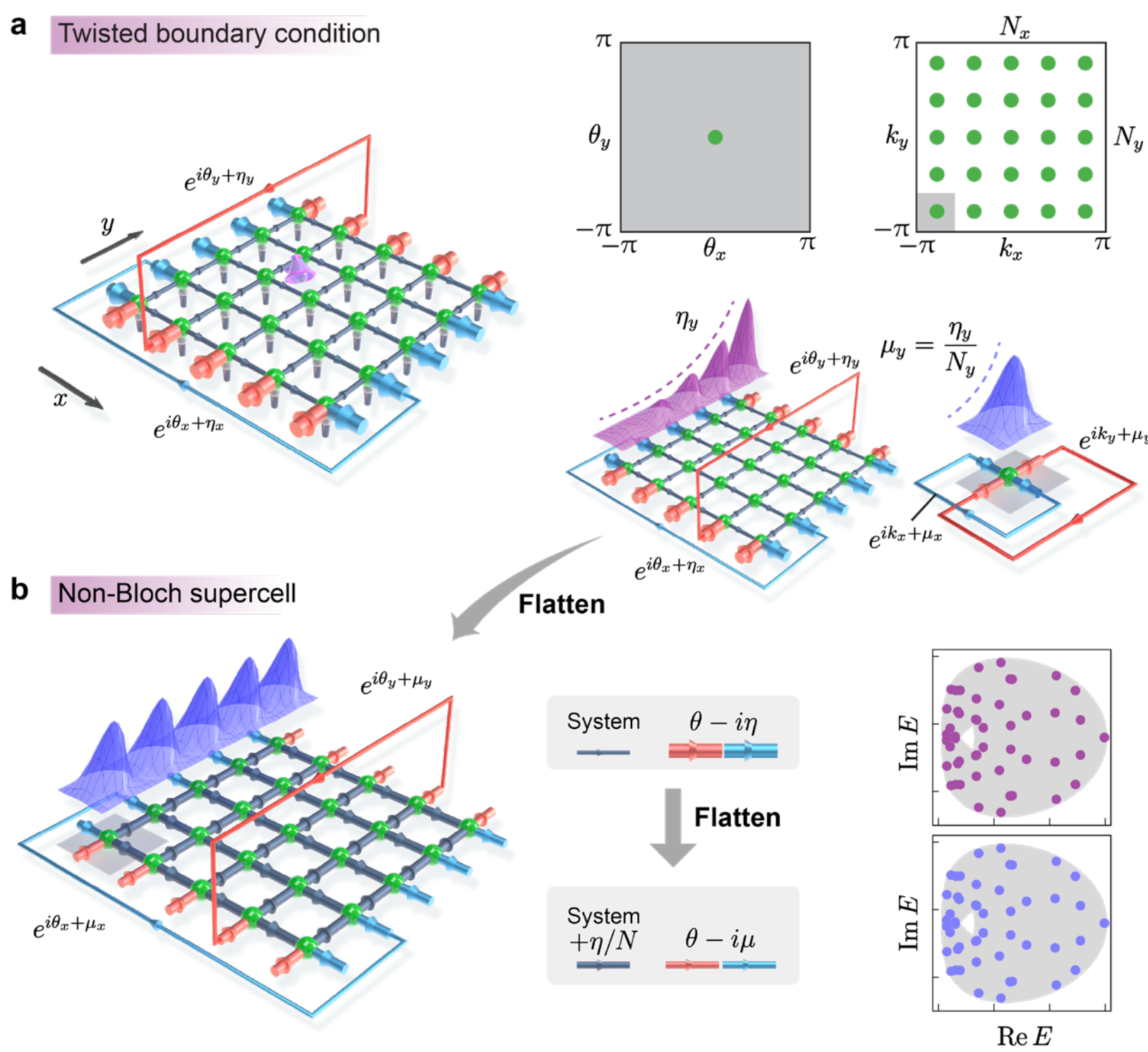

Fig. R12 | Relation between twisted boundary conditions and non-Bloch analysis within the supercell framework. a, Schematic illustration of a supercell with analytically continued TBCs, $\boldsymbol{\theta} - i\boldsymbol{\eta}$ (left). The red and blue thick lines highlight the hopping modifications induced by $\boldsymbol{\theta}$ and $\boldsymbol{\eta}$ at the supercell boundaries, while the thin gray lines denote hoppings of the system under consideration. The first and second rows on the right depict the equivalence of the real and imaginary momentum components, respectively, between complex-continuation TBCs (first column) and primitive-cell level non-Bloch analysis (second column). **b**, The non-Bloch supercell framework. The left panel displays the non-Bloch supercell with $e^{i\boldsymbol{\theta}}$ applied at the boundaries (red and blue thin lines) and the flattening factor $e^{\boldsymbol{\eta}}$ distributed across the entire lattice at each intercell hopping (gray lines). Purple circles in the top-right panel show spectra obtained using complex-continuation TBCs, which coincide with those from the non-Bloch supercell framework (blue circles in the bottom-right panel). N_x (N_y) denotes the number of unit cells along the x (y) direction.

(ii) Equivalence between complex-continuation TBCs and non-Bloch description

We next extend the twist angles into the complex plane through

$$\theta_m \to \theta_m - i\eta_m.$$

This corresponds to a complex continuation of the momentum. The relationship between θ_m and k_m still holds. Here, η_m encodes the imaginary component of momentum, as shown in the bottom-right panels of Fig. R12a. Because exponential attenuation accumulates across the entire supercell, the corresponding supercell parameter satisfies

$$\eta_m = N_m \mu_m,$$

where μ_m denotes the primitive-cell non-Bloch imaginary momentum. Such a complex-continuation TBC configuration (Fig. R12a, left) is equivalent to the configuration in the second row and column on the right of Fig. R12a. The single-cell limit is recovered by setting $N = N_x \times N_y = 1$.

(iii) Non-Bloch supercell: μ-flattening implementation

After introducing the complex-continuation TBCs at the supercell level, the accumulated attenuation $\boldsymbol{\eta}$ can be redistributed uniformly into the internal hoppings of the supercell through the μ-flattening procedure, as shown in Fig. R12b. By transforming the boundary attenuation into a distributed hopping profile, this procedure decouples the control of $\boldsymbol{k}$ and $\boldsymbol{\mu}$, yielding a real-space implementation that is spectrally equivalent to the complex-continuation TBC but experimentally more accessible and robust [see Eq. (2) and related discussions in the main text for details].

In this construction, step (i) serves as the Bloch starting point, step (ii) establishes the correspondence between non-Bloch physics and the supercell description, while step (iii) gives rise to the non-Bloch supercell framework for experimentally probing non-Hermitian band structures.

To clarify this relation in a more transparent and systematic way, we have revised Figs. 2a-b to Fig. R12 and the related discussions to the *Non-Bloch supercell framework* section (third paragraph on page 4 of the revised manuscript) as follows:

"Establishing such a connection experimentally requires a protocol that links non-Bloch analysis with OBC physics. Following the Hermitian recipe, a system with TBCs serves as a practical tool for establishing such a connection. However, generalizing this approach to non-Hermitian systems is nontrivial due to the presence of imaginary momentum. To clarify these challenges, we start with an $N_x \times N_y$ supercell with TBCs and assess the tunability of $\boldsymbol{\mu}$ afforded by the boundary twists. A twisted-phase factor $e^{i\theta_{x,y}}$ with $\theta_{x,y} \in (-\pi, \pi]$ is applied across each

supercell boundary (Fig. 2a). This construction folds the primitive-cell band structure (Supplementary Section I), usually computed over the BZ parameterized by $\boldsymbol{k} = (k_x, k_y)$. *In systems with translational symmetry, varying the twist phases is equivalent to shifting the Bloch momenta and therefore provides a standard approach for probing band structures in finite-size systems. Increasing the supercell size increases the number of BZ sampling points along each direction,* $N_{k_m} \propto N_m$ *for* $m = x, y$ *(Fig. 2a, top-right). In non-Hermitian systems, extending the twist phase into the complex plane naturally provides access to the imaginary momentum and the associated non-Bloch band structure, i.e., the analytic continuation* $\theta_m \to \theta_m - i\eta_m$ *indicates multiplying the inter-supercell hopping along direction* m *by* $e^{\eta_m} e^{i\theta_m}$*, which is equivalent to the configuration in the bottom-right panels of Fig. 2a. It preserves* $N_{k_m} \propto N_m$ *because sampling points still follow Fig. 2a for any* η_m*, while providing access to imaginary parts of the primitive-cell momenta* $\boldsymbol{\mu} = (\mu_x, \mu_y)$ *as* $\mu_m = \eta_m / N_m$*. At this stage, we have established the equivalence between complex-continuation TBCs and the non-Bloch description."*

4. The strongest contribution of the work appears methodological rather than physical.

In my view, the most compelling aspect of the manuscript is the protocol itself: the separation of k and μ, the removal of the associated tradeoff through exponent flattening, and the experimental reconstruction of complex spectra and biorthogonal eigenstates. By contrast, the one-dimensional results mainly serve to validate that this framework reproduces the expected open-boundary spectra and localized states. This is certainly important, but for publication in Nature Communications the broader conceptual or physical significance enabled by the method should be articulated more convincingly.

Response:

Thank you very much for this comment and for the positive assessment of our work. We agree with the reviewers' summary of our methods and the one-dimensional results. At the same time, we would like to emphasize that NBFPs enabled by our framework represent the broader conceptual/physical significance. Besides its theoretical connotation, NBFP also explains how to classify various skin effects and investigate large-*N* physics experimentally.

1. Conceptual significance enabled by NBFPs

As detailed in our response to your second comment, the density of NBFPs determines the spectral structure of fixed-decay-length wavefunctions, their locations forecast the interference directions selected by non-Bloch momenta, and their detection via OBC measurements provides a tool for distinguishing different classes of skin effects.

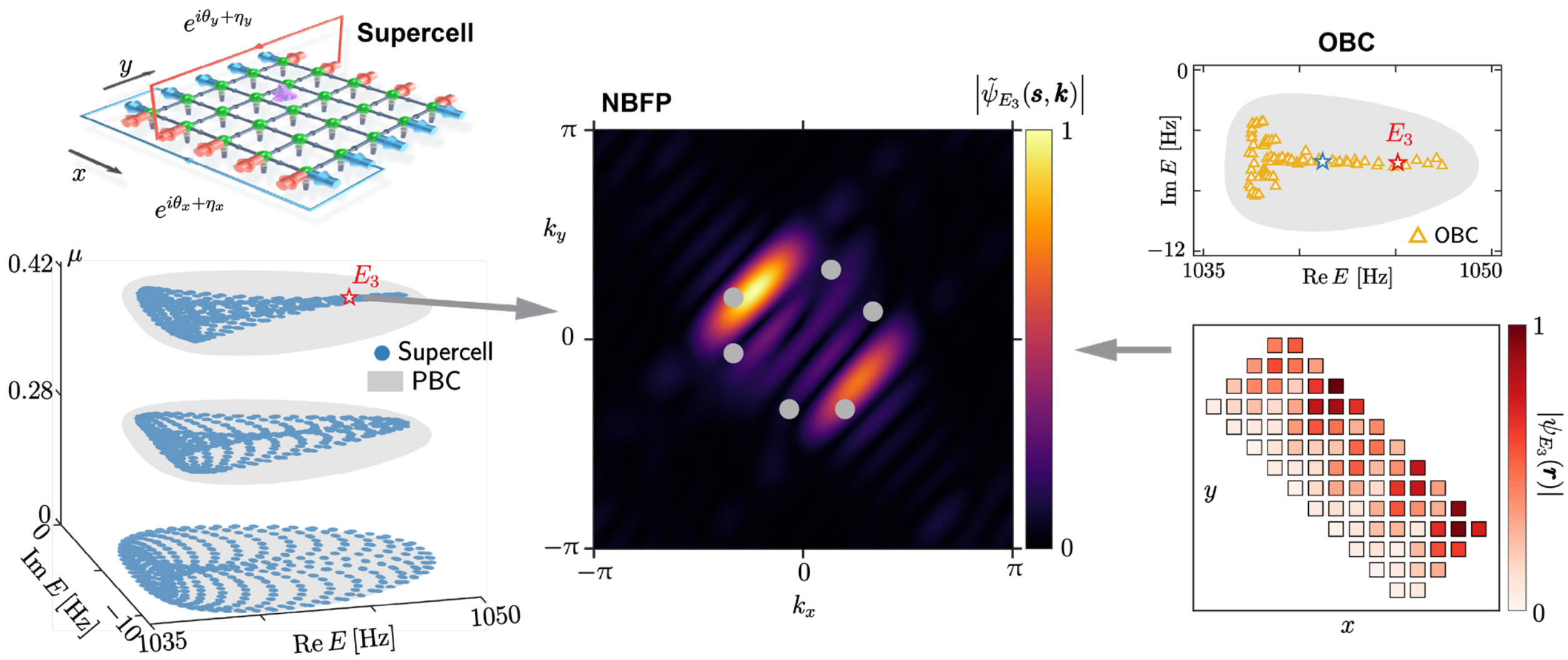

Fig. R13 | Identification of NBFPs as physically meaningful observables. NBFPs shown in the middle can be independently determined from the non-Bloch supercell framework (left) and from open-boundary measurements (right), providing mutual validation.

The excellent agreement between the NBFPs independently obtained from the non-Bloch supercell framework and from OBC measurements not only highlights the physical relevance of NBFPs but also establishes them as experimentally observable quantities whose significance extends beyond non-Bloch band reconstruction alone.

Figure R13 demonstrates such significance. The middle panel shows a typical NBFP result obtained with our non-Bloch supercell framework, along with OBC measurements. As detailed in the above reply and in the main text (Fig. R13, left), the non-Bloch supercell framework can experimentally determine the NBFP positions (grey circles in the middle panel of Fig. R13) and predict the geometries that support interference between non-Bloch momenta. Meanwhile, once the real-space OBC eigenstate is obtained (Fig. R13, right), an s-resolved Fourier-Laplace

transform can be performed to investigate the localization nature of the eigenstate (see also the reply to the second comment for details). Since the OBC wavefunction shown in Fig. R13 is exponentially localized, an appropriate compensation can effectively restore the state toward a nearly extended profile, causing the IPR minimum for $e^{-\boldsymbol{s}_{\min}\cdot\boldsymbol{r}}\psi_E(\boldsymbol{r})$ approaches $1/N$ at a unique value of $\boldsymbol{s}_{\min}$. The resulting hot spots of $\left|\tilde{\psi}_E(\boldsymbol{s},\boldsymbol{k})\right|$ then dictate the NBFP positions, as shown by the color plot in the middle of Fig. R13.

2. Implication: experimentally classifying skin effects and investigating large-*N* physics.

Since skin effect is not limited to exponentially localized cases, and because NBFPs can also be probed through OBC measurements, we now use the critical-skin experiment (Fig. R14a) as an example to show that NBFPs can serve as an indicator for classifying various skin effects experimentally.

Figure R14c shows one OBC spectrum, while the top panels in Figs. R14d and R14e display the eigenstate distributions on chains A and B, respectively. The s-resolved Fourier-Laplace transform of the eigenstates on chains A and B is shown in the left panels of Figs. R14f and R14g, respectively. The resulting hot spots in momentum space, namely, the Fourier peaks obtained at the compensation parameter where the inverse participation ratio (IPR) is minimized ($s_{\min} = 0.01$ and $s_{\min} = 0.16$) for chains A and B, are respectively seen in the right panels of Figs. R14f and R14g. Only a single hot spot is observed for each chain, and the extracted values of $s_{\min}$ differ between the two chains. The compensated wavefunction $e^{-s_{\min}x}\psi_E(x)$ never approach extended states, as shown in the bottom panels in Fig. R14d-e. As a result, neither a reliable value of μ nor the corresponding NBFP structure can be inferred from the OBC measurements alone. It implies that this does not belong to the set of exponentially localized skin modes.

More broadly, similar phenomena can be observed in higher dimensions for the algebraic skin effect, and our recipe for detecting NBFPs via OBC measurements provides a tool for differentiating among various skin effects.

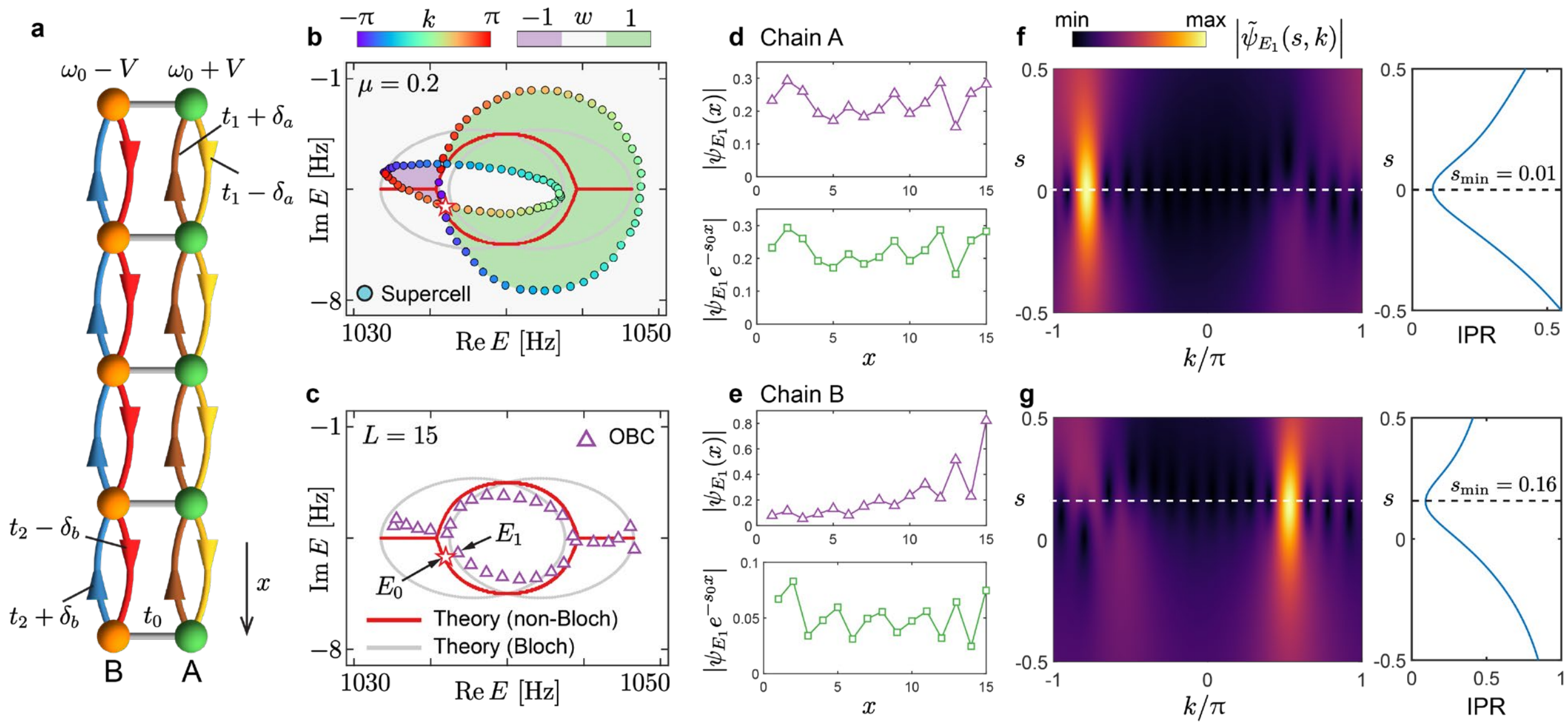

Fig. R14 | Non-Bloch supercell and OBC measurements for critical skin effects. **a**, A two-chain model supporting the critical skin effect. The lattice model is $H_{\mathrm{CSE}}(\beta) = \begin{pmatrix} g_A(\beta) & t_0 \\ t_0 & g_B(\beta) \end{pmatrix}$ with $g_A(\beta) = \omega_0 + t_A^+\beta + t_A^-\beta^{-1} + V$ and $g_B(\beta) = \omega_0 + t_B^+\beta + t_B^-\beta^{-1} - V$. **b**, Measured $E^\mu(k)$ at $\mu = 0.2$ obtained using the non-Bloch supercell framework (filled colour circles). The red star marks the self-intersection energy E_0, which resides on the OBC spectrum in the large-N limit. **c**, Measured OBC spectra (triangles) for a system size of $L = 15$. In both **b** and **c**, the grey lines indicate the theoretical PBC spectrum, and red solid lines denote the OBC spectrum in the large-N limit obtained from generalized Brillouin zone analysis. **d**,**e**, Measured real-space wavefunctions $\psi_{E_1}(x)$ (top) and the corresponding s-compensated wavefunctions $e^{-s_{\min}x}\psi_{E_1}(x)$ (bottom) for chain A (**d**) and B (**e**). The energy E_1 marked in **c** is chosen as the OBC eigenenergy closest to E_0 in **b**. **f**,**g**, s-resolved momentum-space wavefunction $\tilde{\psi}_{E_1}(s,k)$ (left) from open boundary lattices and the corresponding IPR distributions of $e^{-sx}\psi_{E_1}(x)$ (right) for chain A (**f**) and B (**g**). Here, $s_{\min}$ denotes the value of s at which the IPR reaches its minimum. The extracted values are $s_{\min} = 0.01$ and $s_{\min} = 0.16$ for chains A and B, respectively, neither of which coincides with the non-Bloch parameter predicted by the supercell framework.

Last but not least, our non-Bloch supercell framework is a toolkit for experimentally probing non-Bloch spectral and wavefunction information in the large-N limit. It is particularly important in cases such as critical-skin systems, where finite-size OBC measurements alone may not reliably

reflect the behaviour in the large-N limit, as shown in Figs. R14b and R14c (see Fig. 4 in the revised main text for details). More broadly, analogous limitations arise in a wide range of higher-dimensional non-Hermitian systems. Our framework, therefore, overcomes constraints imposed by boundary geometry, system size, and other finite-size effects.

To highlight the broader conceptual and physical significance of our work, we have made the following revisions:

- Adding a new paragraph to the *Discussions and conclusions* section of the revised main text to illustrate the significance of NBFPs and their signatures in OBC measurements.
- Adding a new Supplementary section IX, presenting several examples of how NBFP signatures can be identified from OBC measurements.
- Adding a new Fig. 4 on the critical skin effect, together with related discussions in the revised main text, to highlight the necessity and capability of our framework for accessing large-N physics.

5. The generality of the framework should be clarified.

It is not clear from the current presentation whether the proposed framework can also diagnose regimes in which the skin effect is absent, or whether it is primarily effective in situations where the relevant open-boundary states already display pronounced boundary localization. This issue is important for assessing the real scope and generality of the method, and it should therefore be addressed explicitly.

Response:

Thank you for raising this important question. Our framework does not rely on the presence of skin effect; therefore, it can be applied equally well to both Hermitian and non-Hermitian lattices, with or without skin accumulation. More fundamentally, the non-Bloch supercell framework is built upon the equivalence between complex-continuation twisted boundary conditions and the non-Bloch description, rather than on the existence of skin modes themselves. As a result, its applicability extends well beyond skin-effect physics and provides an experimental platform for characterizing quantities defined on eigenenergies and eigenstates, including band dispersions, Berry curvature, and related spectral and topological properties.

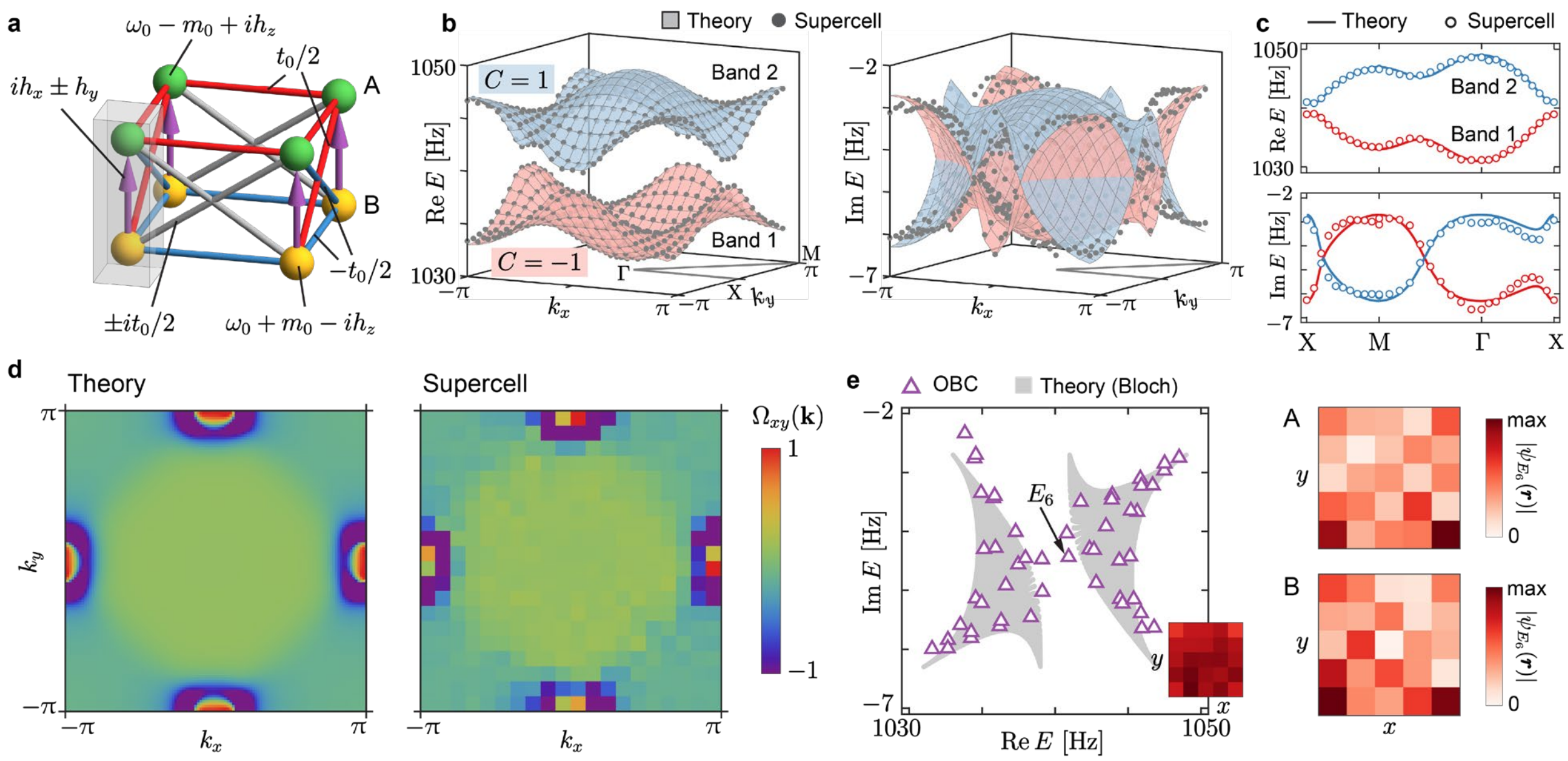

Fig. R15 | Generalizations of the non-Bloch supercell framework: band structures and Berry curvature. **a**, Schematic of a 2D non-Hermitian Chern insulator. The used Hamiltonian is $H_{\text{CI}}(\boldsymbol{\beta}) = \omega_0\sigma_0 + \boldsymbol{\sigma} \cdot [\boldsymbol{d}(\boldsymbol{\beta}) + i\boldsymbol{h}]$, where $\boldsymbol{d}(\boldsymbol{\beta}) = [-it_0(\beta_x - \beta_x^{-1}), -it_0(\beta_y - \beta_y^{-1}), t_0(\beta_x + \beta_x^{-1} + \beta_y + \beta_y^{-1}) - 2m_0]/2$, σ_0 is the identity matrix, $\boldsymbol{\sigma} = (\sigma_x, \sigma_y, \sigma_z)$ denotes the Pauli matrices, and $\boldsymbol{h} = (h_x, h_y, h_z) \in \mathbb{R}^3$. **b**, Measured non-Hermitian band structures $\text{Re}\, E^{\boldsymbol{\mu}}(\boldsymbol{\beta})$ (left) and $\text{Im}\, E^{\boldsymbol{\mu}}(\boldsymbol{\beta})$ (right) at $\boldsymbol{\mu}_{\text{min}} = (0,0)$. The surfaces represent theoretical predictions, while the filled spheres denote experimental measurements. **c**, Band dispersions along the high-symmetry lines indicated in **b**, showing excellent agreement between theory (lines) and experiment (circles). **d**, Berry curvature distributions reconstructed from experimentally measured eigenvectors (right), compared with theoretical calculations (left). **e**, OBC measurements on a finite lattice containing 50 sites. Left: OBC spectra (triangles). The gray background denotes the theoretical PBC spectra. The inset shows the spectral sum of bulk states, confirming the absence of NHSE. Right: measured real-space wavefunction of the A-sublattice at the selected energy E_5 (top) and the corresponding Fourier transforms (bottom). Gray circles mark NBFPs obtained from non-Bloch supercells. The parameters used are $\omega_0 = (1040 - 4.5i)$ Hz, $m_0 = -1$ Hz, $t_0 = 4$ Hz, $h_x = h_y = 0$ Hz, and $h_z = 1.8$ Hz. The supercell is composed of $N_x = N_y = 1$ unit cell, corresponding to the single-cell limit of our non-Bloch supercell framework.

To explicitly demonstrate the generality of our framework, we have deployed a non-Hermitian Chern insulator (Fig. R15a) that respects spectral reciprocity (Fig. R15b). Spectral reciprocity guarantees the absence of nonreciprocal skin effect in this system. Figure R15c shows the real and imaginary parts of the eigenenergies along a typical high-symmetry line. A Re E line gap is clearly observed, thereby enabling experimental investigations of the associated Chern topology and topological boundary modes.

Both left and right eigenstates are experimentally accessible within our framework, so we can directly reconstruct the Berry curvature from the measured eigenstates. Figure R15d compares the experimentally reconstructed Berry-curvature distribution (right) with the corresponding theoretical result (left) for the upper band. Integrating the Berry curvature over the Brillouin zone yields the Chern number of each band, as indicated in Fig. R15b.

According to the bulk-boundary correspondence, we next investigate the OBC behavior experimentally. Figure R15e shows the measured OBC spectrum (left), where topological boundary modes emerge within the Re E line gap. The inset displays the spectral sum of the bulk states, confirming the absence of skin accumulation. Finally, we select the OBC eigenstate at E_5 (Fig. R15e, top right) and perform Fourier transforms of its A-sublattice component. The resulting momentum-space hot spots agree remarkably well with the NBFPs predicted by the supercell framework, as shown in the bottom-right panel of Fig. R15e.

In summary, these results demonstrate that our framework remains fully applicable even in the absence of the skin effect. More broadly, this example shows that the non-Bloch supercell framework naturally extends beyond skin-effect diagnostics to multiband non-Hermitian systems with nontrivial topology, enabling the experimental reconstruction of biorthogonal quantum geometry and topological invariants.

To highlight that the non-Bloch supercell framework provides information beyond the skin effect, we have added a new section entitled *Non-Bloch band reconstruction beyond the skin effect*, together with a new figure (Fig. 7) and a new Methods section entitled *Experimental measurement of Berry curvature and Chern numbers*, to the revised main text.

6. The two-dimensional results would benefit from a stronger predictive interpretation.

The observation that different open-boundary eigenstates activate different combinations of NBFPs is interesting. However, the manuscript does not yet establish whether one can predict, for a given energy and boundary geometry, which NBFPs will be selected. In the absence of such a predictive principle, the two-dimensional part reads more as an application of the method than as a broader conceptual advance of independent significance.

Response:

Thank you for this insightful question. This comment has motivated us to clarify further the predictive role of NBFPs in higher-dimensional systems, which we believe represents an important conceptual aspect of the present framework. The distribution of NBFPs for a given energy is an intrinsic property of the non-Hermitian band structure and independent of any particular open-boundary geometry. How different NBFPs interfere to form standing-wave patterns that satisfy boundary conditions depends sensitively on many details, making a complete description, in general, highly complicated and challenging. However, by employing symmetry and focusing on specific symmetric systems, we can predict possible interference patterns for a given boundary geometry.

1. Energy windows of NBFP-compatible standing waves

We take the example shown in Figs. 5 and 6 of the revised main text. For the energy window depicted by the solid line in Fig. R16a, the procedure developed in the main text determines the corresponding $\boldsymbol{\mu}_{min}$ and the associated NBFP distributions for each energy. As an example, the six NBFPs corresponding to E_b are shown as circles in Fig. R16b and are labeled sequentially as $\boldsymbol{k}_1$ to $\boldsymbol{k}_6$ with $\boldsymbol{\mu}_{min} = (0.42{,}0.42)$. Owing to the symmetry of the model, these six NBFPs are symmetrically distributed with respect to both $k_x = k_y$ and $k_x = -k_y$. Within the energy window of interest, the number and symmetry of NBFPs are the same as E_b.

In principle, any pair of NBFPs can interfere to form a standing-wave pattern along the direction connecting the two NBFPs, thereby satisfying the corresponding OBCs. To make the discussion transparent, we focus on the two high-symmetry directions, namely, $\hat{n}_1 = (\hat{x} - \hat{y})/\sqrt{2}$ and $\hat{n}_2 = (\hat{x} + \hat{y})/\sqrt{2}$.

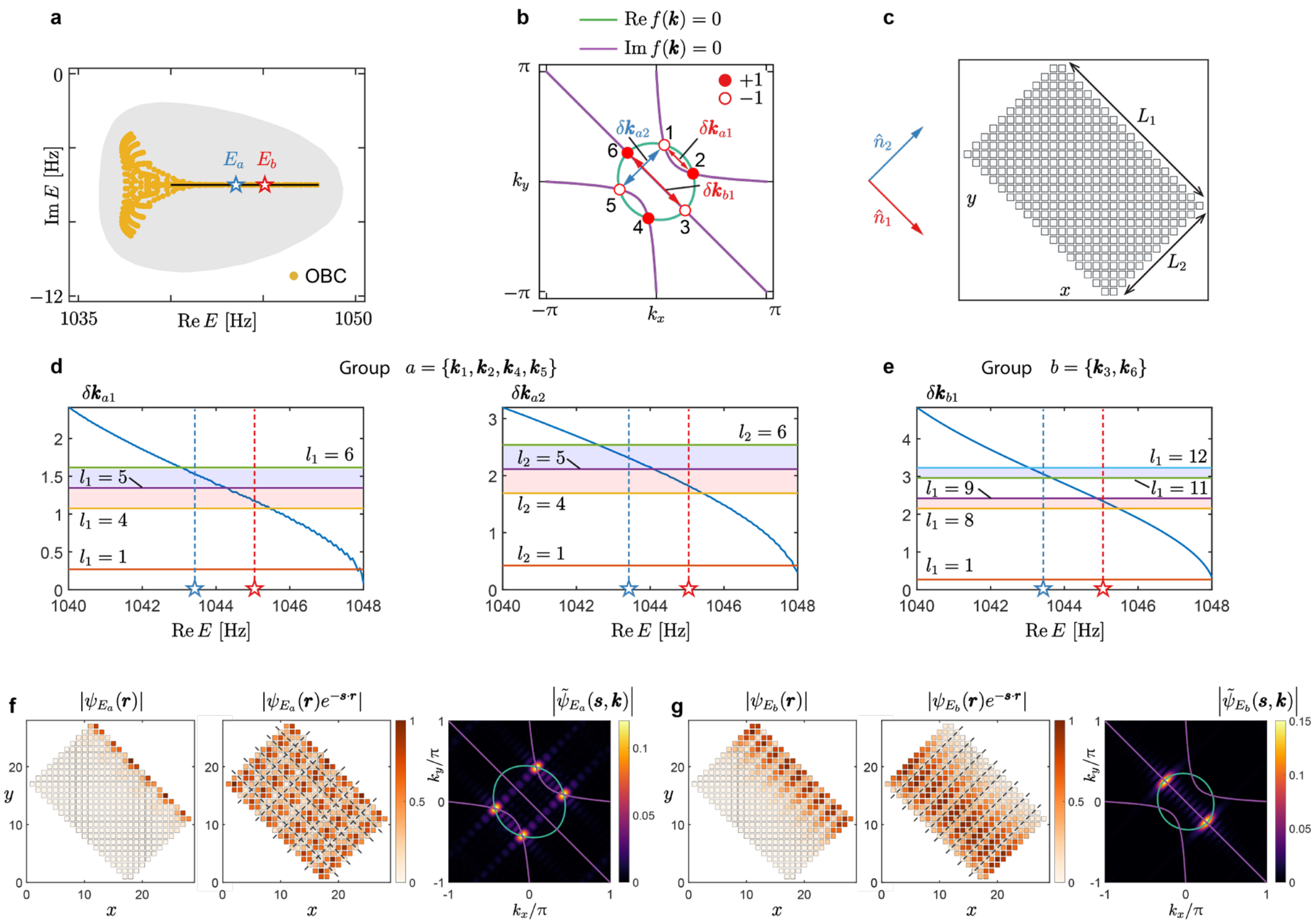

Fig. R16 | NBFP-compatible standing waves. **a**, PBC (gray) and OBC (blue) spectra with the energy window (black line), $\mathrm{Im}\, E = -6.0$ Hz and $\mathrm{Re}\, E \in [1040.0, 1048.0]$Hz. Red and blue stars mark the selected energy E_a and E_b, with the corresponding $\boldsymbol{\mu}_{\min} = (0.44, 0.44)$ and $(0.42, 0.42)$, respectively. **b**, NBFP distribution at E_b for $\boldsymbol{\mu}_{\min}$. The six NBFPs (red circles) are labeled $1 \to 6$ in clockwise order. **c**, Schematic of an OBC geometry. The two edges are oriented along $\hat{n}_1 = (\hat{x} - \hat{y})/\sqrt{2}$ and $\hat{n}_2 = (\hat{x} + \hat{y})/\sqrt{2}$, with lengths $L_1 = 16.5\sqrt{2}$ and $L_2 = 10.5\sqrt{2}$, respectively. **d**,**e**, Energy dependence of the momentum separations between the NBFP pairs in group a, $\{\boldsymbol{k}_3, \boldsymbol{k}_6\}$ (**d**), and group b, $\{\boldsymbol{k}_1, \boldsymbol{k}_2, \boldsymbol{k}_4, \boldsymbol{k}_5\}$ (**e**), i.e., δk_{a1}, δk_{b1}, and δk_{b2} defined in **b**. The horizontal lines denote the quantized momentum separations satisfying the standing-wave condition, i.e., $2\pi l_{1(2)}/L_{1(2)}$, with $l_{1(2)} \in \mathbb{N}$. The vertical dashed lines mark the selected energies E_a and E_b. The blue (red) shaded region indicates the interval in which the momentum separations corresponding to E_a (E_b) lie. **f**,**g**, Eigenstate amplitude (left), compensated wavefunctions $|e^{-\boldsymbol{s}\cdot\boldsymbol{r}}\psi_E(\boldsymbol{r})|$ with corresponding $\boldsymbol{s} = \boldsymbol{\mu}_{\min}$ (middle), and s-resolved momentum-space wavefunction $|\tilde{\psi}_E(\boldsymbol{s}, \boldsymbol{k})|$ (right) at E_a (**f**) and E_b (**g**). Dashed lines are plotted to indicate the interference nodes of $|e^{-\mathbf{s}\cdot\mathbf{r}}\psi_E(\boldsymbol{r})|$. Gray circles indicate the NBFP positions obtained from the non-Bloch supercell.

Figure R16b shows that six NBFPs are certainly three pairs, i.e., $\{\boldsymbol{k}_1, \boldsymbol{k}_2\}$, $\{\boldsymbol{k}_4, \boldsymbol{k}_5\}$, and $\{\boldsymbol{k}_3, \boldsymbol{k}_6\}$, which are protected by the symmetry with respect to $k_x = k_y$. Another symmetry with respect to $k_x = -k_y$ implies that $\{\boldsymbol{k}_1, \boldsymbol{k}_2\}$ and $\{\boldsymbol{k}_4, \boldsymbol{k}_5\}$ are correlated, and hence, the six NBFPs naturally divide into two groups, i.e., group $a = \{\boldsymbol{k}_1, \boldsymbol{k}_2, \boldsymbol{k}_4, \boldsymbol{k}_5\}$ and group $b = \{\boldsymbol{k}_3, \boldsymbol{k}_6\}$.

Group a consists of the four NBFPs $\{\boldsymbol{k}_1, \boldsymbol{k}_2, \boldsymbol{k}_4, \boldsymbol{k}_5\}$, corresponding to two symmetry-related interference channels along the $\hat{n}_1$ and $\hat{n}_2$ directions. Since the momentum separation between paired NBFPs determines the interference pattern, we define

$$\delta k_{a1} = (\boldsymbol{k}_5 - \boldsymbol{k}_4) \cdot \hat{n}_1 = (\boldsymbol{k}_1 - \boldsymbol{k}_2) \cdot \hat{n}_1, \quad \delta k_{a2} = (\boldsymbol{k}_1 - \boldsymbol{k}_5) \cdot \hat{n}_2 = (\boldsymbol{k}_2 - \boldsymbol{k}_4) \cdot \hat{n}_2,$$

as illustrated in Fig. R16b. The corresponding δk_{a1} and δk_{a2} as functions of $\mathrm{Re}\, E$ within the energy window in Fig.R16a are shown in the left and right panels of Fig. R16d, respectively. Similarly, group b consists of the NBFP pair $\{\boldsymbol{k}_3, \boldsymbol{k}_6\}$. We define

$$\delta k_{b1} = (\boldsymbol{k}_6 - \boldsymbol{k}_3) \cdot \hat{n}_1, \;\; \delta k_{b2} = (\boldsymbol{k}_6 - \boldsymbol{k}_3) \cdot \hat{n}_2 \equiv 0.$$

The corresponding δk_{b1} as a function of $\mathrm{Re}\, E$ is shown in Fig. R16e. To evaluate the possible OBC wavefunction patterns for the geometry shown in Fig. R16c, we consider the standing-wave conditions

$$\delta k \; L_1 = 2\pi l_1 \quad \text{and} \quad \delta k \; L_2 = 2\pi l_2,$$

which indicate that the corresponding interference channels can support standing-wave patterns with l_1 and l_2 interference nodes (excluding the boundary nodes) along the $\hat{n}_1$ and $\hat{n}_2$ directions, respectively. The horizontal lines in Figs. R16d and R16e correspond to the quantized values $2\pi l_{1(2)}/L_{1(2)}$ for several values of $l_{1(2)}$, thereby indicating the energy windows in which NBFP-compatible standing waves can arise.

2. Standing-wave nodes inferred from NBFPs under symmetry constraints

For the energy window of interest, there exist several possible standing wave channels, and therefore, without explicitly enforcing the boundary conditions, we cannot predict the OBC wavefunctions at a particular energy. However, examining the energy lying between distinct l_1 and l_2 values can still indicate possible standing-wave patterns, such as the presence of nodes, for an OBC geometry shown in Fig. R16c.

Taking E_a as an example, if the boundary selects group a, both corresponding δk_{a1} and δk_{a2} fall between the $l_{1(2)} = 5$ and 6 lines in Fig. R16d. Therefore, the OBC eigenstate is expected to exhibit approximately 5 to 6 interference nodes along both the $\hat{n}_1$ and $\hat{n}_2$ directions. By contrast, if the boundary selects group b, δk_{b1} falls between the $l_1 = 11$ and 12 lines in Fig. R16e, whereas $l_2 \equiv 0$. Consequently, the corresponding OBC eigenstate is expected to exhibit approximately 11 to 12 interference nodes along the $\hat{n}_1$ direction while 0 interference nodes along the $\hat{n}_2$ direction.

Figure R16f shows the corresponding OBC wavefunction $\psi_{E_a}(\boldsymbol{r})$ (left) and its compensated wavefunction $\left|e^{-\boldsymbol{s}\cdot\boldsymbol{r}}\psi_{E_a}(\boldsymbol{r})\right|$ (middle) with $\boldsymbol{s} = \boldsymbol{\mu}_{\min}$ to compensate for the exponential decay. A clear standing-wave pattern is observed, exhibiting 5 interference nodes along the $\hat{n}_1$ direction and 5 nodes along the $\hat{n}_2$ direction. This agrees well with the above prediction from NBFPs and standing waves, indicating that the OBC eigenstate is formed by the interference of the NBFP pair in group a, $\{\boldsymbol{k}_1, \boldsymbol{k}_2, \boldsymbol{k}_4, \boldsymbol{k}_5\}$, as further confirmed by the s-resolved momentum-space wavefunction $\left|\tilde{\psi}_E(\boldsymbol{s}, \boldsymbol{k})\right|$ shown in the right of Fig. R16f, whose hot spots coincide with the predicted NBFP pair. To demonstrate another NBFP group, i.e., group $b = \{\boldsymbol{k}_3, \boldsymbol{k}_6\}$, we have used another energy E_b, and the results are shown in Fig. R16g. Clearly, $\{\boldsymbol{k}_3, \boldsymbol{k}_6\}$ has been seen in the interference pattern and the s-resolved momentum-space wavefunction.

3. Experimental verification of the inferred standing-wave nodes

We now use the experimental example in Fig. 6 of the revised main text to perform the analysis above and verify the NBFP-inferred standing-wave nodes. By simply replacing the system lengths with the corresponding values of $L_1 = 9.5\sqrt{2}$ and $L_2 = 3.5\sqrt{2}$, as illustrated in Fig. R17a, we calculate interference indices for E_3 and E_4 under this OBC geometry, as shown in Figs. R17b and R17c, respectively. Figures R17d and R17e present the corresponding experimental results for E_4 and E_3. In each figure, the upper-left inset shows the measured OBC wavefunction, while the left panel shows its s-resolved momentum-space wavefunction $\left|\tilde{\psi}_E(\boldsymbol{s}, \boldsymbol{k})\right|$. The right panels display the compensated wavefunction $e^{-\boldsymbol{s}\cdot\boldsymbol{r}}\psi_E(\boldsymbol{r})$, with the experimental result shown in the upper panel and the theoretical result in the lower panel. For E_4 (Fig. R17d), the compensated wavefunction exhibits four nodes along $\hat{n}_1$ and two along $\hat{n}_2$, identifying the interference channel associated with group a (Fig. R17b), consistent with the $\left|\tilde{\psi}_{E_4}(\boldsymbol{s}, \boldsymbol{k})\right|$. By contrast, E_3 (Fig. R17e)

exhibits four nodes along $\hat{n}_1$ and none along $\hat{n}_2$, identifying the interference channel associated with group b (Fig. R17c), again in agreement with the $|\tilde{\psi}_{E_3}(\boldsymbol{s},\boldsymbol{k})|$.

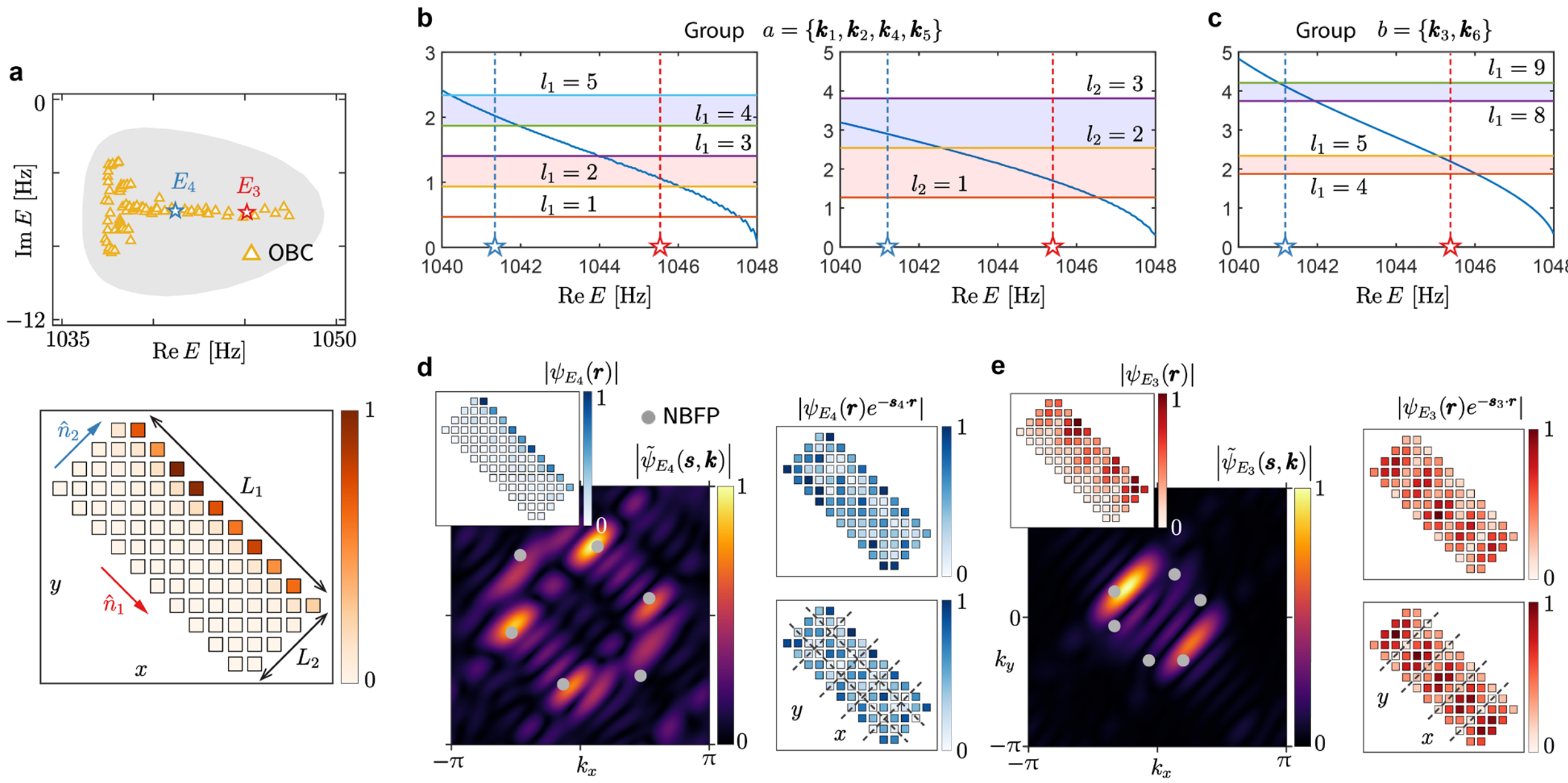

Fig. R17 | Experimental verification of NBFP-inferred standing-wave nodes. a, OBC spectra (top) and schematic of the OBC geometry (bottom) with lengths $L_1 = 9.5\sqrt{2}$ and $L_2 = 3.5\sqrt{2}$. **b**,**c**, Energy dependence (solid line in Fig. R16a) of the momentum separations between the NBFP pairs in group a, $\{\boldsymbol{k}_1, \boldsymbol{k}_2, \boldsymbol{k}_4, \boldsymbol{k}_5\}$ (**b**), and group b, $\{\boldsymbol{k}_3, \boldsymbol{k}_6\}$ (**c**), i.e., δk_{a1}, δk_{a2}, and δk_{b1} defined in Fig. R16b. The horizontal lines denote the quantized momentum separations satisfying the standing-wave condition, i.e., $2\pi l_{1(2)}/L_{1(2)}$, with $l_{1(2)} \in \mathbb{N}$. The vertical dashed lines mark the selected energies E_3 and E_4. The red (blue) shaded region indicates the interval in which the momentum separations corresponding to E_3 (E_4) lie. **d**,**e**, s-resolved momentum-space wavefunctions $|\tilde{\psi}_E(\boldsymbol{s},\boldsymbol{k})|$ (left panels) for $E = E_4$ (**d**) and $E = E_3$ (**e**). Grey circles indicate NBFP positions obtained from non-Bloch supercells. Insets show measured real-space eigenstates. The right panels display compensated wavefunctions $|e^{-\boldsymbol{s}\cdot\boldsymbol{r}}\psi_E(\boldsymbol{r})|$ for $E = E_4$ (**d**) and $E = E_3$ (**e**) with corresponding $s = \mu_{\min} = 0.47$ and 0.42. The top and bottom panels show experimental and theoretical results, respectively. Dashed lines are plotted to indicate the interference nodes of $|e^{-\mathbf{s}\cdot\mathbf{r}}\psi_E(\boldsymbol{r})|$.

In summary, while NBFPs alone do not uniquely determine the exact OBC wavefunction for a given energy and boundary geometry, their distribution, when combined with the relevant symmetry constraints, can identify NBFP-compatible standing waves and thereby suggest possible nodal patterns that can be tested experimentally.

We believe this highlights the conceptual significance of NBFPs. To clarify this point, we have revised Fig. 6c-f and added the related discussions to the *Boundary-activated non-Bloch Fermi points in 2D lattices* section (fourth paragraph on page 11) of the revised main text as follows:

"Since the NBFP localizations closely relate to the underlying symmetry, for a given boundary geometry in Fig. 6c, the selection can be understood as standing-wave interference between different NBFP pairs satisfying the boundary constraints. To reveal, we compensate for the exponential decay by applying $e^{-\boldsymbol{s}\cdot\boldsymbol{r}}\psi_E(\boldsymbol{r})$ with $\boldsymbol{s}=\boldsymbol{\mu}_{min}$, as shown in the right panels of Figs. 6e-f, where the upper and lower panels correspond to the experimental and theoretical results, respectively. For E_3 (Fig. 6e), the compensated wavefunction exhibits four nodes along $\hat{\mathrm{n}}_1$ and none along $\hat{\mathrm{n}}_2$, whereas for E_4(Fig. 6f), it exhibits four nodes along $\hat{\mathrm{n}}_1$ and two along $\hat{\mathrm{n}}_2$. These interference nodes can be inferred by the momentum separations between the corresponding NBFP pairs together with the standing-wave conditions (see Supplementary Section VIII)."

A detailed theoretical analysis of the NBFP-compatible standing waves has been added as a new Supplementary Section VIII.

Minor Comments

1. *The claim that the framework is universal implementation-wise and readily transferable across platforms should be moderated. The present realization relies on a highly programmable active acoustic architecture with engineered couplings and access to full Green's-function information, which is an impressive but also rather specialized setting.*

Response:

Thank you for raising this concern. We agree that the original wording was overly broad. The present framework indeed requires platforms with programmable hoppings and full access to Green's functions. Its generalization to other synthetic platforms, including mechanical, electrical-

circuit, microwave, and photonic systems, is straightforward. However, its application to electronic materials should be regarded as a conceptual possibility rather than an immediately realizable experimental implementation.

Accordingly, in the *Discussions and conclusions* section of the revised main text, we have removed the word "transferable" from the first paragraph and revised the outlook statement at the beginning of the third paragraph as follows:

"Beyond the specific platform demonstrated here, our framework provides a transferable experimental protocol for reconstructing non-Bloch band structures, provided that the hopping amplitudes can be programmed and the full Green's function response can be measured[30,48–51]."

2. *The language of "complete reconstruction" should also be softened somewhat. In practice, the approach still relies on spectral-potential minimization and on finite experimental access to the relevant range of μ. The present achievement is therefore better described as an important practical route toward reconstruction rather than an entirely general and complete solution.*

Response:

Thank you for this important suggestion. We agree that the original wording "complete reconstruction" could be interpreted too broadly and should therefore be moderated.

By "complete reconstruction," we mean reconstruction of the full band and eigenstate information within the experimentally accessible and physically relevant non-Bloch parameter window, rather than an unrestricted reconstruction over all $\boldsymbol{\mu}$. Within this window, our framework recovers both the complex spectrum and the corresponding biorthogonal eigenstates, as demonstrated in the non-Hermitian Chern-insulator experiment where the reconstructed left/right eigenvectors enabled measurements of Berry curvature and the Chern number. Importantly, the $\boldsymbol{\mu}$ window that determines the OBC spectrum is finite and, in the systems considered here, is fully covered by the experimental dynamic range.

To avoid ambiguity and overstatement, we have removed the word "complete" from the third paragraph of the *Introduction* and added the following clarification regarding the experimentally accessible range of μ at the beginning of the third paragraph of the *1D Non-Hermitian Band Structures, Non-Bloch Fermi Points, and Validations* section (page 7 of the revised main text):

“*Although our non-Bloch supercell implementation broadens the experimentally accessible dynamic range of* $\boldsymbol{\mu}$, *access to* $\boldsymbol{\mu}$ *remains finite. What matters is not exhaustive coverage of all* $\boldsymbol{\mu}$, *but whether the accessible range spans the finite* $\boldsymbol{\mu}$ *window that determines the OBC spectrum. Once this condition is met, the measured non-Bloch band structures contain all the information required to predict OBC behaviours. To diagnose such a* $\boldsymbol{\mu}$ *window, we* ...”

Recommendation

In summary, this is a well written and technically sound manuscript. Nevertheless, I cannot recommend it for publication in Nature Communications in its present form. A substantially revised version could become suitable if the authors sharpen the novelty claim, clarify the relation between the twisted-boundary implementation and the non-Bloch theoretical framework, and strengthen the interpretation of the two-dimensional NBFP-selection results, as detailed in the above comments.

Response:

We sincerely thank the reviewers for the careful reading of our manuscript and for all constructive and insightful comments. We have carefully considered all concerns raised in the report and have substantially revised the manuscript accordingly. To sharpen our novelty claim and especially compare with previous works, we have

- Revised the *Abstract* and *Introduction* sections to highlight our motivation, being an experimental reconstruction of non-Hermitian band structures in arbitrary dimensions, with simultaneous verification in open-boundary systems.
- Added new paragraphs to both the Boundary-activated non-Bloch Fermi points in 2D lattices and the *Discussions and conclusions* sections to illustrate the conceptual significance of NBFPs.
- Added a new paragraph to the *Discussions and conclusions* section to highlight the NBFP signatures and functionality in OBC measurements. Details are in the new Supplementary sections VIII and IX.
- Added a new Fig. 4 on the critical skin effect and a new Fig. 7 on a non-Hermitian Chern insulator, together with related discussions, to highlight the capability of our framework for experimentally accessing large-N physics and non-Hermitian topological features.

To clarify the relation between the twisted-boundary implementation and the non-Bloch theoretical framework, we have

- Revised the paragraphs in the *Non-Bloch supercell framework* section, together with an updated Fig. 2, to establish the correspondence between complex-continuation twisted boundary conditions and non-Bloch theory, followed by experimental challenges.
- Revised the Supplementary section I to compare our framework with other experimental approaches on non-Hermitian measurements to illustrate why complex-continuation twisted boundary conditions act as a bridge from non-Bloch theory to OBC analyses.

To strengthen the interpretation of the two-dimensional NBFP-selection results, we have

- Revised the paragraphs in the *Boundary-activated non-Bloch Fermi points in 2D lattices* section, together with an updated Fig. 6, to illustrate how the interference between different NBFPs determines the possible standing-wave nodes.
- Added a new Supplementary Section VIII to present a quantitative procedure for identifying NBFP-compatible standing waves and energy windows under relevant symmetry constraints.

We believe that these revisions substantially strengthen both the conceptual positioning and the physical significance of the work, and we hope that the revised manuscript now satisfactorily addresses the reviewers' concerns.

Reply to Reviewer #3

This manuscript presents a significant experimental advancement in the direct reconstruction of non-Hermitian band structures through a non-Bloch supercell framework. By decoupling the real and imaginary parts of momentum via exponent flattening, the authors achieve scale-free control over the complex momentum and high-resolution sampling of the Brillouin zone, overcoming a fundamental trade-off in previous approaches. The method is implemented in programmable one- and two-dimensional acoustic crystals, enabling the extraction of complex eigen energies, eigenstates, and non-Bloch Fermi points via Green's function measurements. The experimental results are in excellent agreement with theoretical predictions and are further validated by open-boundary measurements, including the observation of boundary-activated non-Bloch Fermi points in two dimensions. The work is timely, well-executed, and conceptually clear, offering a versatile experimental platform for exploring non-Hermitian topology. I recommend publication in Nature Communications after the authors address the following comments.

Response:

We sincerely thank the reviewer for the highly positive and encouraging assessment of our work. We are particularly encouraged that the reviewer finds the manuscript timely, technically strong, and conceptually clear. We have carefully considered all comments and suggestions, and below we provide a detailed point-by-point response.

First, while the manuscript focuses on a single point-gap topology, non-Hermitian systems can also exhibit many point gaps or line gaps. It would be helpful if the authors could clarify whether the proposed non-Bloch supercell framework is equally applicable to these non-Hermitian systems. A brief discussion on the generality of the method across different gap types would enhance its applicability.

Response:

Thank you for this valuable suggestion regarding the generality of the framework across different non-Hermitian gap structures. Our non-Bloch supercell framework is equally applicable to these non-Hermitian systems. We have added two new experiments to the revised manuscript to explicitly show its applicability to systems with multiple point gaps and line gaps.

1. Applicability of the framework to systems with multiple point gaps

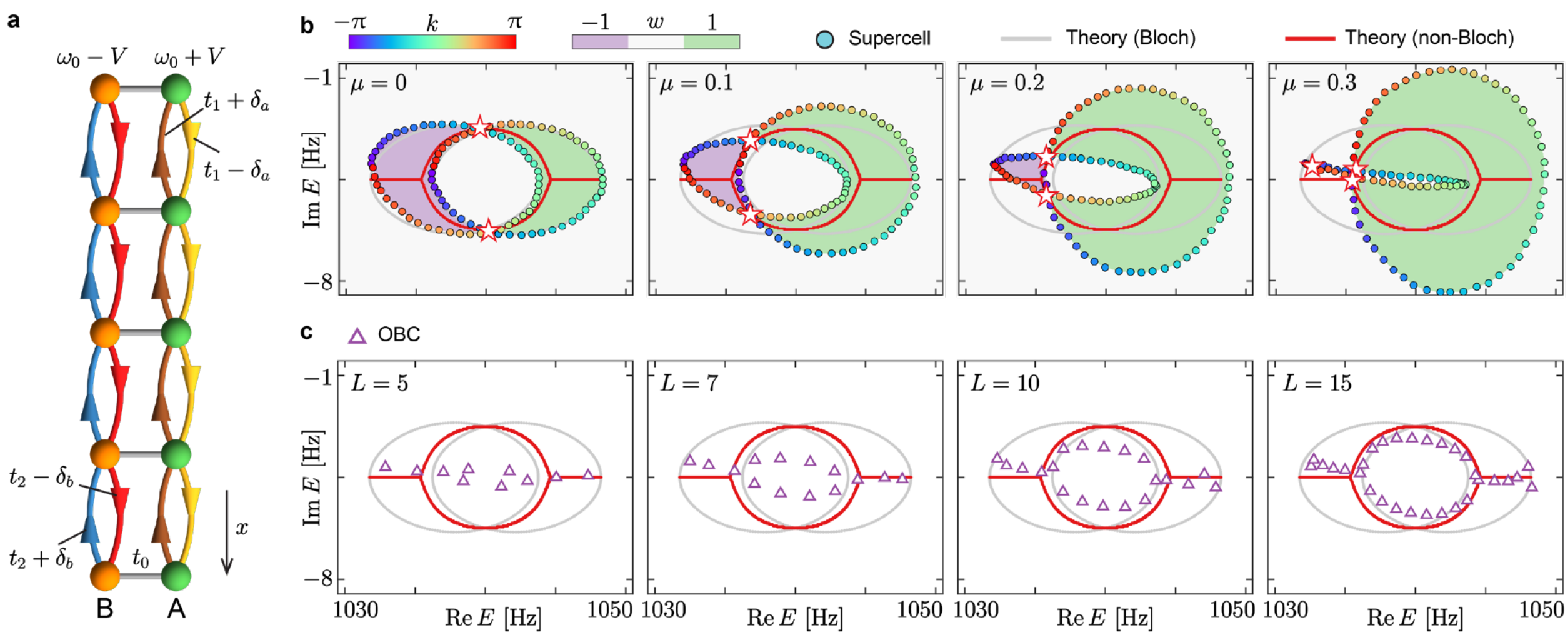

Fig. R18 | Experimental measurement of the system exhibits multiple point gaps. a, Two-chain model supporting the critical skin effect. The lattice model is $H_{\mathrm{CSE}}(\beta) = \begin{pmatrix} g_A(\beta) & t_0 \\ t_0 & g_B(\beta) \end{pmatrix}$ with $g_A(\beta) = \omega_0 + t_A^+\beta + t_A^-\beta^{-1} + V$ and $g_B(\beta) = \omega_0 + t_B^+\beta + t_B^-\beta^{-1} - V$. The two chains are labeled as A and B. **b**, Measured $E^\mu(k)$ for four selected values of μ. Filled circles with their colour encoding k are from the non-Bloch supercell framework, and their self-intersections (marked as stars) indicate the OBC spectrum in the large-N limit, as highlighted by the red stars. **c**, Measured OBC spectra (triangles) for increasing system sizes L (left to right). For comparison, the measured PBC spectra are shown as circles. In both **b** and **c**, the grey lines indicate the theoretical PBC spectrum, and the red solid lines are the OBC spectrum in the large-N limit obtained from the generalized Brillouin zone analysis. The red stars in **b** are in excellent agreement with these generalized Brillouin zone analyses, validating the non-Bloch supercell framework. By contrast, the OBC spectra in **c** deviate from the large-N limit, revealing the strong finite-size dependence characteristic of the critical skin effect. The parameters used are $\omega_0 = (1040 - 4.5i)$ Hz, $t_0 = 1$ Hz, $t_1 = 3$ Hz, $\delta_A = 1$ Hz, $\delta_B = -1$ Hz, and $V = 2$ Hz. The supercell is composed of $N_x = 5$ unit cells.

To illustrate this point, we consider the model discussed in Nature Commun. 11, 5491 (2020), shown in Fig. R18a. We first apply the non-Bloch supercell method to probe its non-Hermitian band structures. The experimentally reconstructed spectra $E^\mu(k)$ for several representative values

of μ are shown in Fig. R18b, revealing multiple intersecting point gaps. Importantly, the self-intersections (marked as stars) of these spectral manifolds agree remarkably well with the OBC spectrum in the large-N limit. This agreement demonstrates that the supercell method directly reconstructs the non-Bloch information associated with the large-N limit spectrum.

We then experimentally measure the OBC spectra of finite systems. As shown in Fig. R18c, the measured spectra exhibit the typical characteristic behavior of the critical skin effect: the OBC spectrum evolves strongly with system size and gradually approaches the red curve, which corresponds to the OBC spectrum in the large-N limit obtained from the generalized Brillouin zone analysis.

We believe this example is particularly important because it not only extends the framework to multiband systems with multiple point gaps but also directly demonstrates a key aspect of the physical necessity of the non-Bloch supercell framework. In size-sensitive non-Hermitian systems such as the critical-skin case, where the spectrum evolves with system size, measurements performed on any finite system cannot capture the full information in the large-N limit. The non-Bloch supercell framework overcomes this limitation by providing direct access to non-Bloch information beyond that available from conventional finite-size OBC spectroscopy.

2. Applicability of the framework to systems with line gaps

To address line-gap issues, we have deployed a non-Hermitian Chern insulator (Fig. R19a) that respects spectral reciprocity (Fig. R19b). Spectral reciprocity guarantees the absence of nonreciprocal skin effect in this system. Figure R18c shows the real and imaginary parts of the eigenenergies along a typical high-symmetry line. A Re E line gap is clearly observed, thereby enabling experimental investigations of the associated Chern topology and topological boundary modes.

Both left and right eigenstates are experimentally accessible within our framework, so we can directly reconstruct the Berry curvature from the measured eigenstates. Figure R19d compares the experimentally reconstructed Berry-curvature distribution (right) with the corresponding theoretical result (left) for the upper band. Integrating the Berry curvature over the Brillouin zone yields the Chern number of each band, as indicated in Fig. R19b.

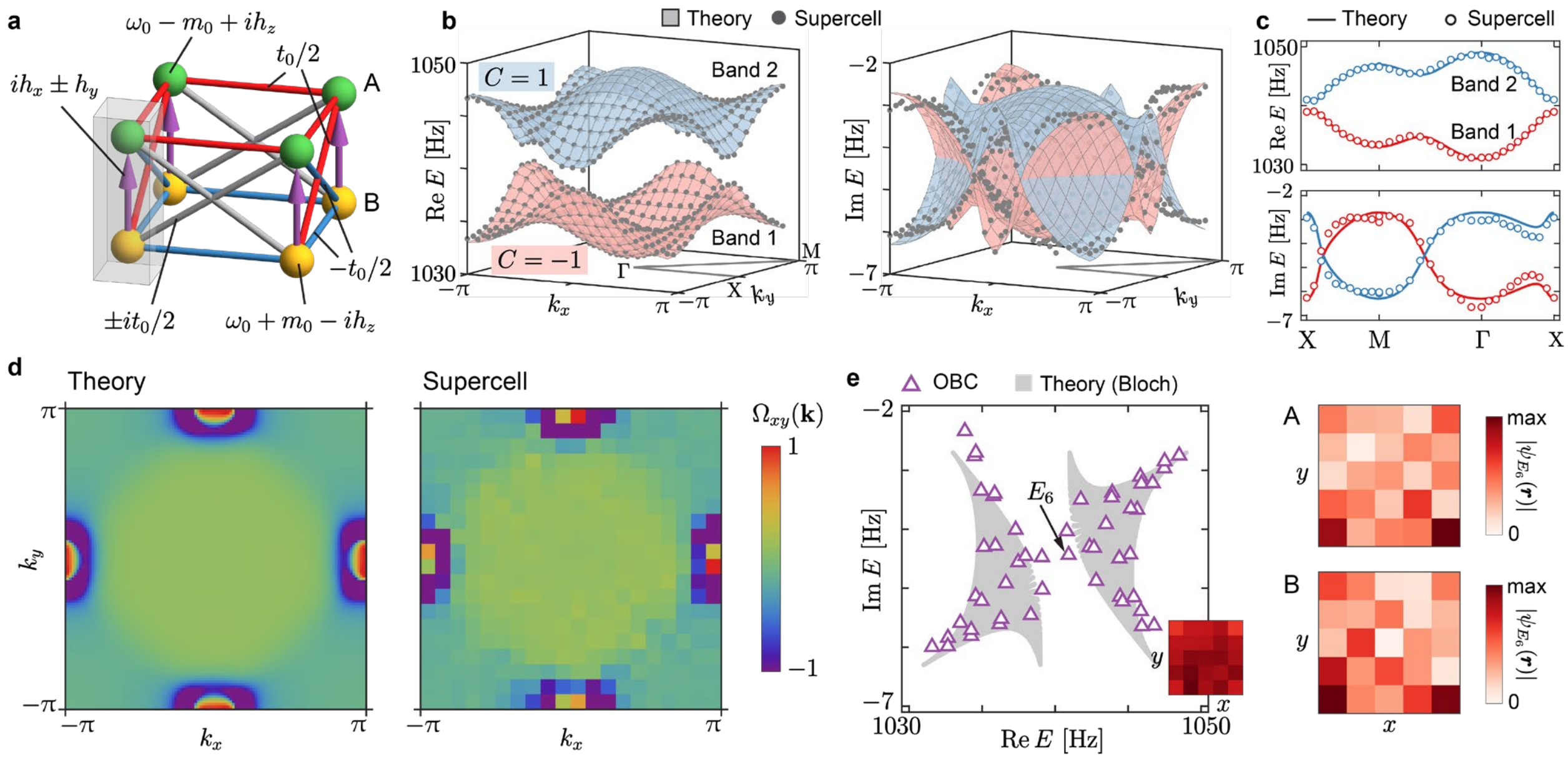

Fig. R19 | Experimental measurement of the system exhibits line gaps. a, Schematic of a 2D non-Hermitian Chern insulator. The used Hamiltonian is $H_{\mathrm{CI}}(\boldsymbol{\beta}) = \omega_0\sigma_0 + \boldsymbol{\sigma}\cdot[\boldsymbol{d}(\boldsymbol{\beta}) + i\boldsymbol{h}]$, where $\boldsymbol{d}(\boldsymbol{\beta}) = [-it_0(\beta_x - \beta_x^{-1}), -it_0(\beta_y - \beta_y^{-1}), t_0(\beta_x + \beta_x^{-1} + \beta_y + \beta_y^{-1}) - 2m_0]/2$, σ_0 is the identity matrix, $\boldsymbol{\sigma} = (\sigma_x, \sigma_y, \sigma_z)$ denotes the Pauli matrices, and $\boldsymbol{h} = (h_x, h_y, h_z) \in \mathbb{R}^3$. **b**, Measured non-Hermitian band structures $\mathrm{Re}\, E^{\boldsymbol{\mu}}(\boldsymbol{\beta})$ (left) and $\mathrm{Im}\, E^{\boldsymbol{\mu}}(\boldsymbol{\beta})$ (right) at $\boldsymbol{\mu}_{\min} = (0,0)$. The surfaces represent theoretical predictions, while the filled spheres denote experimental measurements. **c**, Band dispersions along the high-symmetry lines indicated in **b**, showing excellent agreement between theory (lines) and experiment (circles). **d**, Berry curvature distributions reconstructed from experimentally measured left and right eigenvectors (right), compared with theoretical calculations (left). **e**, OBC measurements on a finite lattice containing 50 sites. Left: OBC spectra (triangles). The gray background denotes the theoretical PBC spectra. The inset shows the spectral sum of bulk states, confirming the absence of NHSE. Right: measured real-space wavefunctions of the A (top) and B (bottom) sublattices at the selected energy E_6, corresponding to the highlighted topological edge state within the line gap. The parameters used are $\omega_0 = (1040 - 4.5i)$ Hz, $m_0 = -1$ Hz, $t_0 = 4$ Hz, $h_x = h_y = 0$ Hz, and $h_z = 1.8$ Hz. The supercell is composed of $N_x = N_y = 1$ unit cell, corresponding to the single-cell limit of the non-Bloch supercell framework.

According to the bulk-boundary correspondence, we next investigate the OBC behavior experimentally. Figure R19e shows the measured OBC spectrum (left), where topological

boundary modes emerge within the Re E line gap. The inset displays the spectral sum of the bulk states, confirming the absence of skin accumulation. The real-space wavefunctions of the A (top right) and B (bottom right) sublattices measured at the selected energy E_6, corresponding to the highlighted topological edge state within the line gap, are shown in the right panels of Fig. R18e.

Together, these two additional experiments demonstrate the broad applicability of the non-Bloch supercell framework to systems with multiple point gaps, line gaps, multiband topology, and even critical-skin behavior. To reflect this more clearly in the manuscript, we have added Fig. R18 (now Fig. 4 in the revised manuscript), together with the related discussion at the end of the *1D Non-Hermitian Band Structures, Non-Bloch Fermi Points, and Validations* section (third paragraph on page 8 of the revised manuscript), to demonstrate the capability of the framework in probing multiband systems with multiple point gaps and critical-skin behavior::

"More importantly, beyond measurement instability, OBC-based approaches are not universally reliable for probing non-Hermitian band structures, particularly in systems with significant finite-size effects, such as those exhibiting the critical skin effect (Fig. 4). Figure 4a shows a representative multiband model supporting the critical skin effect and multiple point gaps[46]. Our framework successfully captures the non-Bloch behavior in the large-N limit (Fig. 4b), which is difficult to access using conventional OBC measurements (Fig. 4c). This demonstrates that the non-Bloch supercell framework overcomes intrinsic limitations of OBC-based approaches and enables direct access to non-Bloch information in the large-N limit."

We have also added a new section entitled *Non-Bloch Band Reconstruction Beyond the Skin Effect*, together with a new Fig. 7 in the revised main text, to demonstrate the applicability of the framework to line-gap systems and, more broadly, to non-Hermitian systems beyond skin-effect physics.

Second, the current work deals with static non-Hermitian Hamiltonians. Given the growing interest in time-varying non-Hermitian systems (e.g. Phys. Rev. Lett. 135, 106601 (2025)), it is worth considering whether the proposed experimental reconstruction technique can be directly extended to such settings. A brief outlook on this direction would be valuable.

Response:

Thank you for this insightful suggestion. We agree that extending non-Bloch band reconstruction to time-dependent and Floquet non-Hermitian systems is an important and timely direction. Recent works have shown that time modulation can generate genuinely dynamical non-Hermitian phenomena beyond static Hamiltonians. For example, the harmonic non-Hermitian skin effect demonstrates that Floquet modulation can generate multiple harmonics with distinct skin morphologies governed by frequency-dependent spectral winding [Nat. Commun. 17, 2198 (2026)]. Related Floquet topological studies, including anomalous Floquet non-Abelian topological phases, further show that periodically driven systems can host quasienergy topology and boundary responses that their static counterparts lack [Phys. Rev. X 16, 011061 (2026)].

The present work focuses on static non-Hermitian Hamiltonians. Nevertheless, the key ingredients of our framework—momentum-resolved Green's-function measurements, twisted-boundary sampling, and non-Bloch momentum continuation—suggest a natural route toward future extensions to driven systems. To make this point more explicit, consider a periodically driven non-Hermitian lattice with a Hamiltonian $H(\beta,t)=H(\beta,t+T)$, where $\beta=\exp(ik+\mu)$. Expanding the Hamiltonian in temporal harmonics gives $H(\beta,t)=\Sigma_p H^{(p)}(\beta)\exp(ip\Omega t)$, where $\Omega=2\pi/T$ is the modulation frequency. Such time-dependent problems can be mapped to an enlarged time-independent Floquet Hamiltonian whose matrix elements are given by $H_{m-n}(\beta)+m\Omega\delta_{mn}$, where m and n label Floquet replicas.

Within this enlarged Floquet space, the non-Bloch continuation used in our work can be applied directly by replacing $\beta=\exp(ik+\mu)$. Equivalently, the μ-flattening strategy can be applied to each spatial hopping component. For example, a hopping term $t_l(p)$ over a spatial displacement l in the p-th temporal harmonic would be transformed into $t_l(p)\exp(-\mu l)$. The real part of the momentum can then be sampled using twisted boundary conditions at the supercell scale, while the imaginary part is controlled by the same flattening protocol introduced in the present work. In this sense, our non-Bloch supercell construction can be viewed as a spatial module that is compatible with the Floquet formulation.

We have therefore added a brief outlook discussion to the *Discussions and Conclusions* section to clarify that Floquet non-Hermitian reconstruction is a promising future direction in

which the present framework may be combined with recent advances in dynamically modulated non-Hermitian systems:

"Looking forward, the non-Bloch supercell framework can serve as a modular probe for more general non-Hermitian systems incorporating additional physical ingredients, such as temporal modulations[58,59,63]. By reconstructing non-Hermitian band structures while retaining predictive power for open-geometry responses, it provides a controllable route to characterize the spatial non-Bloch sector of such systems[60]. Combined with stroboscopic, Floquet, or dynamical measurements, this approach may enable experimental access to broader non-Hermitian phenomena, including Floquet topology and dynamical non-Bloch effects[42,51,58]."

Third, the experimental platform relies on active acoustic cavities with programmable hoppings. Given that acoustic systems inherently possess material losses and parasitic dissipation, it would be instructive to comment on how such intrinsic losses might affect the precision of the extracted complex energies and eigenstates.

Response:

We thank the reviewer for raising this important point. Intrinsic acoustic loss indeed affects the precision of the measured response, mainly by reducing the signal-to-noise ratio (SNR) and the available dynamic range of the detected fields. We provide a detailed discussion below.

In our acoustic platform, passive material loss and parasitic dissipation enter the effective Hamiltonian primarily as imaginary onsite terms. If the loss is spatially uniform, it can be written as a scalar contribution

$$H = H_{\mathrm{id}} - i\alpha_0 I,$$

where H_{id} is the ideal Hamiltonian, I is the identity matrix, and $\alpha_0 > 0$ is the uniform background loss. This term shifts all eigenenergies by the same amount, $E_n \to E_n - i\alpha_0$, but leaves the right and left eigenstates unchanged. Equivalently, with the convention $e^{-i2\pi Et}$, the field acquires a common temporal decay factor $e^{-2\pi\alpha_0 t}$. Therefore, a uniform loss in principle does not modify the extracted eigenstate profiles, complex momenta, generalized Brillouin zone, or point-gap winding structure, except for a global displacement of the spectrum along the imaginary-energy axis.

The more relevant source of experimental uncertainty is residual nonuniform loss. After separating the uniform background loss, such residual loss can be modeled as

$$H = H_{\mathrm{id}} - i\alpha_0 I - i\,\mathrm{diag}(\delta\alpha_1, \delta\alpha_2, \dots),$$

where $\delta\alpha_i$ denotes the site-dependent loss fluctuation. This term acts as an imaginary onsite disorder and can perturb both eigenenergies and eigenstates. In our implementation, this effect is mitigated in two ways. First, the isolated cavity resonances are calibrated before implementing the target Hamiltonian, and the self-couplings are used to tune the onsite frequencies and compensate for cavity-to-cavity variations. Second, the complex spectra and eigenstates are not inferred from an idealized Hamiltonian. They are reconstructed from the experimentally measured full Green's function, including both amplitude and phase responses. As a result, any residual loss remaining in the physical system is incorporated into the measured poles and residues of the Green's function rather than being neglected.

Although residual loss is incorporated into the experimentally measured Green's function, it can still affect the reconstruction accuracy indirectly through finite measurement precision. In particular, when the loss is large, the response away from the excitation site may become weak and approach the noise floor of the microphone measurement. In this situation, the measured Green's function contains uncertainties, which can invalidate the extraction of complex poles and eigenstates. Therefore, the relevant practical limitation is not the existence of loss itself, but the SNR and dynamic range with which Green's function is measured. Such an estimation has been made in our previous work [J.-X. Zhong, J. Kim, K. Chen, J. Lu, K. Ding, and Y. Jing, Experimentally probing non-Hermitian spectral transition and eigenstate skewness, Phys. Rev. B 112, L220301 (2025).] To summarize, we can assume a perturbation ΔG introduced to each measured Green's function G_{ij}

$$\tilde{G}_{ij} = \left[\left|G_{ij}\right| + |\Delta G| U(-0.5,0.5)\right] \exp\left[i(\angle G_{ij} + \angle\Delta G U(-0.5,0.5)\right],$$

where $|\Delta G|U(-0.5,0.5)$ and $\angle\Delta G U(-0.5,0.5)$ represent the magnitude and phase perturbations, respectively. Here, $U(-0.5,0.5)$ generates a random number from a uniform distribution in the range $[-0.5, 0.5]$. To quantify the effect of the loss, the measured SNR is defined as

$$\mathrm{SNR} = \frac{\left|G_{ij}\right|^2}{|\Delta G|^2} \quad \text{or} \quad 20\lg\left(\frac{\left|G_{ij}\right|}{|\Delta G|}\right) \text{ [dB]}$$

In our experiments, the typical measured SNR is higher than 40 dB over the frequency range used for spectral reconstruction.

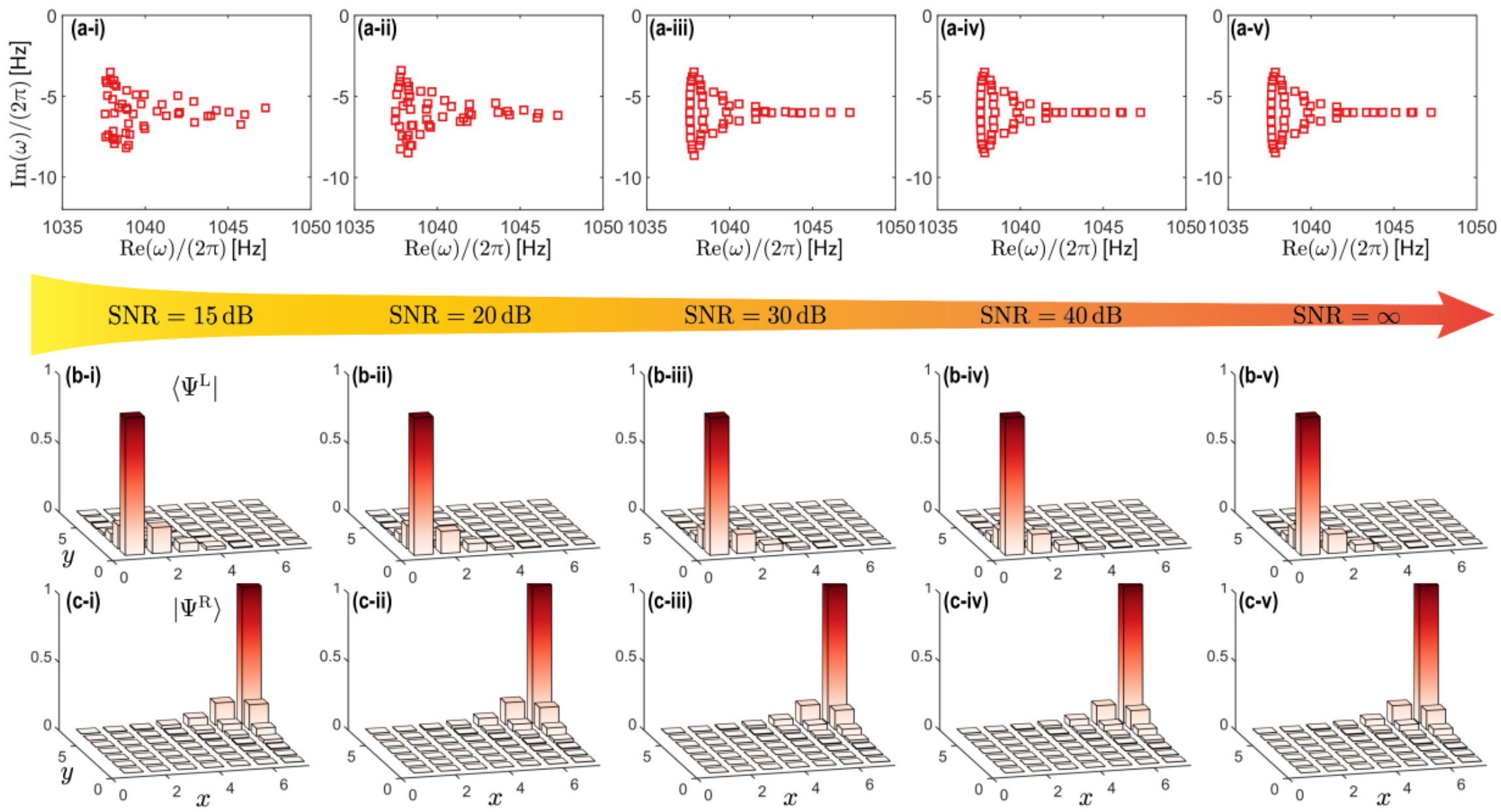

Fig. R20 | Influence of finite SNR on the reconstruction of complex spectra and eigenstates. Results are obtained for the representative non-Hermitian lattice shown in Fig. 5a of the revised main text using the Green's-function-based reconstruction method. To emulate the finite measurement precision caused by intrinsic loss and detector noise, perturbations are added to the Green's function. **a**, Extracted complex spectra under different SNR conditions. **b**,**c**, Reconstructed left and right eigenstates, respectively. The SNR values are set to (i) 15 dB, (ii) 20 dB, (iii) 30 dB, (iv) 40 dB, and (v) infinity, corresponding to the ideal noiseless case. The spectra exhibit visible fluctuations at low SNR but converge to the ideal result when the SNR exceeds 40 dB, while the eigenstate profiles remain comparatively robust.

Figure R20 shows simulated reconstructions for a representative non-Hermitian lattice, defined in Fig. 5a of the main text, under different SNR conditions. The results show that finite SNR mainly affects the extracted complex spectra, while the eigenstate distributions are comparatively more robust. At low SNR, the complex eigenenergies exhibit visible fluctuations due to noise-induced uncertainty in the Green's function poles. In contrast, when the SNR exceeds 40 dB, both the reconstructed spectra and eigenstates become nearly indistinguishable from the ideal noiseless results. This confirms that the intrinsic acoustic loss in our experiments does not significantly affect the measured non-Hermitian spectra and eigenstates within the present experimental precision.

To clarify how intrinsic losses may affect the extraction of complex eigenenergies and eigenstates, we have added the following sentence in the last paragraph of the *Non-Bloch supercell framework* section in the revised manuscript (second paragraph in page 6): "*Its robustness against intrinsic acoustic losses and other uncertainties is guaranteed by a high signal-to-noise ratio and dynamic range with which the Green's function is measured (Methods)*." We have also added the following discussion as a new section in the revised Methods:

"***Robustness of Green's-function-based spectral reconstruction***

In our acoustic platform, passive material loss and parasitic dissipation enter the effective Hamiltonian primarily as imaginary onsite terms. If the loss is spatially uniform, it can be written as a scalar contribution

$$H = H_{id} - i\alpha_0 I, \tag{8}$$

where H_{id} *is the ideal Hamiltonian,* I *is the identity matrix, and* $\alpha_0 > 0$ *is the uniform background loss. This term shifts all eigenenergies by the same amount,* $E_n \rightarrow E_n - i\alpha_0$*, but leaves the right and left eigenstates unchanged. Equivalently, with the convention* $e^{-i2\pi Et}$*, the field acquires a common temporal decay factor* $e^{-2\pi\alpha_0 t}$*. Therefore, a uniform loss in principle does not modify the extracted eigenstate profiles, complex momenta, generalized Brillouin zone, or point-gap winding structure, except for a global displacement of the spectrum along the imaginary-energy axis.*

However, the more relevant source of experimental uncertainty is residual nonuniform loss, and the complex spectra and eigenstates are reconstructed from the experimentally measured full Green's function. As a result, any residual loss remaining in the physical system can still affect the reconstruction accuracy indirectly through finite measurement precision. In particular, when the loss is large, the response away from the excitation site may become weak and approach the noise floor of the microphone measurement. In this situation, the measured Green's function contains uncertainties, which can invalidate the extraction of complex poles and eigenstates. Therefore, the relevant practical limitation is not the existence of loss itself, but the signal-to-noise ratio and dynamic range with which the Green's function is measured. In our experiments, the signal-to-noise ratio is more than 40 dB, ensuring a negligible impact on the measured results[30]."

Overall, this work represents a major step forward in the experimental characterization of non-Hermitian band structures and will be of broad interest to the community.

Response:

We sincerely thank the reviewer for this positive overall assessment and for recognizing the significance and broad interest of our work. We have carefully addressed all comments and improved the manuscript accordingly. We hope the revised version meets the reviewer's expectations.

This manuscript presents a non-Bloch supercell framework for the reconstruction of non-Hermitian band structures and its implementation in programmable one- and two-dimensional acoustic lattices. The work is technically sophisticated, the experimental platform is impressive, and the overall agreement between theory and experiment appears convincing. The introduction of non-Bloch Fermi points (NBFPs) is also well motivated, and the manuscript makes a plausible case that they organize changes in directional winding and connect to open-boundary spectral features.

Notwithstanding these strengths, I am not persuaded that the manuscript, in its present form, makes a sufficiently compelling case for publication in *Nature Communications*. My concerns pertain primarily to conceptual positioning and scope rather than to the technical execution itself.

Major Comments

1. **The novelty with respect to prior experimental work should be articulated more precisely.**

   The manuscript presents the proposed framework as a general route toward reconstructing non-Hermitian band structures. At the same time, it is stated that the approach recapitulates earlier artificial-boundary protocols in the single-cell limit. This considerably weakens the novelty claim unless the authors explain in a more explicit and precise manner what is genuinely new in the present work relative to existing synthetic-boundary, point-gap, and related non-Hermitian band-diagnostic approaches. In its current form, the distinction is not sufficiently sharp for a journal of this scope.

2. **The conceptual role of NBFPs remains insufficiently established.**

   NBFPs constitute an appealing and potentially useful construction, and the manuscript suggests that they organize discontinuous changes in directional winding. However, it remains unclear whether NBFPs should be regarded as a substantive conceptual advance in non-Hermitian band theory or primarily as an experimentally convenient reformulation of already established non-Bloch ideas. The present manuscript does not yet make this distinction sufficiently clear.

3. **The relation between twisted boundary conditions and the periodic/non-Bloch analysis requires clearer exposition.**

   The experimental implementation relies on twisted boundary conditions, whereas the theoretical interpretation is framed in terms of non-Bloch band structure and associated winding properties. The manuscript should explain this connection in a more transparent and systematic way. Since this point is central to the interpretation of the measured data as bona fide band-structure information rather than as a feature of a particular finite setup, the present discussion is not yet sufficiently clear.

4. **The strongest contribution of the work appears methodological rather than physical.**

   In my view, the most compelling aspect of the manuscript is the protocol itself: the separation of k and μ, the removal of the associated tradeoff through exponent flattening, and the experimental reconstruction of complex spectra and biorthogonal eigenstates. By contrast, the one-dimensional results mainly serve to validate that this framework reproduces the expected open-boundary spectra and localized states. This is certainly important, but for publication in *Nature Communications* the broader conceptual or physical significance enabled by the method should be articulated more convincingly.

5. **The generality of the framework should be clarified.**

   It is not clear from the current presentation whether the proposed framework can also diagnose regimes in which the skin effect is absent, or whether it is primarily effective in situations where the relevant open-boundary states already display pronounced boundary localization. This issue is important for assessing the real scope and generality of the method, and it should therefore be addressed explicitly.

6. **The two-dimensional results would benefit from a stronger predictive interpretation.**

   The observation that different open-boundary eigenstates activate different combinations of NBFPs is interesting. However, the manuscript does not yet establish whether one can predict, for a given energy and boundary geometry, which NBFPs will be selected. In the absence of such a predictive principle, the two-dimensional part reads more as an application of the method than as a broader conceptual advance of independent significance.

Minor Comments

1. The claim that the framework is universal implementation-wise and readily transferable across platforms should be moderated. The present realization relies on a highly programmable active acoustic architecture with engineered couplings and access to full Green's-function information, which is an impressive but also rather specialized setting.

2. The language of "complete reconstruction" should also be softened somewhat. In practice, the approach still relies on spectral-potential minimization and on finite experimental access to the relevant range of μ. The present achievement is therefore better described as an important practical route toward reconstruction rather than an entirely general and complete solution.

Recommendation

In summary, this is a well written and technically sound manuscript. Nevertheless, I cannot recommend it for publication in *Nature Communications* in its present form. A substantially revised version could become suitable if the authors sharpen the novelty claim, clarify the relation between the twisted-boundary implementation and the non-Bloch theoretical framework, and strengthen the interpretation of the two-dimensional NBFP-selection results, as detailed in the above comments.
